# Whole Proteome Profiling of *N*-Myristoyltransferase Activity and Inhibition Using Sortase A*[Fn FN2]

**DOI:** 10.1074/mcp.RA118.001043

**Published:** 2018-10-19

**Authors:** Andrea Goya Grocin, Remigiusz A. Serwa, Julia Morales Sanfrutos, Markus Ritzefeld, Edward W. Tate

**Affiliations:** From the ‡Department of Chemistry, Imperial College London, White City Campus, 80 Wood Lane, London W12 0BZ, UK.

**Keywords:** Chemical Biology, Drug Targets, N-terminal Modifications, Substrate Identification, Tandem Mass Spectrometry, IMP-1088, N-Myristoylation, NMT Inhibitor, Sortase A

## Abstract

*N*-myristoylation is the covalent addition of a 14-carbon saturated fatty acid (myristate) to the N-terminal glycine of specific protein substrates by *N*-myristoyltransferase (NMT) and plays an important role in protein regulation by controlling localization, stability, and interactions. We developed a novel method for whole-proteome profiling of free N-terminal glycines through labeling with *S. Aureus* sortase A (SrtA) and used it for assessment of target engagement by an NMT inhibitor. Analysis of the SrtA-labeling pattern with an engineered biotinylated depsipeptide SrtA substrate (Biotin-ALPET-Haa, Haa = 2-hydroxyacetamide) enabled whole proteome identification and quantification of *de novo* generated N-terminal Gly proteins in response to NMT inhibition by nanoLC-MS/MS proteomics, and was confirmed for specific substrates across multiple cell lines by gel-based analyses and ELISA. To achieve optimal signal over background noise we introduce a novel and generally applicable improvement to the biotin/avidin affinity enrichment step by chemically dimethylating commercial NeutrAvidin resin and combining this with two-step LysC on-bead/trypsin off-bead digestion, effectively eliminating avidin-derived tryptic peptides and enhancing identification of enriched peptides. We also report SrtA substrate specificity in whole-cell lysates for the first time, confirming SrtA promiscuity beyond its recognized preference for N-terminal glycine, and its usefulness as a tool for unbiased labeling of N-terminal glycine-containing proteins. Our new methodology is complementary to metabolic tagging strategies, providing the first approach for whole proteome gain-of signal readout for NMT inhibition in complex samples which are not amenable to metabolic tagging.

## Abstract (precis)

A new method to quantify cellular myristoylation at physiological levels and in response to *N*-myristoyltransferase inhibition is presented and validated. Sortase A is an effective tool to label protein glycine N-termini across the whole proteome, and its specificity is determined in this context. Generally applicable improvements to the biotin/avidin affinity enrichment protocol are described that effectively eliminate avidin-derived tryptic peptide contaminants.

## Graphical Abstract

**Figure:**
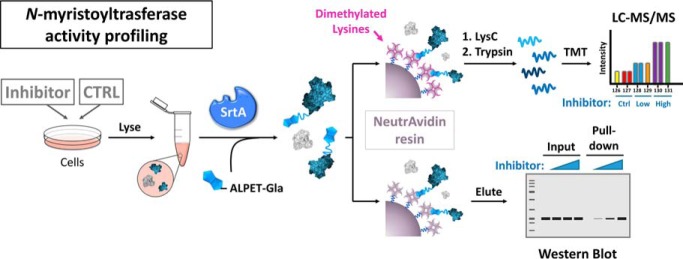

**Highlights**

- Application of Sortase A to label protein N-termini across the whole proteome.
- Novel gel, proteomic and ELISA-based methods to determine *N*-myristoylation of proteins in cells, without metabolic labelling.
- Side by side mass spectrometric quantification of changes in protein *N*-myristoylation by two complementary methods.
- Improved Biotin-Neutravidin affinity enrichment protocol.

*N*-Myristoylation involves attachment of a 14-carbon saturated fatty acid (myristate) to the N-terminal glycine of protein substrates. In humans, it is catalyzed by *N*-myristoyltransferases (NMT)^1^ 1 and 2 and occurs mostly co-translationally, resulting in an irreversible protein modification that controls protein localization, stability, and protein-protein interactions (1). With important roles in protein regulation, *N*-myristoylation has been shown to be essential in several organisms and has been linked to a wide variety of human diseases ranging from viral (2) and parasitic infections (3–5) to cancer (6, 7).

To better understand and efficiently target this important process, methods for convenient and robust assessment of NMT activity are needed. The lipophilic nature and poor antigenicity of this modification has made the identification of modification sites particularly challenging by classical mass spectrometry-based proteomics methods. We and others have applied chemically tagged myristate analogues that are cell-permeable and readily transferred by endogenous NMT onto protein substrates to allow for visual assessment of NMT activity or enrichment of tagged proteins upon chemical ligation to fluorophores or affinity reagents *in vitro* (8). Although this method has proven to be very powerful for substrate identification and NMT activity profiling in cells (8, 9), it relies on metabolic tagging with a chemically modified (*e.g.* alkynylated) analogue. Importantly, this approach is limited to systems that are metabolically active and is difficult to scale to complex *in vivo* systems because of the need to administer animal models with large amounts of analogue, which raises challenges of cost, pleotropic perturbations, and metabolic instability. Thus, zebra fish embryos are the most complex model in which comprehensive NMT substrate metabolic profiling has been reported to date (9).

*Staphylococcus aureus S*ortase A (SrtA) is a transpeptidase that catalyzes the attachment of proteins bearing a conserved LPXTG motif to the peptidoglycan cell wall in Gram-positive bacteria (10). SrtA cleaves between the threonine and glycine residues of the recognition motif and ligates it by amide bond formation to the N-terminal amine of proteins containing a free N-terminal glycine (11). Because of its purported substrate promiscuity, SrtA-mediated site-specific ligation has been very widely used for a plethora of labeling applications for molecules including peptides, proteins, glycopeptides, nucleic acids and lipid analogues bearing an unhindered N-terminal glycine (12). SrtA mutants have been engineered through directed evolution and rational design to deliver mutants with improved reaction rates and stability (13).

Here we present a novel approach for whole-proteome analysis of changes in NMT activity using an optimized SrtA mutant as a tool for enrichment and quantitative profiling of N-terminal Gly containing proteins. We demonstrate its application to measuring the response of specific NMT substrates to an inhibitor in cells across multiple cell lines, with cross-validation by in-gel and ELISA assays, and direct comparison to profiling by metabolic tagging. We also present the first profile of SrtA substrates at the proteome level and introduce an operationally simple and generally applicable optimization to improve performance of the widely-used biotin/avidin affinity enrichment protocol. We anticipate that this first method for gain-of-signal proteome profiling of NMT inhibition will be widely applicable to any protein sample, expanding the scope of analysis beyond that readily accessible to metabolic tagging.

## EXPERIMENTAL PROCEDURES

### 

#### 

##### Materials and Chemicals

Primary antibodies were purchased from Proteintech (UK) (ARL1, 16012–1-AP), Cell Signaling Technology (Germany) (PRKACA, 5842S) and Abcam (Germany) (YES1, Ab133314; GAPDH, Ab9485). Secondary antibodies were purchased from Advansta (CA) (rabbit-HRP, R-05072–500). Pierce^TM^ NeutrAvidin^TM^ agarose beads were purchased from Thermo Scientific (MA, 29201).

##### Cells

MDA-MB-231, Panc-1 and HeLa cells were purchased from the American Type Culture Collection (ATCC®) (VA) (HTB-26 TM, CRL-1469TM and CCL-2 TM respectively). Cells were cultured in low-glucose Dulbecco's Modified Medium (DMEM) (Merck, Germany, D6046) supplemented with 10% heat-inactivated fetal bovine serum (FBS; Gibco, 10270). MDA-MB-231 cells were cultured at 5% CO_2_ concentration and 37 °C and Panc-1 and HeLa were cultured at 10% CO_2_ and 37 °C.

##### Synthesis of Sortase Substrate Peptides

All reagents, amino acids and solvents for solid phase peptide synthesis (SPPS) were obtained from commercial sources (Merck, AGTC bioproducts) and were used without further purification. The protected depsipeptide amino acid Fmoc-Thr(OtBu)-OCH_2_COOH was synthesized in two solution-phase steps according to previously published procedure (14). Semi-preparative LC-MS purification was performed on a Waters system (2767 autosampler, 515 pump, 3100 ESI-MS) and HRMS was performed on a Waters LCT Premier Spectrometer operating in W mode ES +. Rink amide resin (50.0 μmol, 1 eq) was swollen in DMF (2 ml, 30 min), Fmoc deprotected with 20% v/v piperidine in DMF (2 ml, 10 min × 3) and washed sequentially with DMF, DCM and DMF. All coupling steps were carried out with HBTU/DIPEA in 2 ml DMF (30 min × 2). Specifically, Fmoc-Thr(OtBu)-OCH_2_COOH (3eq) was activated with HBTU (3eq) and DIPEA (6eq); TAMRA (2eq) with HBTU (2eq) and DIPEA (4eq); and E, P, L, A, and biotin (5eq) with HBTU (5eq) and DIPEA (10eq).

Crude products were cleaved from the resin with 95% TFA, 2.5% water and 2.5% triisopropylsilane (3 h) and precipitated with cold TBME. The solids were pelleted by centrifugation (15 min, 4300 rpm, 4 °C) and washed three times with TBME. Pelleted products were dried and purified (≥ 95%) by semi-preparative LC-MS equipped with Waters X Select C18 columns running a gradient of MeOH (0.1% FA) in water (0.1% FA) (5–98%, 30 min for the biotin derivative and 20–98%, 30 min for the TAMRA derivative). The products were obtained by lyophilization as bright pink (in case TAMRA derivative) or off-white amorphous solid (in case of biotin derivative). TAMRA-ALPET-Haa was obtained in 46.5% yield (23.2 mg) and Biotin-ALPET-Haa in 32.6% yield (13.3 mg). Purified compounds were characterized by HRMS. The main [M+H]^+^ peak found for TAMRA-ALPET-Haa was of *m*/*z* = 813.3799 (calc. 813.3817) and of *m*/*z* = 999.4451 (calc. 999.4451) for Biotin-ALPET-Haa.

##### Sortase A Reaction

MDA-MB-231, Panc-1 or HeLa cells were seeded in 100 mm dishes 1 day before the start of the experiment and treated next day with DMSO, 1 nm, 10 nm or 100 nm IMP-1088 NMT inhibitor. 24 h later, cells were washed with 1X PBS and scraped in SrtA buffer (50 mm TRIS pH 7.5, 150 mm NaCl, 10 mm CaCl_2_, EDTA-free protease inhibitor mixture (Roche, Germany)). Cell suspensions were transferred to microcentrifuge tubes, lysed by passing through a 21-gauge needle and protein concentration measured using the DC^TM^ Protein Assay Kit II (BioRad, CA). Samples (20–300 μg protein content) were adjusted to 1 mg/ml protein concentration in SrtA buffer. The SrtA reaction was carried out by adding 75 μm ALPET-Haa substrate and 0.1 μm SrtA pentamutant (a kind gift from Dr. M. Jamshidiha, Imperial College London, expressed and purified from Addgene (MA) plasmid 86962 (15)) to each sample and incubating with mild shaking overnight (16 h) at 4 °C. The reaction was stopped by addition of 5 mm EDTA. Proteins where then precipitated by addition of 4:1:2 methanol to chloroform to lysate ratio and washed once with methanol. Samples were re-suspended in 2% SDS, 10 mm DTT. Once dissolved, the samples were diluted to a final concentration of 1 mg/ml of proteins and 0.2% SDS by addition and resuspended in 1/10 of the initial volume of PBS containing 2% SDS and 10 mm DTT and then diluted to 1 mg/ml and 0.2% SDS.

##### Affinity Enrichment and Western Blot Analysis

Samples were labeled by SrtA overnight and precipitated as described above. For the affinity enrichment, 50 μl of NeutrAvidin agarose resin slurry, previously equilibrated with 0.2% SDS in PBS, were used for 50 μg of SrtA-labeled protein sample. Samples were incubated with the beads for 2 h at RT. The nonbound solution was collected to be used as the *supernatant fraction*. The beads where then washed three times with 0.2% SDS/1× PBS before elution in 2× protein loading buffer for 15 min at 95 °C. Typically, proteins enriched from 50 μg of protein were eluted in 20 μl 2× protein loading buffer. Equal volumes of every sample were loaded into 12% polyacrylamide gels and run for 10 min at 100 V followed by 55 min at 180 V. Gels were transferred by wet transfer to nitrocellulose membranes for 1 h at a constant voltage of 100 V. The membranes were briefly incubated in Ponceau staining to assess the quality of the transfer before being cropped according to the needs. Membranes were then blocked for 1 h at RT in 5% (m/w) semi skimmed milk in TBST before incubation with primary antibodies (ARL1, PRKACA, YES1 or GAPDH) overnight at 4 °C. Membranes were washed three times in TBST for 10 min, incubated with the corresponding secondary antibody for 1 h at RT and washed three times with TBST. The membranes were developed with 200 μl Luminata^TM^ Crescendo Western HRP substrate (Merck) and imaged using an ImageQuant^TM^ LAS 4000 scanner (GE Healthcare Life Sciences, IL).

##### In-gel Fluorescence

Samples were subjected to SrtA reaction, precipitated and resuspended as described above. Protein loading buffer was added, and samples loaded into 12% polyacrylamide gels. Gels were run for 10 min at 100 V followed by 55 min at 180 V and scanned using a Typhoon FLA 9500 imager (GE Healthcare Life Sciences). For TAMRA, 532/575 nm excitation/emission wavelengths were used. For the ladder, Cy5 fluorescence settings were used, with 635/670 nm excitation/emission wavelengths.

##### Streptavidin-shift Assay

Samples were subjected to SrtA reaction, precipitated and resuspended as described above. Protein loading buffer was added, and protein samples boiled for 5 min at 95 °C. The samples were cooled down to room temperature before addition of 1 μl of 100 μm Streptavidin stock for every 5 μg of SrtA-labeled protein lysate. Streptavidin was allowed to bind to biotinylated proteins for 5 min at RT before loading the protein samples to 12% polyacrylamide gels. Gels were run for 10 min at 100 V followed by 55 min at 180 V. The gels were transferred and blotted as described in the Affinity Enrichment and Western Blot Analysis section.

##### ELISA

MDA-MB-231 cells were treated with DMSO, 1 nm, 10 nm or 100 nm IMP-1088 NMT inhibitor for 24 h before lysis in SrtA buffer. Samples were subjected to SrtA reaction, precipitated and resuspended to a final concentration of 0.02 mg/ml in 1× PBS containing 0.2% SDS. Pierce® streptavidin coated black ELISA 96-well plates (ThermoScientific) were washed four times for 3 min with wash buffer (25 mm Tris-HCl pH 7.2, 50 mm NaCl, 0.1% (w/v) BSA, 0.05% (v/v) Tween®20). All subsequent washing steps were performed in the same way. 50 μl of the SrtA-labeled protein sample containing 1 μg protein (0.02 mg/ml) were added and incubated for 3 h at RT. The plate was then washed and incubated with 100 μl ARL1 primary antibody at 1:500 dilution for 1 h at RT. After washing, the HRP-conjugated anti-rabbit secondary antibody was added in 100 μl and 1:8000 dilution, and incubated for 1 h at RT. The plate was washed, and the kinetics of substrate conversion measured on an EnVision Xcite 2104 (Perkin Elmer, MA; excitation filter: 320 nm, emission filter: 460 nm) every minute for 30 min after addition of the QuantaBlu^TM^ fluorogenic peroxidase substrate (ThermoScientific).

### Protein-based Enrichment Proteomics

#### 

##### Experimental Design and Statistical Rational

We compared protein-level enrichment of MD-MB-231 triple-negative breast cancer cell proteomes labeled with SrtA post-lysis to metabolically tagged cells with YnMyr after 24 h exposure to DMSO, 1 nm or 100 nm IMP-1088 NMT inhibitor (2). Three biological replicates were used per condition, making up a total of 18 samples (9 labeled with SrtA and 9 metabolically tagged with YnMyr). Each set of 9 samples was multiplexed by TMT labeling (using nine out of the ten channels of 10-plex TMT) and fractionated into 6 different fractions. In both cases, one of the 129 channels was excluded, as it has previously been shown that that is one of the pairs that suffers greatest reporter ion coalescence issues (16). Fractions were merged together for database searches. Average values and standard deviations were calculated across biological replicates for each treatment type (DMSO control, 1 nm or 100 nm IMP-1088) and labeling method (SrtA *versus* YnMyr). Proteins differentially enriched across treatments were determined by one-way ANOVA, allowing for 0.01 false-discovery rate (FDR) calculated by random permutations, which allows efficient control of FDR regardless of sample distribution. To maximize the identification of changes of true biological relevance, a log_2_ fold-change cut-off threshold of 1 was used.

##### Preparation of SrtA-labeled Samples

Cells were harvested and lysed in SrtA buffer. 200 μg protein per sample were subjected to SrtA reaction and precipitated as described above. Protein samples were resuspended in 0.2% SDS, 1 mm DTT, 50 mm Tris pH 8.0 to 1 mg/ml. Bead derivatization: NeutrAvidin agarose beads (1 μl per μg of SrtA-labeled protein) were washed five times with 5-bead volumes of 100 mm TEAB pH 8.0 to remove all traces of possible free-amines, and lysines on NeutrAvidin di-methylated with 5-bead volumes of 0.2% formaldehyde and 25 mm sodium cyanoborohydride in 100 mm TEAB pH 8.0 for 1 h at RT. The reaction was quenched with 100 mm TEAB pH 8.0 containing 1% ethanolamine. Excess ethanolamine was washed away with 50 mm Tris pH 8.0 and beads equilibrated to 0.2% SDS/50 mm Tris pH 8.0. Protein-based enrichment and two-step on-bead/off-bead digestion: Samples were added to the derivatized beads and incubated for 2 h at RT. The supernatant was discarded, and the beads washed twice with 0.2% SDS/50 mm Tris pH 8.0 and three times with 50 mm Tris pH 8.0 to wash out the SDS. Bound proteins were partially digested by incubation with 0.4 μg LysC (Promega, WI, V1671) in 50 mm Tris pH 8.0 for 1h at 37 °C. The supernatant containing the cleaved proteins was then transferred to a new tube for full digestion of the proteins with 0.5 μg trypsin (Promega, V5111) overnight at 37 °C in the presence of 5 mm TCEP and 10 mm chloroacetamide. Buffer exchange on stage tips: Samples were acidified with 0.5% (v/v) trifluoroacetic acid (TFA) and loaded onto stage tips containing three SDB-XC poly(styrenedivinyl-benzene) copolymer discs (Merck). The stage tipping procedure was carried out as described previously (17). Peptide samples were eluted in 60% acetonitrile in water and the solvent removed by incubation in a Savant SPD1010 SpeedVac® Concentrator (Thermo Scientific) at 45 °C. 9-plex TMT labeling: The TMT reaction was performed by adding 0.08 mg TMT reagent (Thermo Fisher Scientific, MA) dissolved in 15 μl acetonitrile to each of the samples resuspended in 15 μl 50 mm HEPES pH 8.0 and incubating the mixture for 2 h at RT. Each reaction was quenched with 1 μl 5% hydroxylamine before combining all nine conditions into one tube. The sample was dried by centrifugal evaporation at 45 °C. Desalting and fractionation: The peptide sample was first desalted on a stage tip and dried as described previously. Samples were resuspended in 150 μl 1% (v/v) TFA/H_2_O and loaded into a second stage tip containing SCX polystyrene-divinylbenzene copolymer modified with sulfonic acid (Supelco) and separated into six fractions as reported previously (18) with slight modification in the composition of the fraction solutions (supplemental Table S1). Samples were evaporated to dryness in a Savant SPD1010 SpeedVac® Concentrator at 45 °C. Peptide fractions were dissolved in LC-MS grade H_2_O containing 2% (v/v) acetonitrile and 0.5% (v/v) TFA. 1/6 of fractions 1 and 2, 1/9 of fractions 3 and 4, and 1/12 of fractions 5 and 6 were injected into the LC-MS/MS system. Peptide samples were measured on a QExactive mass spectrometer coupled to an easy-spray source and an easy-nLC system (all Thermo Fisher Scientific).

##### Preparation of Samples Metabolically Tagged with YnMyr

Cells were lysed in lysis buffer (1% Triton, 0.1% SDS, 1× PBS) and protein concentration adjusted to 1 mg/ml. 400 μg of protein were ligated to the AzRB capture reagent by copper-catalyzed alkyne-azide cycloaddition (CuAAC) as described previously (8). Briefly, the click reagent mixture was prepared by mixing 1 μl of 10 mm capture reagent (AzTB), 2 μl of 50 mm CuSO4, 2 μl of 50 mm TCEP and 1 μl of 10 mm TBTA. 24 μl of the click mix were added and the samples incubated for 1 h at RT. The click reaction was quenched by addition of 5 mm EDTA followed by chloroform/methanol precipitation. Protein samples were resuspended in in 50 mm HEPES pH 8.0 containing 0.2% SDS and 1 mm DTT. Protein-based enrichment and on-bead digestion: 8 μl NeutrAvidin agarose beads and 32 μl agarose blank beads (Pierce® Control Agarose Resin, ThermoScientific) were used for every sample. The beads were equilibrated with 0.2% SDS in 50 mm HEPES pH 8.0 before incubation with the samples (2 h, RT). The supernatant was discarded, and the beads washed twice with 0.2% SDS in 50 mm HEPES pH 8.0, and three times with 50 mm HEPES. Samples were then resuspended in 50 μl HEPES buffer containing 5 mm TCEP and 10 mm chloroacetamide and 0.5 μg trypsin added for overnight on-bead digestion at 37 °C. 9-plex TMT labeling, desalting, and fractionation and measurement of the samples by LC-MS/MS was performed as described for SrtA samples.

##### nLC-MS/MS Data Acquisition

Peptides were separated on an Acclaim PepMap RSLC column 50 cm × 75 μm inner diameter (Thermo Fisher Scientific) using a 3 h acetonitrile gradient in 0.1% aqueous formic acid at a flow rate of 250 nl/min. Easy nLC-1000 was coupled to a QExactive mass spectrometer via an easy-spray source (all Thermo Fisher Scientific). The QExactive was operated in data-dependent mode with survey scans acquired at a resolution of 70,000 at *m*/*z* 200. Scans were acquired from 350 to 1800 *m*/*z*. Up to 10 of the most abundant isotope patterns with charge +2 or higher from the survey scan were selected with an isolation window of 1.6 *m*/*z* and fragmented by HCD with normalized collision energy of 31. The maximum ion injection times for the survey scan and the MS/MS scans (acquired with a resolution of 35,000 at *m*/*z* 200) were 20 and 120 ms, respectively. The ion target value for MS was set to 10^6^ and for MS/MS to 2 × 10^5^, and the intensity threshold was set to 1.7 × 10^3^.

##### Protein Database Search and TMT-labeling Quantification

Raw files were uploaded into MaxQuant (version 1.6.1.0) (19) and searched against the curated Swiss-Prot human proteome (with isoforms) (UniProt; December 2017; 42,326 entries) (20) using the built-in Andromeda search engine. Cysteine carbamidomethylation was selected as a fixed modification and methionine oxidation and acetylation of protein N terminus as variable modifications. For in silico digests of the reference proteome, the following peptide bond cleavages were allowed: arginine or lysine followed by any amino acid (a general setting referred to as Trypsin/P). Up to two missed cleavages were allowed. The false discovery rate was set to 0.01 for peptides, proteins, and sites. Other parameters were used as preset in the software (maximal mass error = 4.5 ppm and 20 ppm for precursor and product ions, respectively, minimum peptide length = 7, minimum razor + unique peptides = 2, minimum scores for unmodified and modified peptides = 0 and 40, respectively). “Match between runs” option (time window 0.7 min) was allowed and “Unique and razor peptides” mode was selected to allow identification and quantification of proteins in groups (razor peptides are uniquely assigned to protein groups and not to individual proteins), and for TMT quantification (MS2 mode) the minimal ratio count = 2 was selected.

##### Data Analysis

Data analysis was performed using Perseus (version 1.6.0.2) (18). MaxQuant proteinGroups.txt output files were filtered against contaminants, reverse and proteins identified only by site. Base 2 logarithm was applied to all measurements and the median values within each column (TMT channel) subtracted. Protein groups with at least three valid values were kept. Protein groups were annotated with previous evidence of myristoylation and nature of the second amino acid position (Gly or other) according to their isoform protein ID. An ANOVA test (Permutation-based FDR = 0.01; S0 = 1) was applied to all proteins in the dataset and results analyzed according to their statistical significance and myristoylation evidence (8, 9, 20).

### Peptide-based enrichment proteomics of SrtA-labeled samples

#### 

##### Experimental Design and Statistical Rational

To study the substrate specificity of SrtA in whole-cell lysates we compared enrichment of labeled peptides of MDA-MB-231 cell lysates to peptides from control (SrtA-untreated) samples. To increase the chances of identification of NMT substrates, cells were incubated in the presence of 100 nm IMP-1088 NMT inhibitor for 24 h before lysis in SrtA buffer. Three biological replicates were used, from which two 500 μg aliquots were separated, resulting in two identical sets of triplicates, one of which was subjected to a normal overnight SrtA reaction whereas the second set constituted a negative control in which incubation was carried out in the absence of enzyme. The search for modified peptides was performed using a custom-modified database to include the peptidic modification at the N terminus of every protein in the database. The aim of this experiment was identification rather than quantification, so no statistical analysis was performed.

##### Sample Preparation

IMP-1088 treated MDA-MB-231 cell lysate pairs were subjected to SrtA labeling in the presence or absence of SrtA. All reactions were quenched by addition of 5 mm EDTA, followed by chloroform/methanol precipitation and two washes with methanol. Trypsin digestion: Each protein sample was resuspended in 500 μl of 50 mm HEPES pH 8.0 containing 5 mm TCEP and 10 mm chloroacetamide, to which 4 μg of trypsin where added for overnight digestion, reduction and alkylation at 37 °C. Trypsin was quenched with the addition of 1X protease inhibitor mixture. Peptide-based enrichment: 500 μl NeutrAvidin agarose bead slurry were used for each sample containing 500 μg of protein sample. Beads were equilibrated with 50 mm HEPES pH 8.0, before incubation with the biotinylated peptide sample for 2 h at RT. The unbound fraction was discarded, and the beads washed twice with 50 mm HEPES pH 8.0, twice with H_2_O, and three times with 10% acetonitrile in H_2_O. Bead-bound peptides were eluted in two 15-min-incubations with 150 μl of 80% acetonitrile,0.1% TFA and 0.2% formic acid as described previously (20) and pooled. Enriched samples were dried in a centrifugal evaporator at 45 °C. Stage tip desalting was carried out as described above for protein-based enrichment sample preparation. Measurement of the samples by LC-MS/MS: Samples were resuspended in LC-MS grade H_2_O containing 2% (v/v) acetonitrile and 0.5% (v/v) TFA. 30% of the peptide samples were injected into the LC-MS/MS.

##### nLC-MS/MS Data Acquisition

Peptides were separated using an Acclaim PepMap RSLC column 50 cm × 75 μm inner diameter (Thermo Fisher Scientific) using a 2 h acetonitrile gradient in 0.1% aqueous formic acid at a flow rate of 250 nl/min. Easy nLC-1000 was coupled to a QExactive mass spectrometer via an easy-spray source (all Thermo Fisher Scientific). The QExactive was operated in data-dependent mode with survey scans acquired at a resolution of 70,000 at *m*/*z* 200. Scans were acquired from 350 to 1650 *m*/z. Up to 10 of the most abundant isotope patterns with charge +2 or higher from the survey scan were selected with an isolation window of 2.0 *m*/*z* and fragmented by HCD with normalized collision energy of 25. The maximum ion injection times for the survey scan and the MS/MS scans (acquired with a resolution of 17,500 at *m*/*z* 200) were 20 and 120 ms, respectively. The ion target value for MS was set to 10^6^ and for MS/MS to 10^5^, and the intensity threshold was set to 8.3 × 10^2^.

##### Protein Database Search

The search was performed in MaxQuant (v.1.6.1.0) (19) using the built-in Andromeda search engine against the curated Swissport human proteome (with isoforms) (UniProt; December 2017; 42,326 entries) (20), modified using R (21) so that the first two “MX” residues of every protein sequence were replaced by “MALPETX”. This allows for the automated assignment of ions resulted from HCD-induced fragmentation of the peptidic element of the N-terminal modification. We further allowed a biotin modification at the N terminus of any protein (formula: H_14_O_2_C_10_N_2_S; exact mass: 226.0776 Da) that we set as a variable modification to act as an internal control that would reinforce the validity of the identification by combination of the two independently found elements of Biotin-ALPET. Cysteine carbamidomethylation was set as fixed modification and methionine oxidation as variable modification. For in silico digests of the reference proteome Trypsin/P was selected (cleavage after arginine or lysine followed by any amino acid), allowing up to two missed cleavages. The false discovery rate was set to 0.01 for peptides, proteins, and sites. The minimum scores for unmodified and modified peptides were set to 0 and 25, respectively. Other parameters were used as preset in the software (maximal mass error = 4.5 ppm and 20 ppm for precursor and product ions, respectively, minimum peptide length = 7, minimum razor + unique peptides = 1). Identification of second peptides was allowed and “Unique and razor peptides” mode was selected to allow identification and quantification of proteins in groups (razor peptides are uniquely assigned to protein groups and not to individual proteins.

##### Data Analysis

Data analysis was performed using Perseus (version 1.6.0.2) (22), into which MaxQuant peptides.txt output file was loaded, and data were filtered against contaminants and reverse identifications. Only peptides with at least 2 out of 3 valid values were kept. Only peptides containing the N-terminal biotinylation followed by the ALPET- sequence were kept. To generate sequence logos all input sequences must have the same length. Therefore, each identified peptide was annotated with the first 10 amino acid sequence of the protein isoform it had been mapped to. To ensure quality of the sequence analysis, every single peptide spectrum was inspected manually, leaving only the spectra that showed at least three b- and y-ions supporting peptide backbone fragmentation in the sequence region following the added “ALPET.” Peptides unique to a protein group were kept and in cases were peptides matched to several proteins within a protein group, the first 10 amino acid sequences of all matched proteins were inspected manually, only to keep the IDs that contained a single, unambiguous N-terminal amino acid sequence across matching proteins within the group. N-terminal 10 amino acid sequence duplicates were removed before creating N-terminal sequence logos using iceLogo (23).

## RESULTS

### 

#### 

##### Labeling N-terminal glycines in whole-cell lysates with S. aureus SrtA pentamutant

**Fig. 1.**
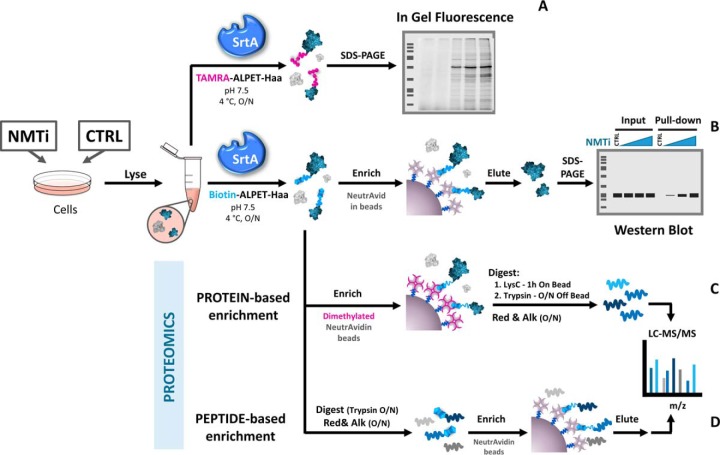
**SrtA labeling workflows enable multiple detection methods for NMT activity.** Protein samples are resuspended in SrtA reaction buffer, here exemplified by cells from tissue culture; however, in principle this approach is equally applicable to tissues exposed to a change in NMT activity. *A*, Overnight reaction with SrtA and TAMRA modified depsipeptide substrate (TAMRA-ALPET-Haa) enables in-gel fluorescence analysis of the SrtA-labeled protein lysates. Alternatively, samples subjected to SrtA reaction with biotin-modified depsipeptide substrate (Biotin-ALPET-Haa) are enriched and studied by in-gel analysis (*B*) or proteomics approaches (*C* and *D*), following dimethylation of NeutrAvidin-coated beads in the case of protein-based enrichment.

**Fig. 2.**
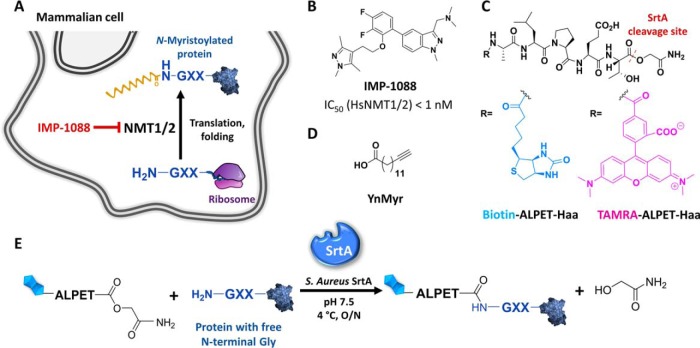
**Chemical tools for SrtA profiling of inhibition of NMT activity in cells.** *A*, Depiction of the *N*-myristoylation reaction by NMT in the cell. *B*, Structure and IC_50_ for human NMT inhibitor IMP-1088 (2). *C*, Structures of SrtA ALPET-Haa depsipeptide substrates used in this study (biotin tag in blue and TAMRA fluorophore in pink). *D*, Structure of alkynylated myristic acid analogue YnMyr. *E*, Schematic representation of the SrtA labeling reaction.

SrtA has been extensively used to ligate engineered peptide or protein substrates containing an LPXTG recognition sequence and an N-terminal Gly at a physiological pH (12, 24). Here we aimed to extend this ligating capability to label free N-terminal glycine-containing proteins in whole-cell lysates with a tagged (fluorophore/biotin) LPXTG depsipeptide SrtA substrate. To optimize labeling with SrtA in whole-cell lysates, we generated a high-activity *S. aureus* SrtA pentamutant (15, 25) and a synthetic TAMRA-ALPET-Haa depsipeptide (Haa = 2-hydroxyacetamide), and tested a range of concentrations through an in-gel fluorescence workflow (Fig. 1, supplemental Fig. S1*A* and S1*B*). Proteome samples were generated from MDA-MB-231 breast cancer cells treated with a potent and specific NMT inhibitor (IMP-1088, structure in Fig. 2) (2) to generate free N-terminal Gly residues at sites of *N*-myristoylation, before lysing with SrtA reaction buffer. Based on these results, 100 nm SrtA and 75 μm substrate depsipeptide were selected for subsequent labeling experiments.

**Fig. 3.**
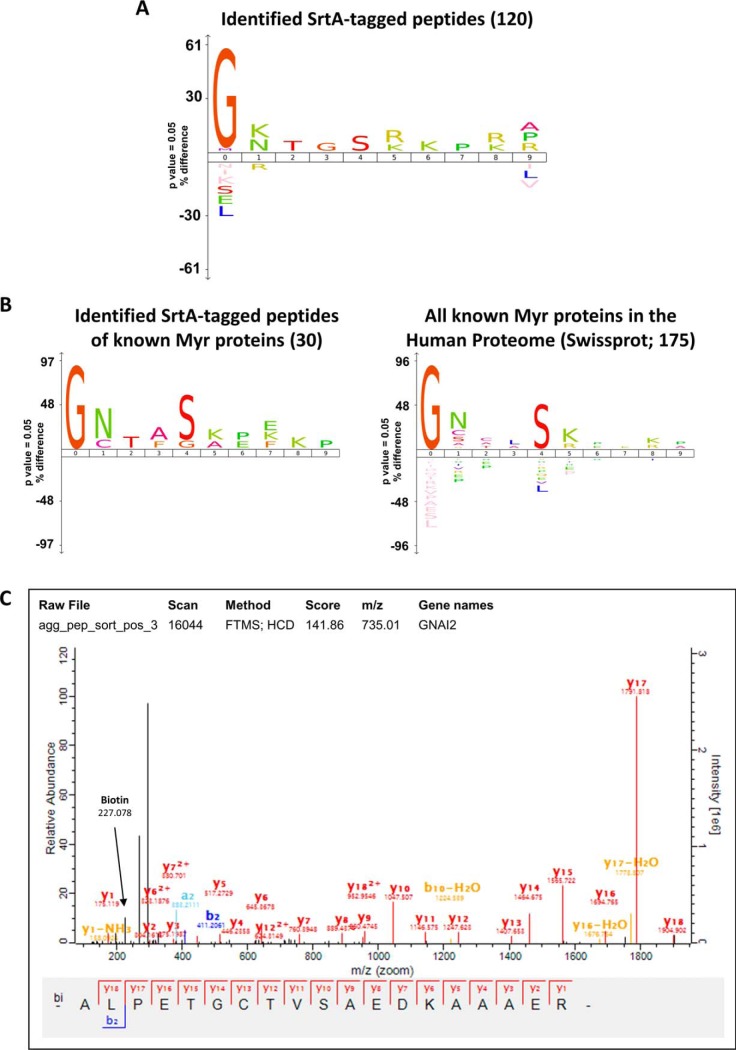
**SrtA selectively labels N-terminal Gly containing proteins in complex cell lysates.** *A*, Sequence analysis using iceLogo (23) of the N-terminal first 10 amino acids of the 120 SrtA-modified peptides identified by nanoLC-MS/MS after peptide-level enrichment of SrtA-labeled peptides. The frequencies of residues at each position were compared with the frequencies at that position in the SwissProt human proteome (pre-compiled in iceLogo, accessed on 17^th^ September 2018*). B*, Side-by side sequence analyses of known NMT substrates identified containing the SrtA-labeling modification, and of all known human myristoylated proteins. Both logos were generated against the Swiss-Prot human proteome reference data set (pre-compiled in iceLogo, accessed on 17/09/18). *C*, Example MS/MS spectrum of an N-terminal peptide modified with Biotin-ALPET.

To characterize the SrtA-labeling reaction in whole-cell lysates and to determine any bias toward labeling of protein substrates, we performed enrichment of SrtA-labeled N-terminal tryptic peptides followed by LC-MS/MS analysis (Fig. 1). Cells were treated in triplicate with IMP-1088 for 24 h to induce nearly complete depletion of protein *N*-myristoylation. Samples were labeled with SrtA, precipitated to remove excess depsipeptide and digested with trypsin. SrtA-biotinylated peptides were enriched using NeutrAvidin agarose beads, eluted as reported previously (18), desalted and analyzed by nanoLC-MS/MS. The peptide sequences identified were matched to a modified version of the Swiss-Prot human proteome (UniProt; December 2017; 42,326 entries), in which the ALPET peptide sequence was added to the N-termini of all proteins in the database. Analysis of 120 identified modified peptides (supplemental Table S2, supplemental Figs. S2 and S3) revealed high selectivity of *S. aureus* SrtA for Gly in the first amino acid (61%) compared with the average frequency of N-terminal Gly across the human proteome (Fig. 3*A*, supplemental Table S2 and supplemental Fig. S2). Interestingly we observed direct evidence for low levels of labeling of N termini other than Gly (*e.g.* Ala, Met, Pro, Val), suggesting that this SrtA pentamutant has somewhat relaxed specificity at the N terminus under conditions of excess SrtA and depsipeptide substrate (supplemental Fig. S2). Analysis of amino acid positions 2–10 did not reveal any strong preference of SrtA for amino acids in any of these positions. Modified N-terminal peptides corresponding to 30 known NMT substrates were identified, which show a very similar profile to the complete list of human myristoylated proteins derived from UniProt and previous proteomics experiments using YnMyr (8) (Fig. 3*B*).

##### NeutrAvidin Dimethylation and Two-step LysC/Trypsin Digestion Eliminates Avidin Background in On-bead Digests

SrtA labels a wide range of proteins in the lysate containing an accessible N-terminal Gly, only ca. 25% of which derive from NMT substrates, and to a much lesser extent proteins with an N-terminal Ala or other amino acids. Protein-level quantification of NMT activity against this high level of endogenous N-terminal Gly labeling proved challenging; for example, in a preliminary experiment following a previously reported protein pull-down and on-bead tryptic digest protocol (8) for SrtA-labeled samples, only 7 NMT substrates could be identified (data not shown), urging us to seek strategies to reduce background signals. Noting that a prominent source of contamination during biotin/avidin enrichment comes from digestion of avidin itself, we derivatized commercial NeutrAvidin resin to make it proteolytically resistant to LysC by on-bead dimethylation of NeutrAvidin Lys residues prior to enrichment, combined with two-step on-bead/off-bead digestion with LysC and trypsin, respectively. Derivatized NeutrAvidin retained its biotin-binding capacity but resists LysC cleavage; by eluting resin bound-polypeptides first with LysC and then completing digestion off-bead with trypsin we were able to eliminate Neutravidin-derived peptide contamination (supplemental Fig. S4). The resultant uncontaminated MS^1^ spectra boosted identification rates significantly, suggesting this simple method may provide a generally applicable enhancement for pull-downs on avidin resins.

##### Evaluation of SrtA Labeling as a Method for Studying NMT Activity by Protein-based Enrichment Proteomics

**Fig. 4.**
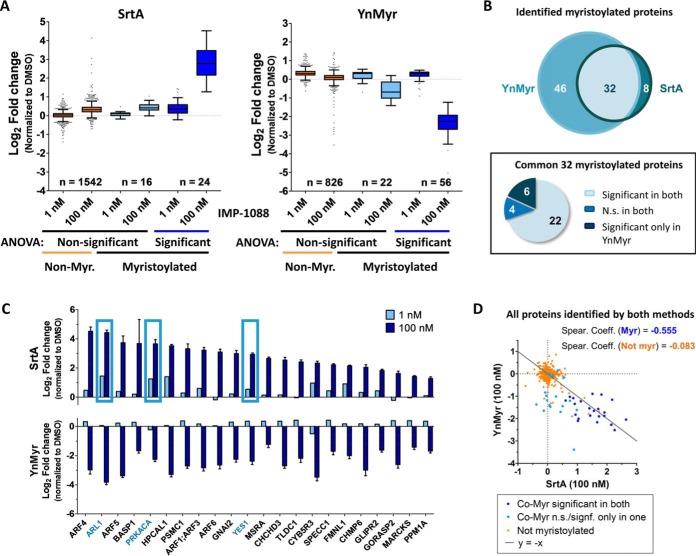
**SrtA-labeling complements YnMyr metabolic tagging for quantitative profiling of NMT activity by protein-enrichment proteomics analysis.** MDA-MB-231 cells were incubated in the presence of control (DMSO), low (1 nm) or high (100 nm) concentrations of NMT inhibitor IMP-1088 for 24 h and either biotinylated with SrtA post-lysis or fed with YnMyr for the duration of the NMT inhibitor treatment and biotinylated via CuAAC. Biotin-labeled proteins were enriched on NeutrAvidin beads and analyzed by nanoLC-MS/MS. *A*, Boxplot representation of fold changes for known myristoylated proteins *versus* all other identified proteins for both methods, derived from a one-way ANOVA test performed on all identified proteins (FDR = 0.01 and S_0_ = 1). *B*, Pie chart and Venn diagram comparing number of identified known myristoylated proteins and their response to NMT inhibition by each method. N.S., nonsignificant (ANOVA, FDR = 0.01 and S_0_ = 1). *C*, Bar plots showing side-by-side comparison of myristoylated proteins identified and significantly changing in both methods. Boxes highlight proteins selected for follow-up studies. *D*, Correlation of SrtA and YnMyr labeling enrichments in 100 nm IMP-1088 treated samples.

Having shown that SrtA can be used to label proteins at endogenous levels in complex whole-cell lysate, we next applied labeling of free N-terminal glycines by SrtA to indirectly measure protein myristoylation levels and to discriminate between cells possessing different levels of NMT activity. Lysates from cells treated with vehicle, low, or high concentrations of NMT inhibitor IMP-1088 in triplicate for 24 h were subjected to overnight labeling by SrtA and Biotin-ALPET-Haa. For direct comparison with a well-established method for the study of NMT activity, we cultured another set of cells under the same conditions in the presence of ω-alkynyl myristate (YnMyr) followed by post-lysis chemical ligation to a biotinylated reagent (AzRB) as reported previously (9). After protein precipitation, affinity enrichment with dimethylated NeutrAvidin agarose beads and two step on-bead/off-bead digestion, samples were labeled with 9-plex TMT, combined, and analyzed by LC-MS/MS (workflow scheme in Fig. 1). In the SrtA-labeled set of samples, 40 known NMT substrates were identified, 24 of which were responsive to NMT inhibition (ANOVA with FDR = 0.01 and S_0_ = 1), compared with a total of 78 NMT substrates (56 responsive to NMT inhibition) in the YnMyr experiment (ANOVA with FDR = 0.01 and S_0_ = 1) (Fig. 4*A* and supplemental Table S3). Comparison between the two data sets revealed significant overlap with 32 NMT substrates common to both methods, 22 of which were significantly responsive to NMT inhibition; 4 substrates did not show any response to NMT inhibition by either method, 6 showed a strong response only by YnMyr labeling (Figs. 4*B* and 4*C*), and no substrates responded only by SrtA. Six proteins not previously reported to be myristoylated responded to NMT inhibition in both methods: BAG5, PSMC2, PSMC5, SPAG1, SPANXB1, and SPANXC (supplemental Table S3 and supplemental Fig. S5*A*). One of these, SPANXB1 was identified as a novel NMT substrate as it contains an N-terminal Gly and its N-terminal peptide was found to be modified with YnMyr-AzRB (MS/MS spectra in supplemental Fig. S5*B*). SPAG1 and SPANXC have been reported to be interactors of SPANXB1 (26), whereas PSMC2 and PSMC5 form part of the proteasome with myristoylated protein PSMC1 and are known to coprecipitate with this protein (8, 27). More detailed quantitative analysis of proteins revealed small differences in the levels of enrichment (Fig. 4*C*), but SrtA and YnMyr labeling patterns showed the expected complementary inverse correlation for known NMT substrates, such that SrtA-labeling increases with NMT inhibition and new exposure of N-terminal Gly, whereas YnMyr labeling is reduced, a trend which is absent in nonsubstrate proteins (Fig. 4*D*). Sequence analysis of the 10 first amino acids of all known myristoylated proteins identified in both protein-enrichment based proteomics experiments revealed a highly similar consensus sequence with minimal differences, which are most likely because of the more limited number of substrates identified with SrtA labeling (supplemental Fig. S5*C*).

##### In-gel Analysis and SrtA-ELISA as High-throughput NMT Target Engagement Biomarker Assays

**Fig. 5.**
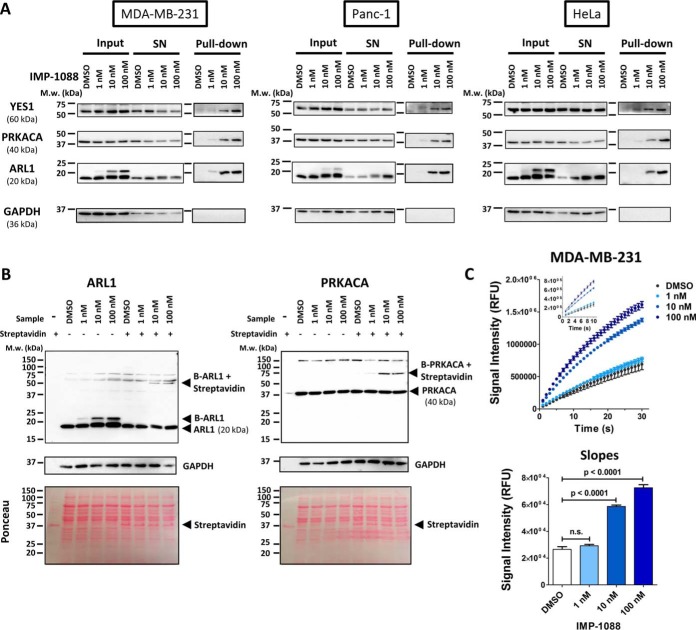
**Validation and high-throughput biomarker assays by Western blotting and ELISA.** *A*, Pull-down and Western blot analysis of SrtA-labeled proteins. MDA-MB-231, Panc-1 and HeLa cells were treated with DMSO or increasing concentration of IMP-1088 NMT inhibitor, lysed and labeled with SrtA overnight; biotinylated proteins were enriched on NeutrAvidin beads, resolved by SDS-PAGE and blotted against YES1 (60 kDa), PRKACA (40 kDa), ARL1 (20 kDa) and GAPDH as loading control (36 kDa). SN: supernatant. NMT substrate proteins show concentration-dependent increase in enrichment with increasing concentration of NMT inhibitor, whereas the low molecular weight of ARL1 also allows for direct identification of the SrtA-labeled fraction in the input sample because of the gel shift induced by ligation to Biotin-ALPET. *B*, Streptavidin shift analysis of SrtA-labeled proteins. SrtA-labeled samples were briefly incubated with streptavidin before SDS-PAGE and blotting against ARL1 and PRKACA. SrtA biotinylated ARL1 (B-ARL1) shows a molecular weight shift induced by the Biotin-ALPET label that gets shifted by 30 kDa on Streptavidin binding. Biotinylated PRKACA (B-PRKACA) shows no apparent shift in the absence of streptavidin, but a clear shift of 30 kDa upon addition of streptavidin. These shifts match the apparent molecular weight of streptavidin alone, as shown by Ponceau staining. *C*, SrtA-ELISA analysis of labeled proteins. After protein precipitation to remove excess depsipeptide, 1 μg labeled protein lysate was applied to each well of a streptavidin-coated 96-well plate and incubated for 3 h at RT. After primary (anti-ARL1) and secondary (anti-rabbit HRP) antibody incubations, turnover of QuantaBlu fluorogenic HRP substrate was monitored for 30 min. Slopes were calculated from the first 10 min (linear range) after fitting to a straight line (*Y* = Slope · *X* + *Y_intercept_*) by nonlinear regression (Insert). Data were analyzed by one-way ANOVA followed by Dunnett's multiple comparison test.

Based on the proteomics results, we chose three NMT substrates (Fig. 4*C*, highlighted) that showed a dose-dependent signal for validation in a gel-based target engagement biomarker assay: ARL1, PRKACA and YES1. These biomarkers were further selected accounting for their molecular weight such that they could be analyzed in a single gel together with a loading control (GAPDH). Cells were treated with vehicle control or increasing concentration of IMP-1088 and lysed in SrtA reaction buffer. Proteins in lysates were labeled with SrtA in the presence of Biotin-ALPET-Haa, enriched on standard NeutrAvidin beads, subjected to SDS-PAGE and blotted for ARL1, PRKACA and YES1 (Fig. 1). Increased NMT inhibitor concentration resulted in a higher signal intensity in the pull-down fractions, consistent with an NMT inhibitor-dependent increase in labeling by SrtA, and confirming the results obtained from protein-based enrichment proteomic analysis (Fig. 5*A*, supplemental Fig. S6*A*, S6*C*, and S6*D*). This result was robustly reproducible across both pancreatic (Panc1) and cervical (HeLa) cell lines. Because of the low molecular weight of ARL1 (∼20 KDa), labeling with Biotin-ALPET (∼800 Da) peptide by SrtA results in a detectable molecular weight shift, enabling this protein to be analyzed directly without prior enrichment, thus considerably increasing the throughput. To enable facile analysis of biomarkers of greater molecular weight we performed a streptavidin-shift assay (28). In this experiment, SrtA-labeled lysates were precipitated to remove excess depsipeptide and incubated with streptavidin before loading onto polyacrylamide gels. Streptavidin binds to the biotinylated proteins, shifting their apparent molecular weight by c.a. 30 KDa, corresponding to the molecular weight of the streptavidin dimer; ARL1 and PRKACA gels showed the expected mass shift (Fig. 5*B*), whereas YES1 was found to be unsuited to this assay because of the high background of the antibody (supplemental Fig. S6*B*).

Finally, to enable high-throughput biomarker analysis, we developed and optimized a SrtA-ELISA assay using streptavidin-coated 96-well plates to capture all SrtA-biotinylated proteins (*i.e.* N-terminal glycine-containing proteins), and detected ARL1 using an HRP conjugate antibody. This assay robustly and specifically quantified the dose-dependent increase in biotinylated ARL1 in IMP-1088 treated MDA-MB-231 cells at inhibitor concentrations as low as 10 nm, providing a straightforward assay to distinguish between NMT inhibition and noninhibited controls (Fig. 5*C*).

## DISCUSSION

Here we have demonstrated a new proteomic application for SrtA: labeling N-terminal Gly proteins across the proteome at endogenous levels. Our analysis of the substrate specificity of a SrtA pentamutant in whole-cell lysates verifies its promiscuity beyond the recognized preference for N-terminal glycine, and in contrast to previous reports in engineered systems (11, 29) did not suggest a particular preference for substrates bearing multiple glycine residues at the N terminus, or indeed any other specific amino acid preference in positions 2–10. These results demonstrate the versatility of SrtA for nonselective labeling of complex mixtures of proteins bearing an N-terminal glycine.

We successfully combined SrtA labeling of whole-cell lysates with a potent and selective NMT inhibitor, delivering a method capable of quantifying changes in cellular NMT activity at multiple substrates at the whole proteome level. This new SrtA-based labeling method shows good overlap in substrates with a previously reported YnMyr metabolic tagging strategy (8), and a similar capability to measure cellular NMT activity. Although YnMyr tagging has superior identification power for NMT substrates thanks to selective enrichment of myristoylated proteins, our SrtA approach successfully detects the response to NMT inhibition for most (81%) of the NMT substrates identified by both methods. We hypothesize that where SrtA does not detect a response to inhibition this is most likely because of the requirement for an exposed N-terminal glycine which can be affected by changes in structural conformation, or N-terminal modifications including proteolysis or N-terminal acetylation (30, 31), although the latter is relatively rare at N-terminal Gly in human cells (32). NMT substrate destabilization in the absence of *N*-myristoylation, as described previously (33–35), may also counterbalance enrichment. SrtA labelling did not result in false positives: all proteins responding to NMT inhibition were either NMT substrates, or in a few cases, known interactors of these substrates. The SrtA-based approach described here is complementary to YnMyr metabolic tagging, because the increase in signal for newly synthesized proteins exposing an N-terminal glycine mirrors the loss of signal seen with YnMyr. More importantly, this new method overcomes the limitations imposed by metabolic tagging as it can be applied in samples which are not metabolically active, as shown here for cell lysates. In future, we anticipate this will provide a useful approach for exploring changes in NMT activity in a wide range of samples, including organoids or tissue biopsies. This robust SrtA-based NMT activity assay can be coupled to a variety of detection techniques, from MS-based proteomics for in-depth quantitative analysis and *de novo* discovery, through simple gel-based analyses, to a novel SrtA-ELISA format for increased throughput. Analysis of ARL1 is particularly appealing as provides a quick visual readout of NMT activity status thanks to the gel shift seen in SrtA labeling when NMT activity is inhibited, without the need for affinity enrichment or any additional steps after SrtA-labeling.

To enhance sensitivity of detection in samples where the analyte of interest (NMT substrates) is diluted by SrtA labeling of a large number of other proteins with exposed N-terminal glycines (and to certain extent other N-terminal residues), we have developed an improved affinity enrichment procedure via on-bead lysine dimethylation of NeutrAvidin and selective elution of enriched proteins by a short LysC on-bead cleavage step followed by full digestion with trypsin off-bead. This procedure almost completely removes avidin-derived high intensity peaks without affecting the yield of enriched protein-derived peptides, thus increasing successful identification and overall performance. Notably, this straightforward protocol should be widely applicable to any biotin-avidin affinity enrichment procedure to provide improved results independently of the sample source.

In conclusion, the present work constitutes the first report of applying SrtA not only as a labeling mechanism but as a sensor that detects the status of the cellular *N*-myristoylated proteome, thereby broadening the scope of SrtA as a chemical proteomic tool. Moreover, because SrtA labeling is performed post-lysis this labeling strategy provides a readout of the cell status in the absence of any external perturbation, giving clear advantages over other in-cell labeling strategies. We anticipate that this method will enable the exploration of new avenues for NMT as a therapeutic target by providing a method for *de novo* identification of substrates most responsive to NMT inhibition, and a multifunctional target engagement biomarker assay suited to both animal models and patient biopsies.

## DATA AVAILABILITY

The mass spectrometry proteomics data have been deposited into the ProteomeXchange Consortium through the PRIDE (36) partner repository with the data set identifier PXD010634 (URL: https://www.ebi.ac.uk/pride/archive/projects/PXD010634).

## Supplementary Material

supplemental Table S1

## Footnotes

^1^ The abbreviations used are:

NMT: *N*-myristoyltransferase
AzTB: Azido-TAMRA-Biotin, bi-functional click chemistry reagent
Biotin-ALPET-Haa/TAMRA-ALPET-Haa: reagents used in Sortase A catalysed ligation
CuAAC: copper-catalyzed alkyne-azide cycloaddition (click chemistry)
Haa: 2-hydroxyacetamide
IMP-1088: 1-(5-(3,4-difluoro-2-(2-(1,3,5-trimethyl-1*H*-pyrazol-4-yl)ethoxy)phenyl)-1-methyl-1*H*-indazol-3-yl)-*N*,*N*-dimethylmethanamine, *N*-myristoyltransferase inhibitor
SrtA: Sortase A
TMT: Tandem Mass Tag; isobaric MS^2^ quantification tag
YnMyr: tetradecynoic acid; alkynyl myristic acid analogue.
