# Supplementary material for: Whole Proteome Profiling of N-Myristoyltransferase Activity and Inhibition Using Sortase A
Source: Mol Cell Proteomics. 2018 Oct 19;18(1):115–26. doi: 10.1074/mcp.RA118.001043 (PMC6317481; doi:10.1074/mcp.RA118.001043)
Supplement: supplemental Table S1 [file RA118.001043_index.html]

Supplement to Whole proteome profiling of N-myristoyltransferase activity and inhibition using Sortase-A | Molecular & Cellular Proteomics

## Supplemental Data

- Supplemental Tables (to be published online) - Tables
- Reviewed Supplementary Figures - Reviewed Supplementary Figures
