# Supplementary material for: Whole Proteome Profiling of N-Myristoyltransferase Activity and Inhibition Using Sortase A
Source: Mol Cell Proteomics. 2018 Oct 19;18(1):115–26. doi: 10.1074/mcp.RA118.001043 (PMC6317481; doi:10.1074/mcp.RA118.001043)
Supplement: supplemental Table S1 [file 140172_1_supp_208105_pfjxts.pdf]

SUPPLEMENTARY FIGURES

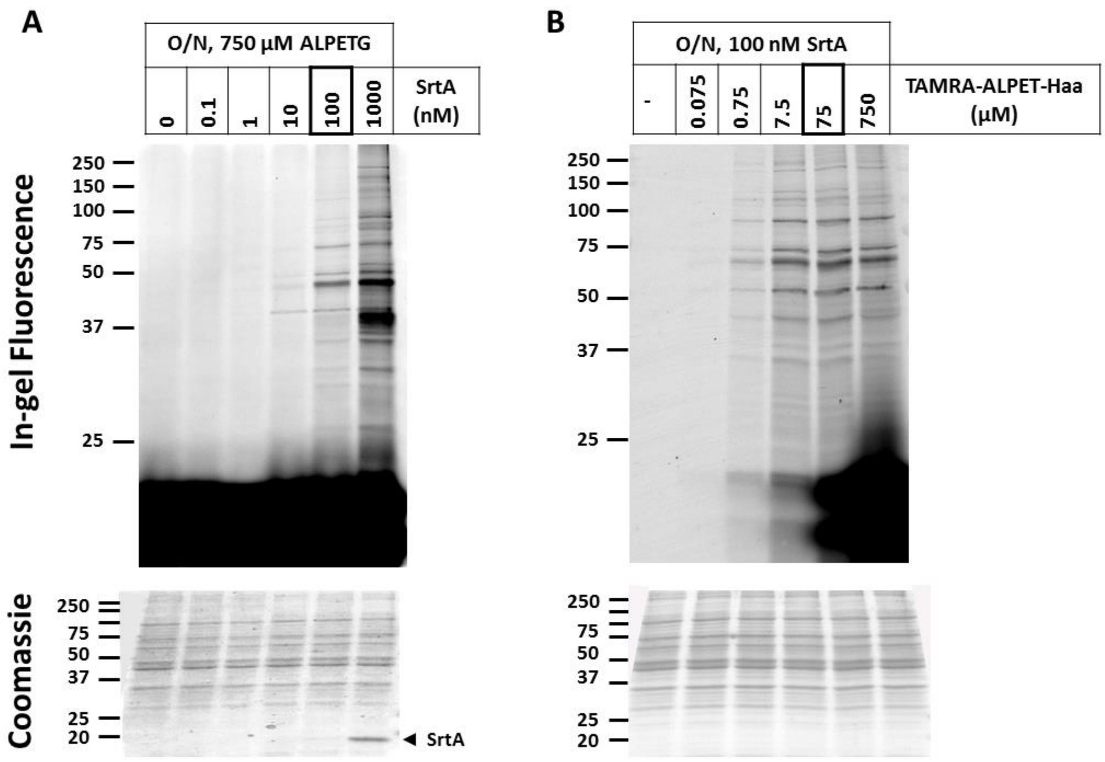

Figure S1. Optimization of overnight SrtA labeling reaction in whole-cell lysates. MDA-MB-231 cells were incubated with 100 nM IMP-1088 for 24 h before lysis. Lysates were labeled with SrtA and TAMRA-ALPET-Haa overnight at 4 °C and subjected to SDS-PAGE. In-gel fluorescence images were acquired with excitation/emission wavelengths of 532/568 nM. **(A)** Optimization of SrtA concentration with constant depsipeptide substrate. **(B)** Optimization of TAMRA-ALPET-Haa concentration with constant SrA concentration. Highlighted are the concentrations selected for further experiments.

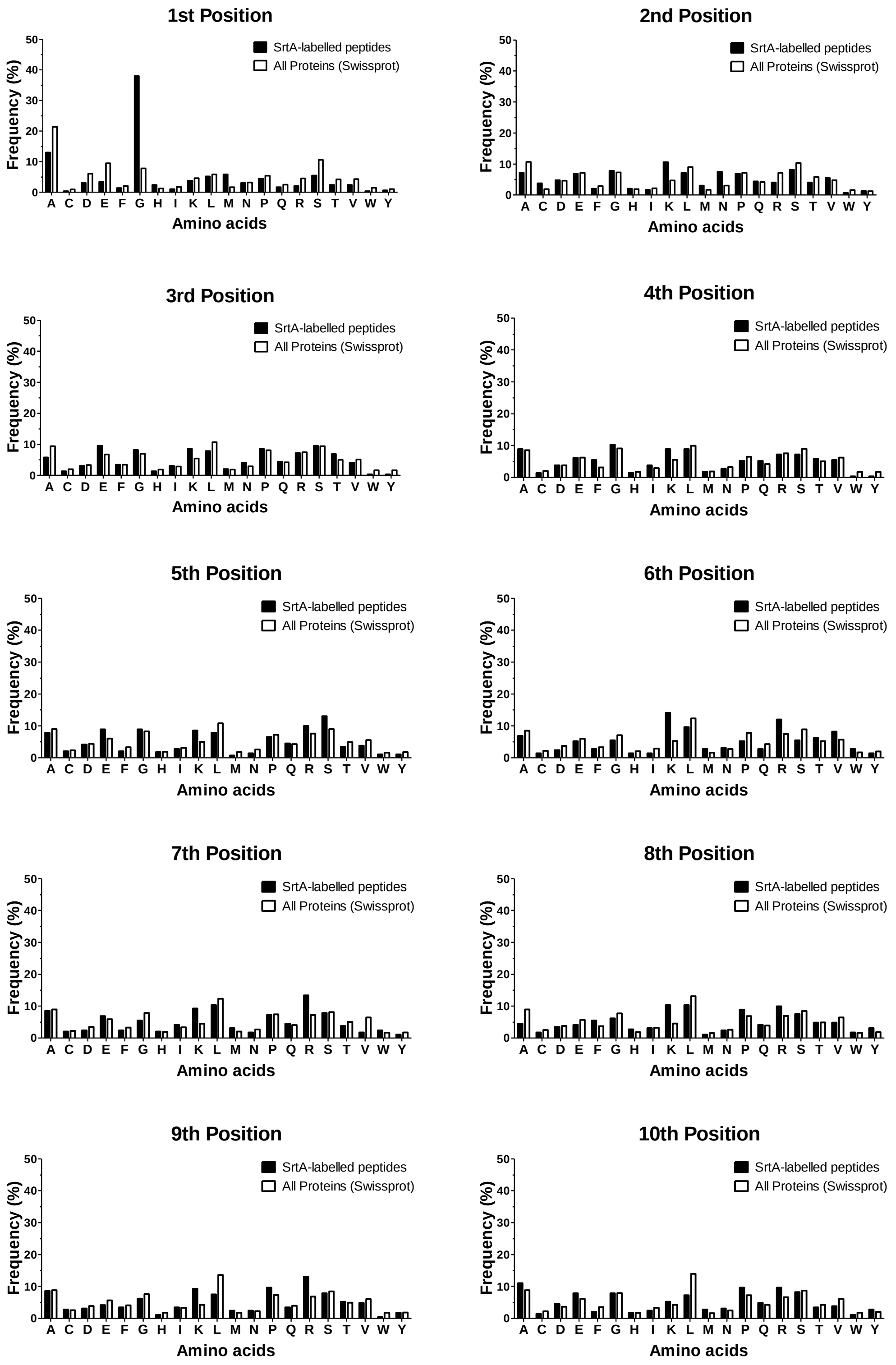

Figure S2. Bar plots comparing the frequencies for every amino acid per position in experimentally identified SrtA-labeled peptides and for all peptides of the human proteome (Swissprot, Uniprot, December 2017; 42,326 entries).

Raw File	Scan	Method	Score	m/z	Gene names
agg_pep_sort_pos_1	19070	FTMS; HCD	66.43	846.94	ACSS2

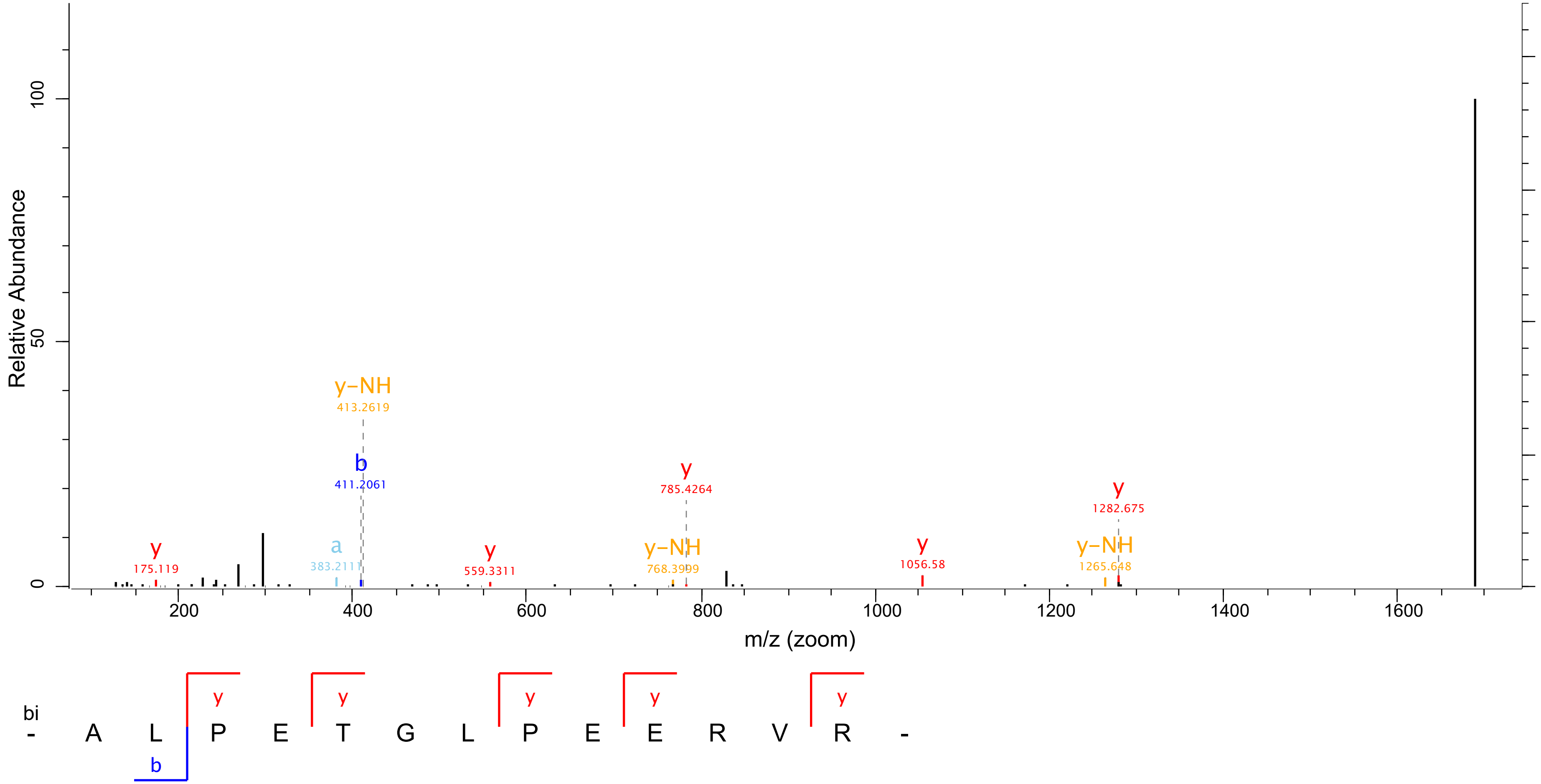

Raw File	Scan	Method	Score	m/z	Gene names
agg_pep_sort_pos_2	21799	FTMS; HCD	105.4	719.36	ACSS2

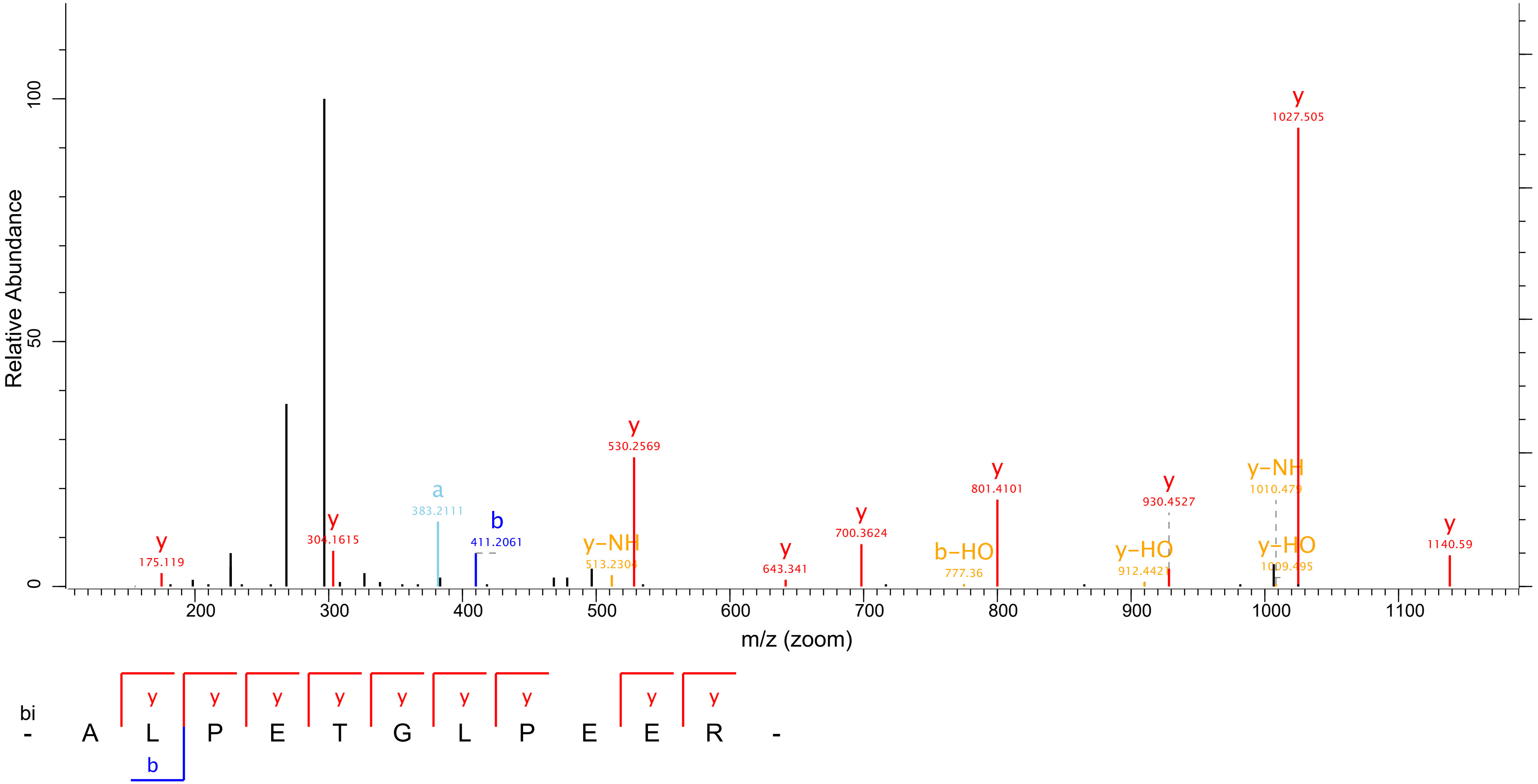

Raw File	Scan	Method	Score	m/z	Gene names
agg_pep_sort_pos_3	11600	FTMS; HCD	102.51	583.29	AGL

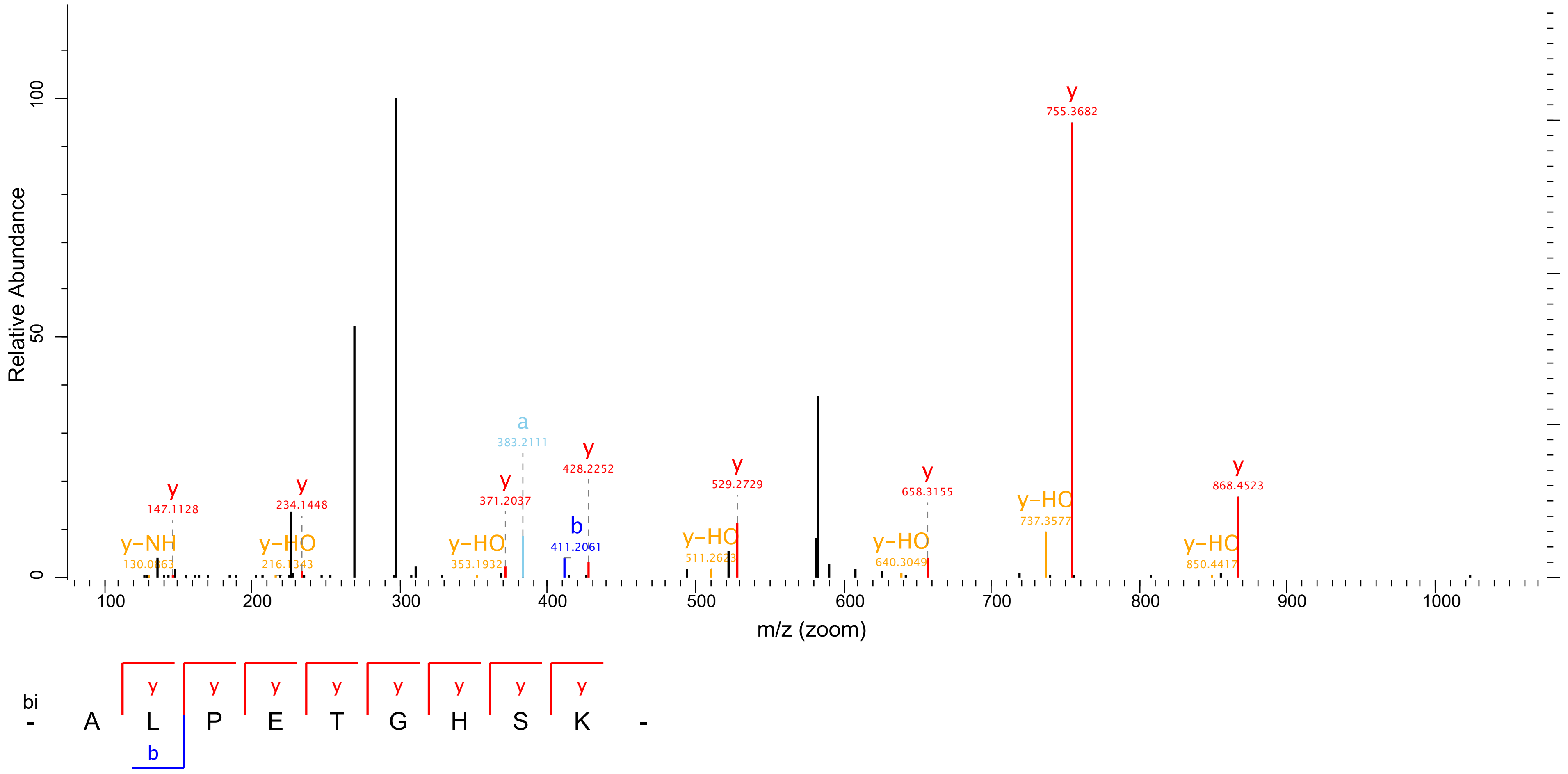

Raw File	Scan	Method	Score	m/z	Gene names
agg_pep_sort_pos_2	16988	FTMS; HCD	109.24	815.88	AKAP12

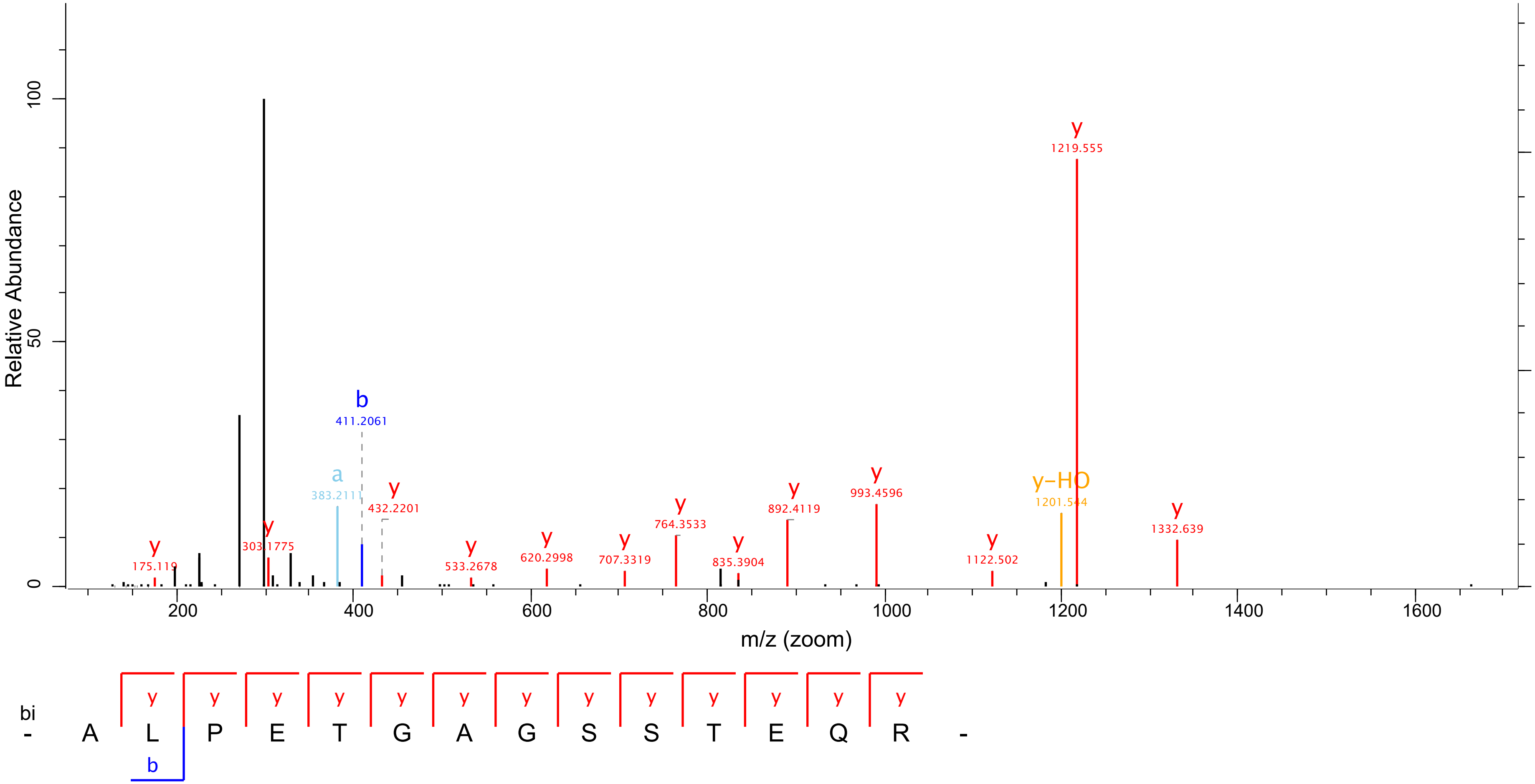

Raw File	Scan	Method	Score	m/z	Gene names
agg_pep_sort_pos_1	20649	FTMS; HCD	52.52	661.34	ANKS1A

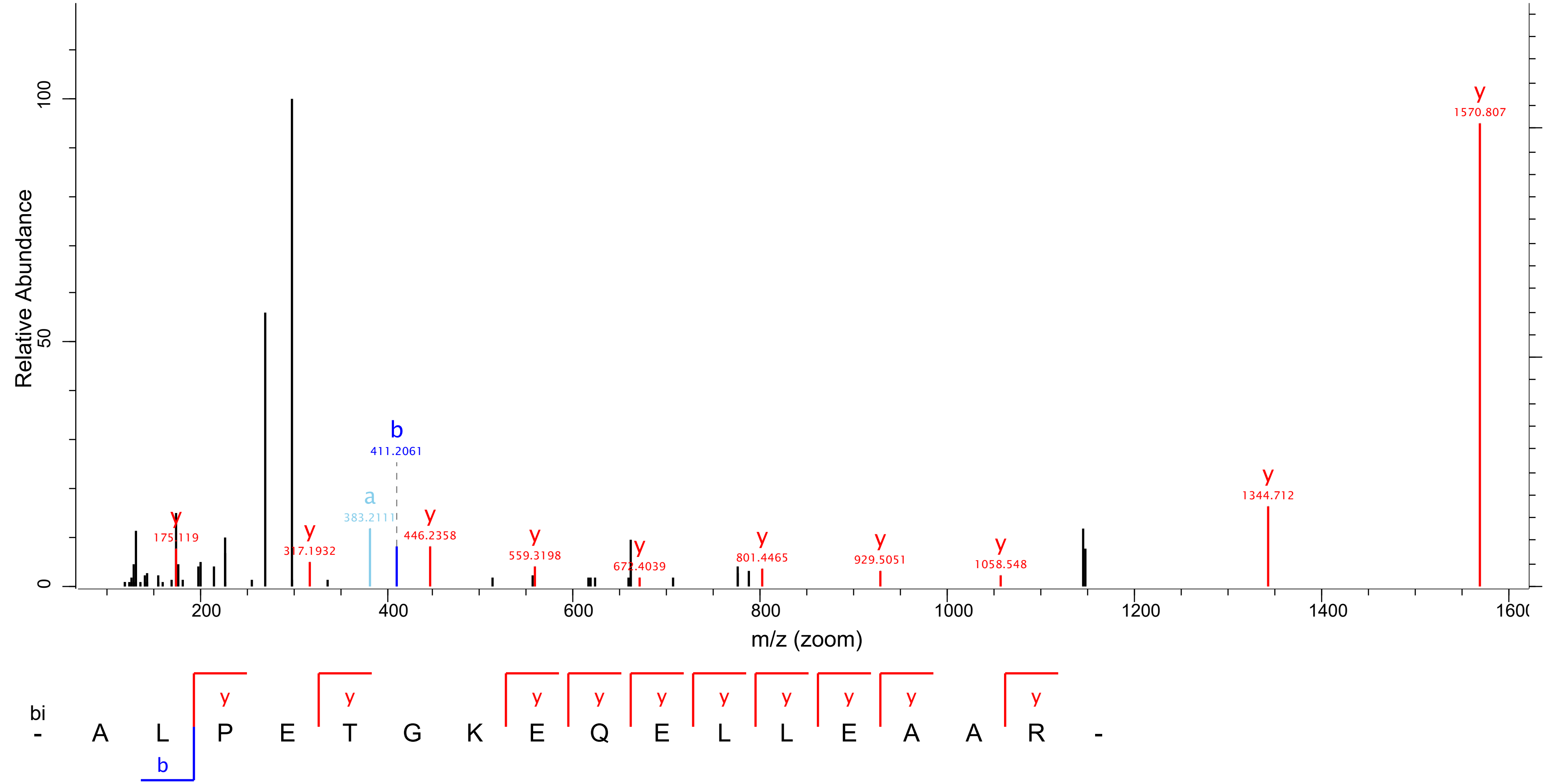

Raw File	Scan	Method	Score	m/z	Gene names
agg_pep_sort_pos_1	22174	FTMS; HCD	56.36	864.42	APLF

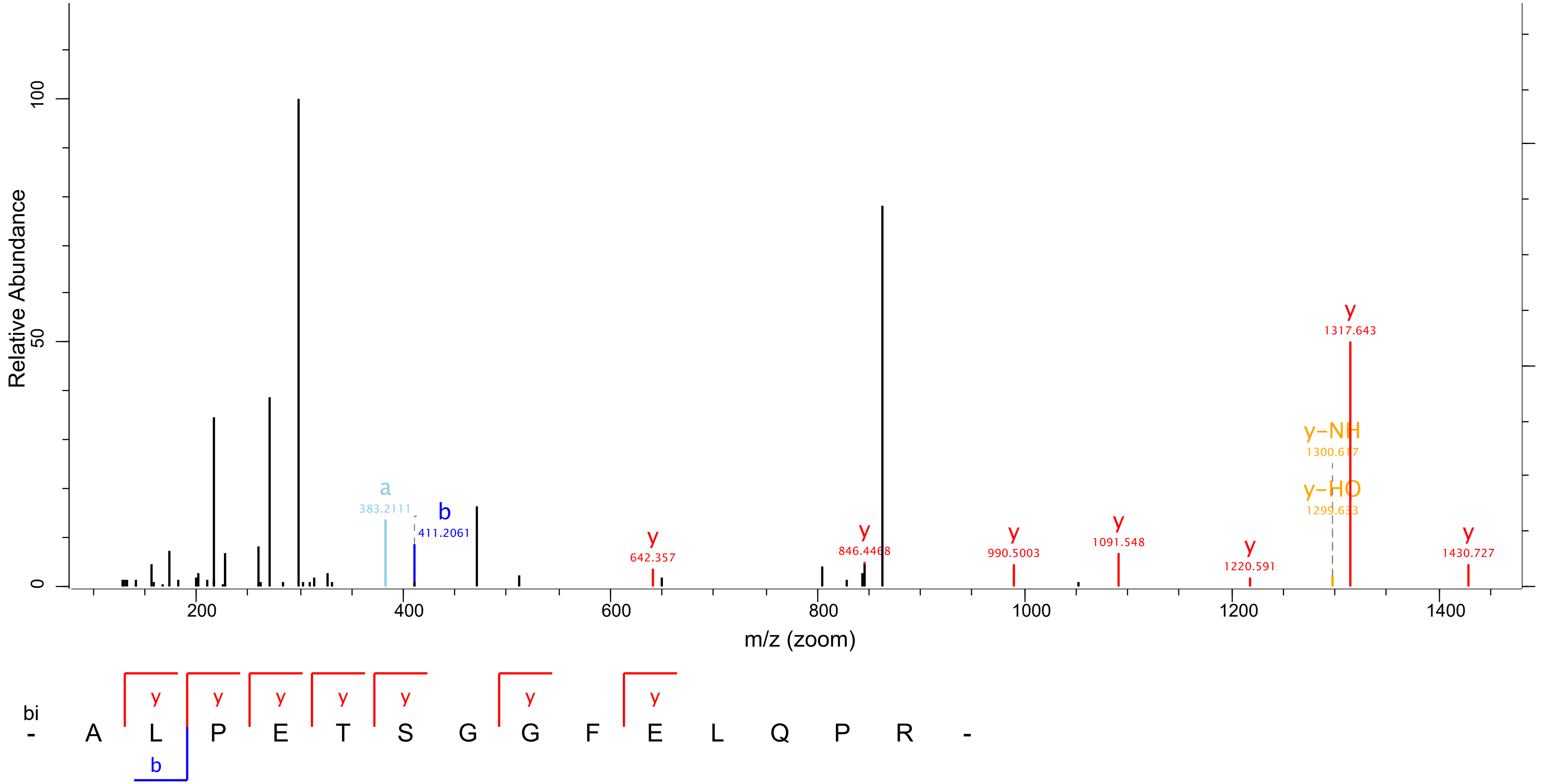

Raw File	Scan	Method	Score	m/z	Gene names
agg_pep_sort_pos_1	31832	FTMS; HCD	168.69	880.96	ARF1

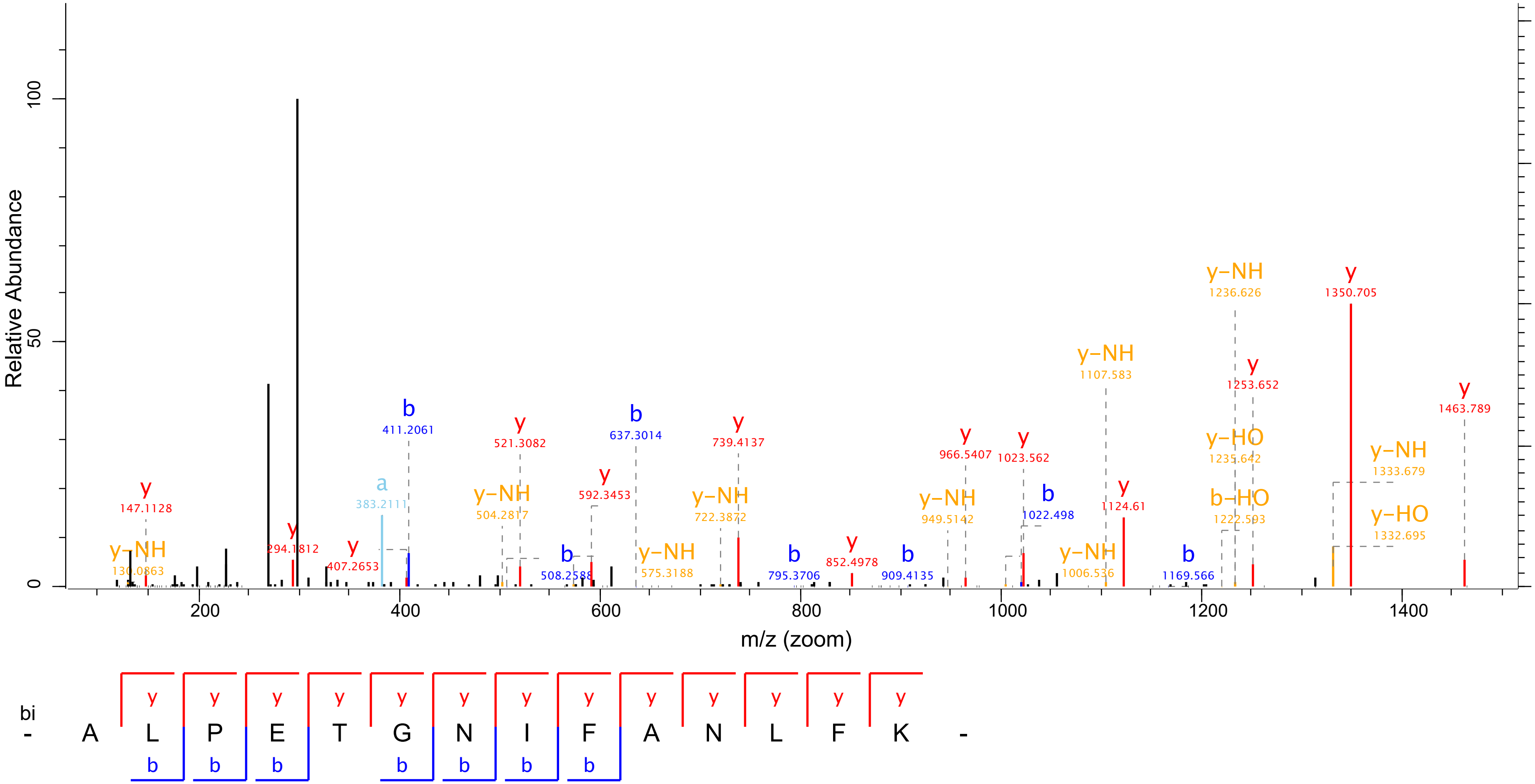

Raw File	Scan	Method	Score	m/z	Gene names
agg_pep_sort_pos_1	31640	FTMS; HCD	190.86	857.46	ARF3

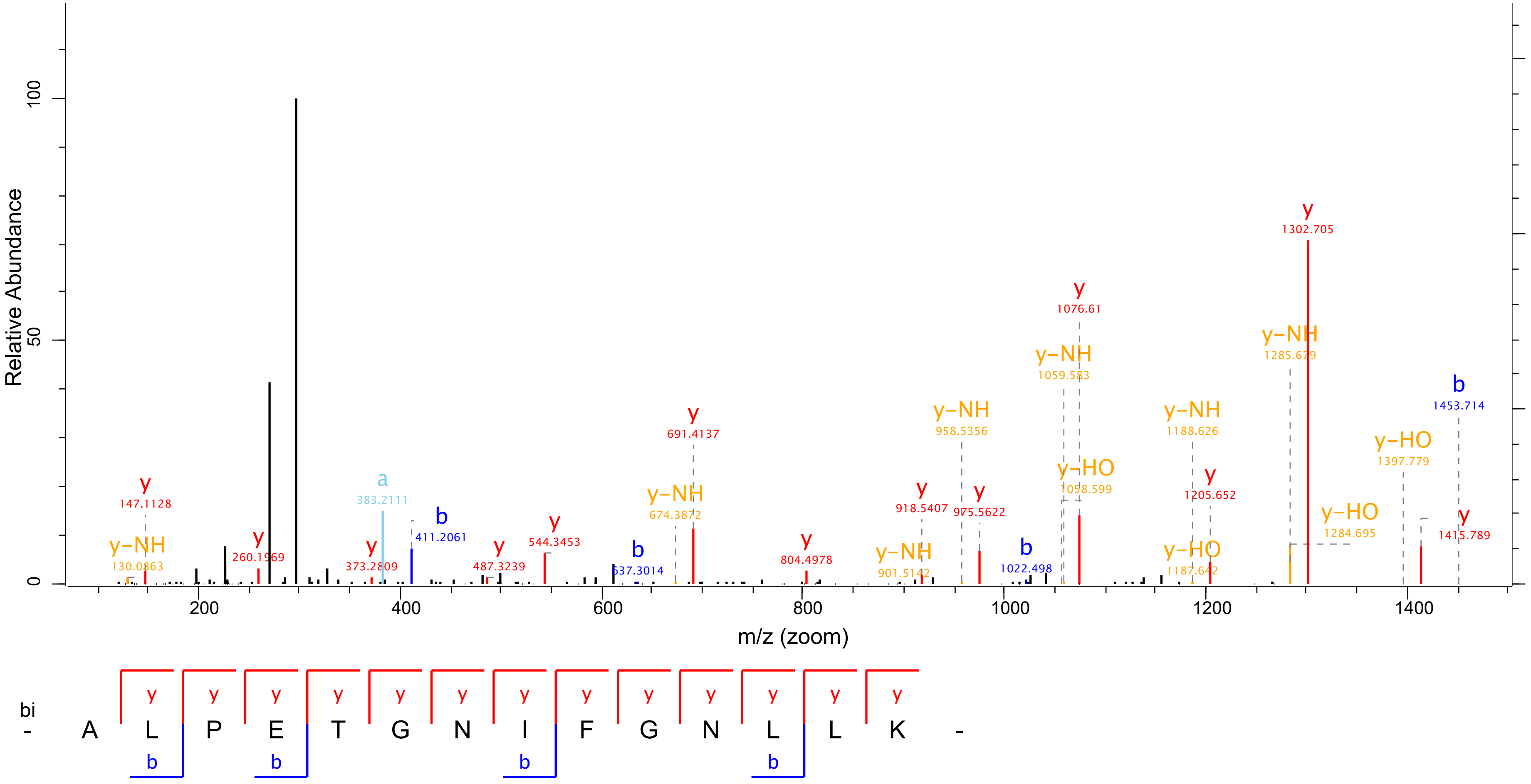

Raw File	Scan	Method	Score	m/z	Gene names
agg_pep_sort_pos_1	31721	FTMS; HCD	250.26	909.48	ARF4

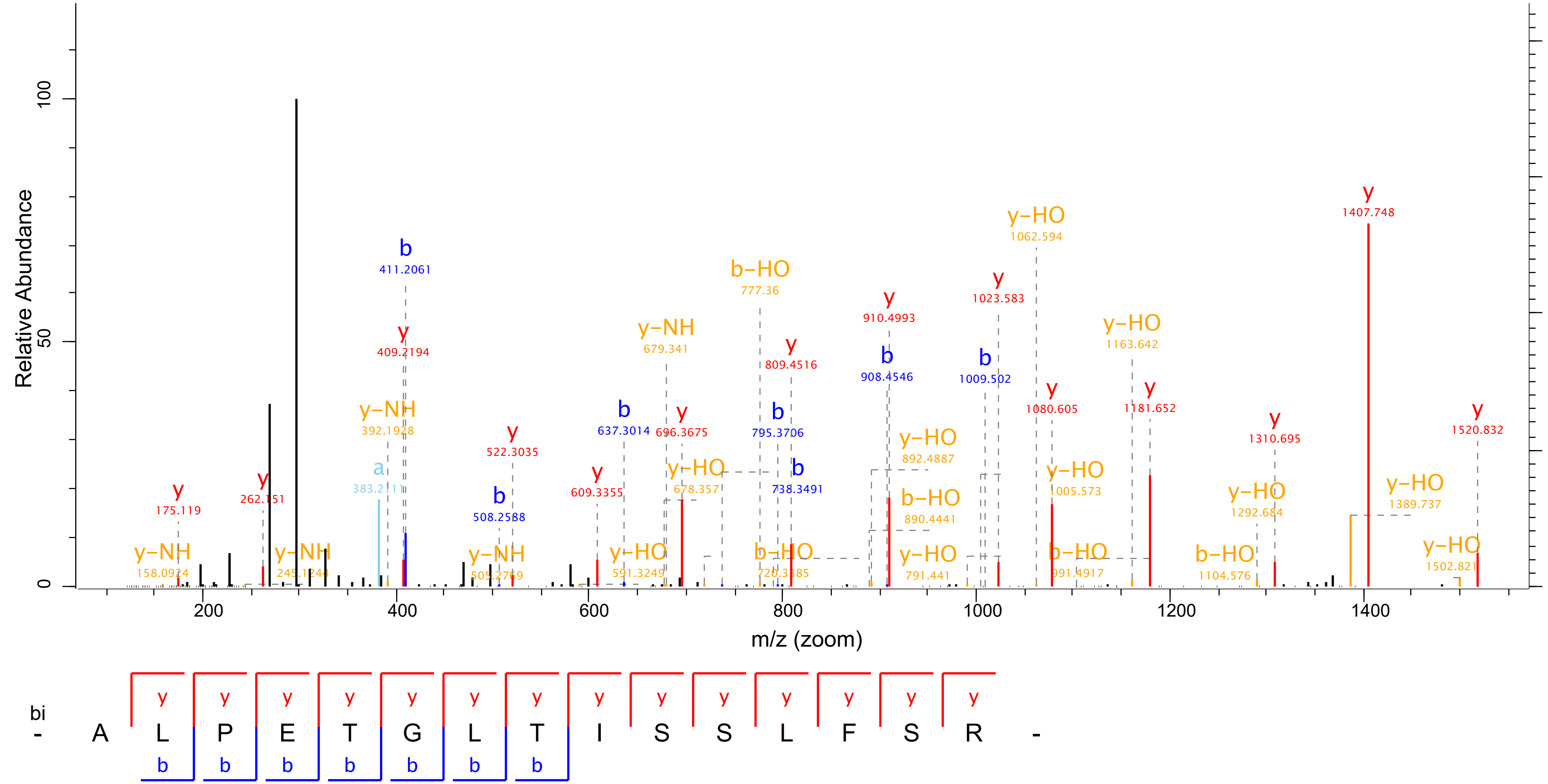

Raw File	Scan	Method	Score	m/z	Gene names
agg_pep_sort_pos_3	31282	FTMS; HCD	207.88	894.47	ARF5

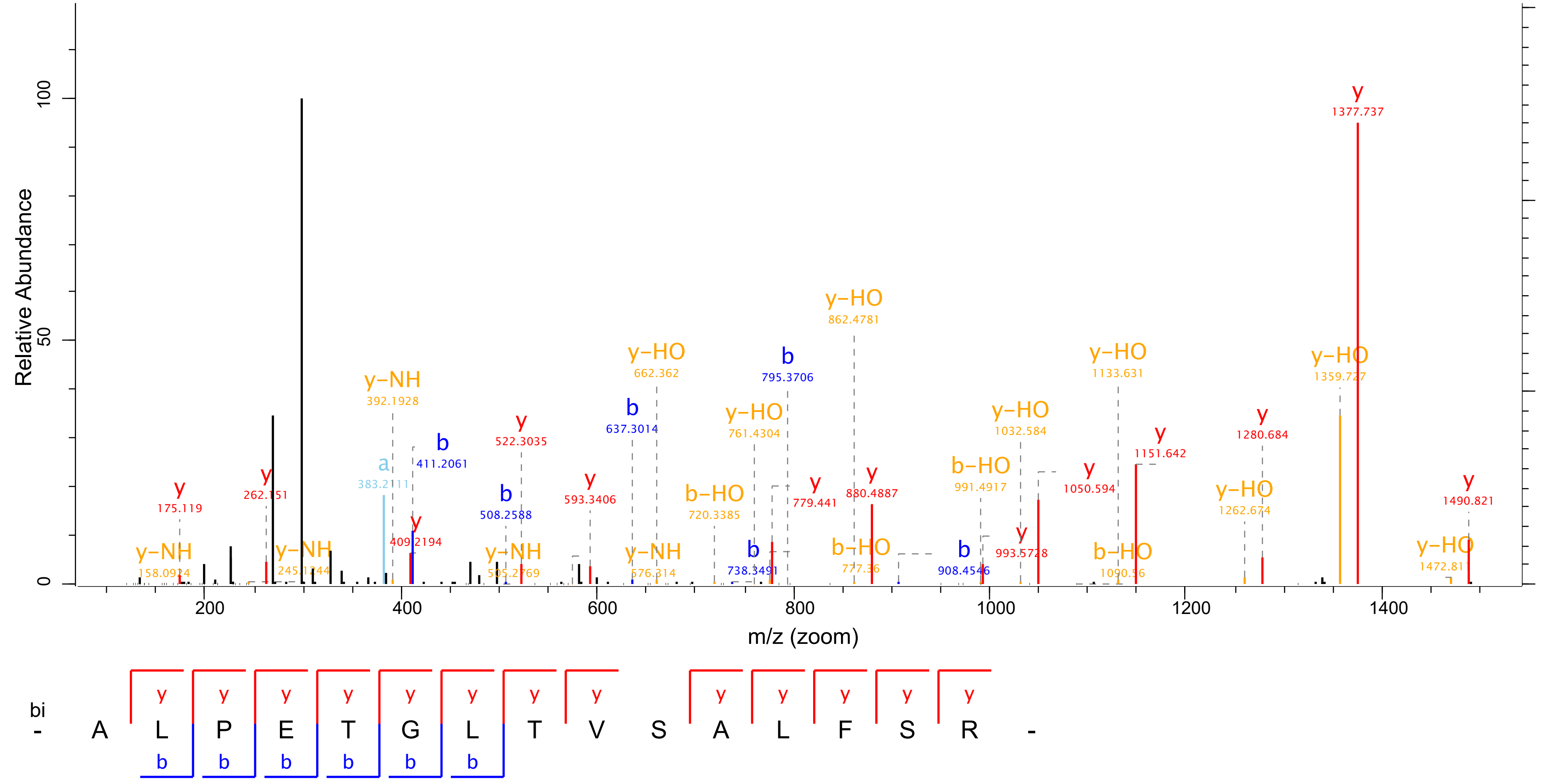

Raw File	Scan	Method	Score	m/z	Gene names
agg_pep_sort_pos_3	7498	FTMS; HCD	27.9	562.08	ARGFX

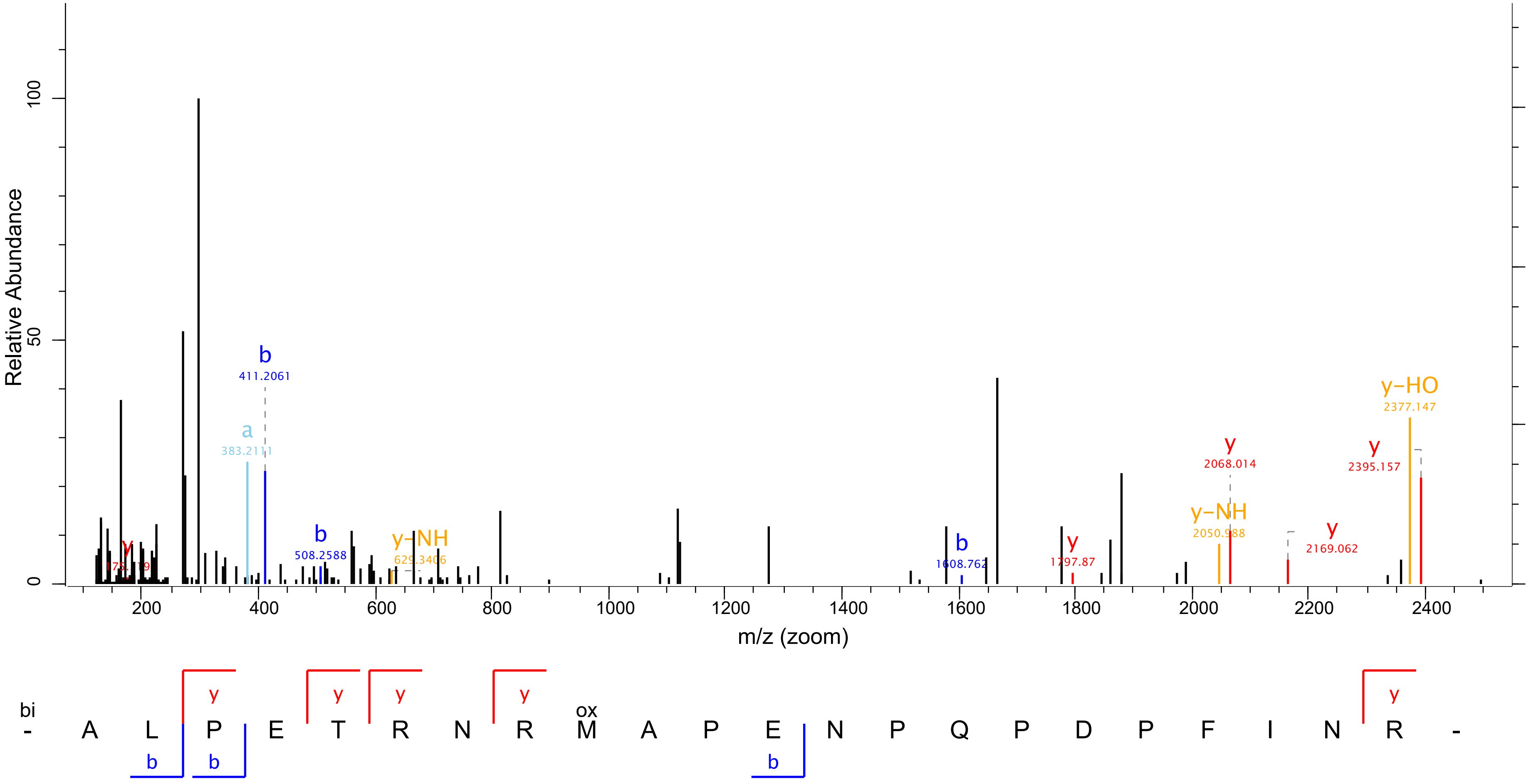

Raw File	Scan	Method	Score	m/z
agg_pep_sort_pos_2	17275	FTMS; HCD	98.52	597.8

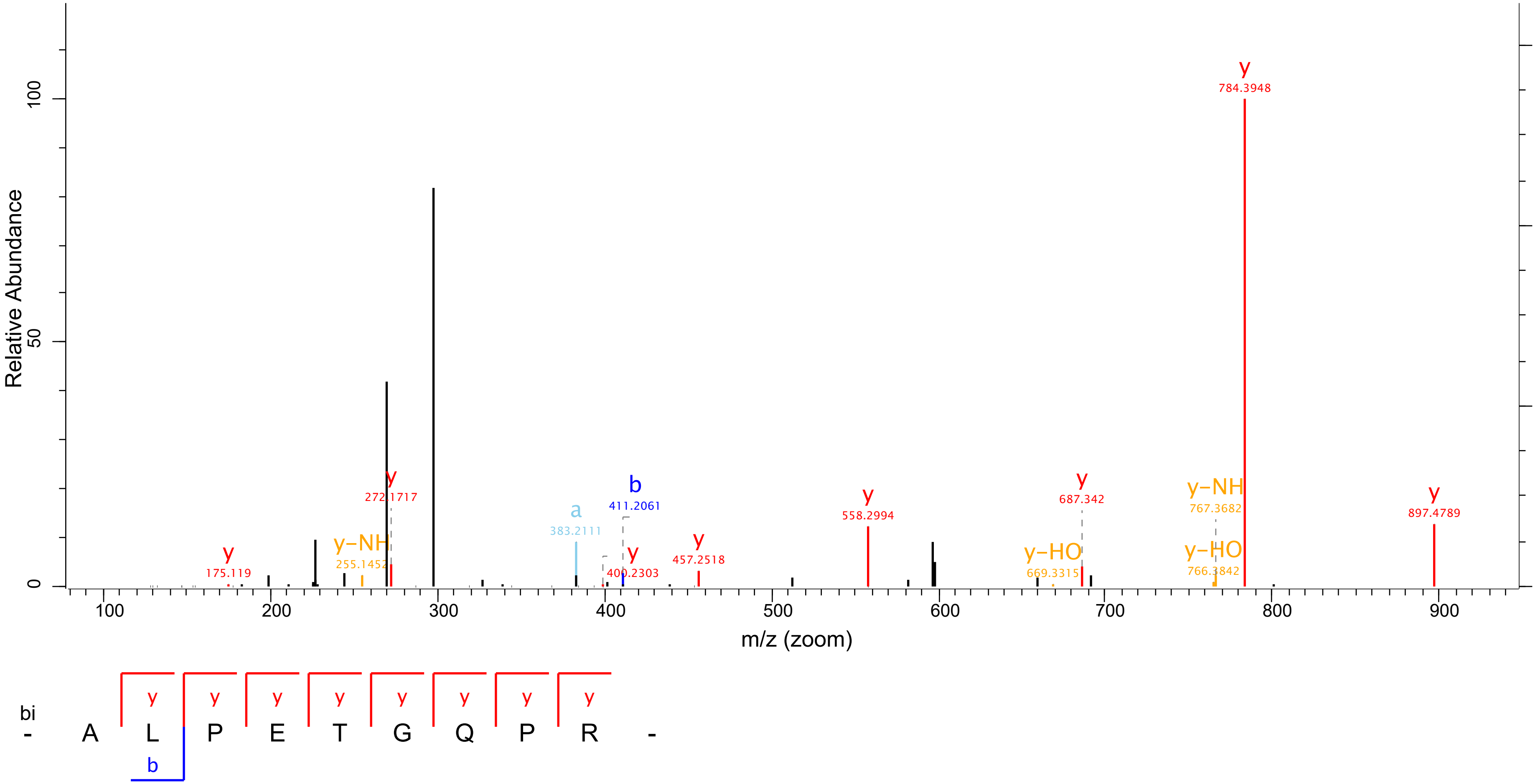

Raw File	Scan	Method	Score	m/z	Gene names
agg_pep_sort_pos_1	26814	FTMS; HCD	107.06	811.98	ARL2

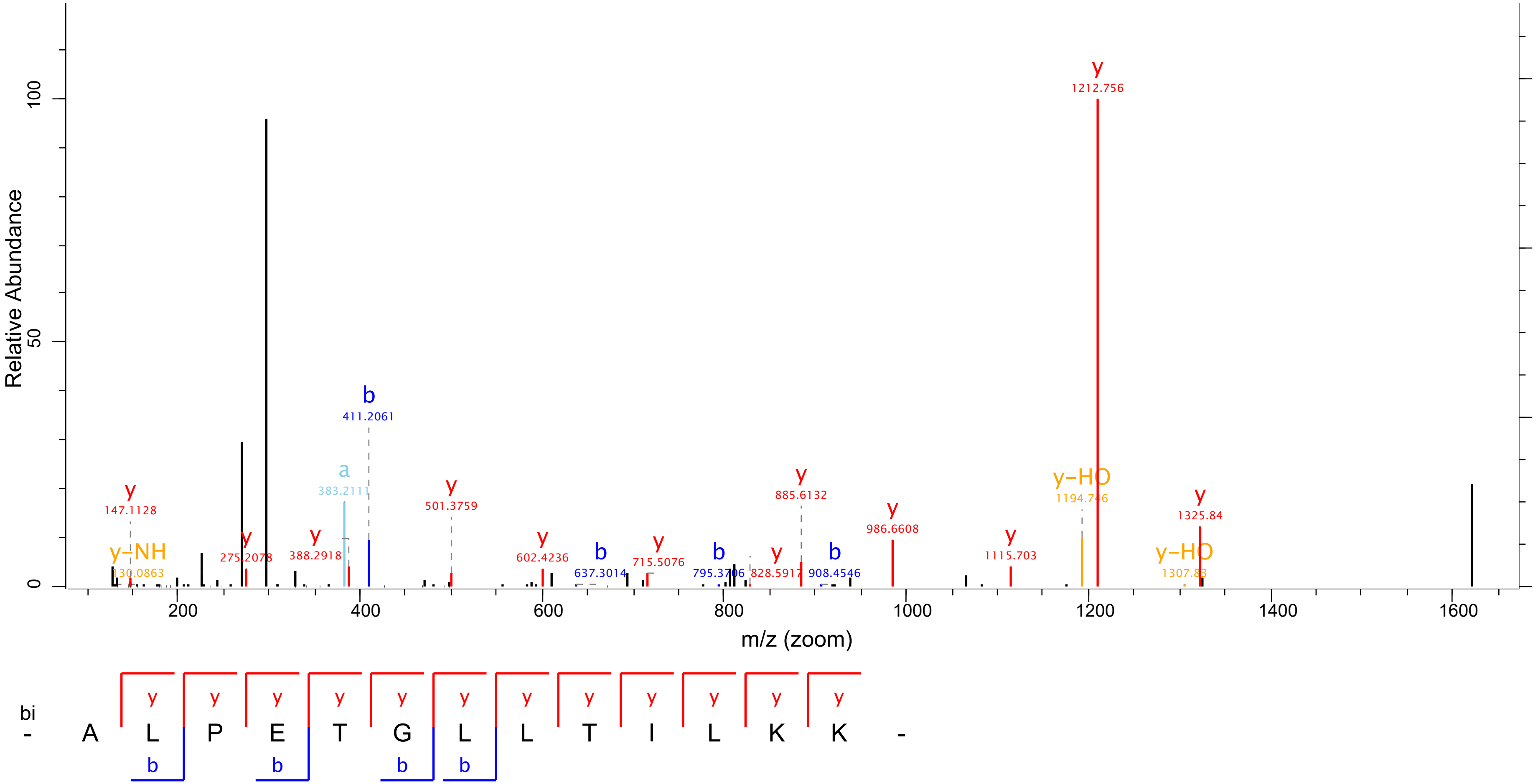

Raw File	Scan	Method	Score	m/z	Gene names
agg_pep_sort_pos_3	31323	FTMS; HCD	98.05	747.94	ARL2

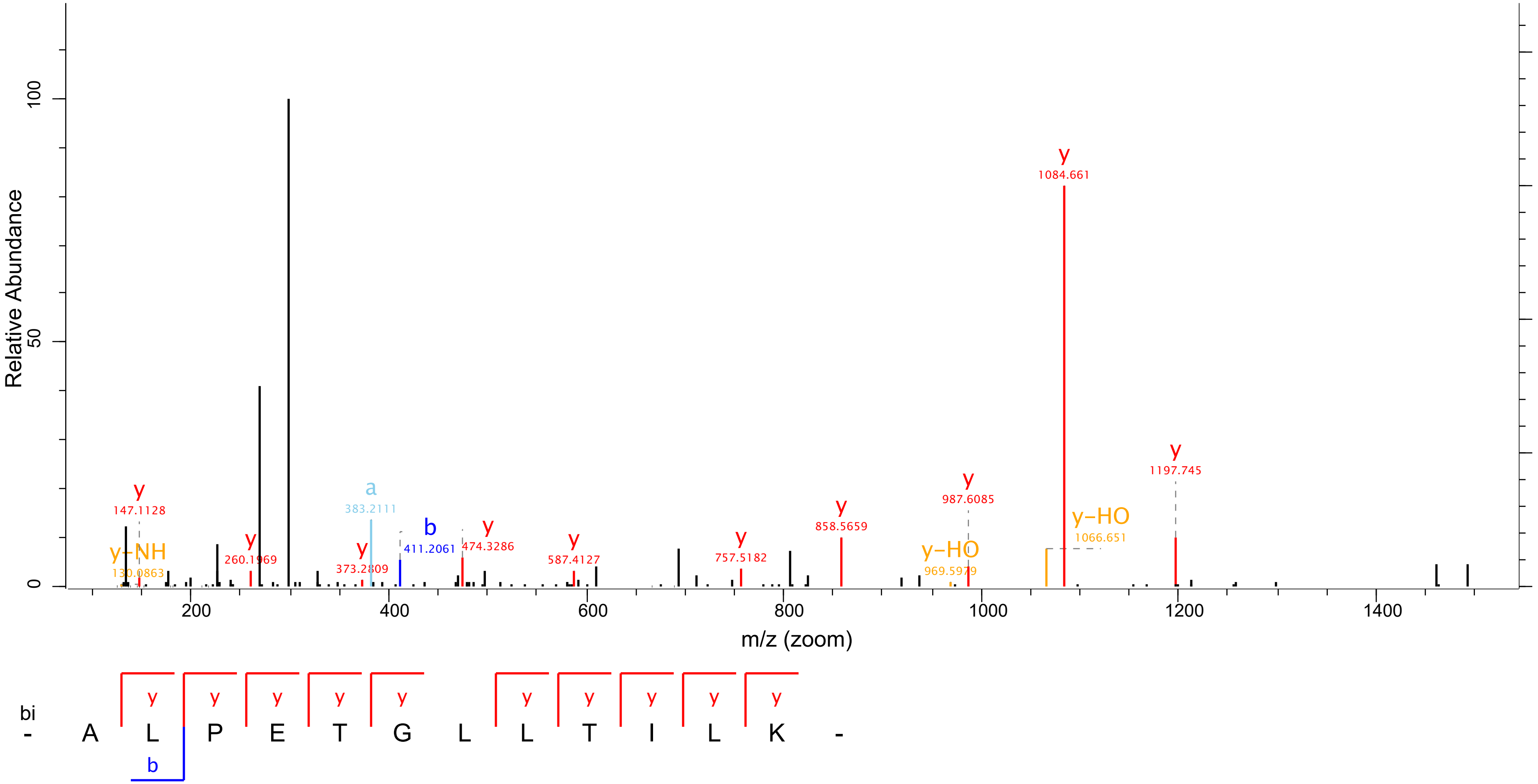

Raw File	Scan	Method	Score	m/z
agg_pep_sort_pos_1	18578	FTMS; HCD	68.07	1085.04

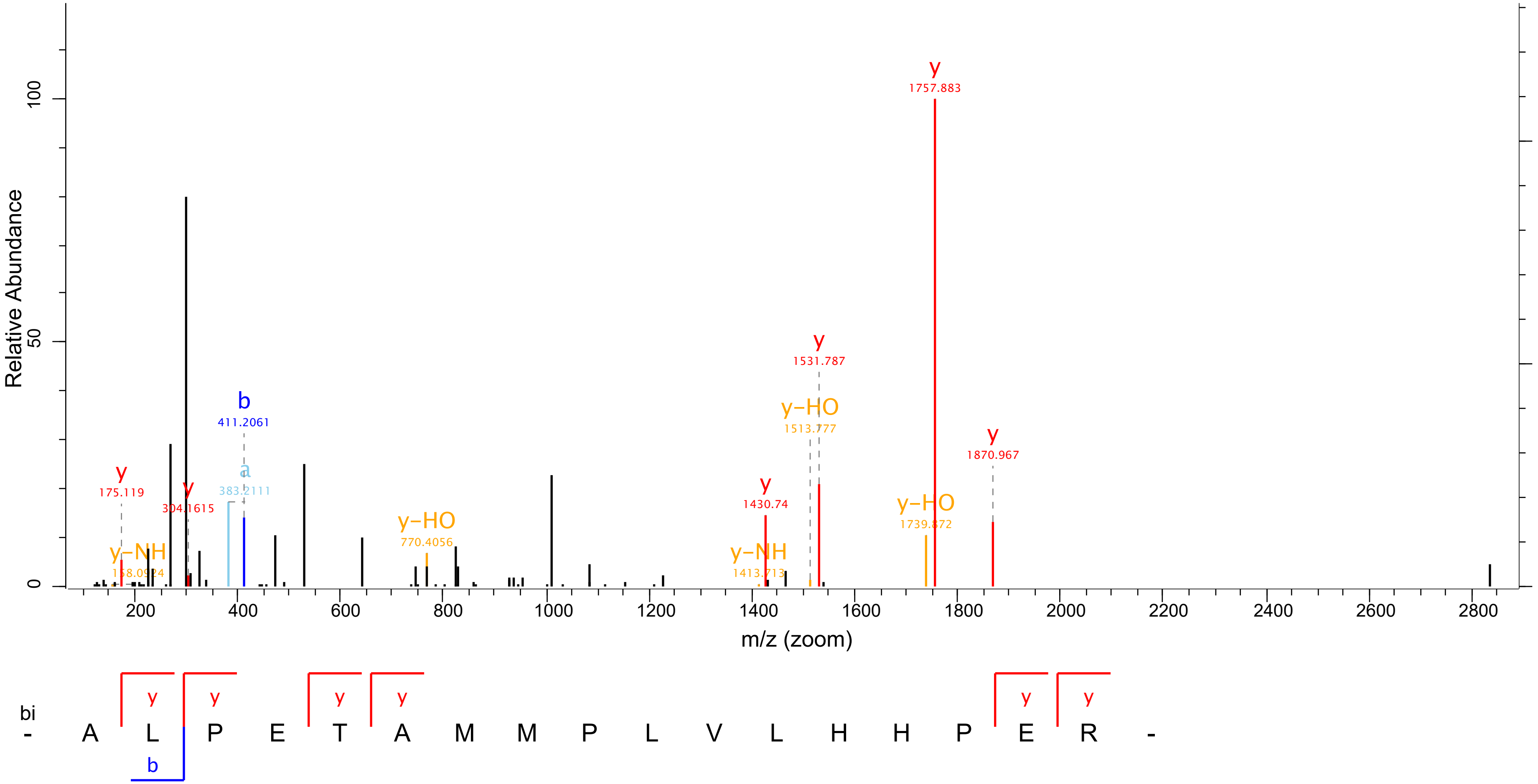

Raw File	Scan	Method	Score	m/z
agg_pep_sort_pos_1	14207	FTMS; HCD	67.99	656.34

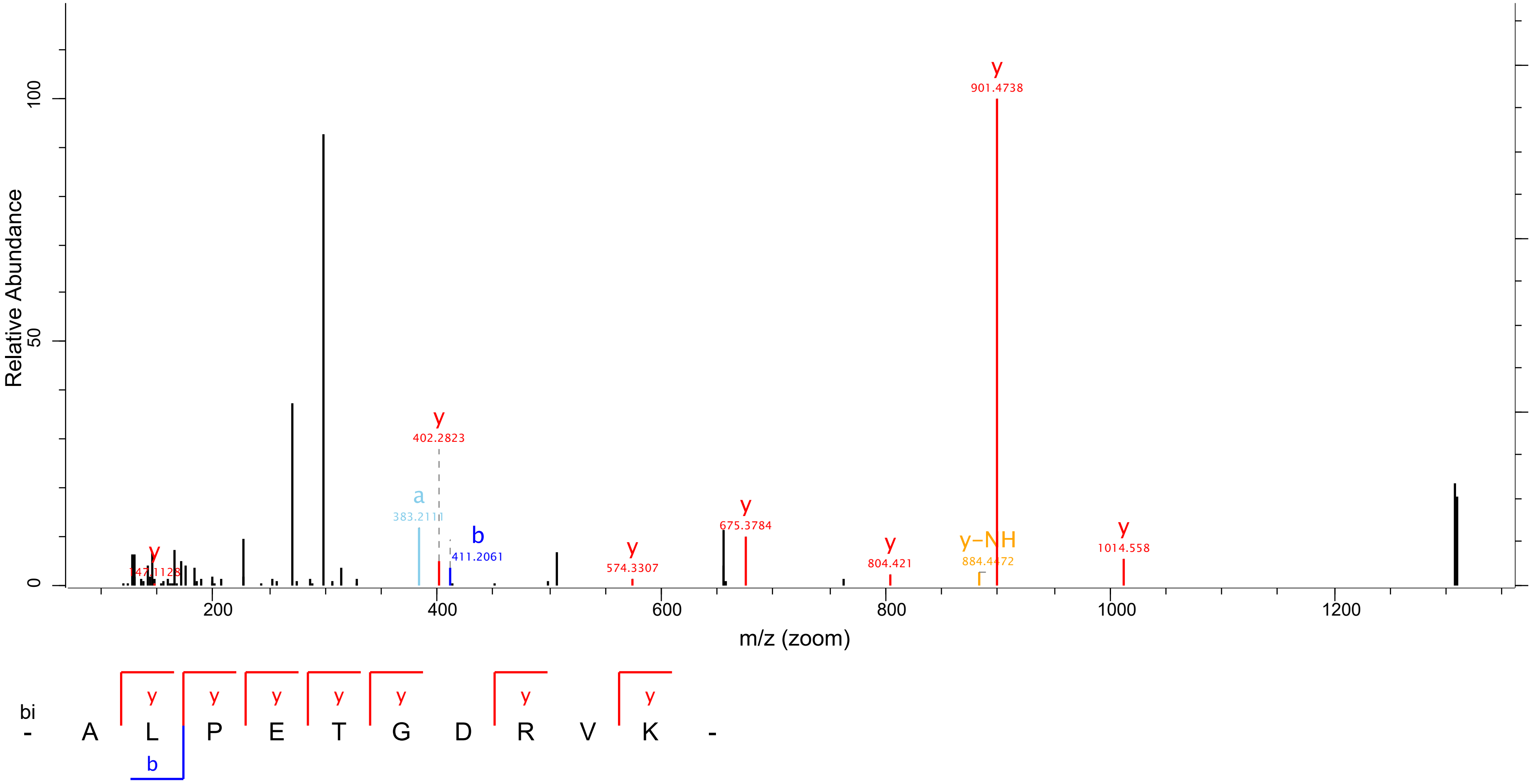

Raw File	Scan	Method	Score	m/z	Gene names
agg_pep_sort_pos_1	9512	FTMS; HCD	57.84	495.6	ATP1B3

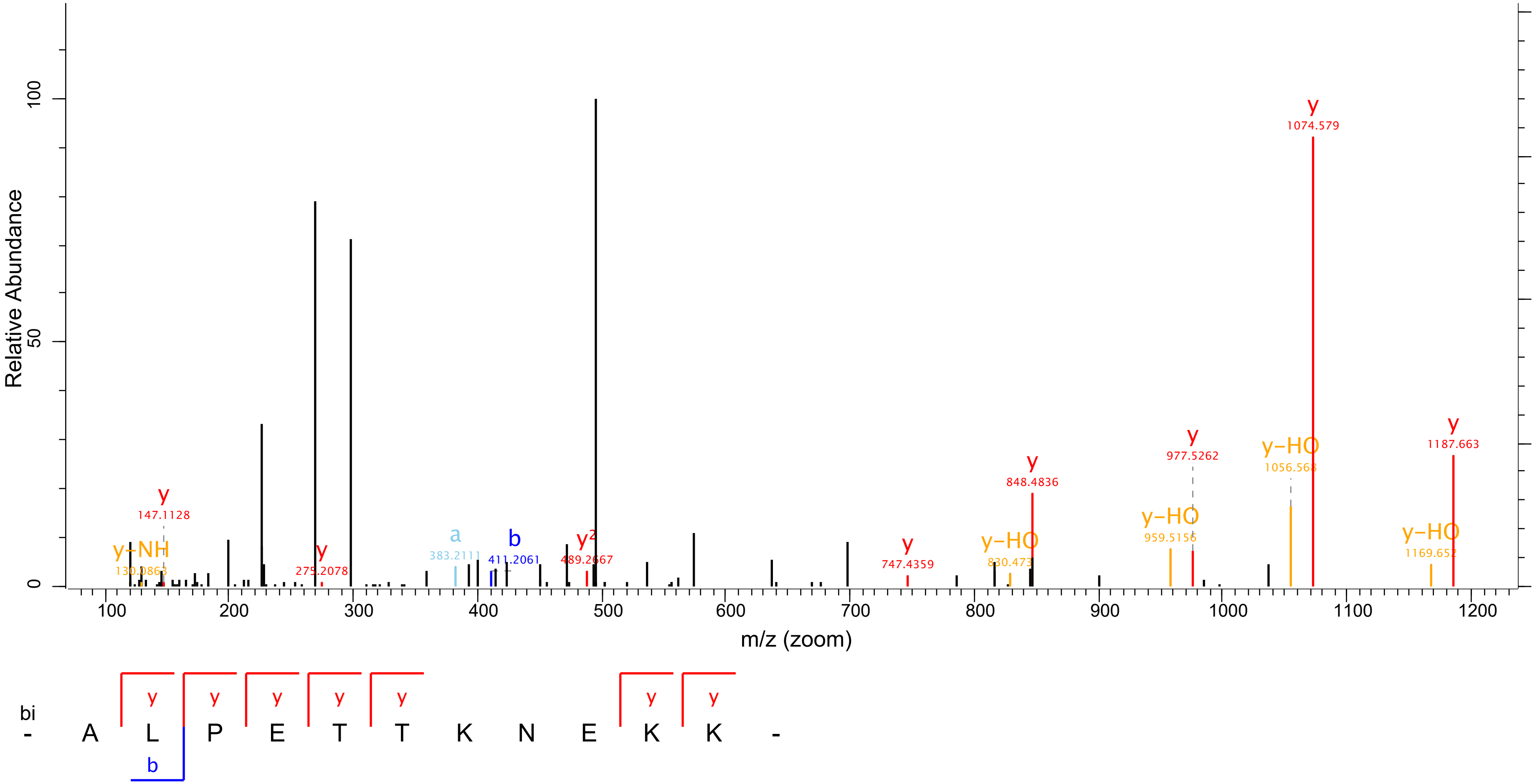

Raw File	Scan	Method	Score	m/z
agg_pep_sort_pos_1	17652	FTMS; HCD	38.03	796.37

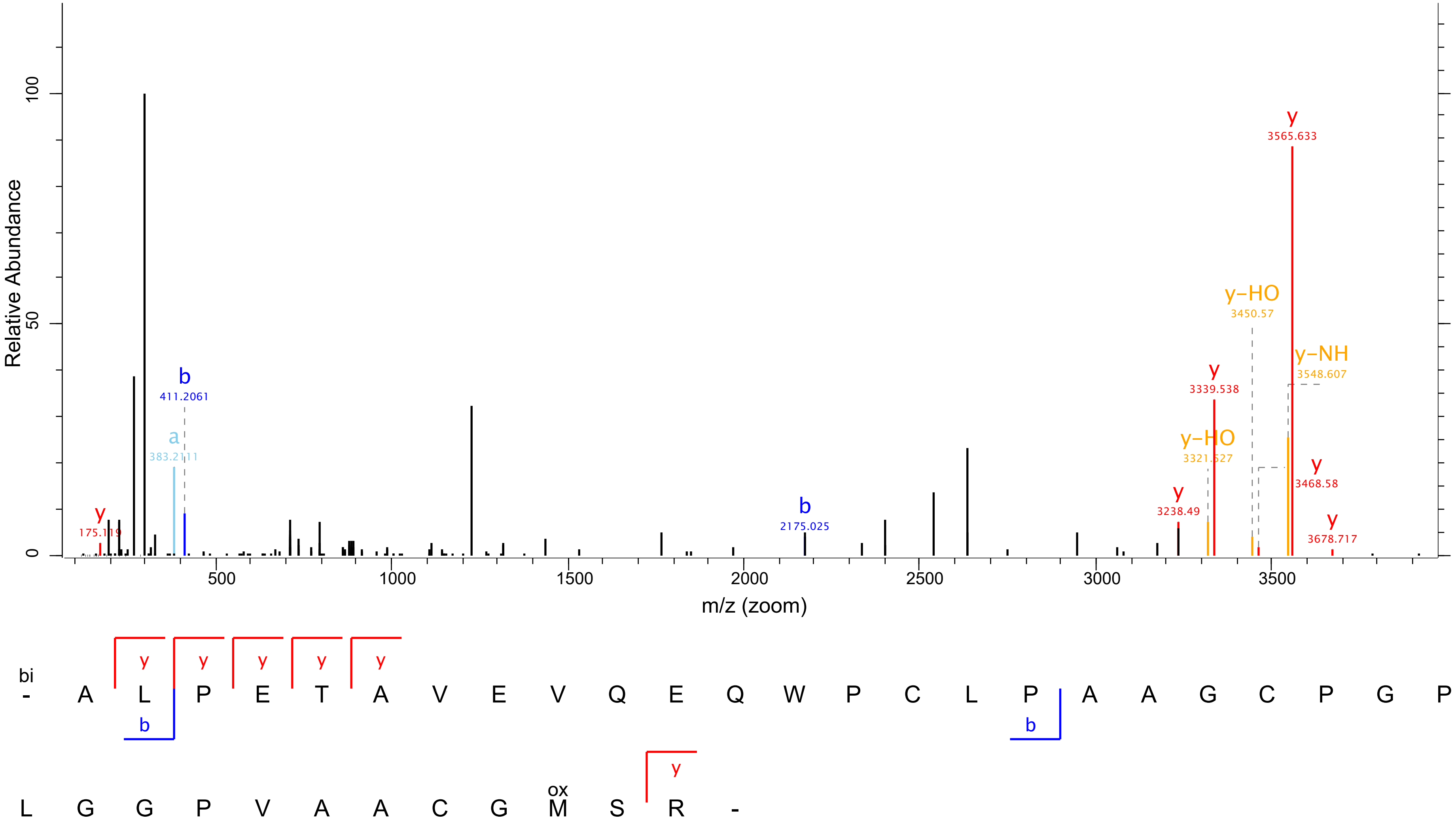

Raw File	Scan	Method	Score	m/z	Gene names
agg_pep_sort_pos_1	12771	FTMS; HCD	47.55	621	C11orf98

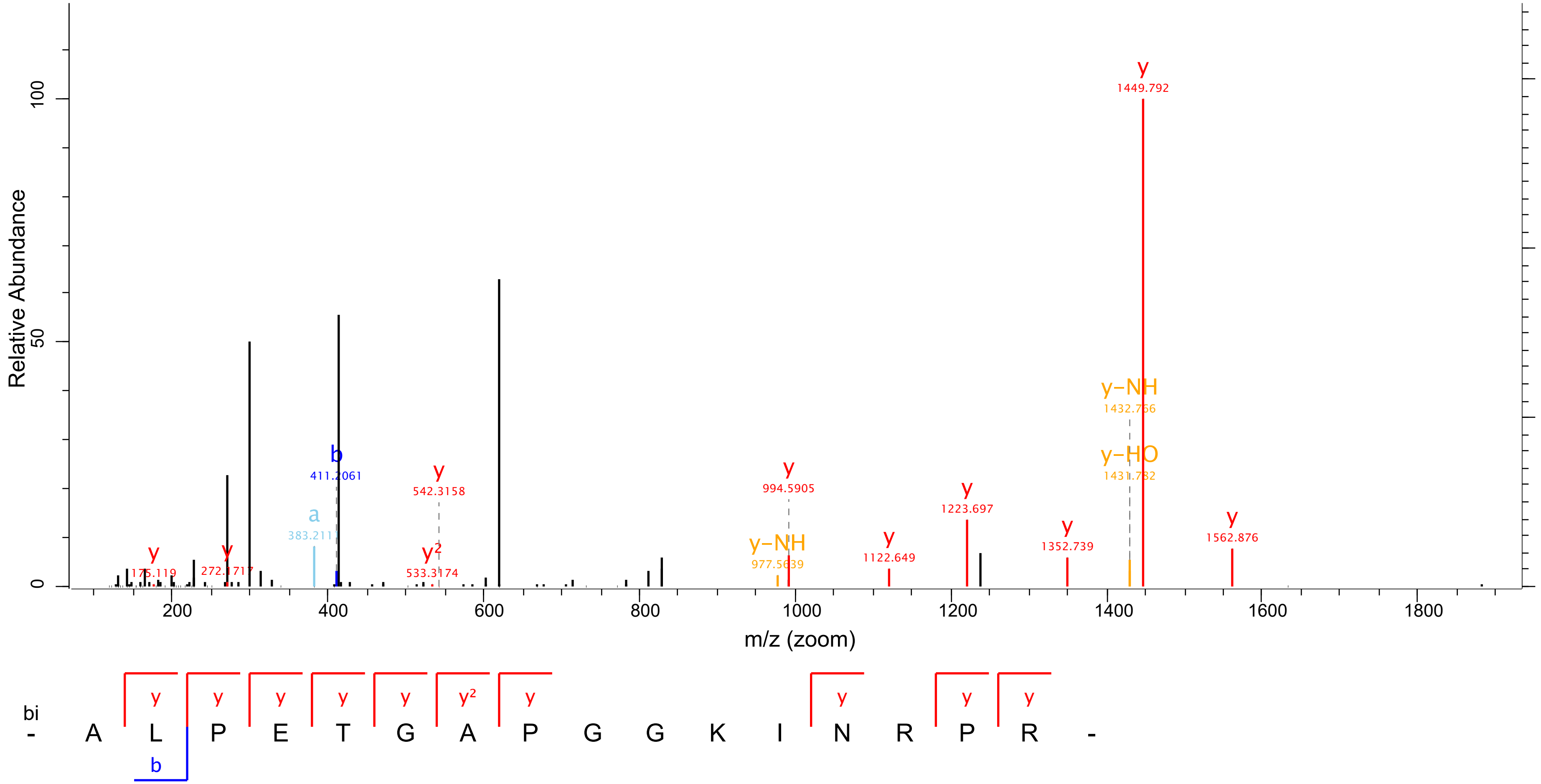

Raw File	Scan	Method	Score	m/z	Gene names
agg_pep_sort_pos_1	17941	FTMS; HCD	95.5	612.31	C11orf98

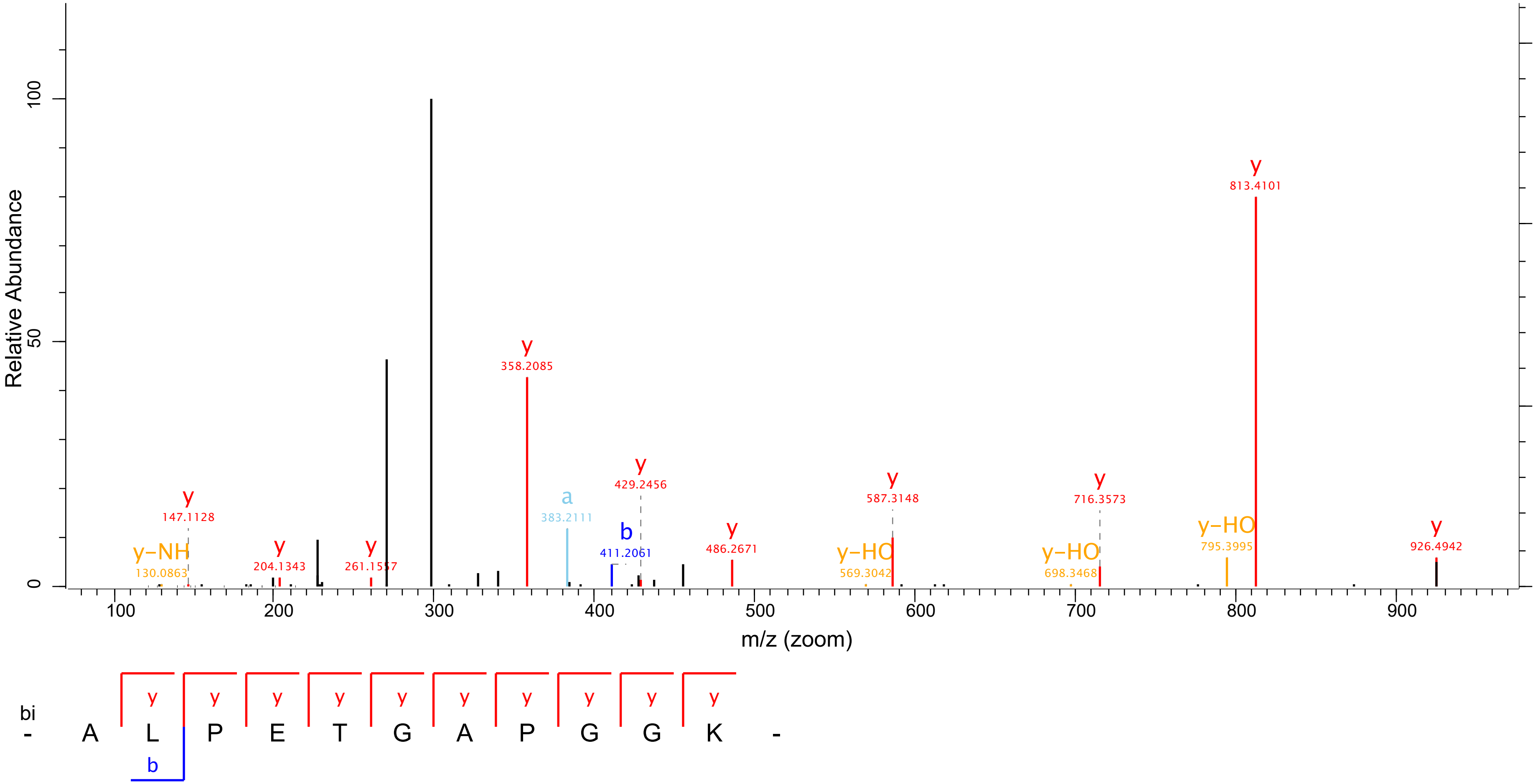

Raw File	Scan	Method	Score	m/z	Gene names
agg_pep_sort_pos_3	15078	FTMS; HCD	111.04	654.36	C8orf86

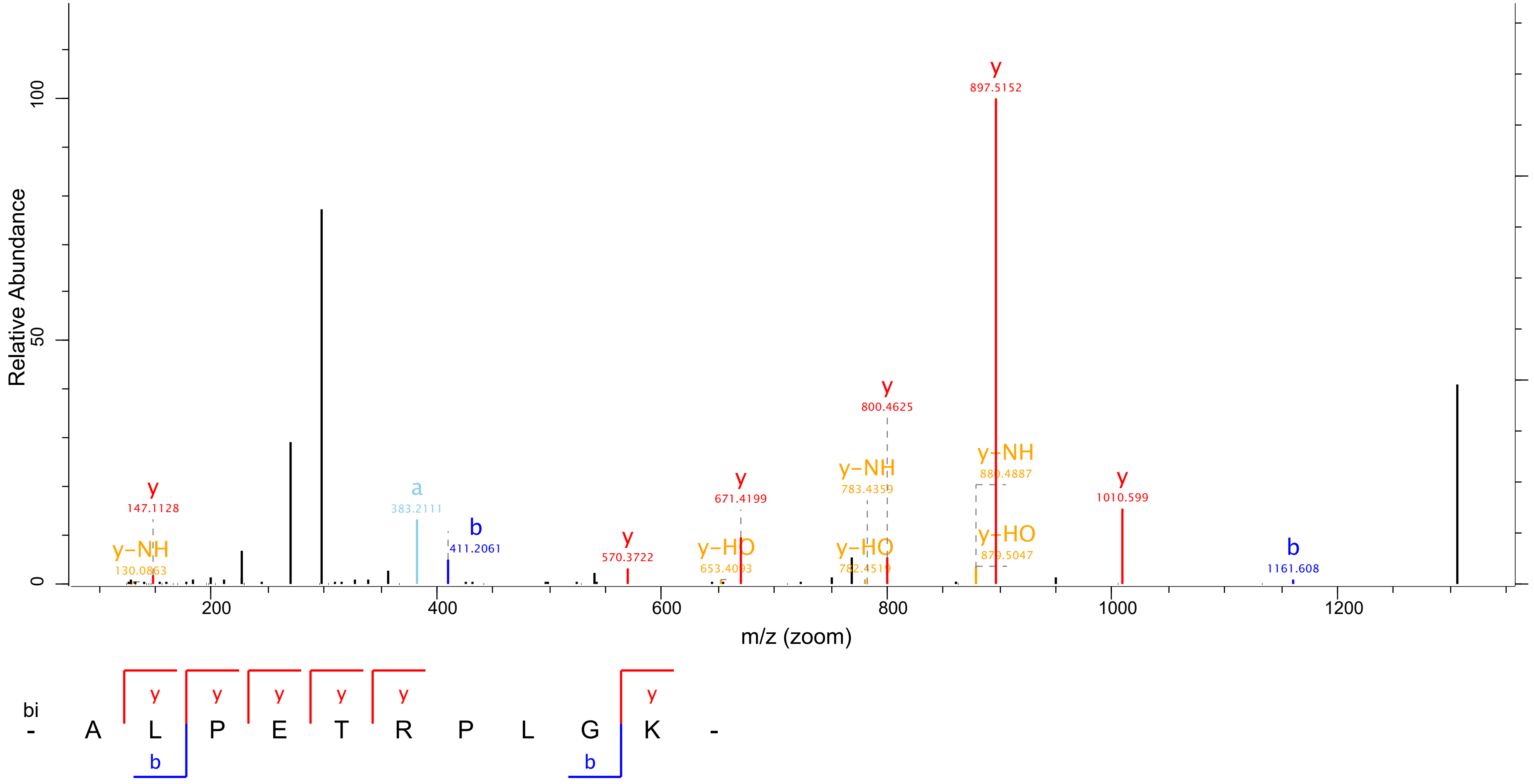

Raw File	Scan	Method	Score	m/z	Gene names
agg_pep_sort_pos_3	16400	FTMS; HCD	94.3	643.8	CABP2

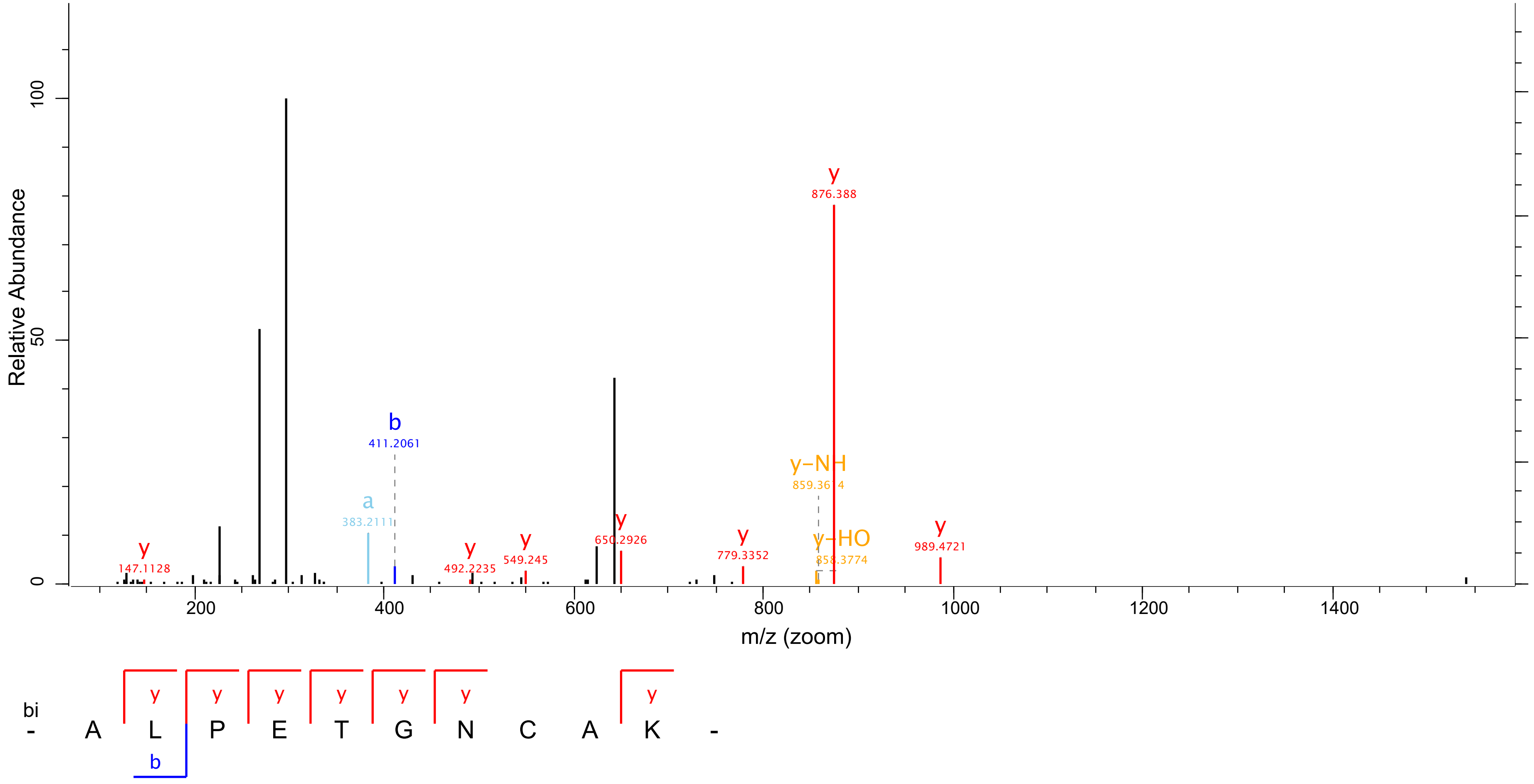

Raw File	Scan	Method	Score	m/z	Gene names
agg_pep_sort_pos_3	16400	FTMS; HCD	94.3	643.8	CABP2

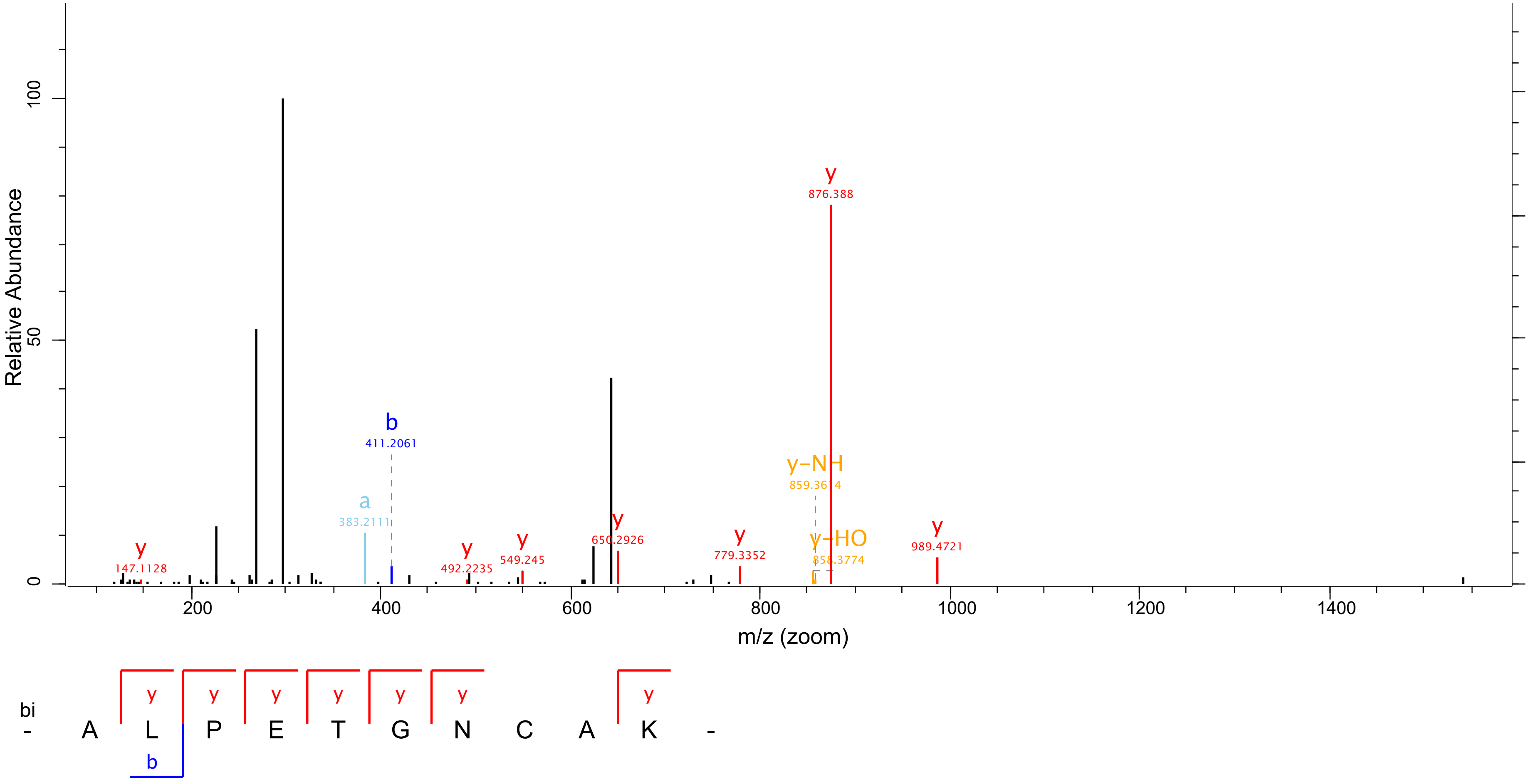

Raw File	Scan	Method	Score	m/z	Gene names
agg_pep_sort_pos_3	20007	FTMS; HCD	48.42	779.87	CALU

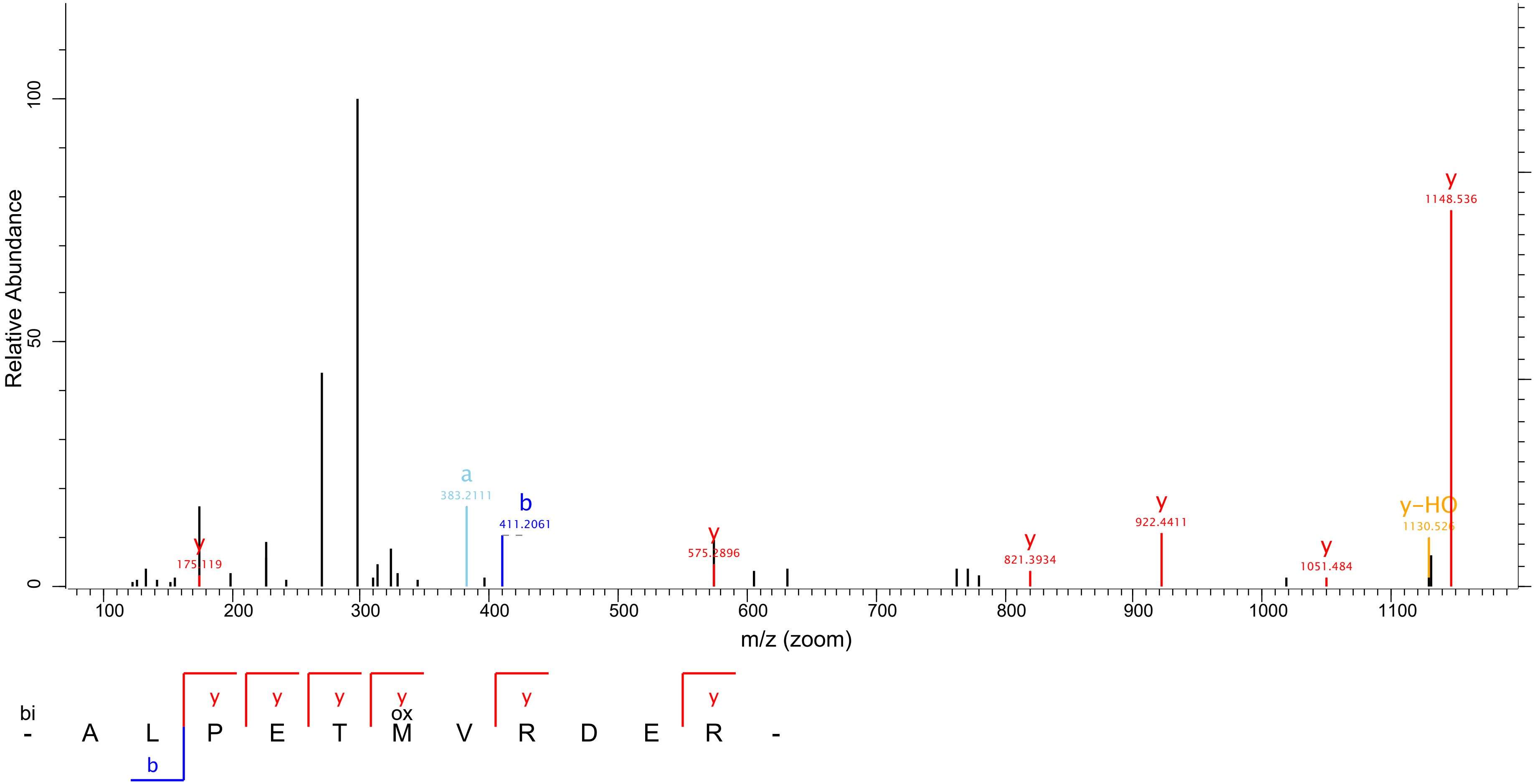

Raw File	Scan	Method	Score	m/z	Gene names
agg_pep_sort_pos_2	16537	FTMS; HCD	40.84	773.06	CBR4

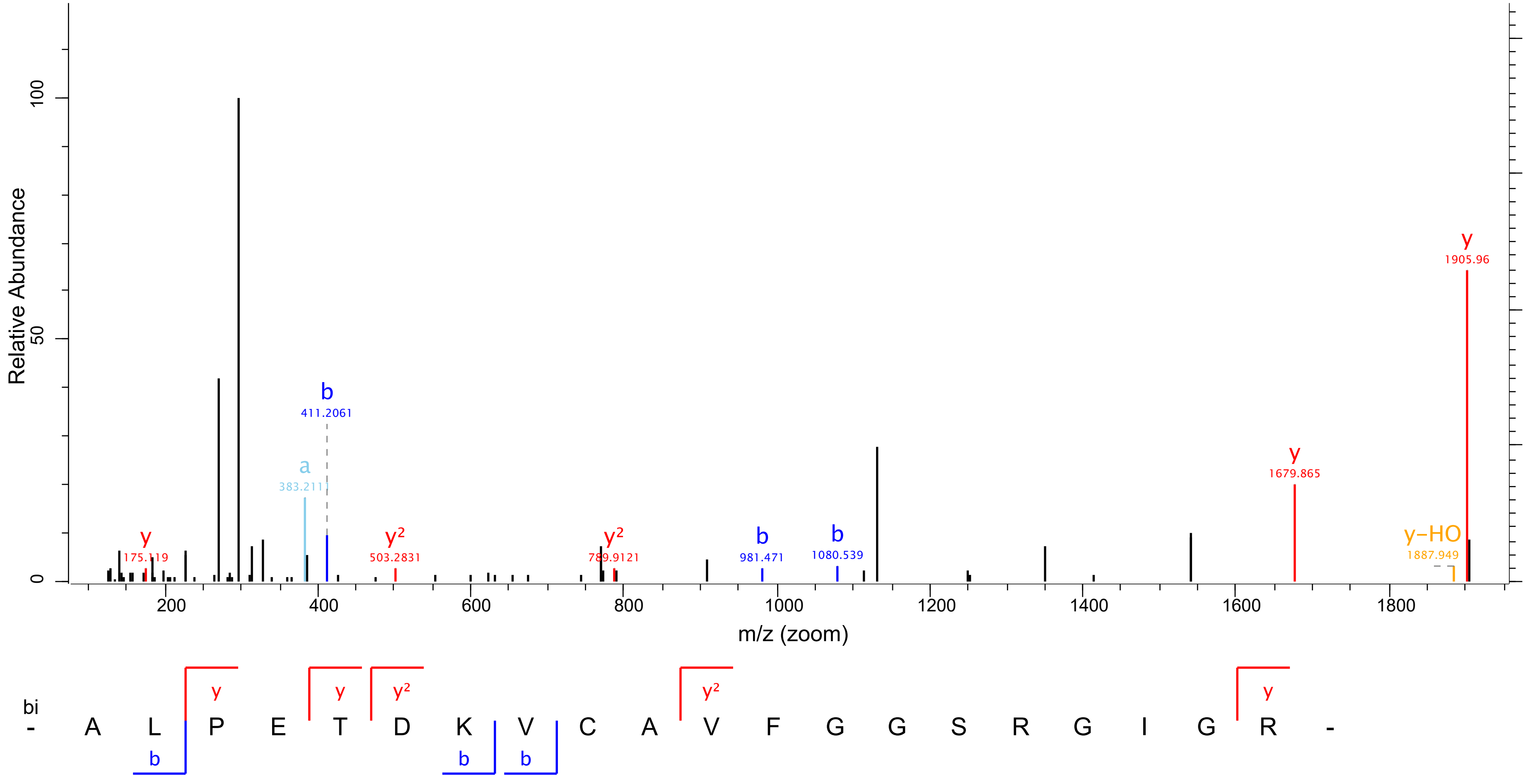

Raw File	Scan	Method	Score	m/z
agg_pep_sort_pos_1	14207	FTMS; HCD	67.99	656.34

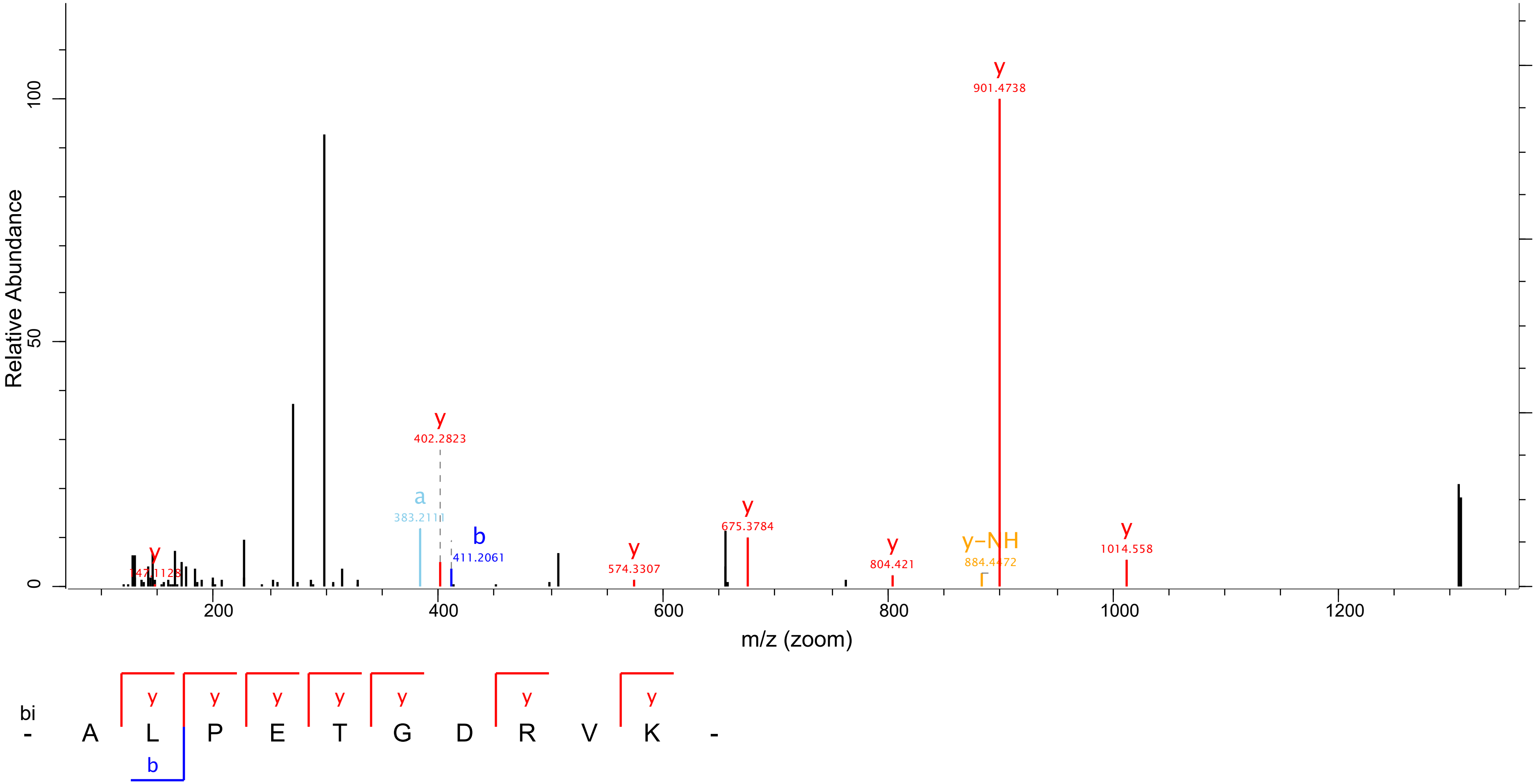

Raw File	Scan	Method	Score	m/z
agg_pep_sort_pos_3	18005	FTMS; HCD	49.93	612.65

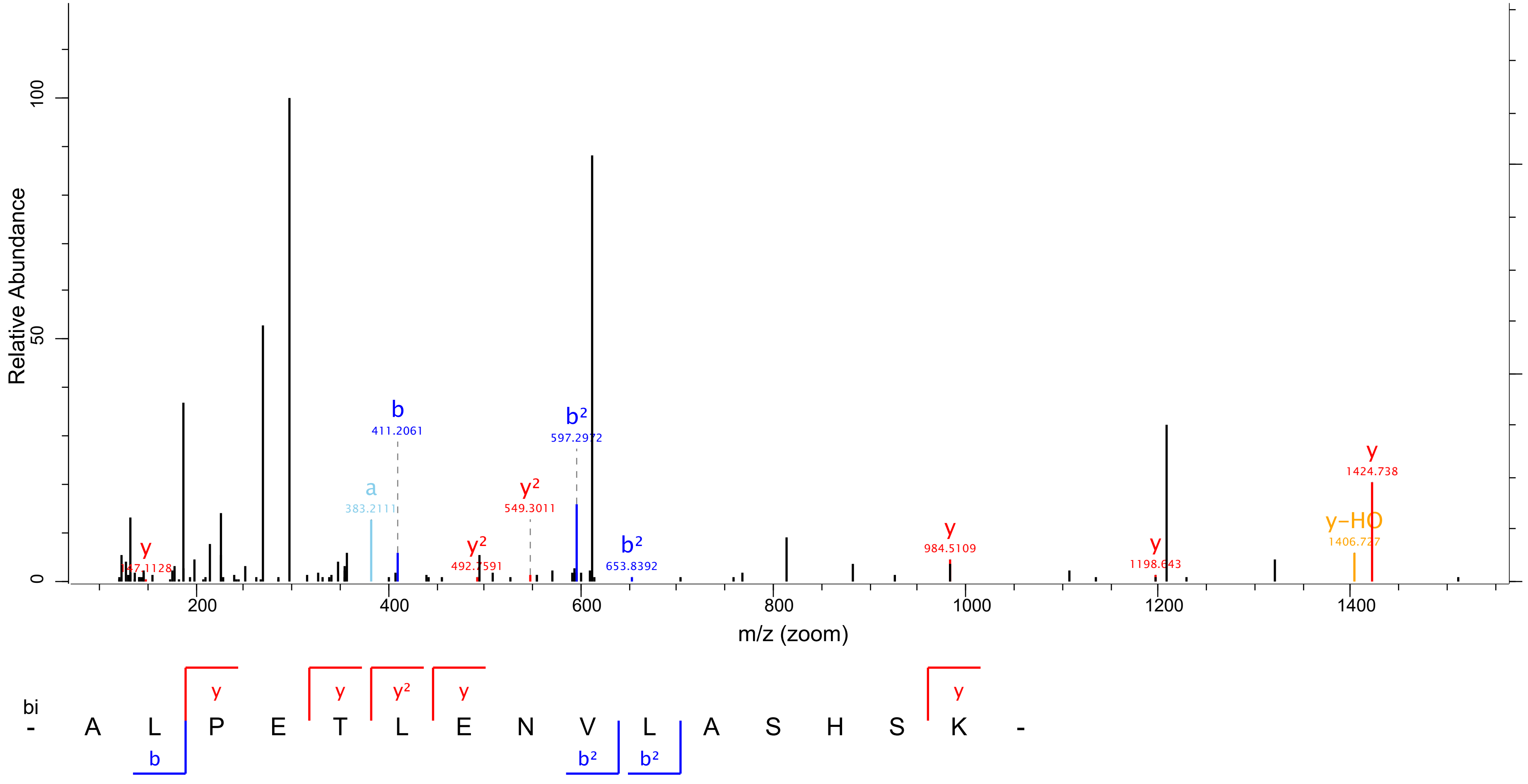

Raw File	Scan	Method	Score	m/z	Gene names
agg_pep_sort_pos_3	20012	FTMS; HCD	62.82	771.87	CDK15

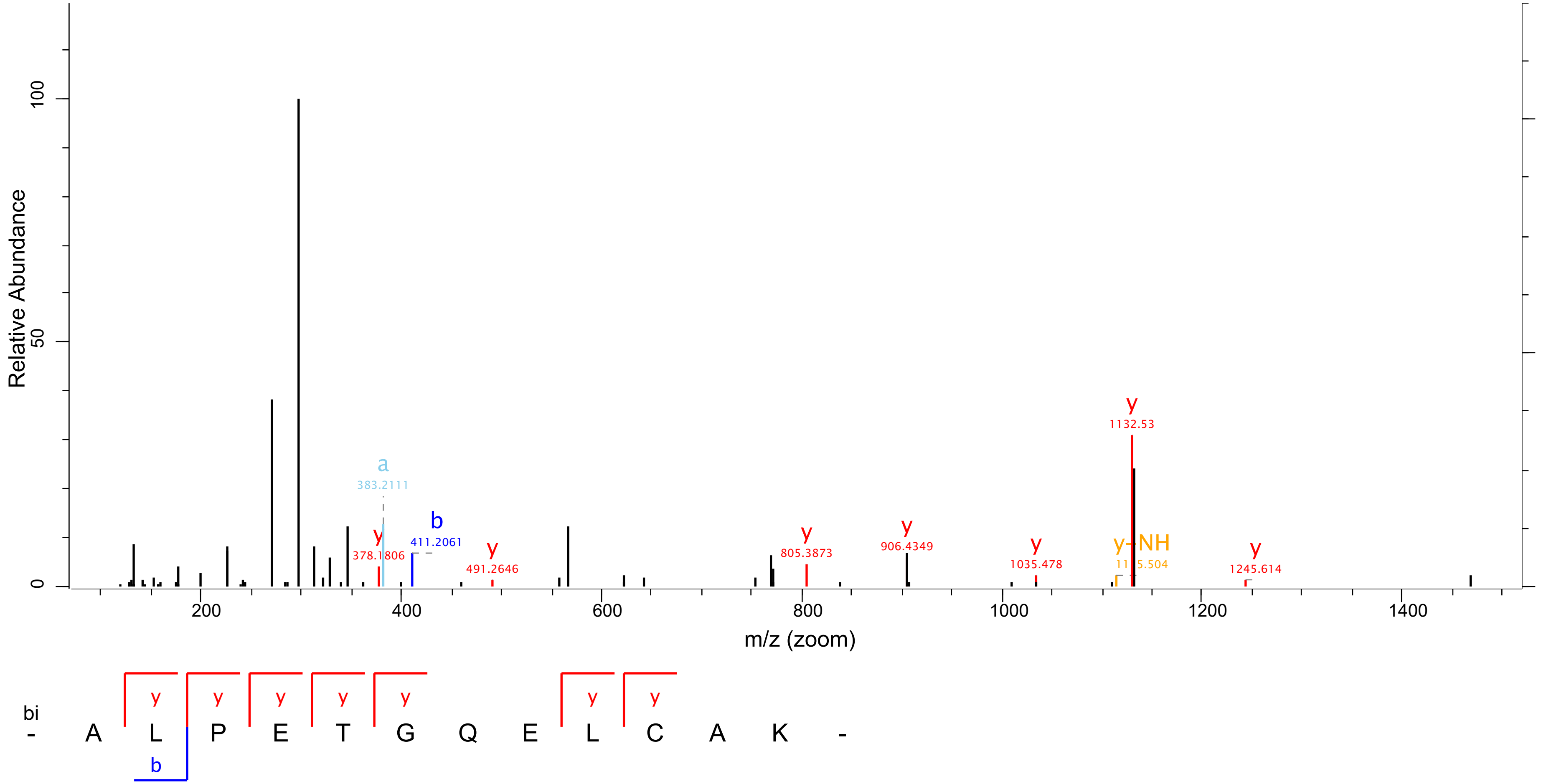

Raw File	Scan	Method	Score	m/z	Gene names
agg_pep_sort_pos_1	17383	FTMS; HCD	76.46	708.84	CHCHD3

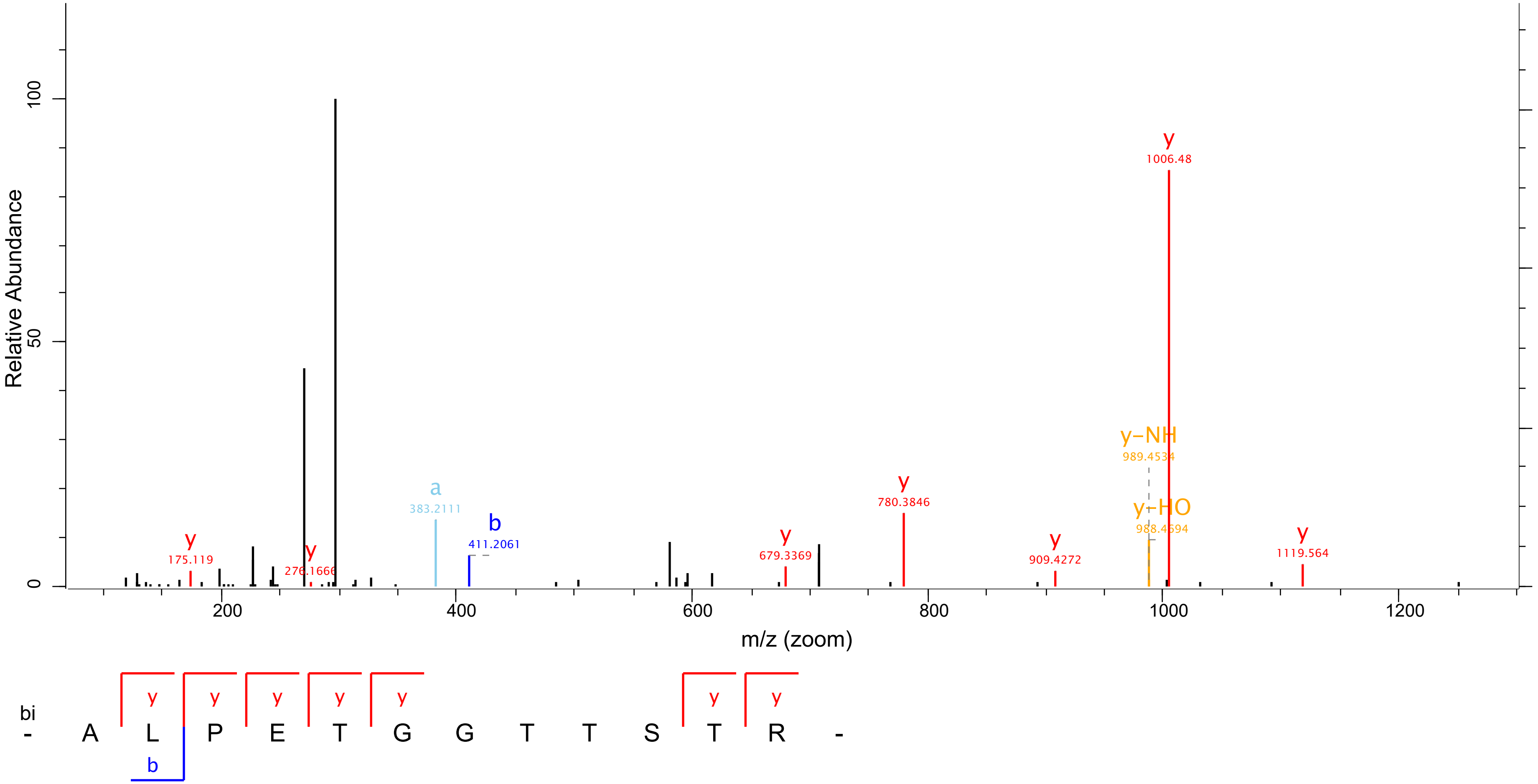

Raw File	Scan	Method	Score	m/z	Gene names
agg_pep_sort_pos_1	26620	FTMS; HCD	101.54	629.83	CHMP3

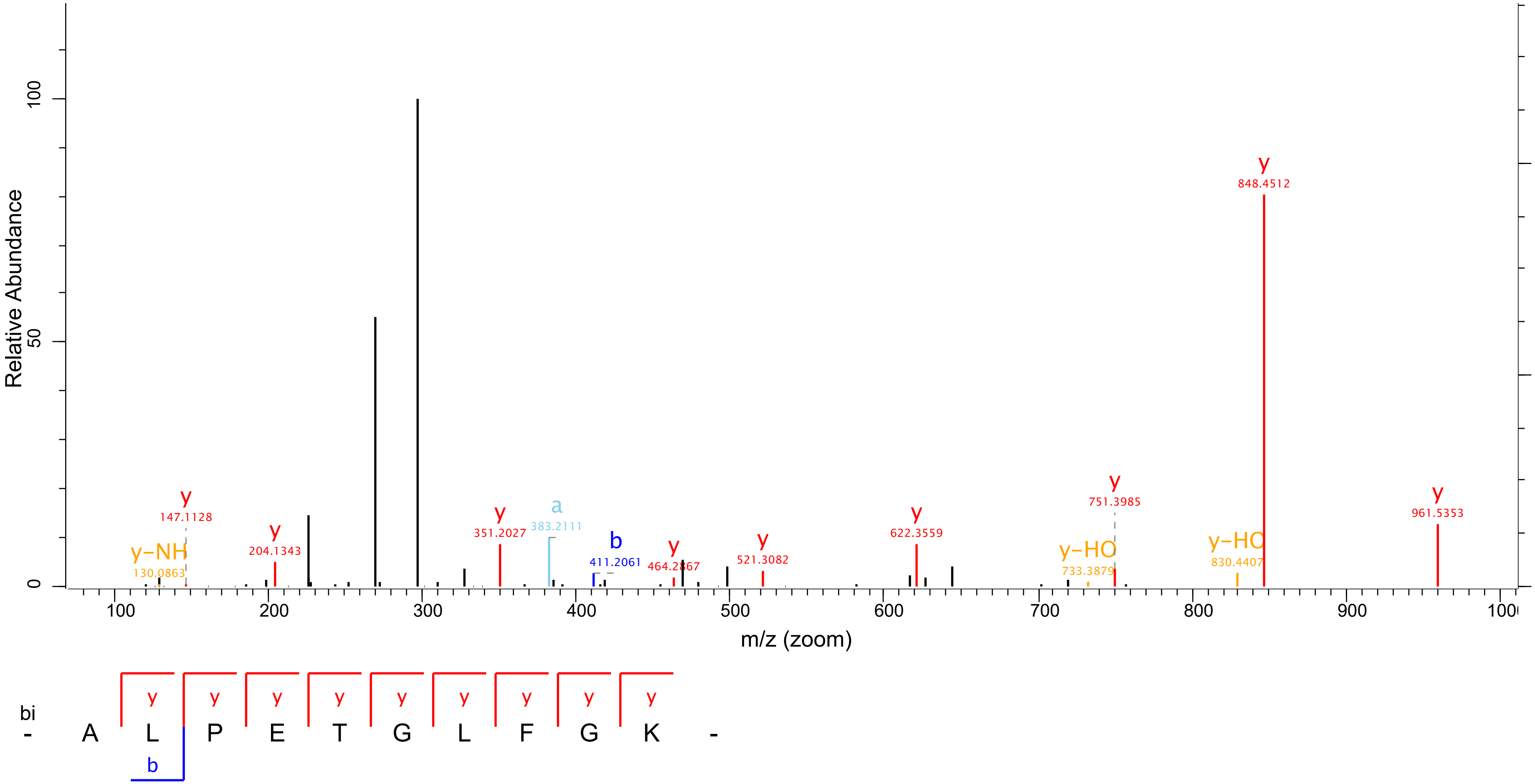

Raw File	Scan	Method	Score	m/z	Gene names
agg_pep_sort_pos_1	26498	FTMS; HCD	130.75	700.85	CHMP6

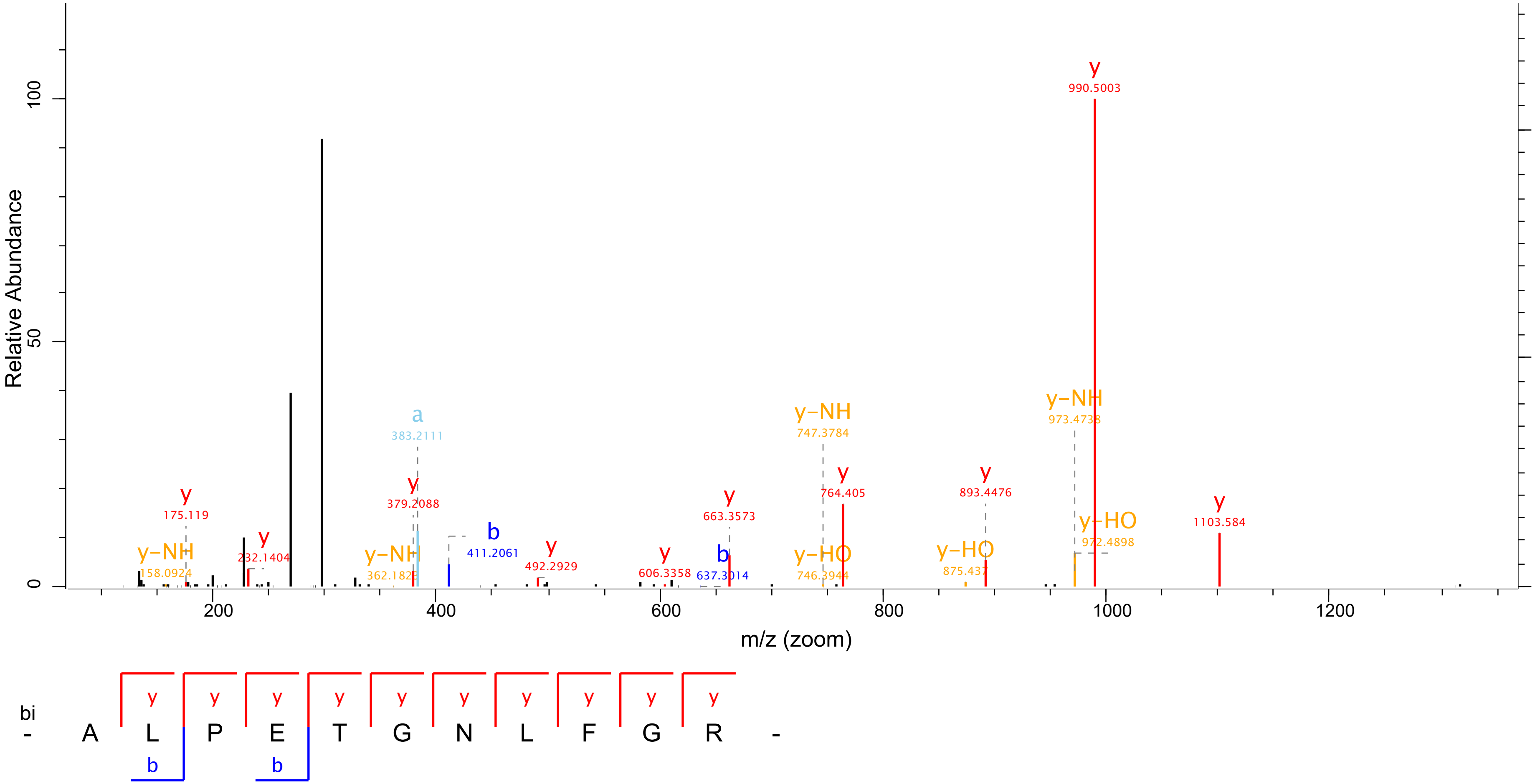

Raw File	Scan	Method	Score	m/z	Gene names
agg_pep_sort_pos_2	25802	FTMS; HCD	117.65	790.06	CKAP5

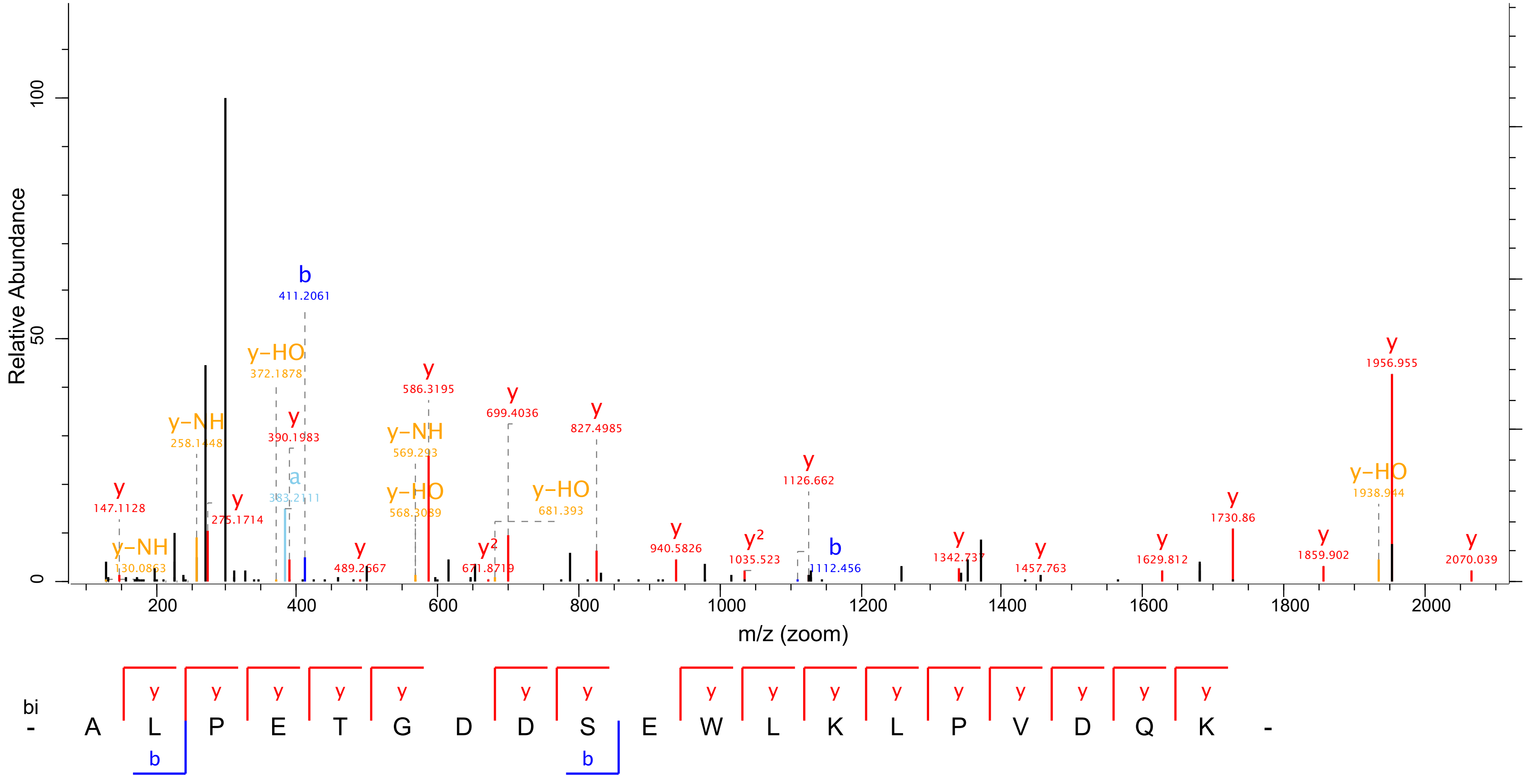

Raw File	Scan	Method	Score	m/z
agg_pep_sort_pos_2	15944	FTMS; HCD	75.65	815.12

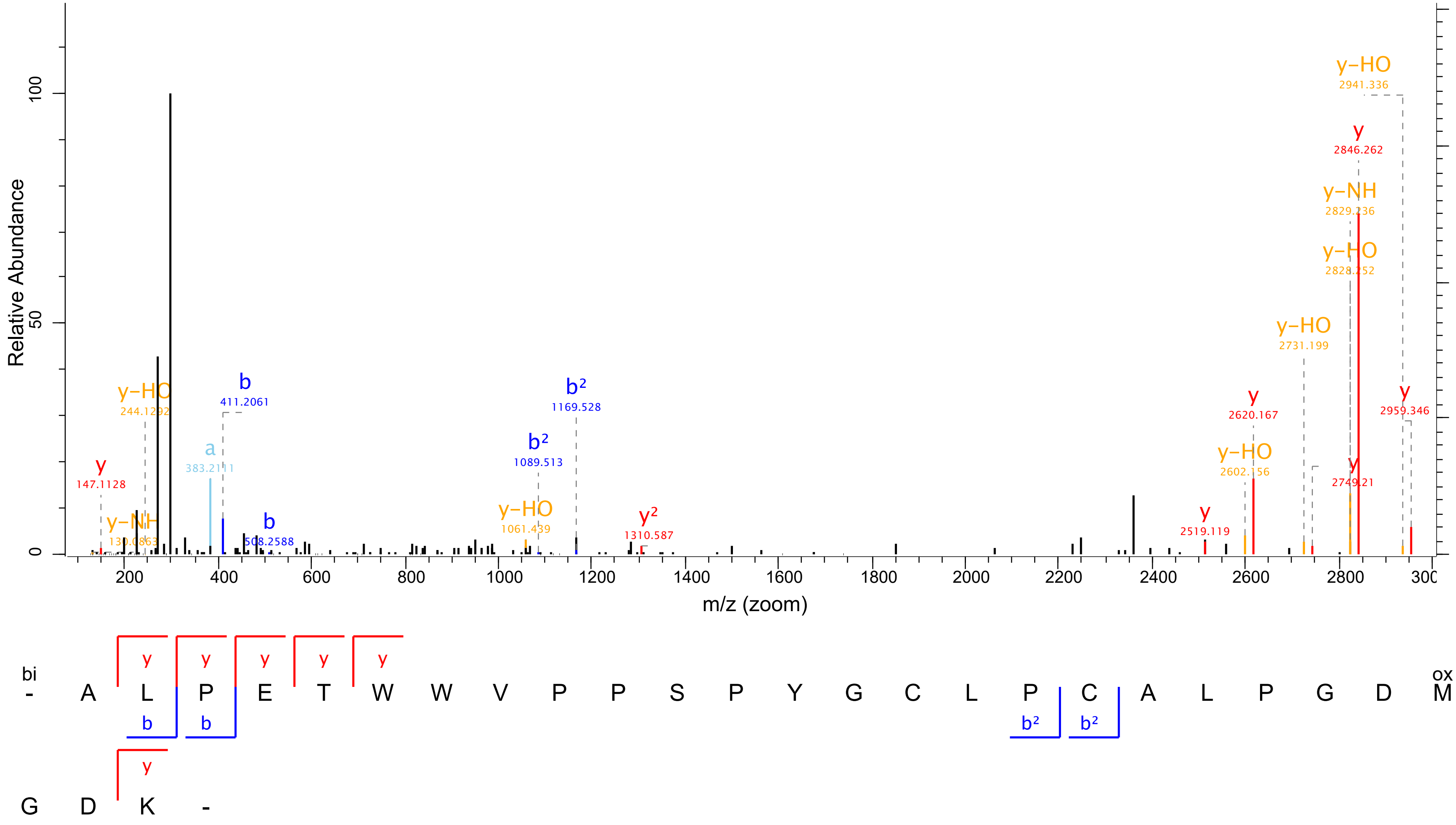

Raw File	Scan	Method	Score	m/z
agg_pep_sort_pos_2	12993	FTMS; HCD	77.38	785.88

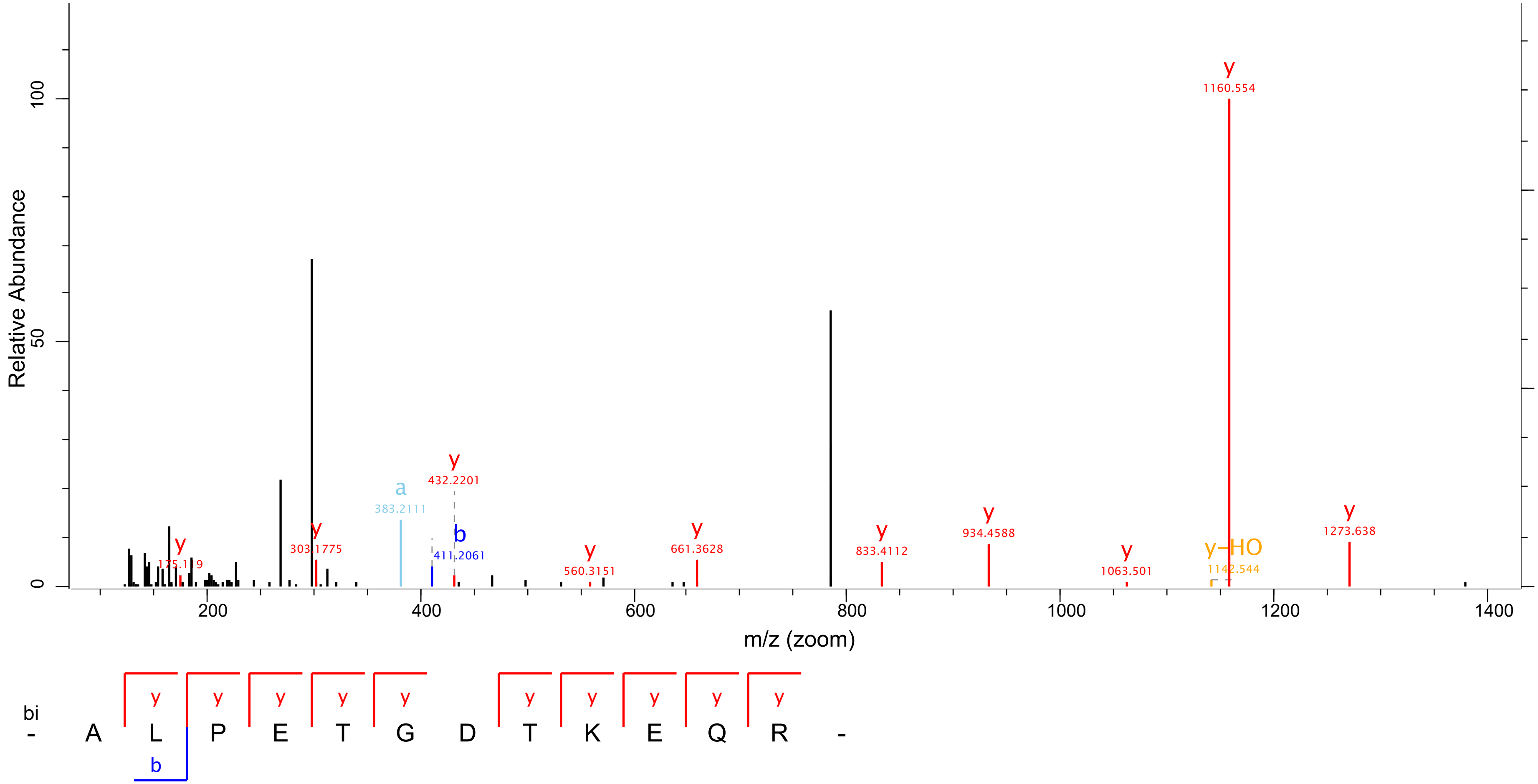

Raw File	Scan	Method	Score	m/z	Gene names
agg_pep_sort_pos_1	10383	FTMS; HCD	46.32	448.48	DDAH1

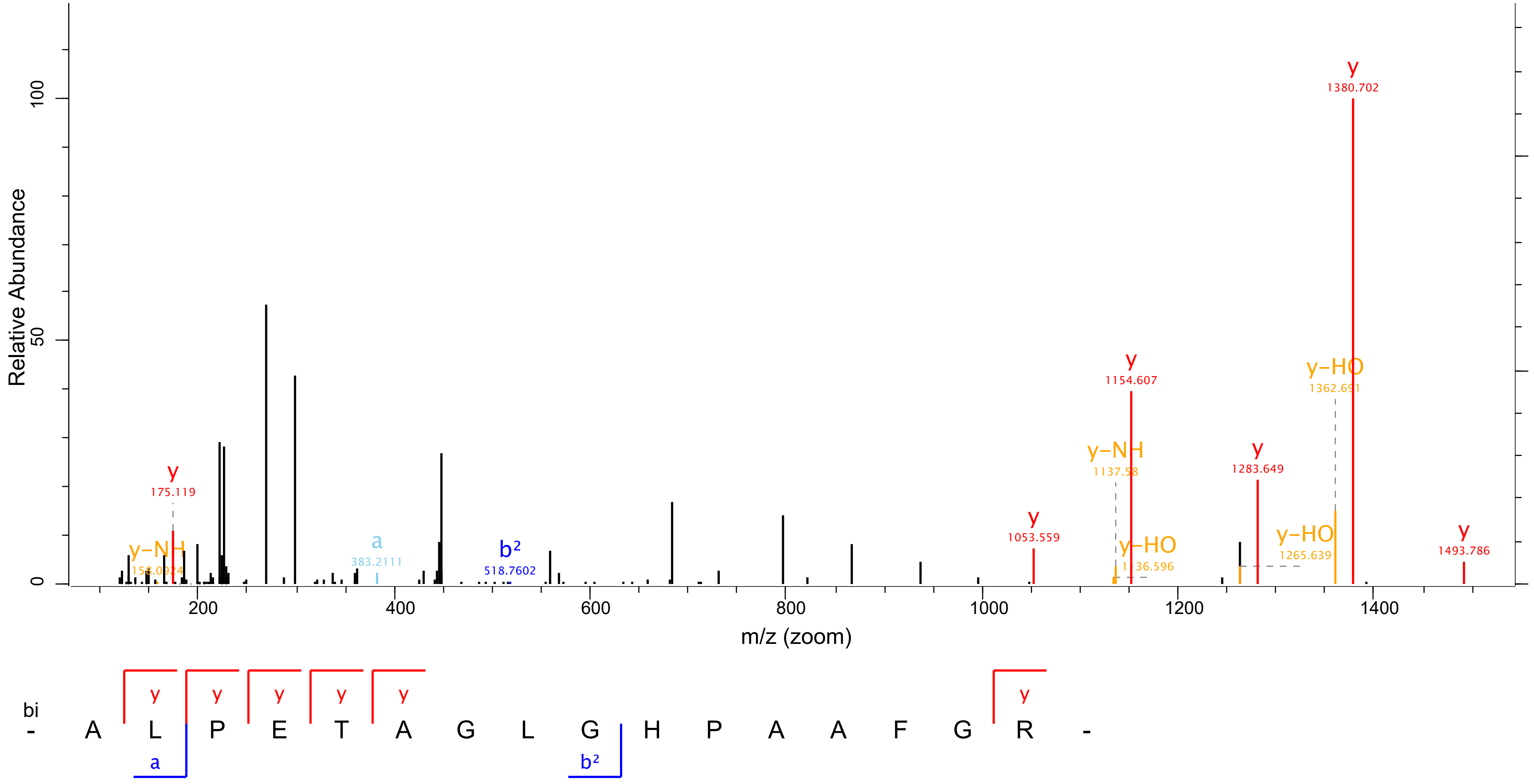

Raw File	Scan	Method	Score	m/z	Gene names
agg_pep_sort_pos_1	15482	FTMS; HCD	115.46	798.71	DNTTIP1

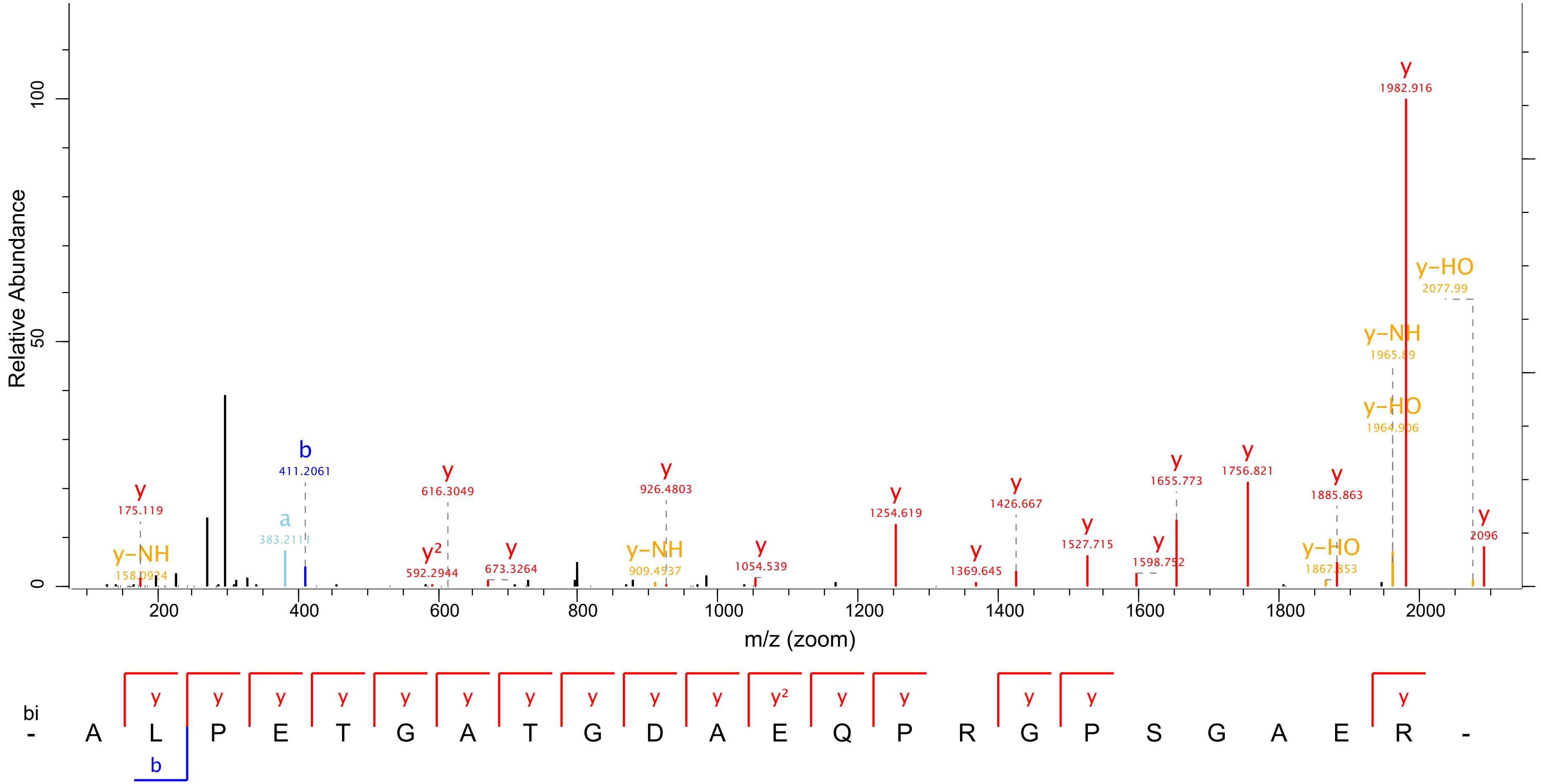

Raw File	Scan	Method	Score	m/z	Gene names
agg_pep_sort_pos_1	18883	FTMS; HCD	82.17	870.41	DNTTIP1

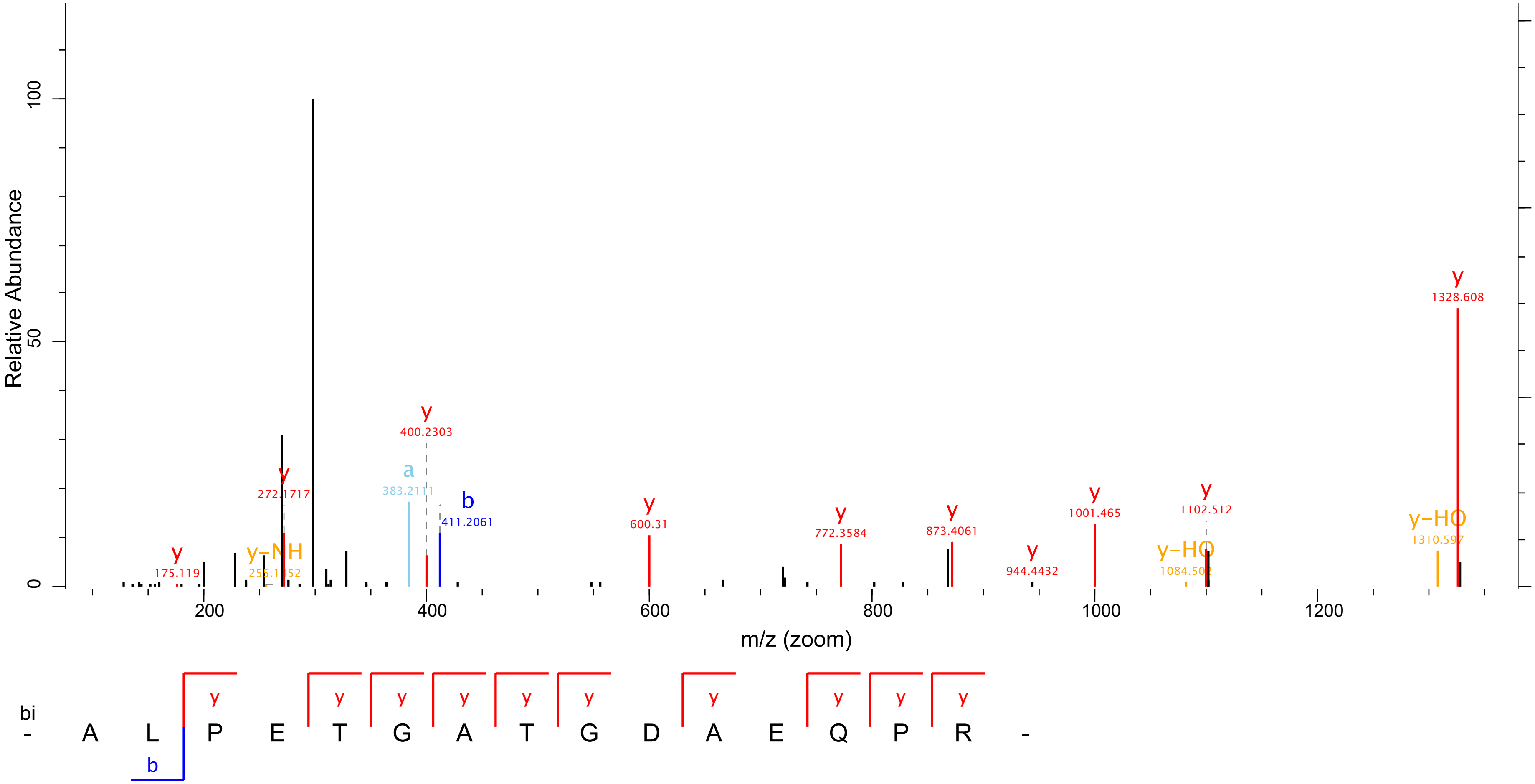

Raw File	Scan	Method	Score	m/z	Gene names
agg_pep_sort_pos_2	17592	FTMS; HCD	67.9	648.32	DPEP3

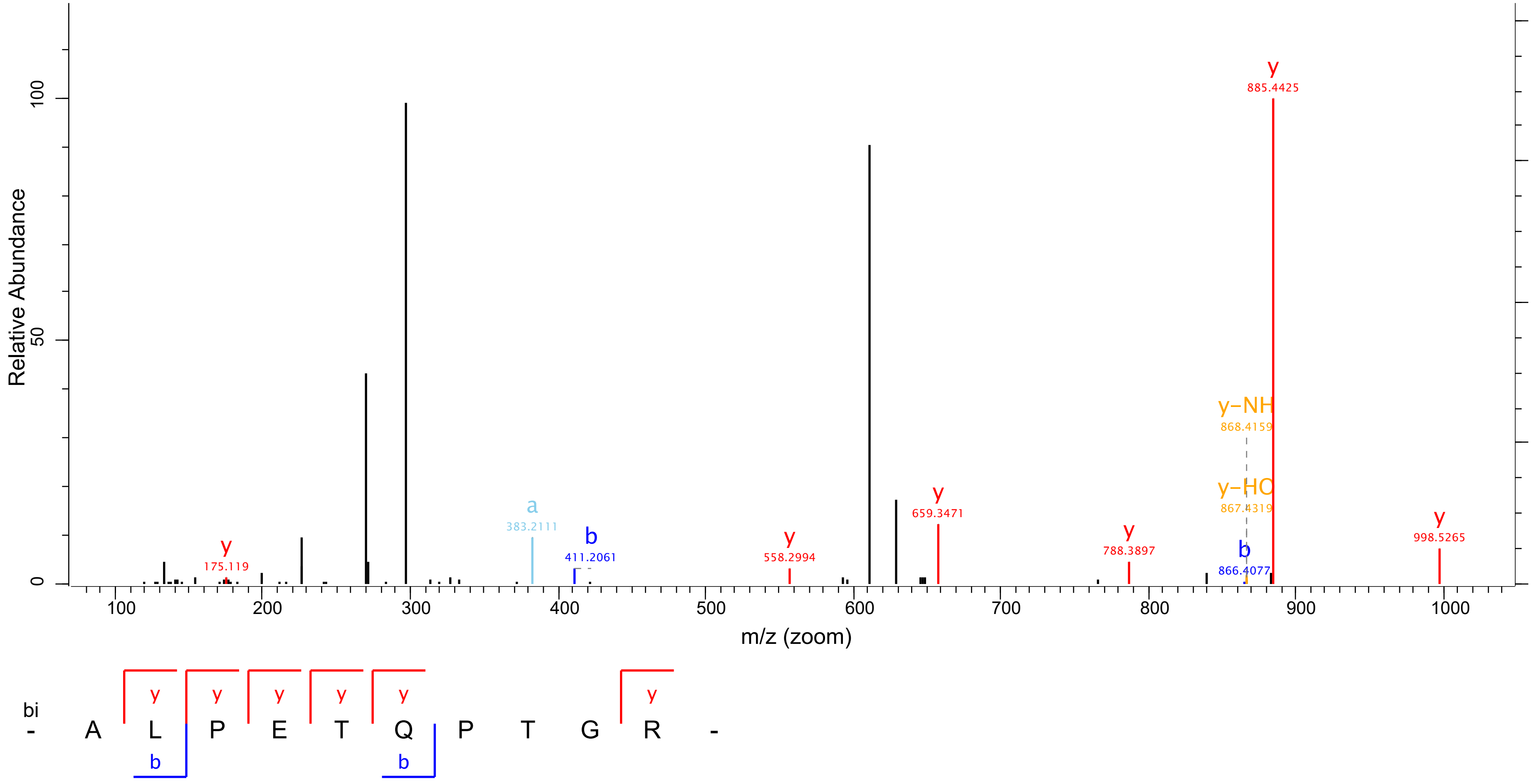

Raw File	Scan	Method	Score	m/z	Gene names
agg_pep_sort_pos_2	25042	FTMS; HCD	94.69	687.84	EEF1B2

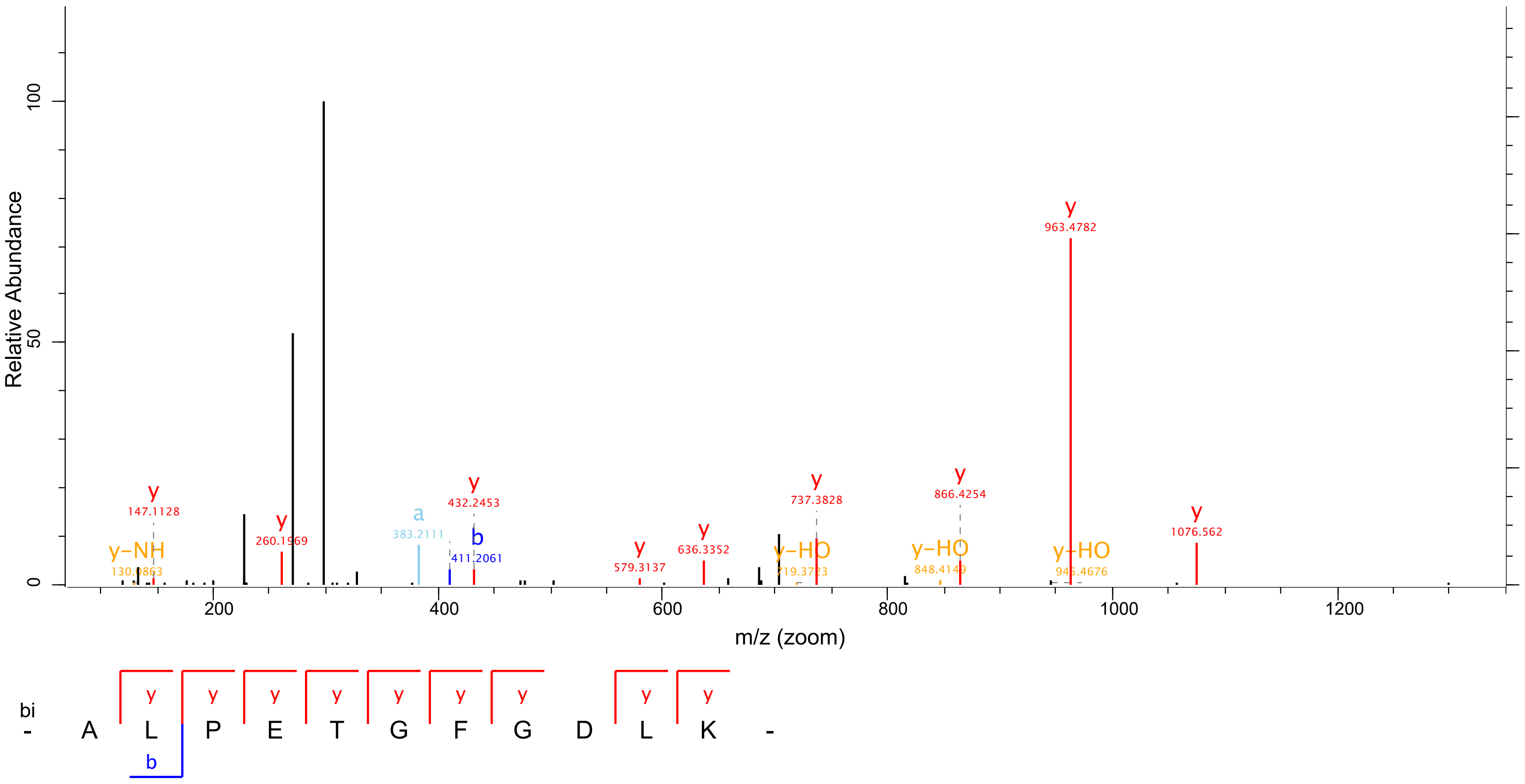

Raw File	Scan	Method	Score	m/z	Gene names
agg_pep_sort_pos_1	17488	FTMS; HCD	55.55	781.43	EZR;RDX

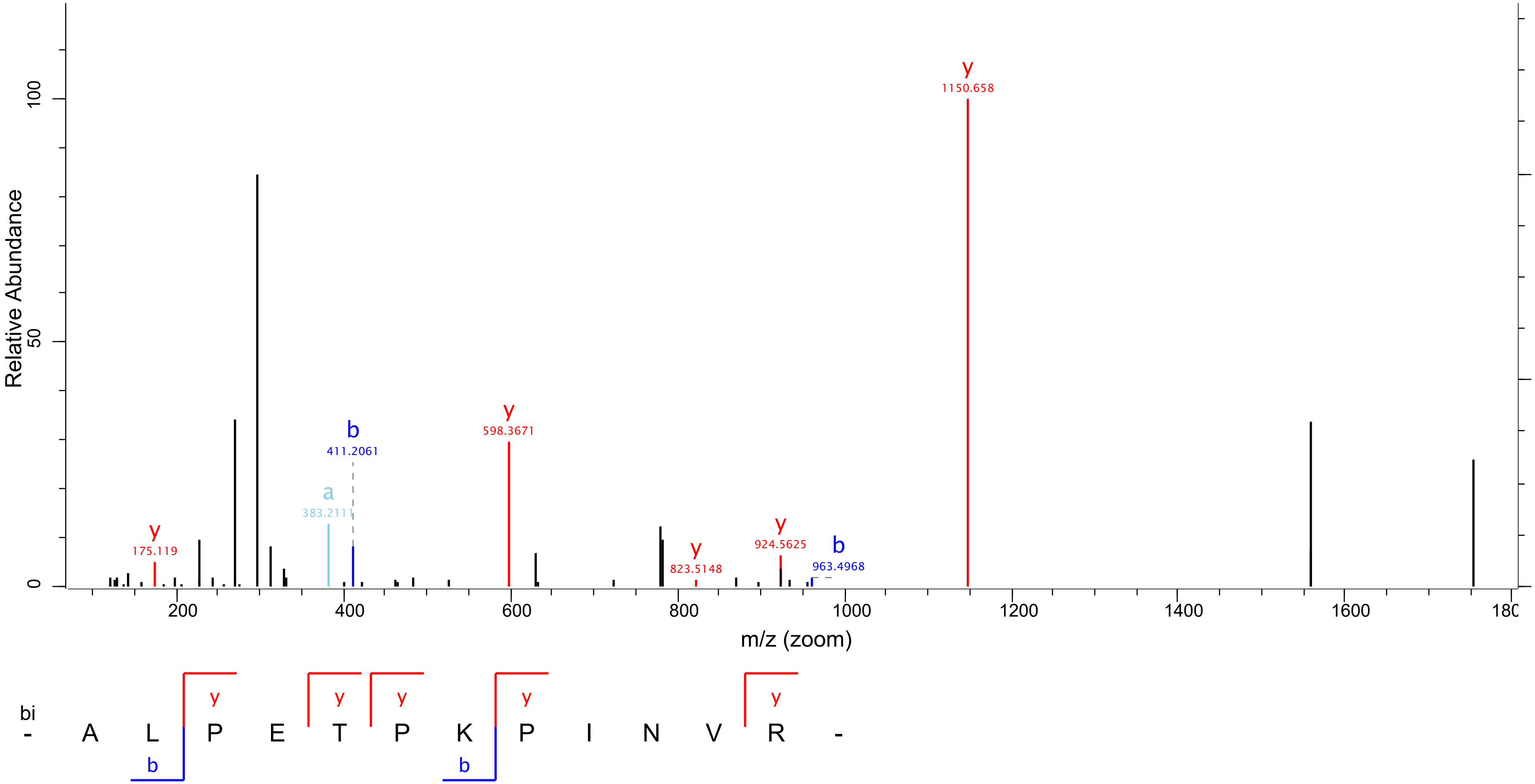

Raw File	Scan	Method	Score	m/z	Gene names
agg_pep_sort_pos_3	18442	FTMS; HCD	77.68	846.4	FAM129A

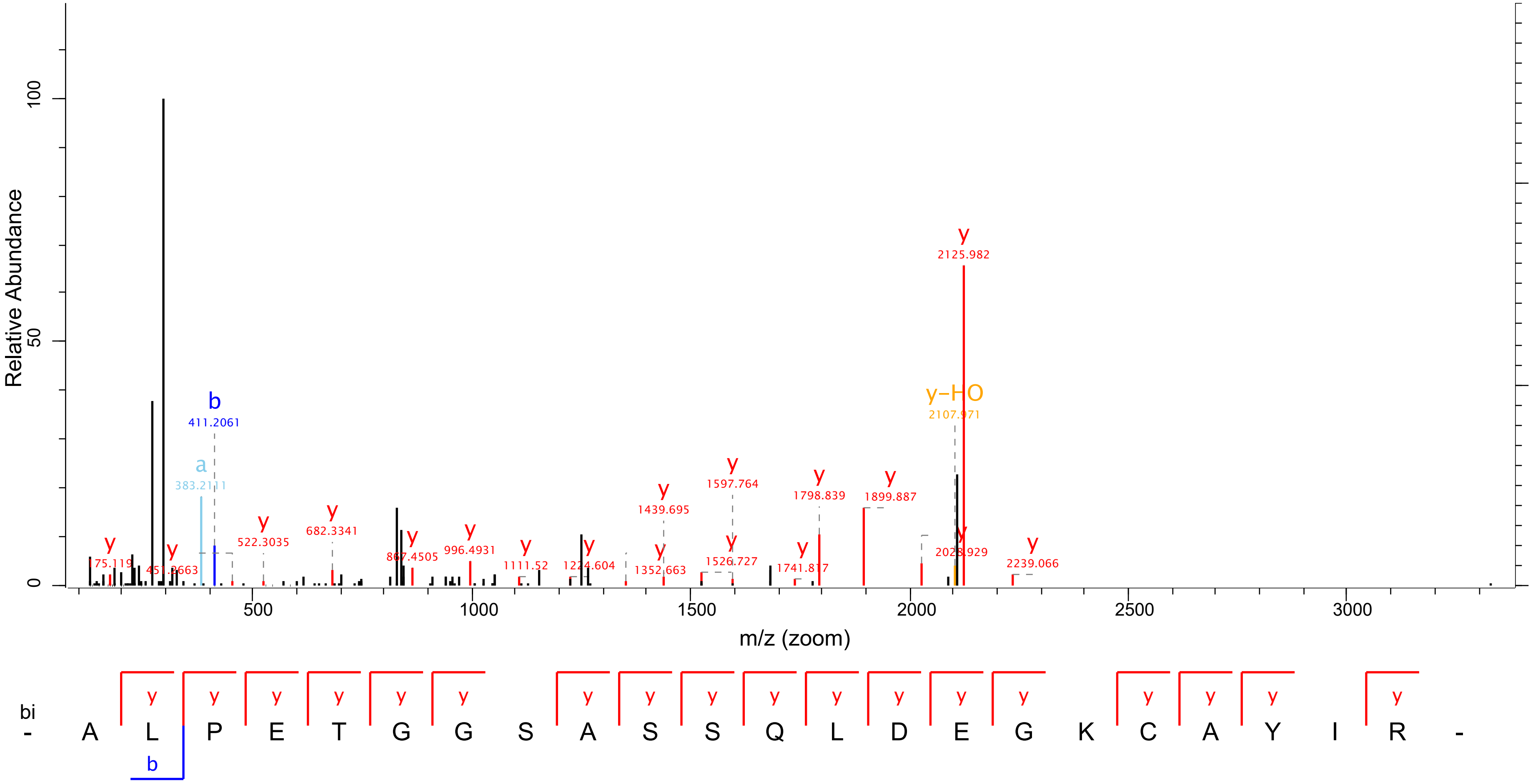

Raw File	Scan	Method	Score	m/z	Gene names
agg_pep_sort_pos_1	19601	FTMS; HCD	143.86	936.94	FAM129A

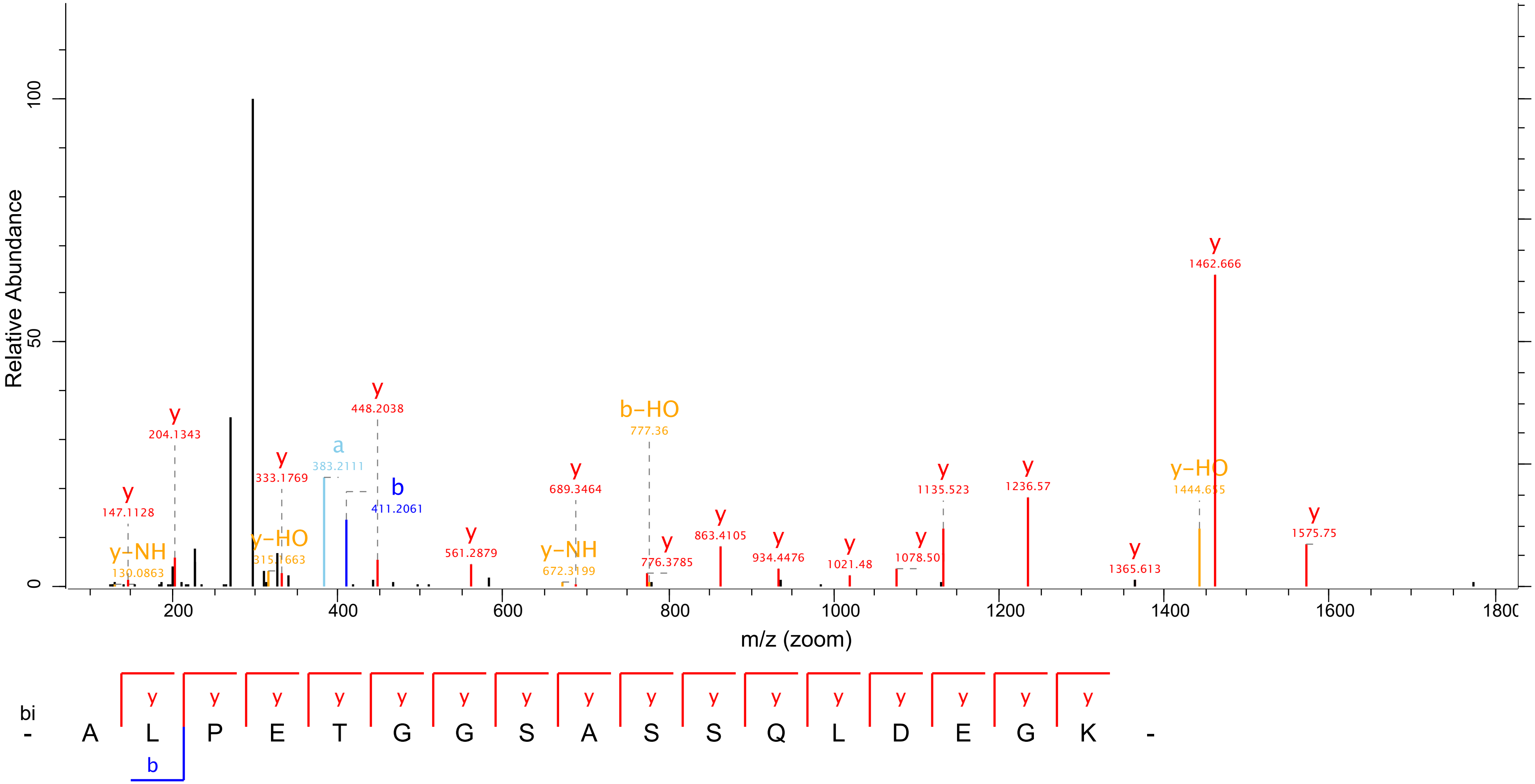

Raw File	Scan	Method	Score	m/z	Gene names
agg_pep_sort_pos_3	21095	FTMS; HCD	80.24	685.33	FAM216B

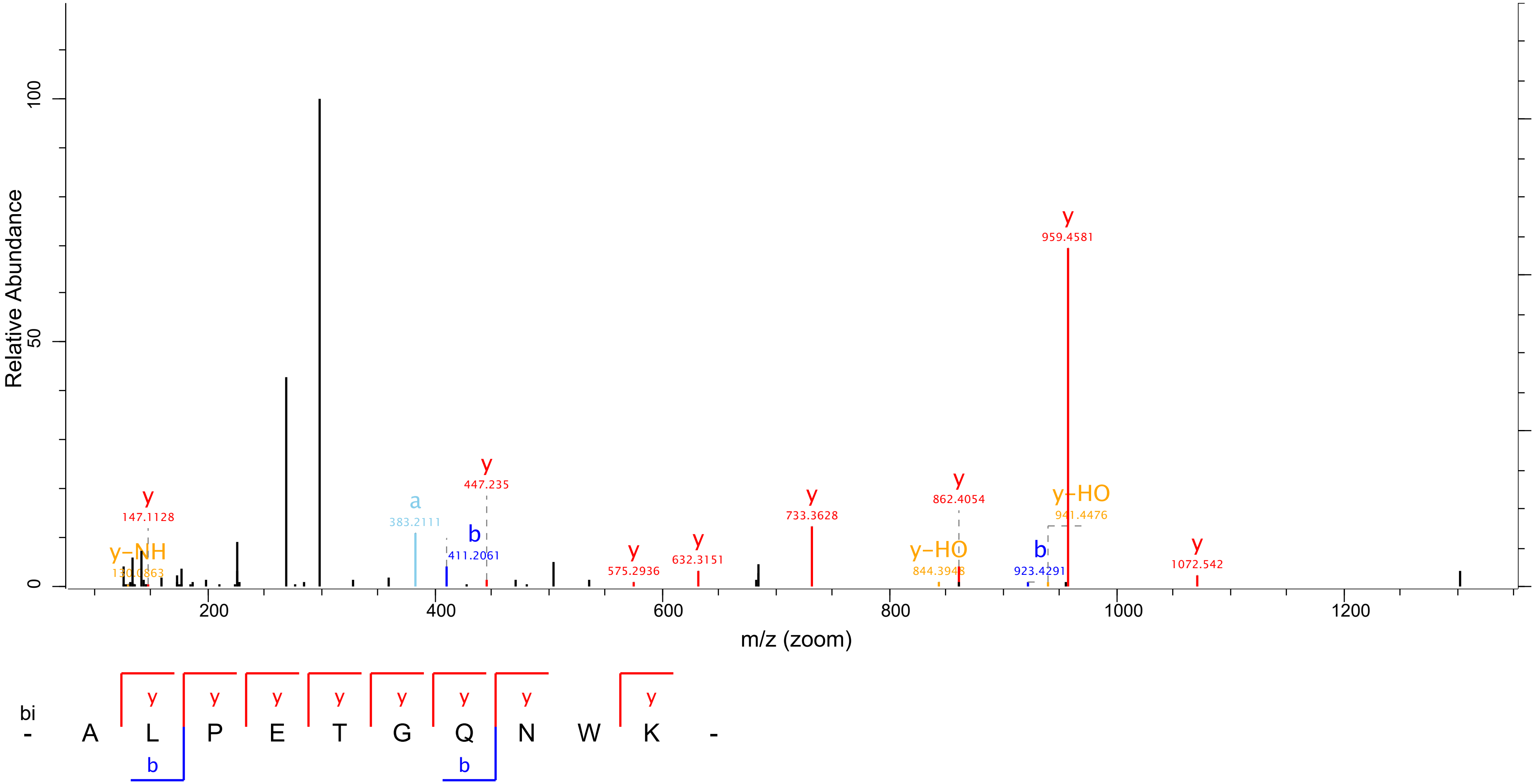

Raw File	Scan	Method	Score	m/z	Gene names
agg_pep_sort_pos_2	23260	FTMS; HCD	122.13	712.88	FEN1

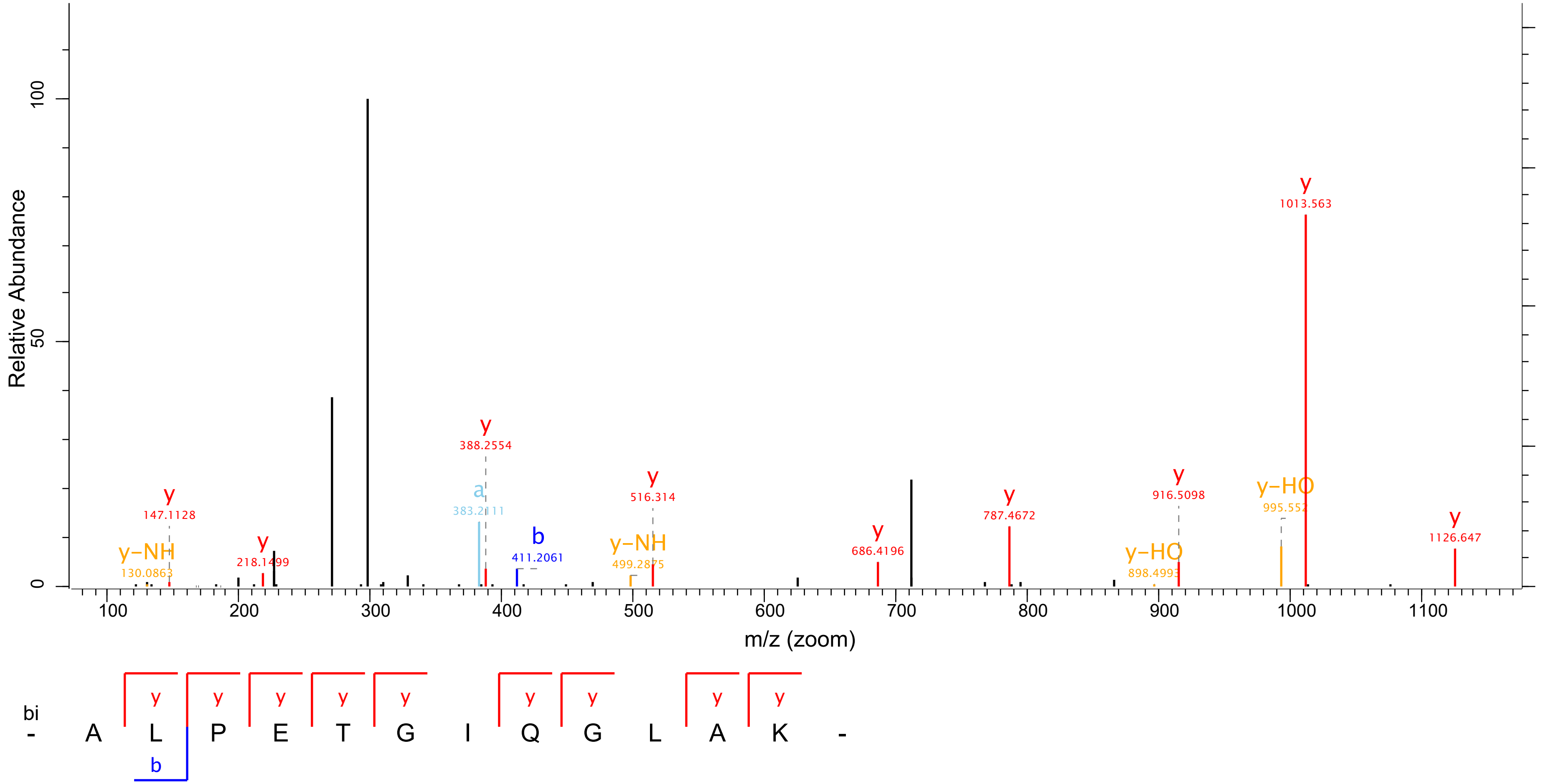

Raw File agg_pep_sort_pos_1 | Scan 24289 | Method FTMS; HCD | Score 124.86 | m/z 1027.01 | Gene names FKBP1A

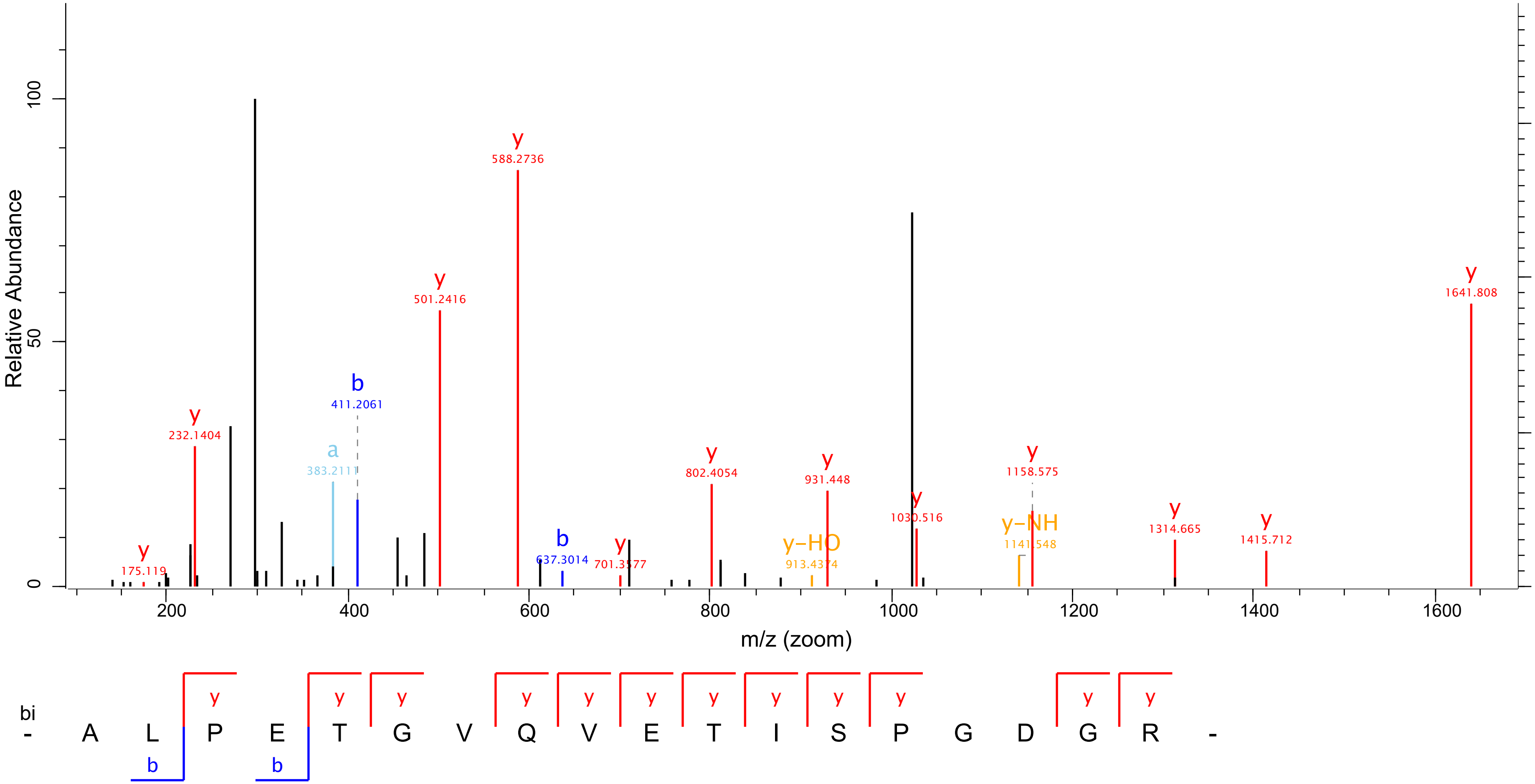

Raw File	Scan	Method	Score	m/z	Gene names
agg_pep_sort_pos_2	15998	FTMS; HCD	70.93	1093.29	FMNL1

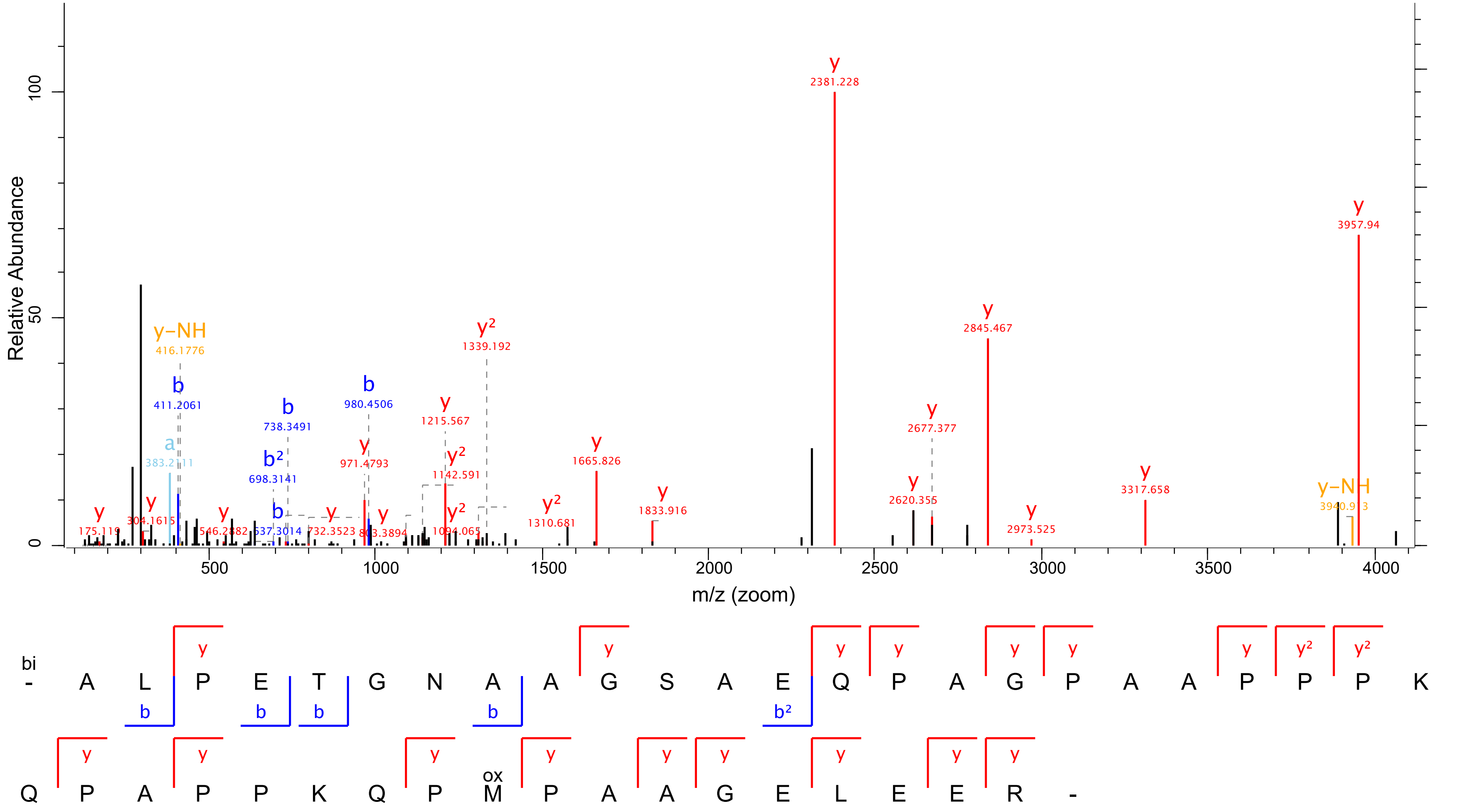

Raw File	Scan	Method	Score	m/z	Gene names
agg_pep_sort_pos_2	16015	FTMS; HCD	41.56	1015.52	FMNL1

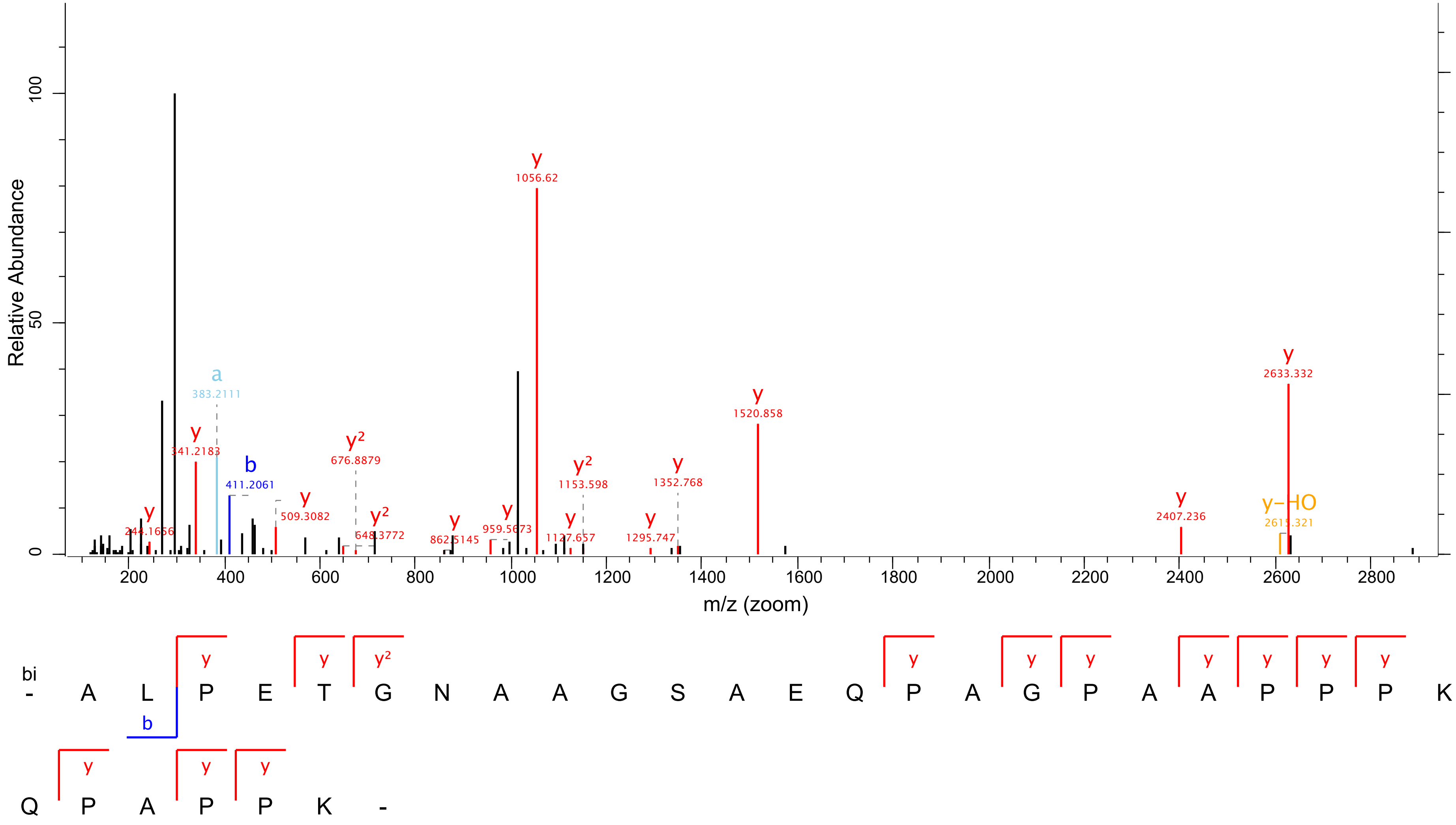

Raw File	Scan	Method	Score	m/z	Gene names
agg_pep_sort_pos_3	17255	FTMS; HCD	82.31	1089.54	FMNL1

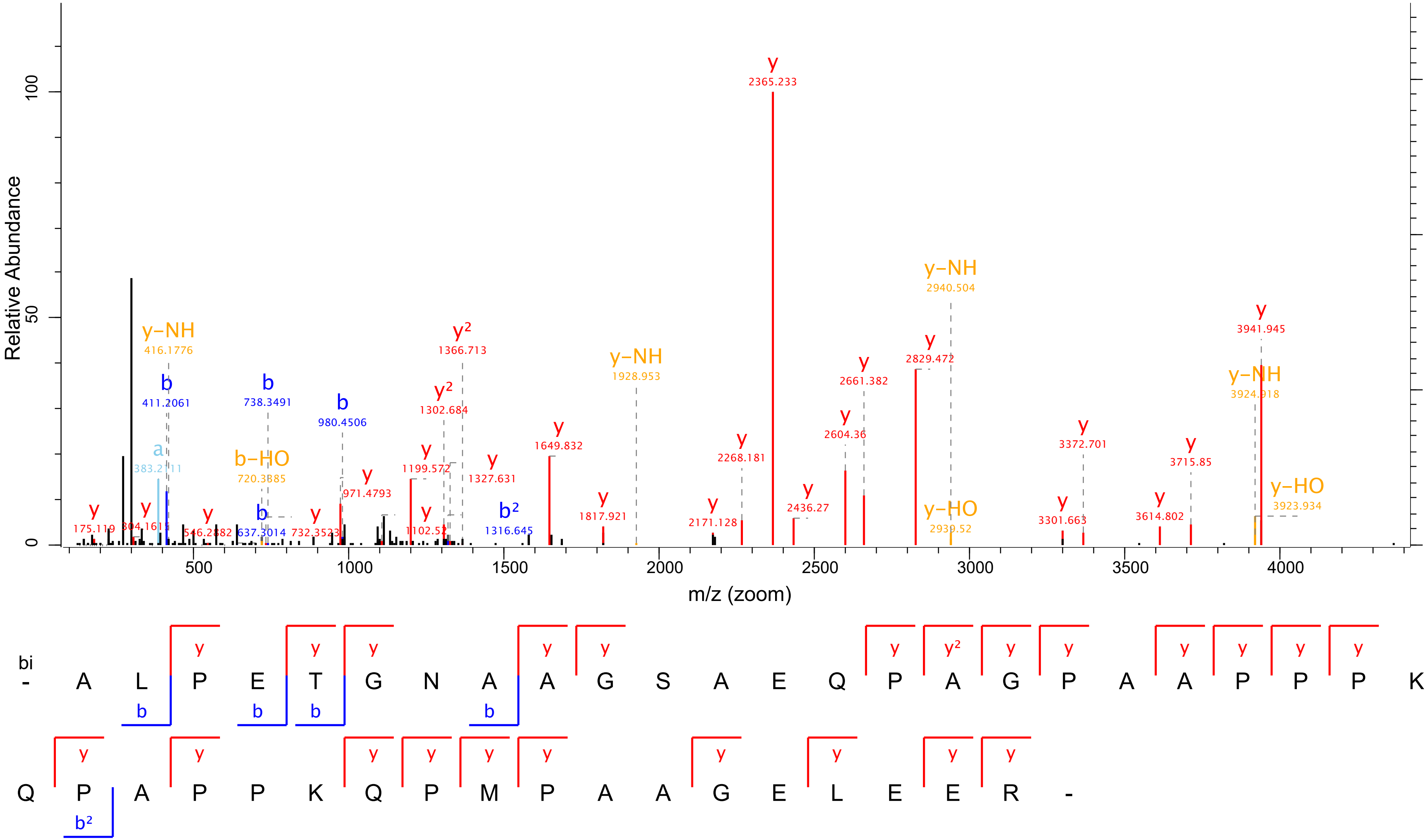

Raw File	Scan	Method	Score	m/z	Gene names
agg_pep_sort_pos_3	18903	FTMS; HCD	64.52	809.4	FMNL1

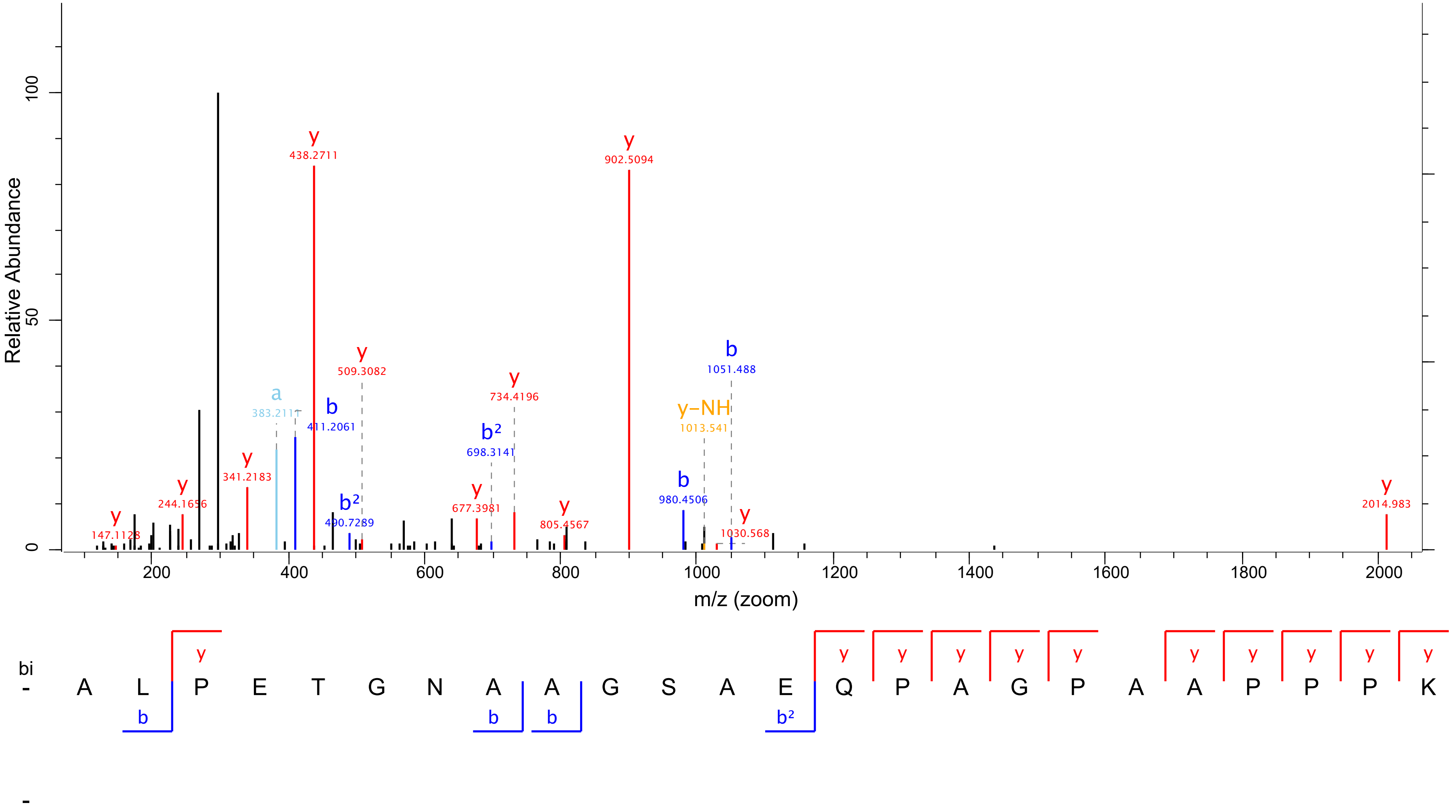

Raw File	Scan	Method	Score	m/z	Gene names
agg_pep_sort_pos_2	19983	FTMS; HCD	40.35	756.66	FMNL2

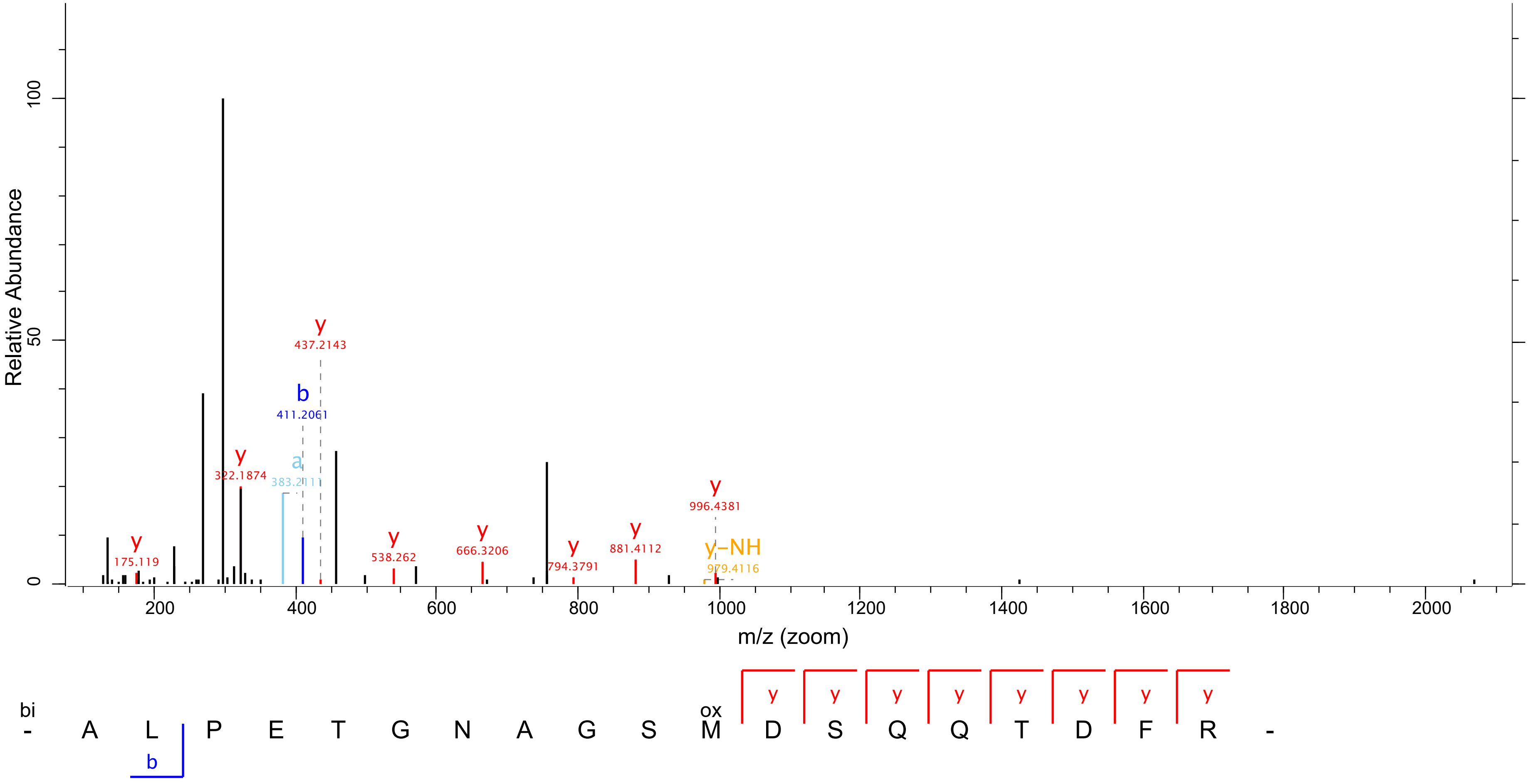

Raw File	Scan	Method	Score	m/z	Gene names
agg_pep_sort_pos_3	21412	FTMS; HCD	71.56	751.33	FMNL2

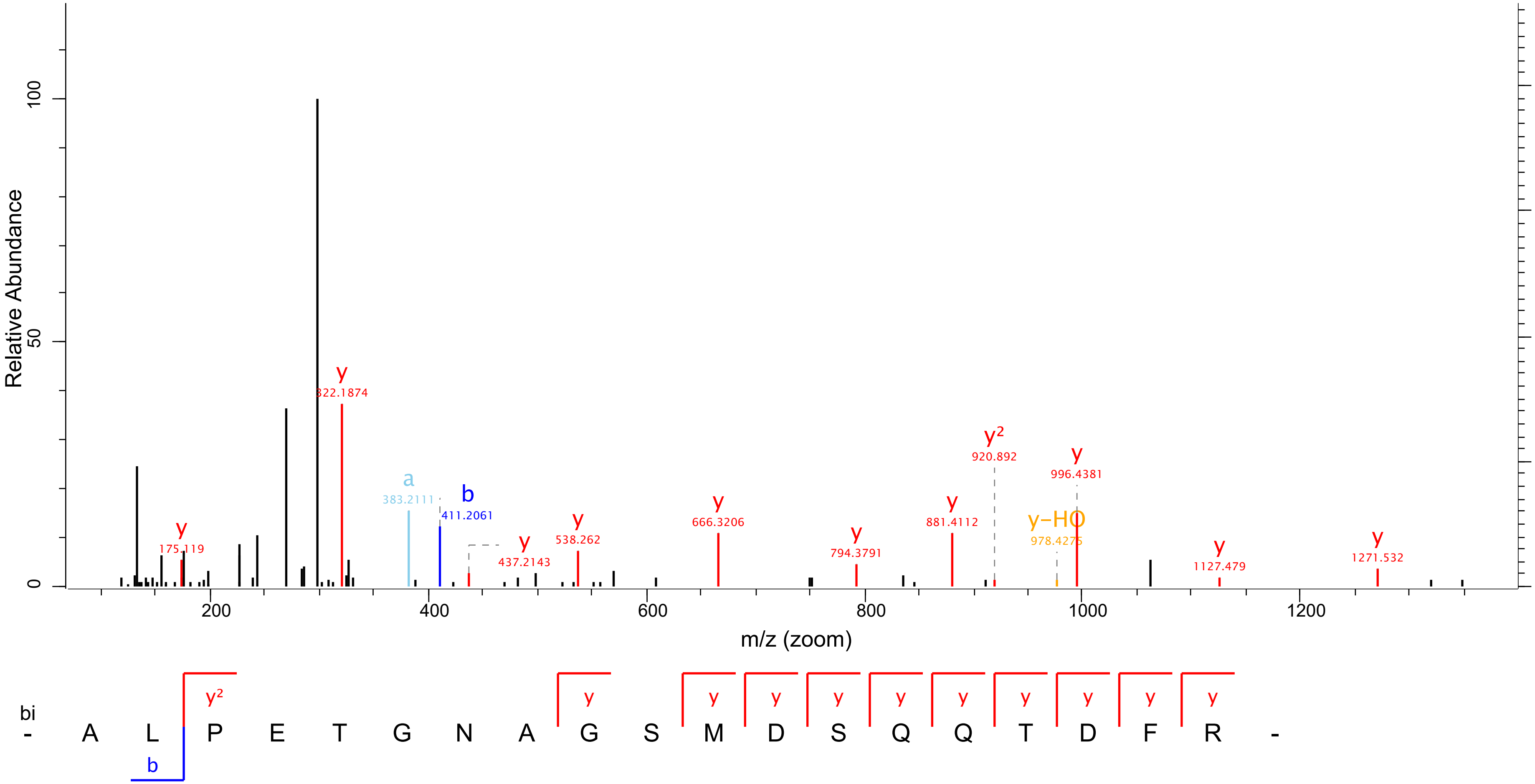

Raw File	Scan	Method	Score	m/z
agg_pep_sort_pos_3	16575	FTMS; HCD	65.56	590.31

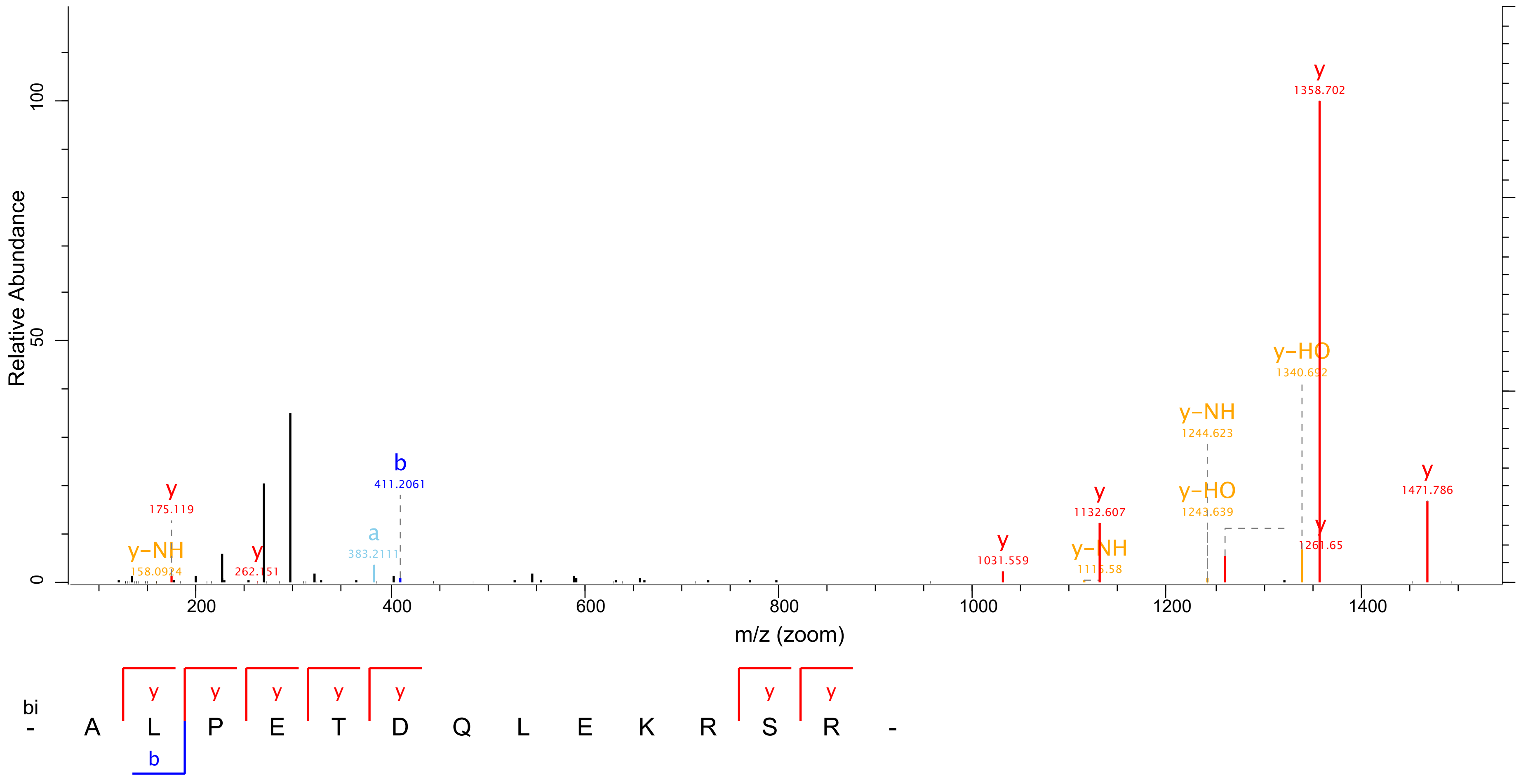

Raw File	Scan	Method	Score	m/z	Gene names
agg_pep_sort_pos_2	12916	FTMS; HCD	116.37	584.82	GAPDH

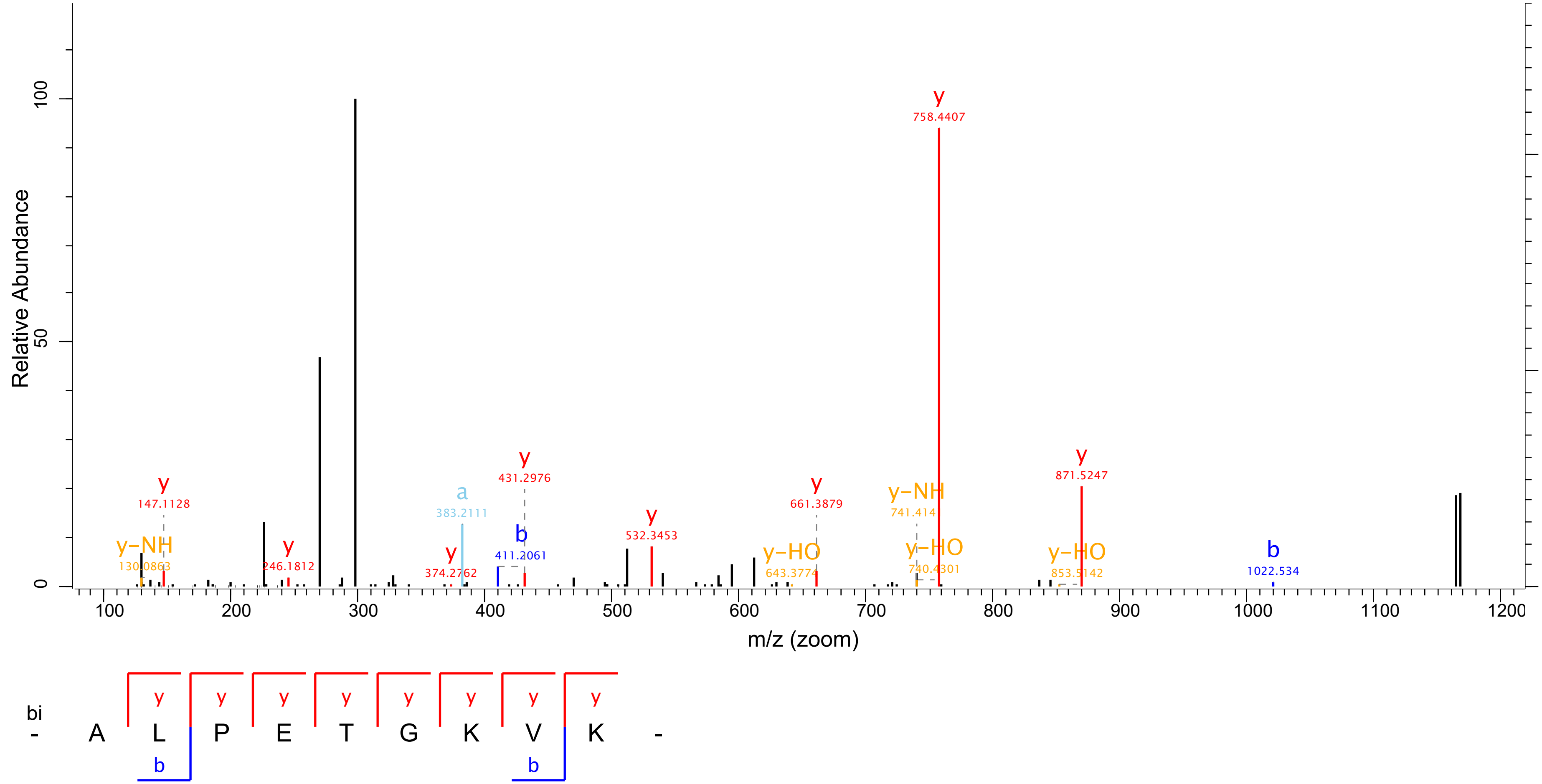

Raw File agg_pep_sort_pos_1 | Scan 17445 | Method FTMS; HCD | Score 97.84 | m/z 652.69 | Gene names GAPDH

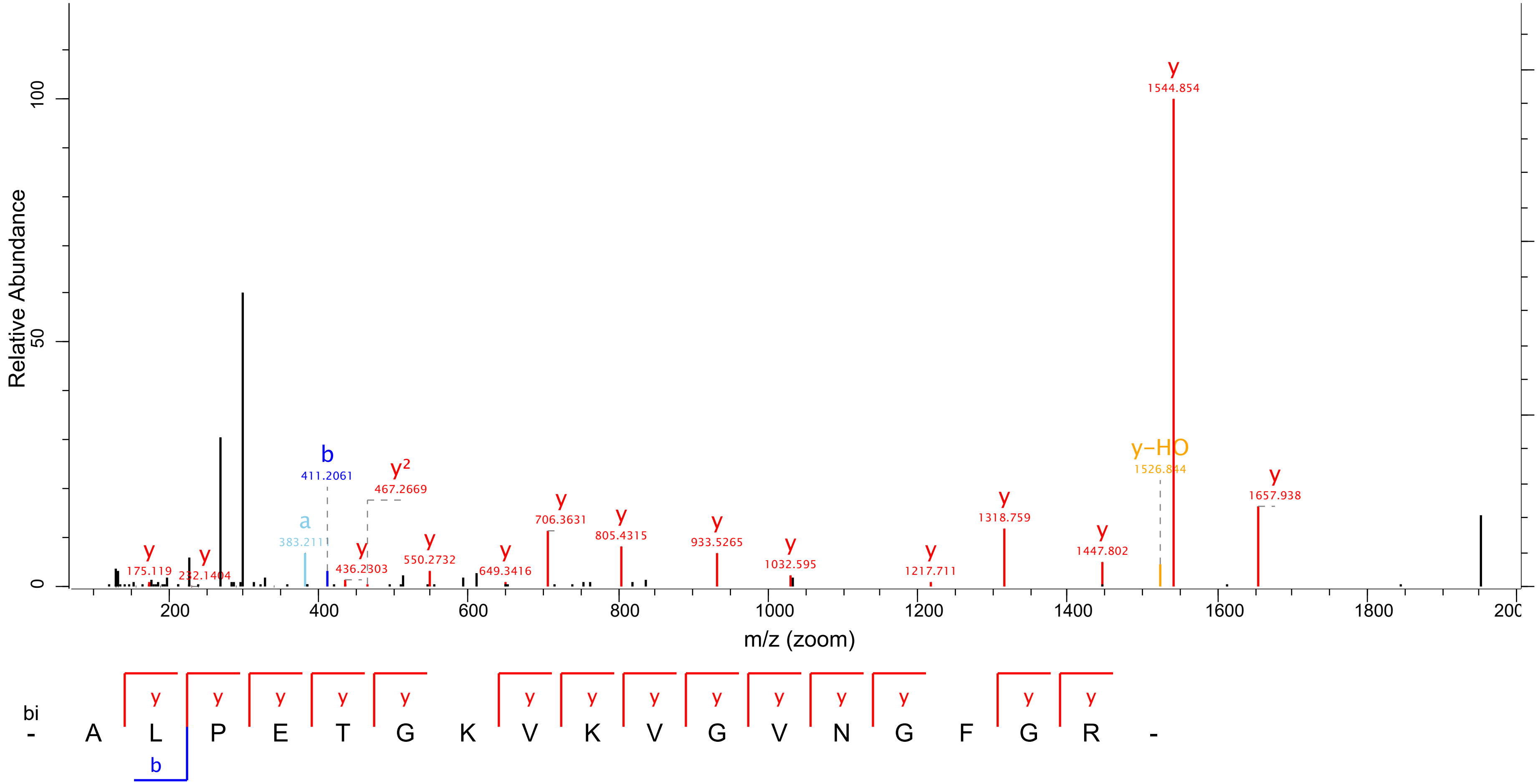

Raw File	Scan	Method	Score	m/z	Gene names
agg_pep_sort_pos_3	25685	FTMS; HCD	172.84	842.76	GCLC

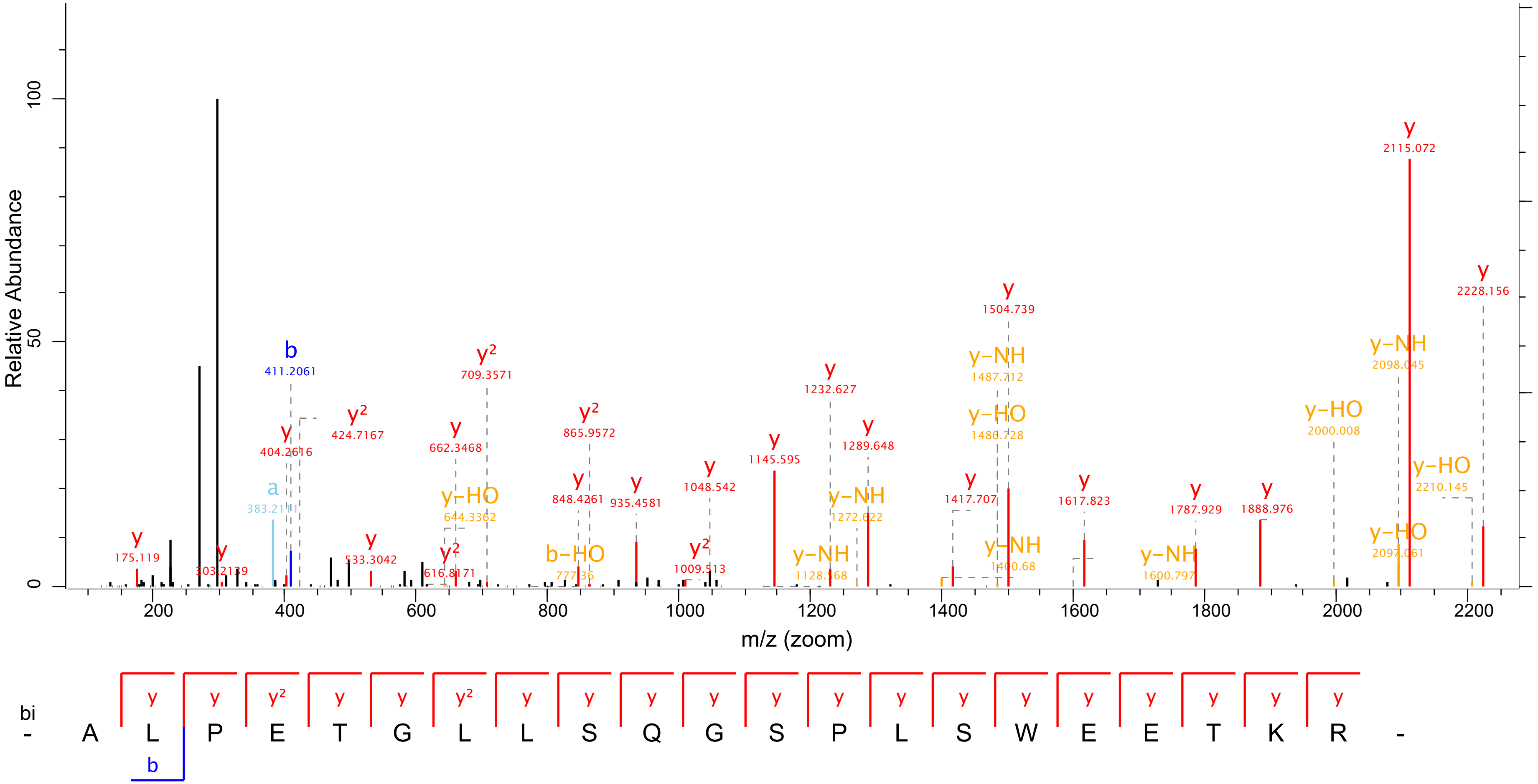

Raw File	Scan	Method	Score	m/z	Gene names
agg_pep_sort_pos_3	18017	FTMS; HCD	104.61	749.03	GNAI1;GNAI3

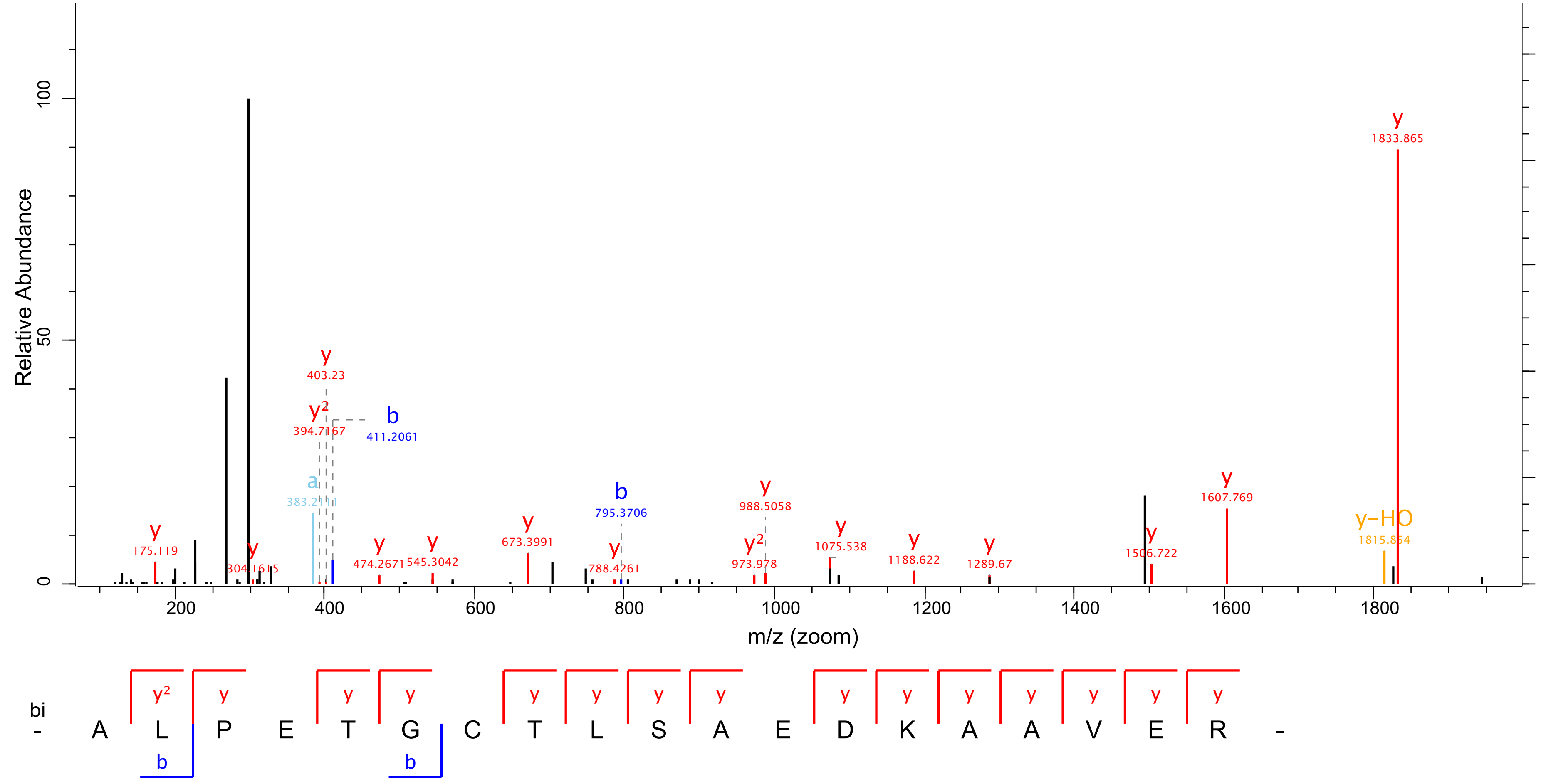

Raw File	Scan	Method	Score	m/z	Gene names
agg_pep_sort_pos_2	20403	FTMS; HCD	49.5	859.89	GNAI1;GNAI3

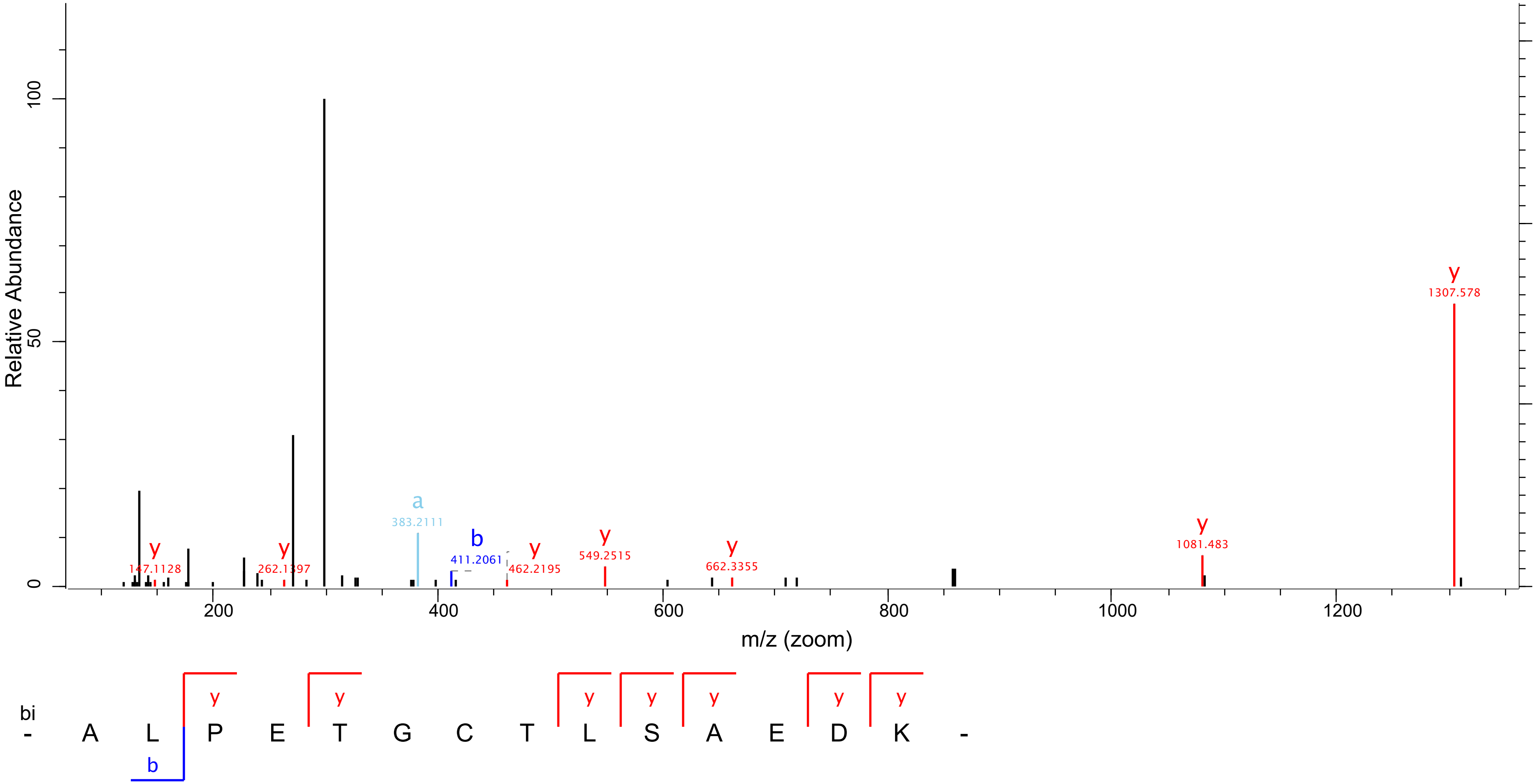

Raw File	Scan	Method	Score	m/z	Gene names
agg_pep_sort_pos_3	16044	FTMS; HCD	141.86	735.01	GNAI2

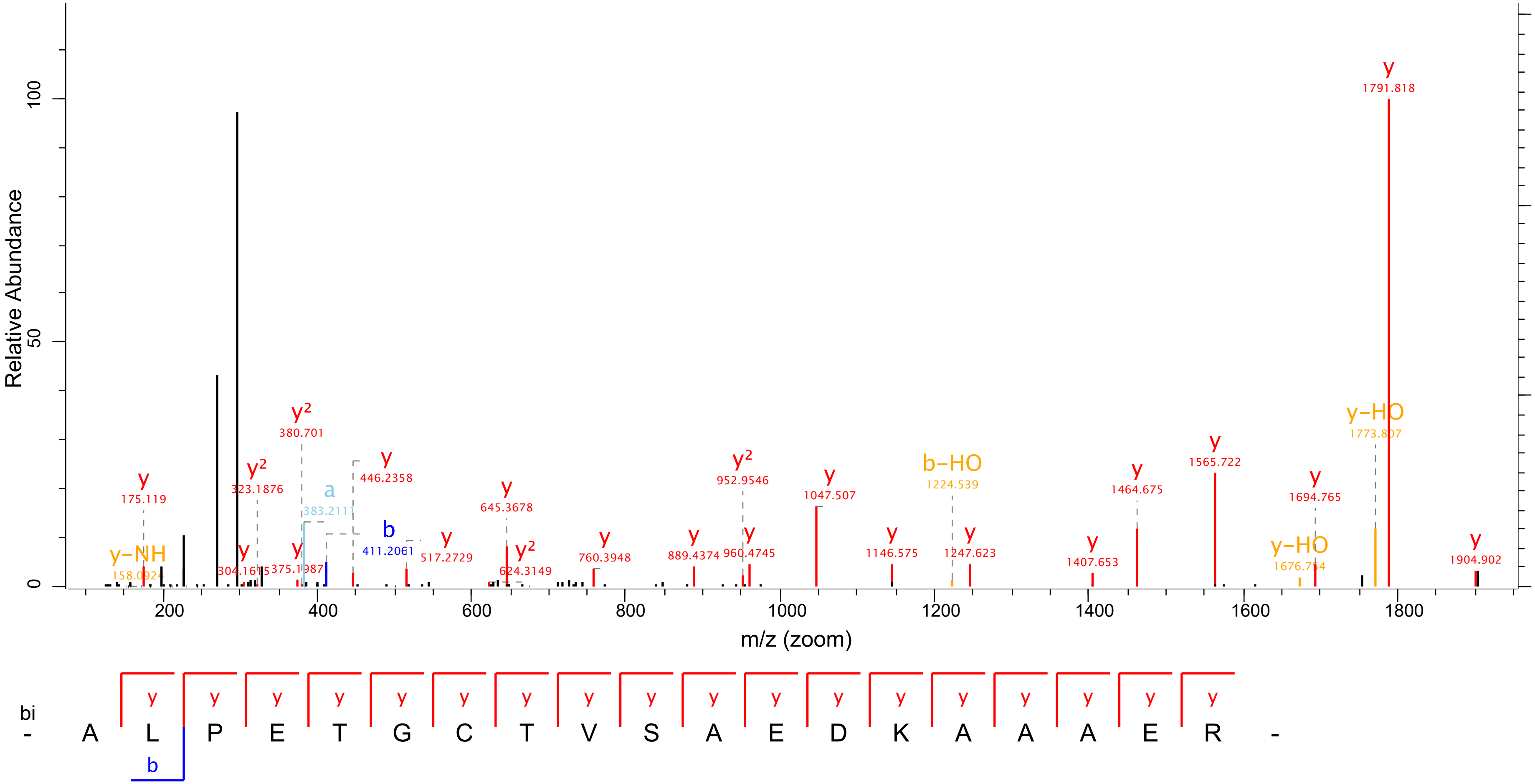

Raw File	Scan	Method	Score	m/z	Gene names
agg_pep_sort_pos_1	19277	FTMS; HCD	85.29	852.38	GNAI2

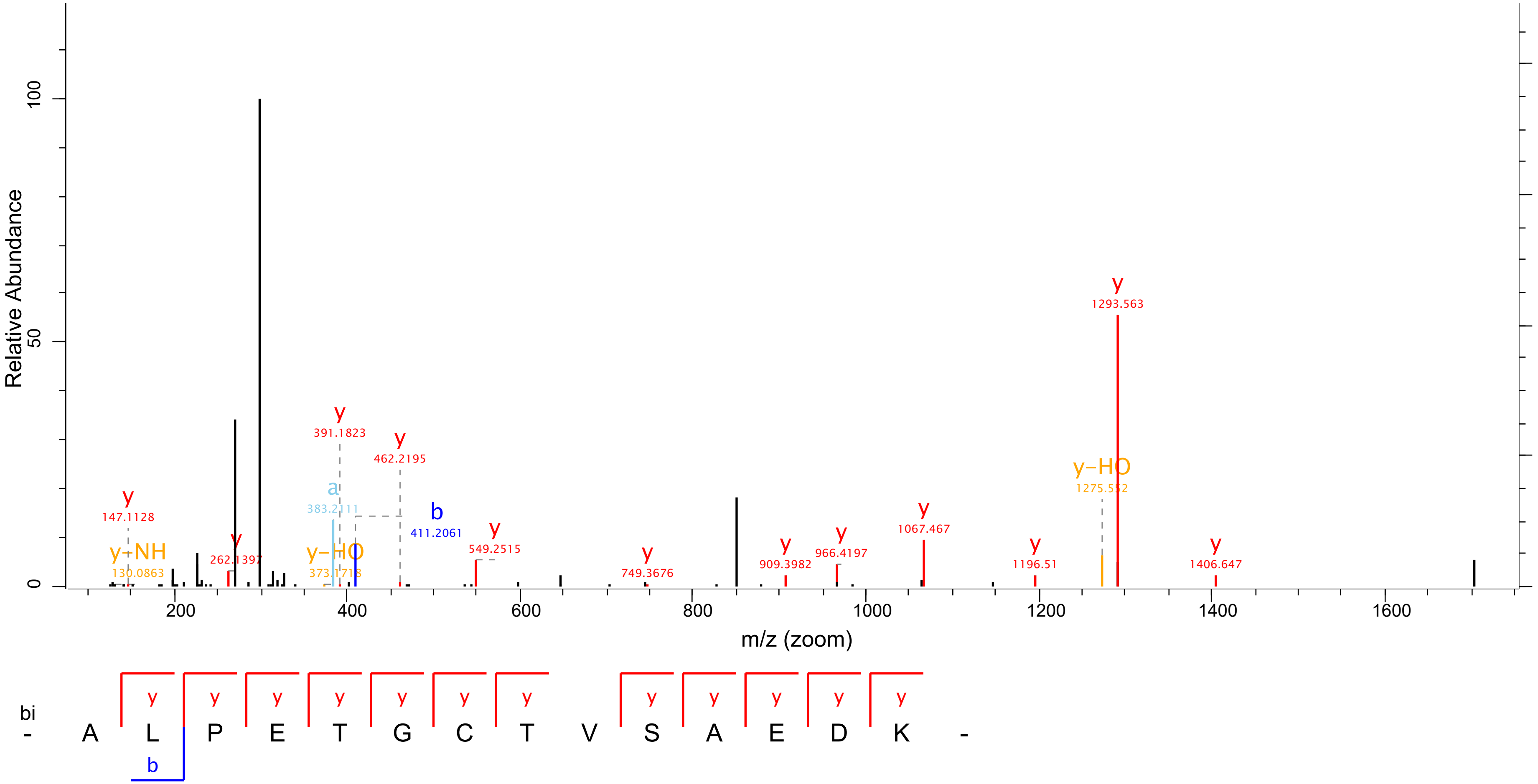

Raw File	Scan	Method	Score	m/z	Gene names
agg_pep_sort_pos_2	20634	FTMS; HCD	113.63	880.9	GNAO1

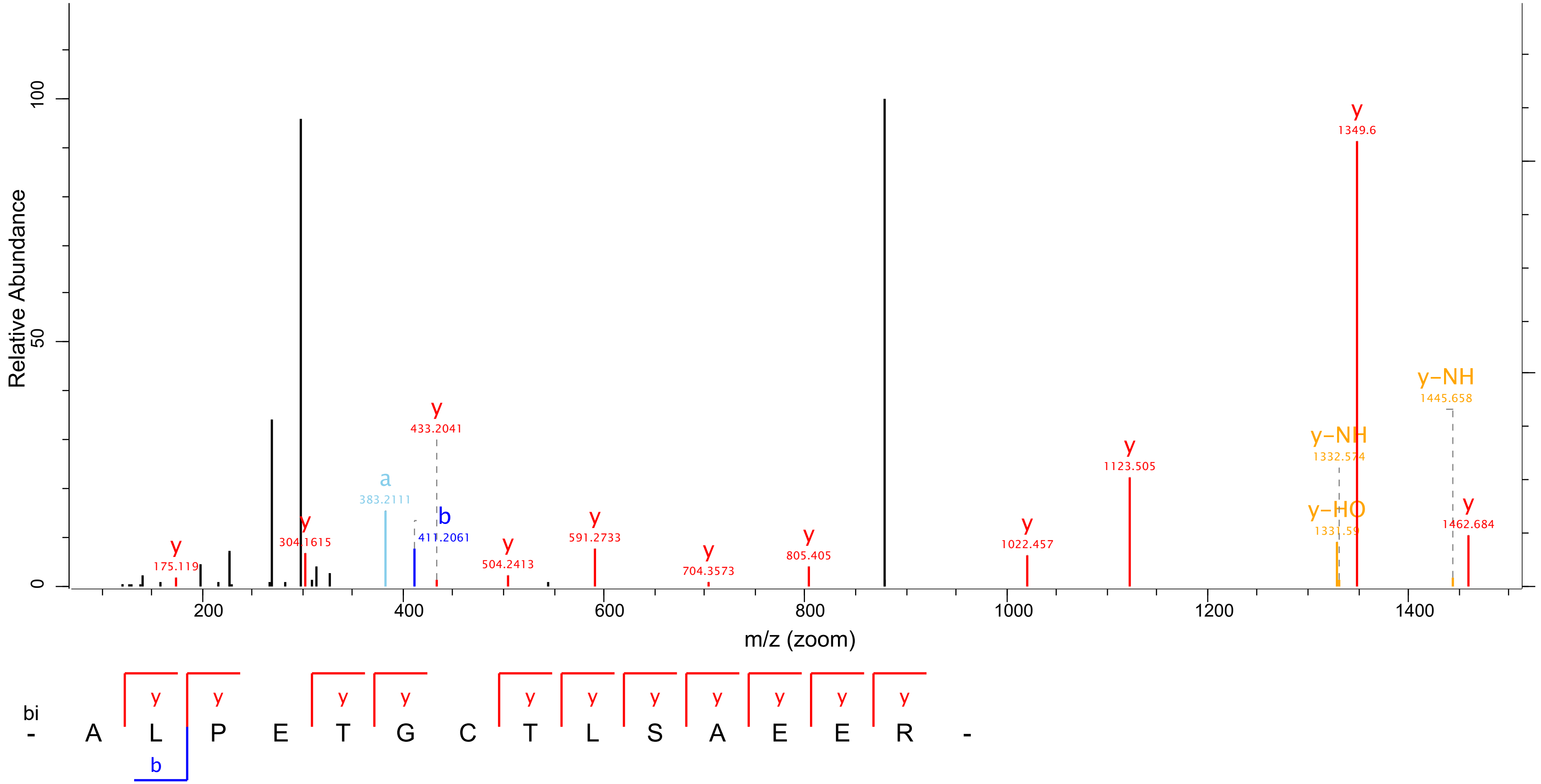

Raw File	Scan	Method	Score	m/z	Gene names
agg_pep_sort_pos_1	17320	FTMS; HCD	42.32	749.11	GNAQ

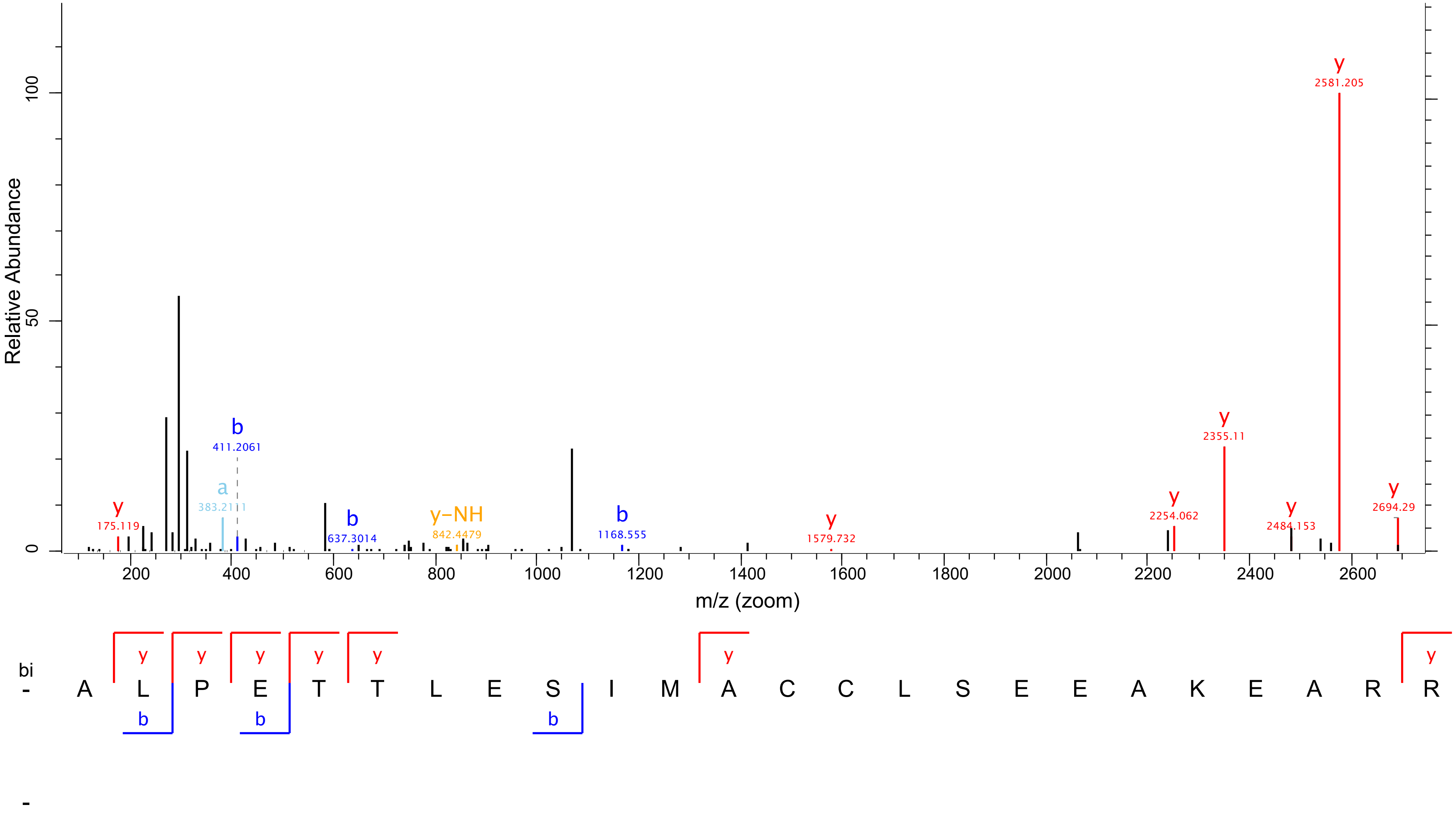

Raw File	Scan	Method	Score	m/z	Gene names
agg_pep_sort_pos_1	13691	FTMS; HCD	118.52	868.4	GNAS

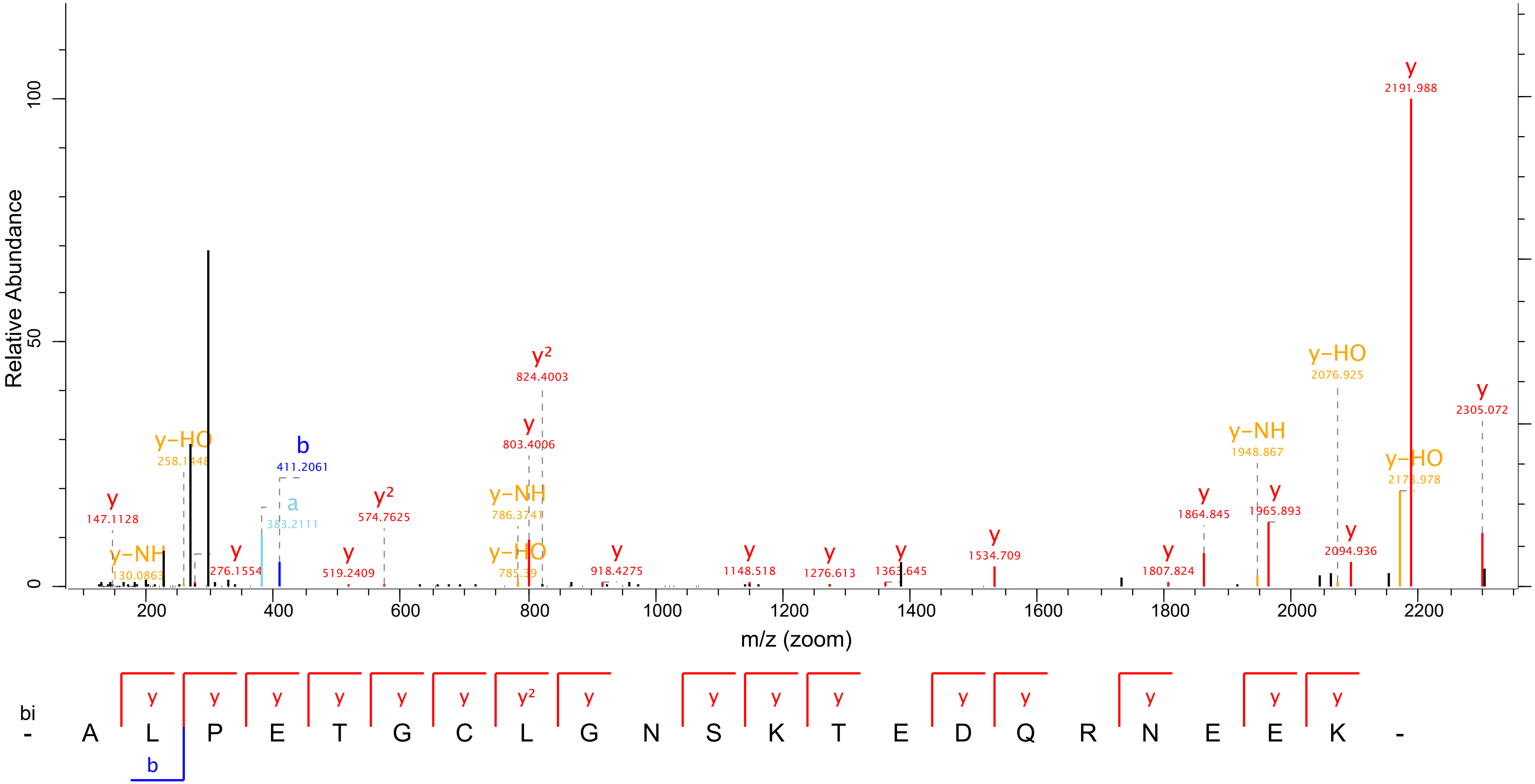

Raw File	Scan	Method	Score	m/z	Gene names
agg_pep_sort_pos_3	16056	FTMS; HCD	98.51	701.33	GNAS

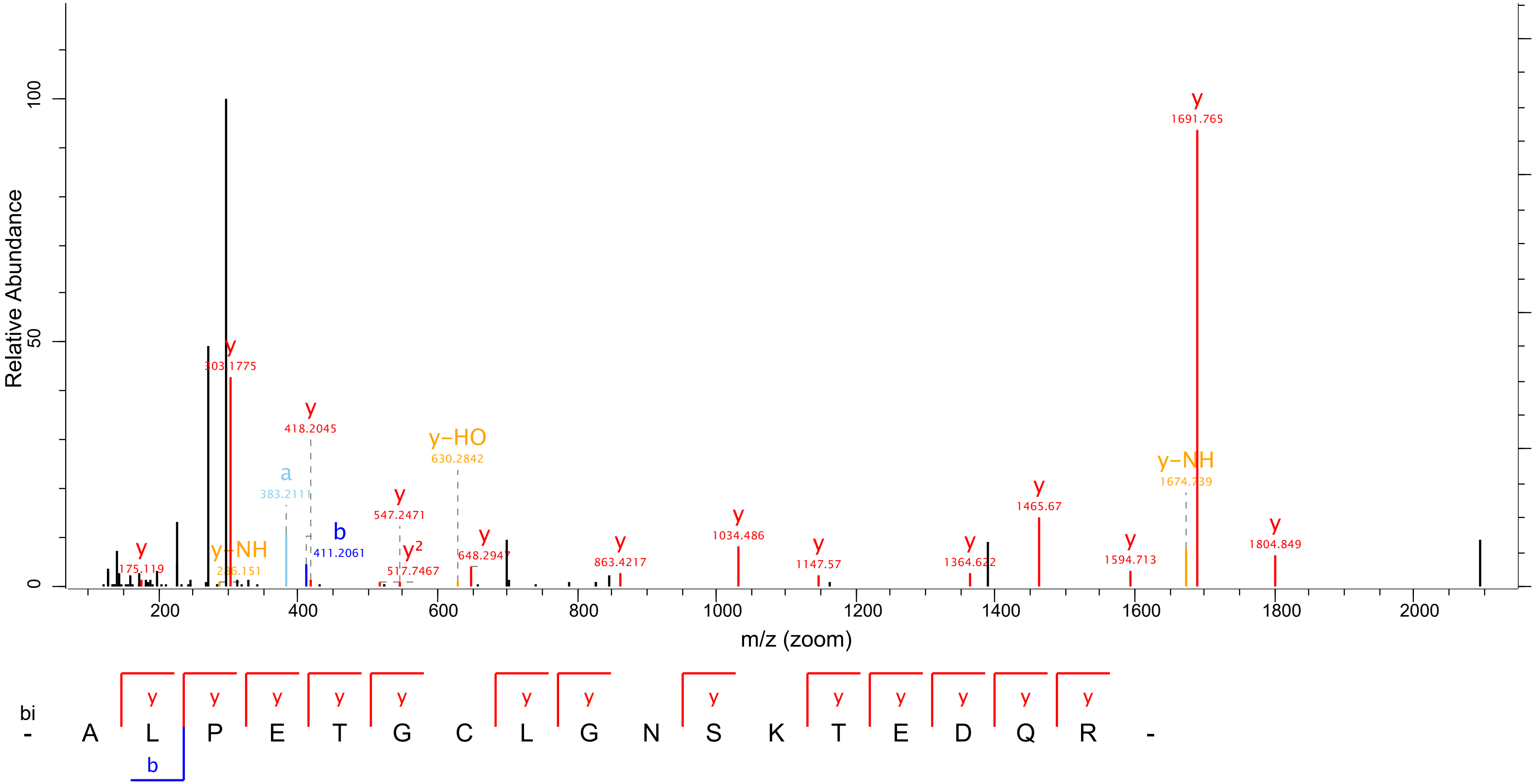

Raw File	Scan	Method	Score	m/z	Gene names
agg_pep_sort_pos_3	19653	FTMS; HCD	88.06	736.85	GNAS

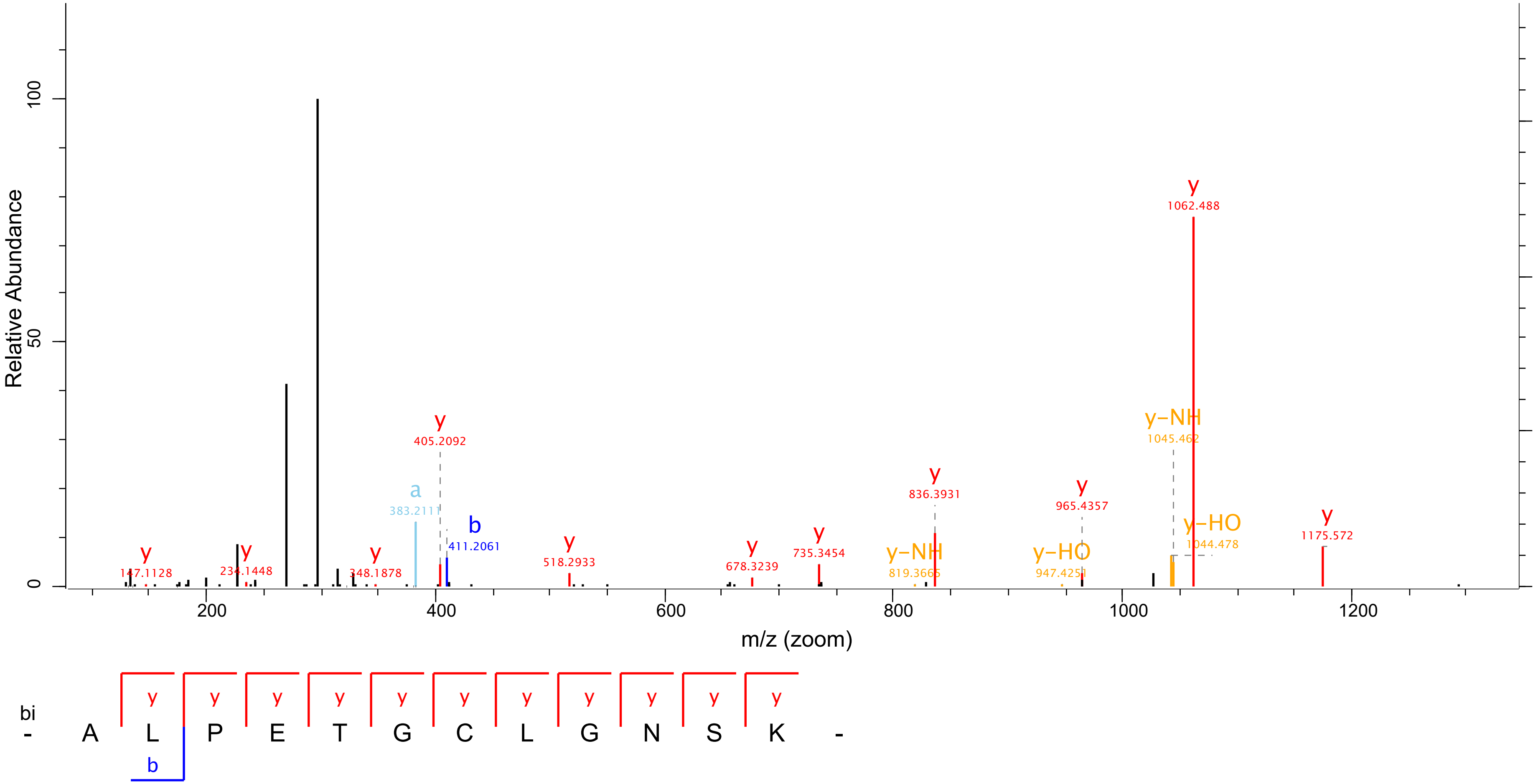

Raw File	Scan	Method	Score	m/z	Gene names
agg_pep_sort_pos_3	21150	FTMS; HCD	86.79	923.45	GORASP2

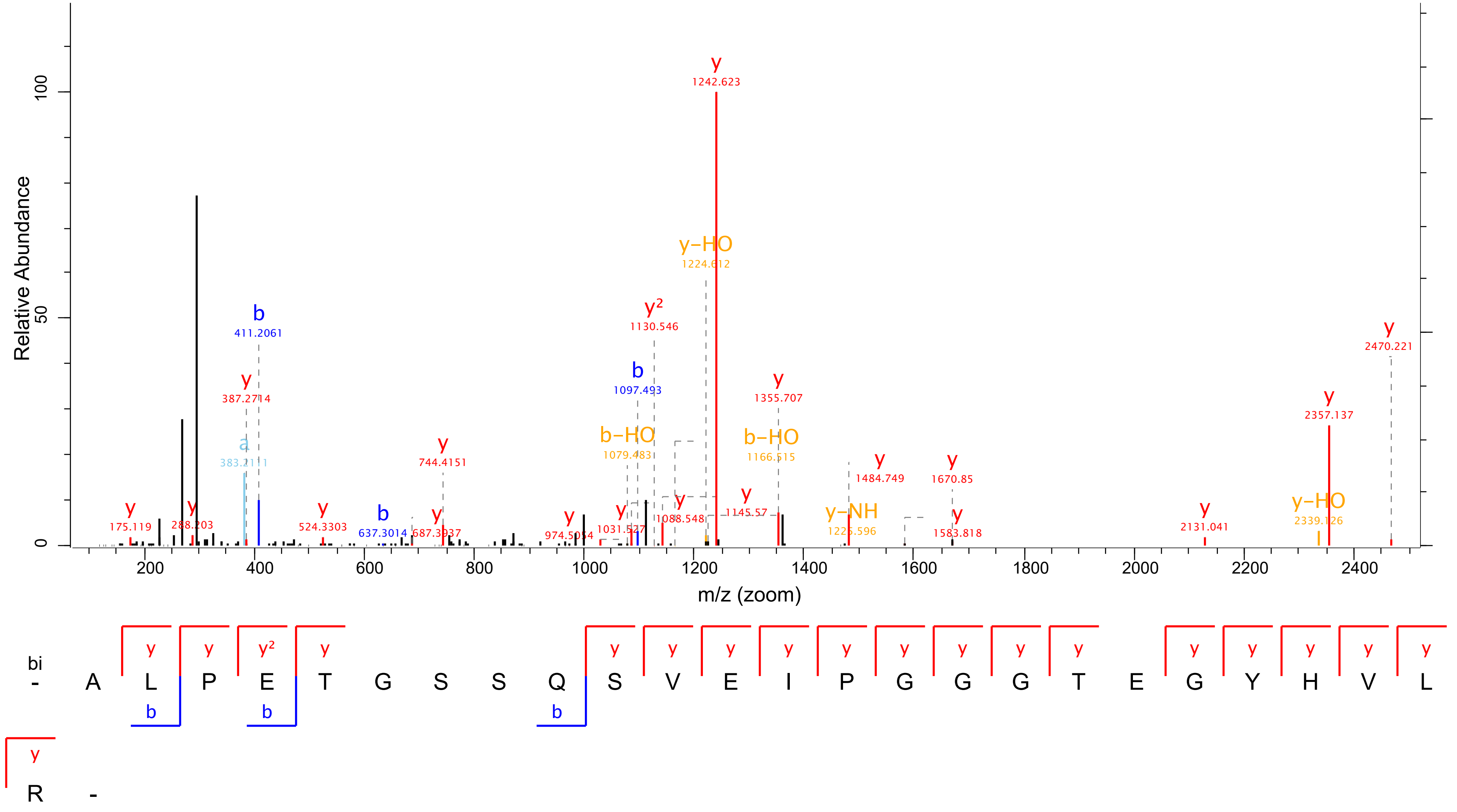

Raw File	Scan	Method	Score	m/z	Gene names
agg_pep_sort_pos_1	17822	FTMS; HCD	82.54	626.32	GPRC5B

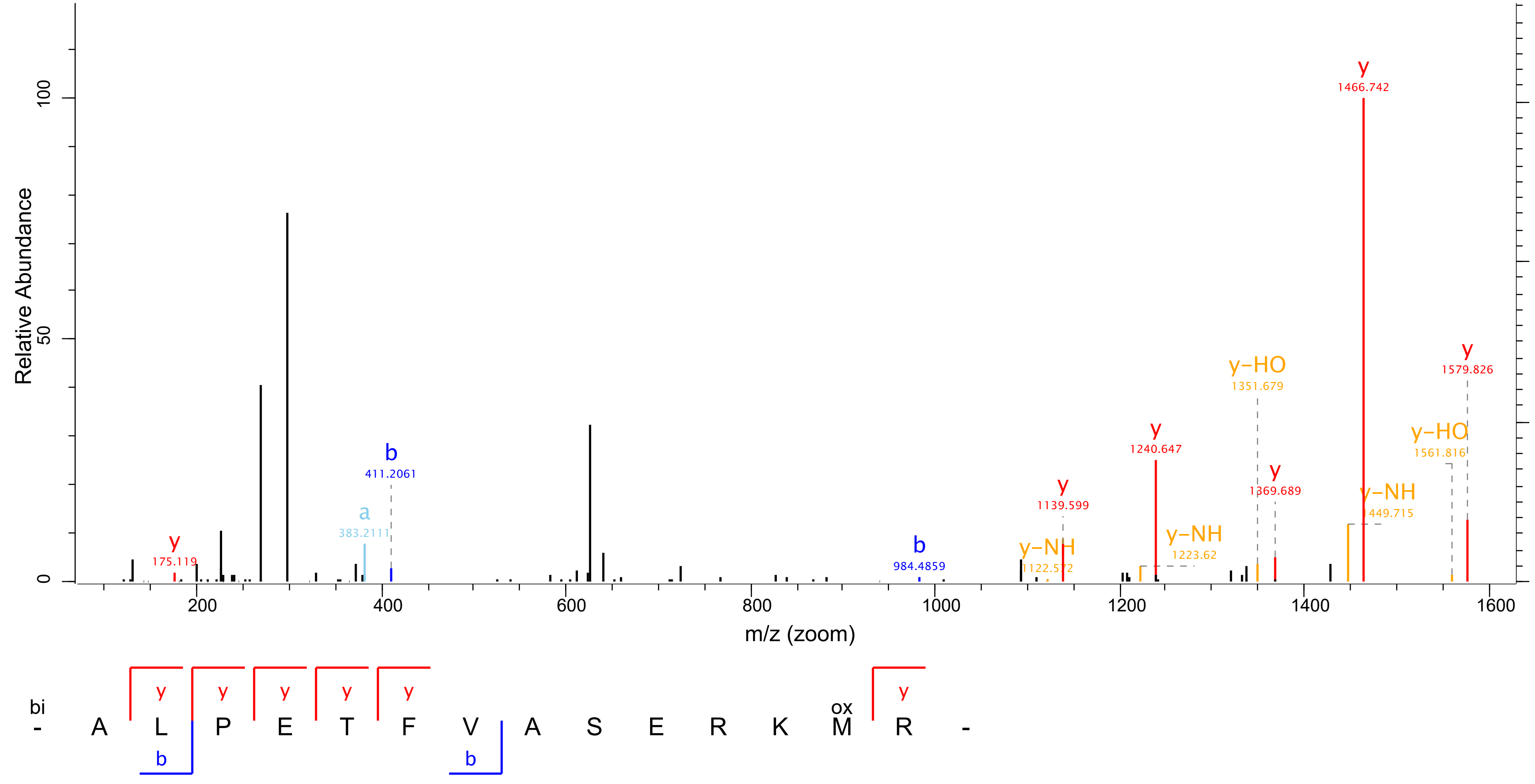

Raw File	Scan	Method	Score	m/z	Gene names
agg_pep_sort_pos_1	17337	FTMS; HCD	83.21	571.29	GSTZ1

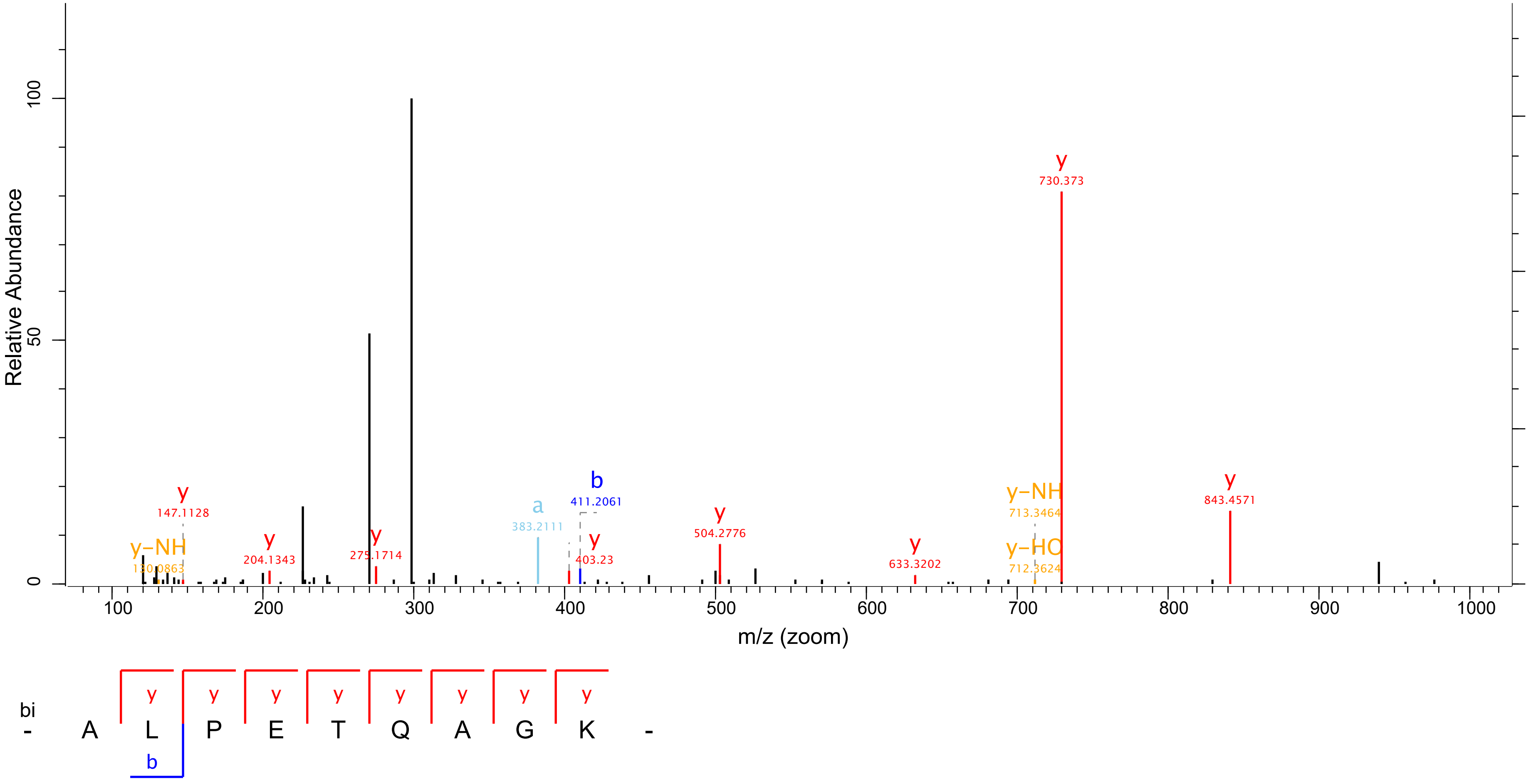

Raw File	Scan	Method	Score	m/z	Gene names
agg_pep_sort_pos_1	18957	FTMS; HCD	44.81	686.37	HACD4

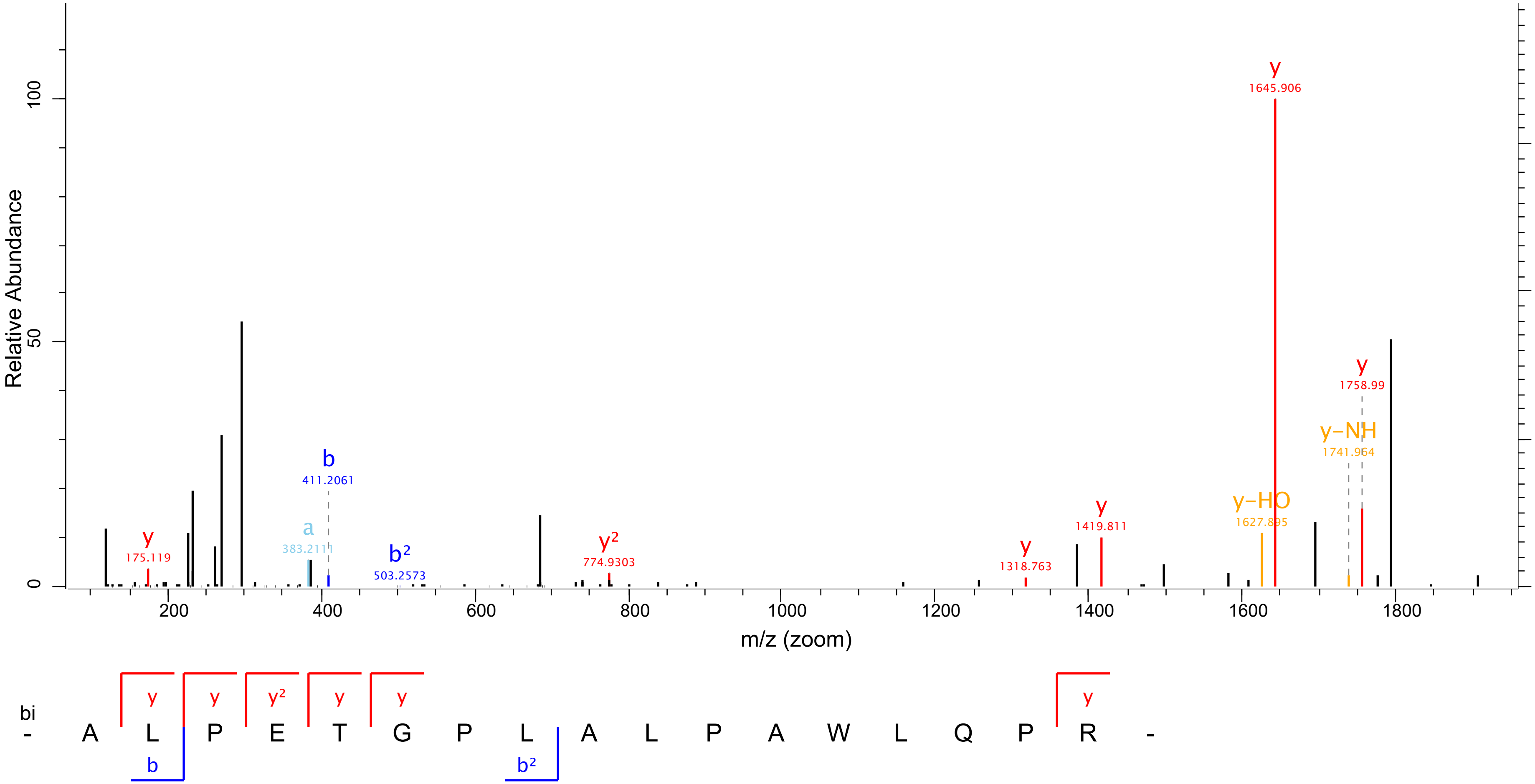

Raw File	Scan	Method	Score	m/z	Gene names
agg_pep_sort_pos_1	21429	FTMS; HCD	65.78	912.77	HGH1

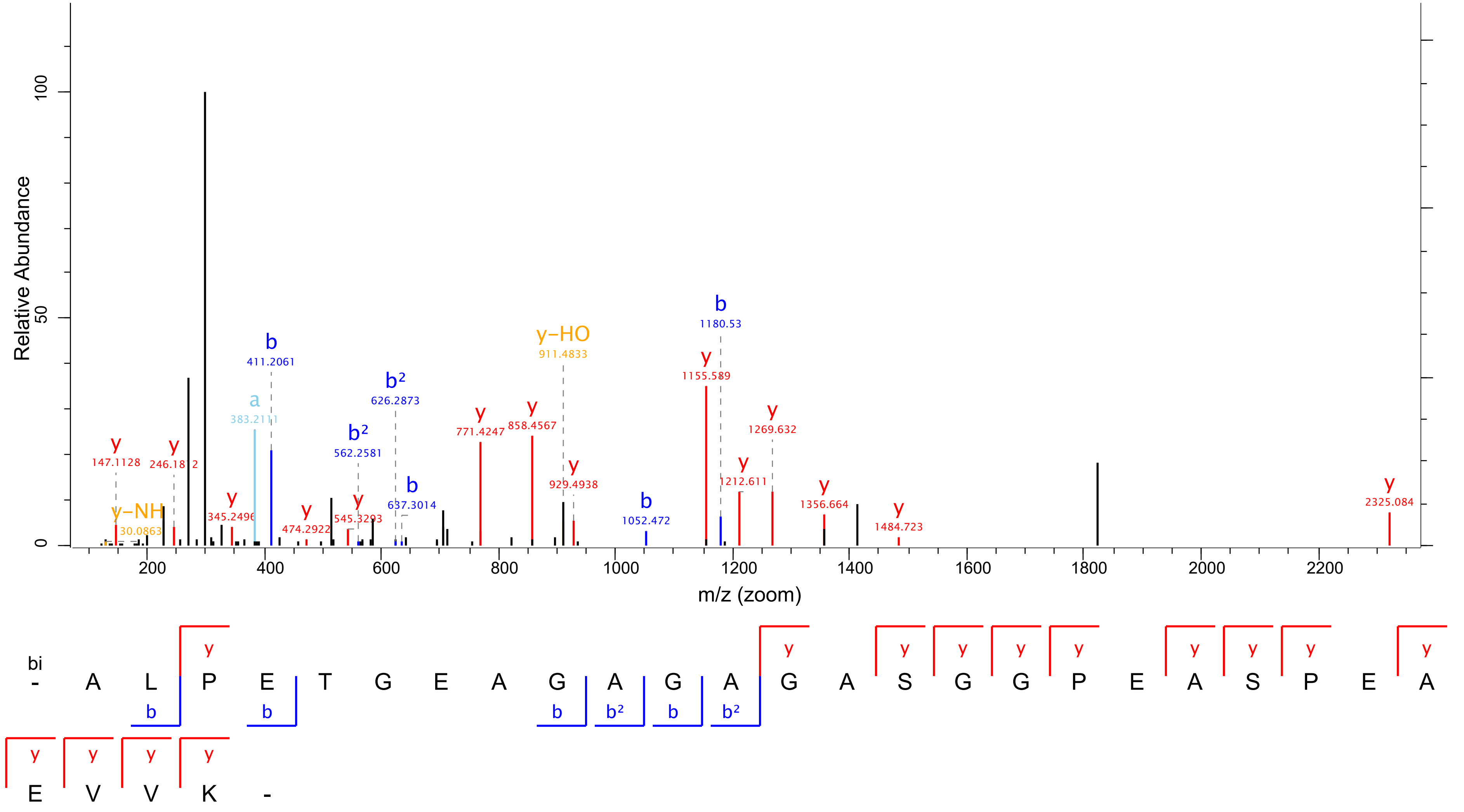

Raw File	Scan	Method	Score	m/z	Gene names
agg_pep_sort_pos_2	14609	FTMS; HCD	55.4	915.98	HIST2H2BE;HIST1H2BC;H2BFS;HIST1H2BJ;HIST1H2BK

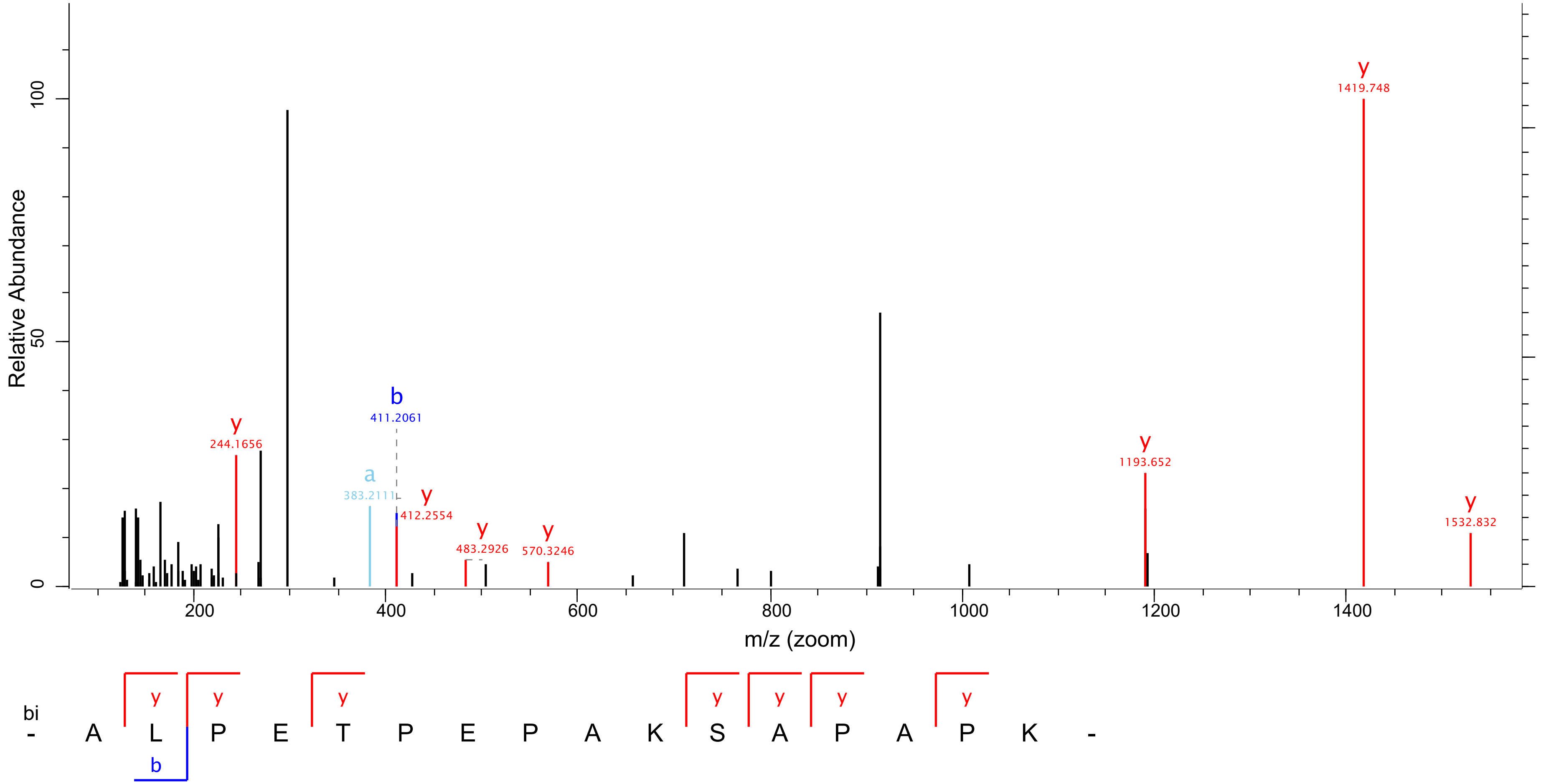

Raw File	Scan	Method	Score	m/z	Gene names
agg_pep_sort_pos_2	12260	FTMS; HCD	99.82	669.84	HMGB1;HMGB1P1

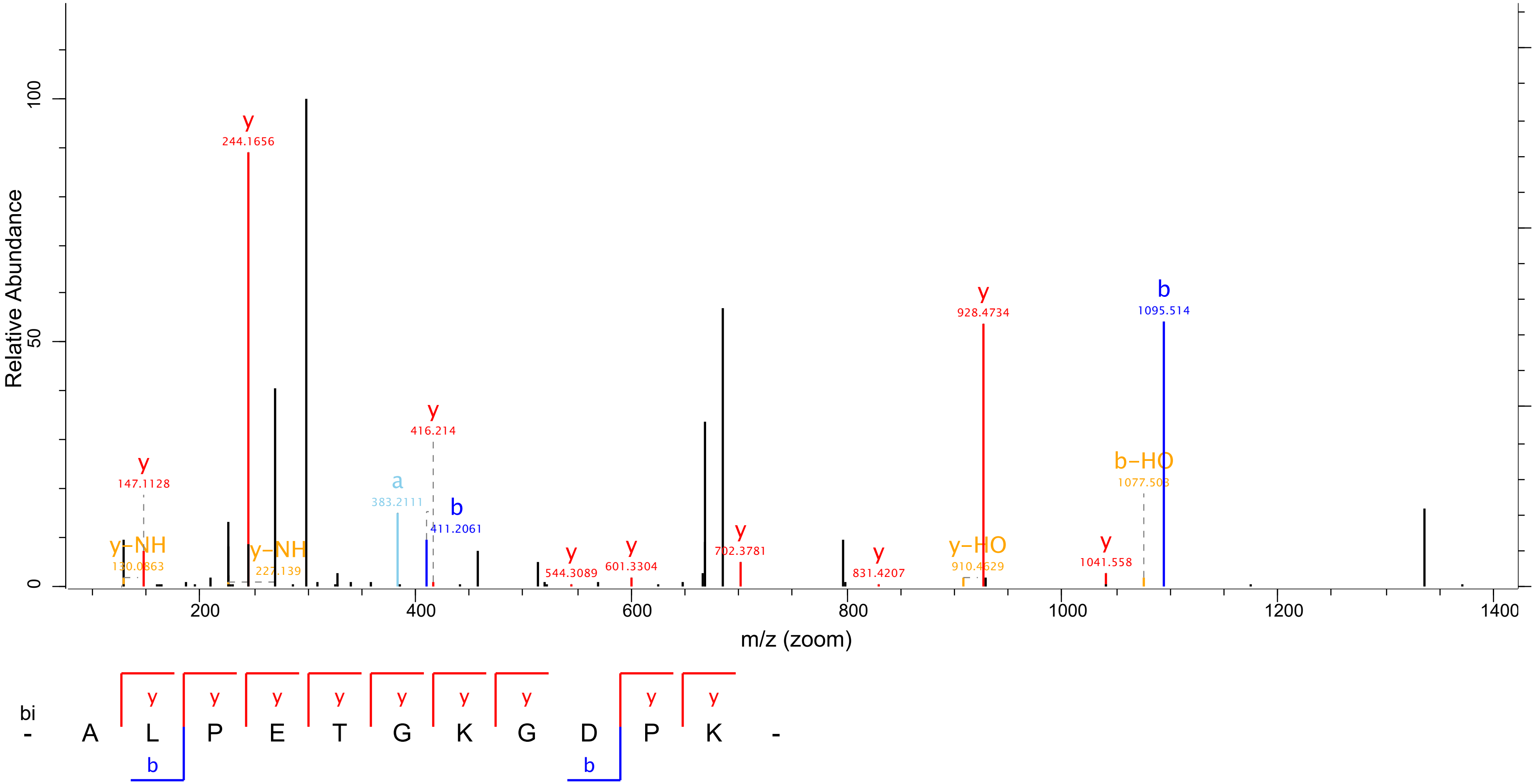

Raw File	Scan	Method	Score	m/z	Gene names
agg_pep_sort_pos_1	10291	FTMS; HCD	143.98	569.3	HMGB2

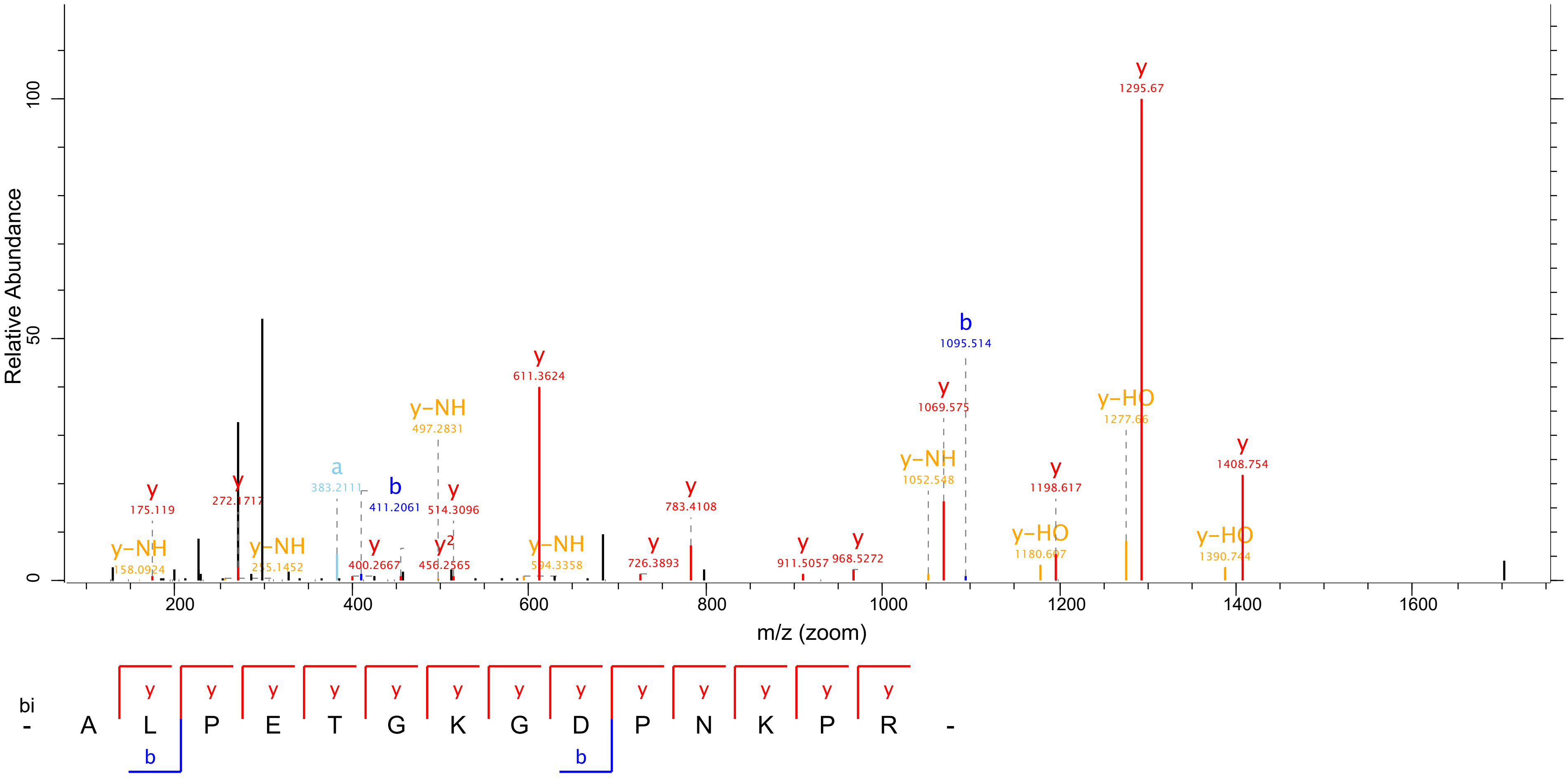

Raw File	Scan	Method	Score	m/z	Gene names
agg_pep_sort_pos_3	13735	FTMS; HCD	84.21	697.37	IRF3

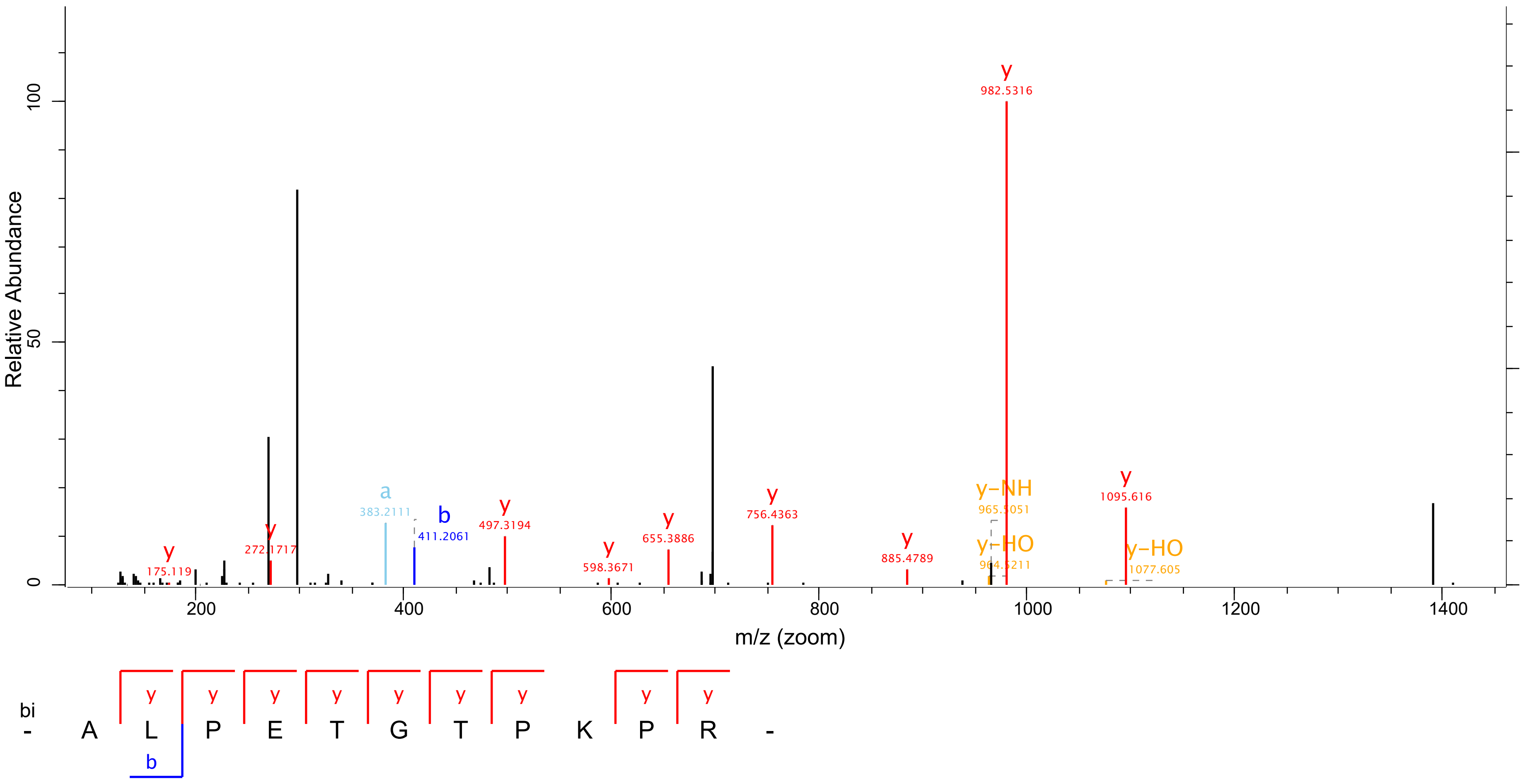

Raw File	Scan	Method	Score	m/z
agg_pep_sort_pos_3	13392	FTMS; HCD	66.38	724.1

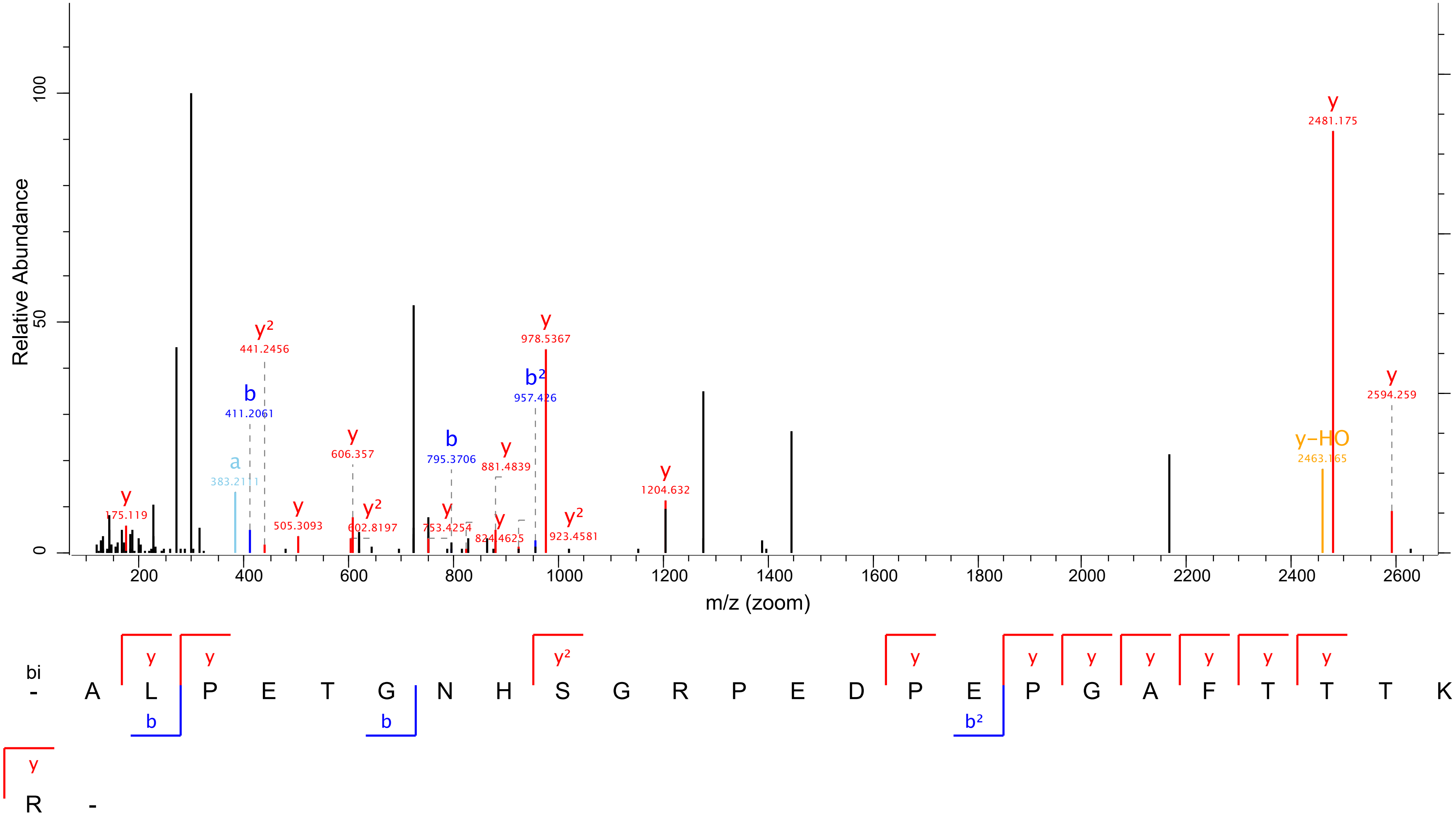

Raw File	Scan	Method	Score	m/z	Gene names
agg_pep_sort_pos_1	20266	FTMS; HCD	92.06	705.86	KIF7

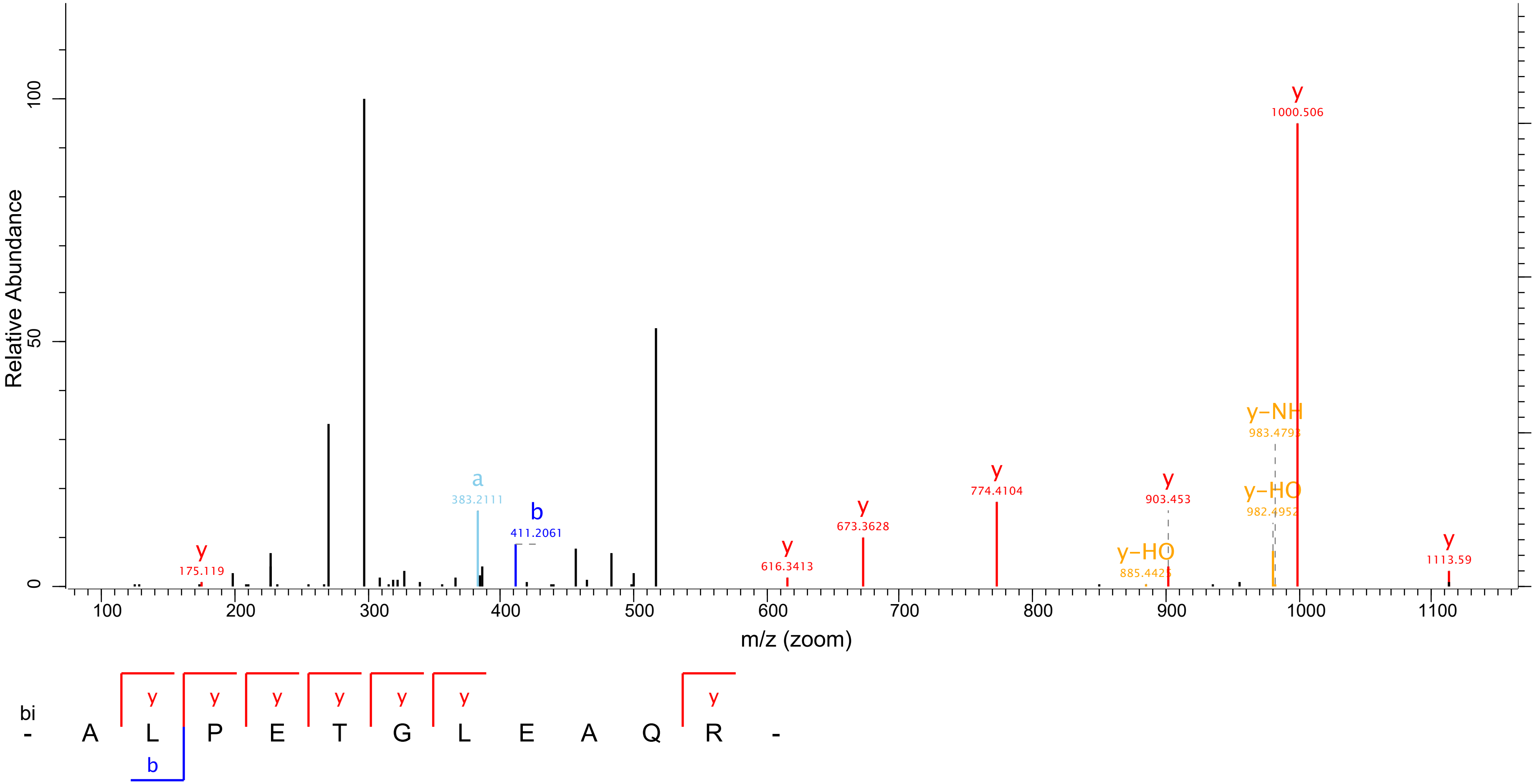

Raw File	Scan	Method	Score	m/z	Gene names
agg_pep_sort_pos_2	20986	FTMS; HCD	78.67	811.09	KIF7

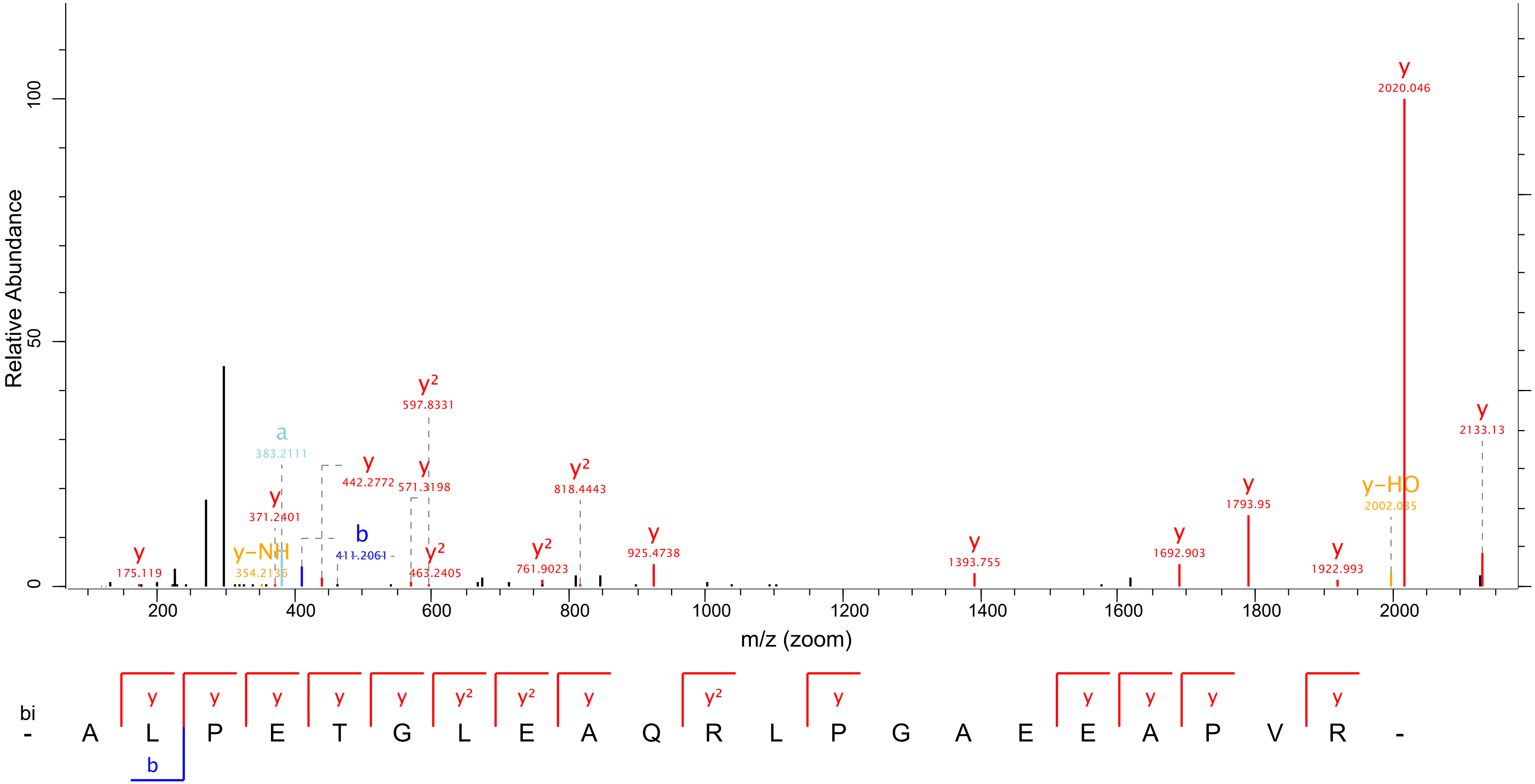

Raw File	Scan	Method	Score	m/z	Gene names
agg_pep_sort_pos_2	19811	FTMS; HCD	117.09	865.44	KIN

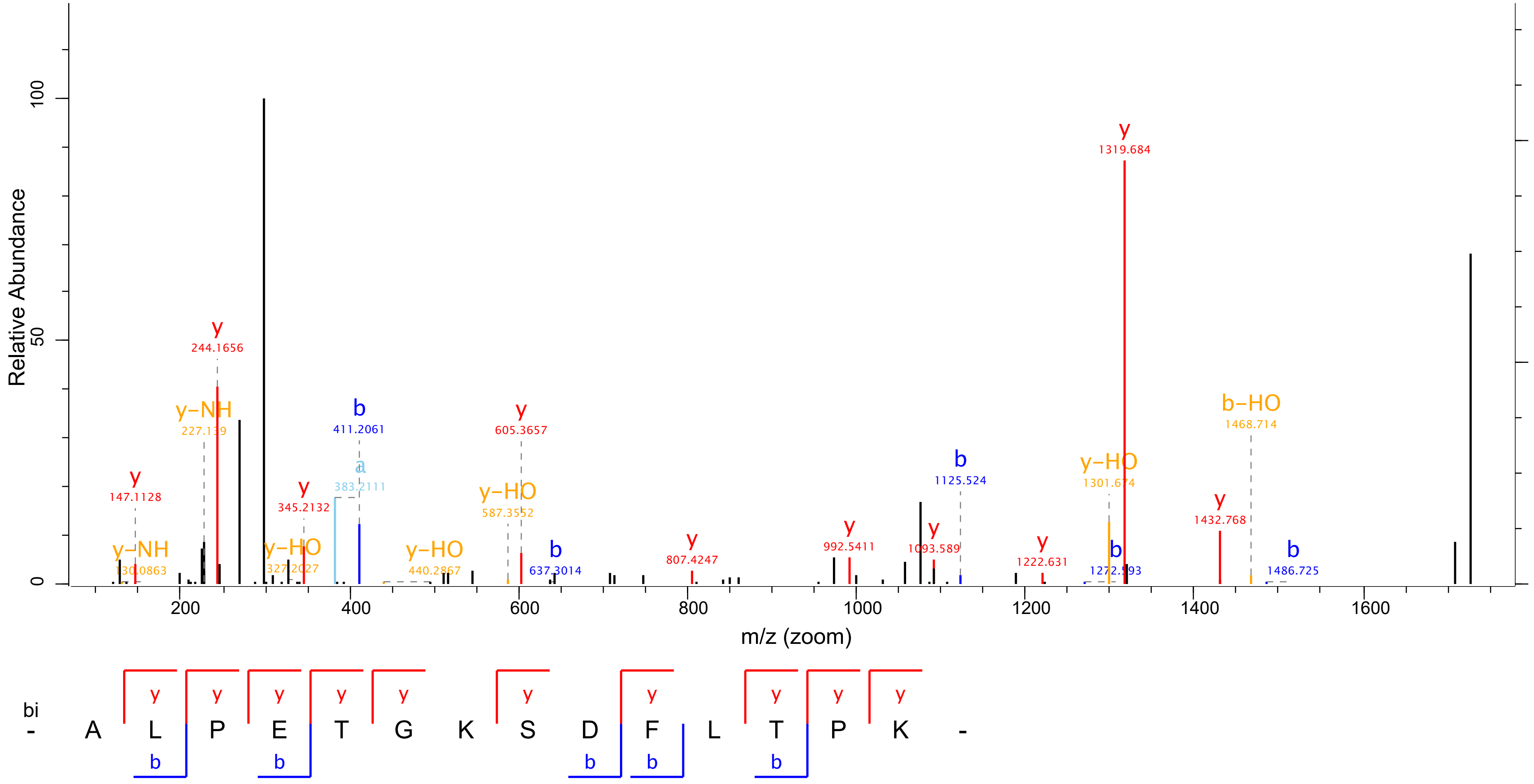

Raw File	Scan	Method	Score	m/z
agg_pep_sort_pos_3	23228	FTMS; HCD	61.35	829.91

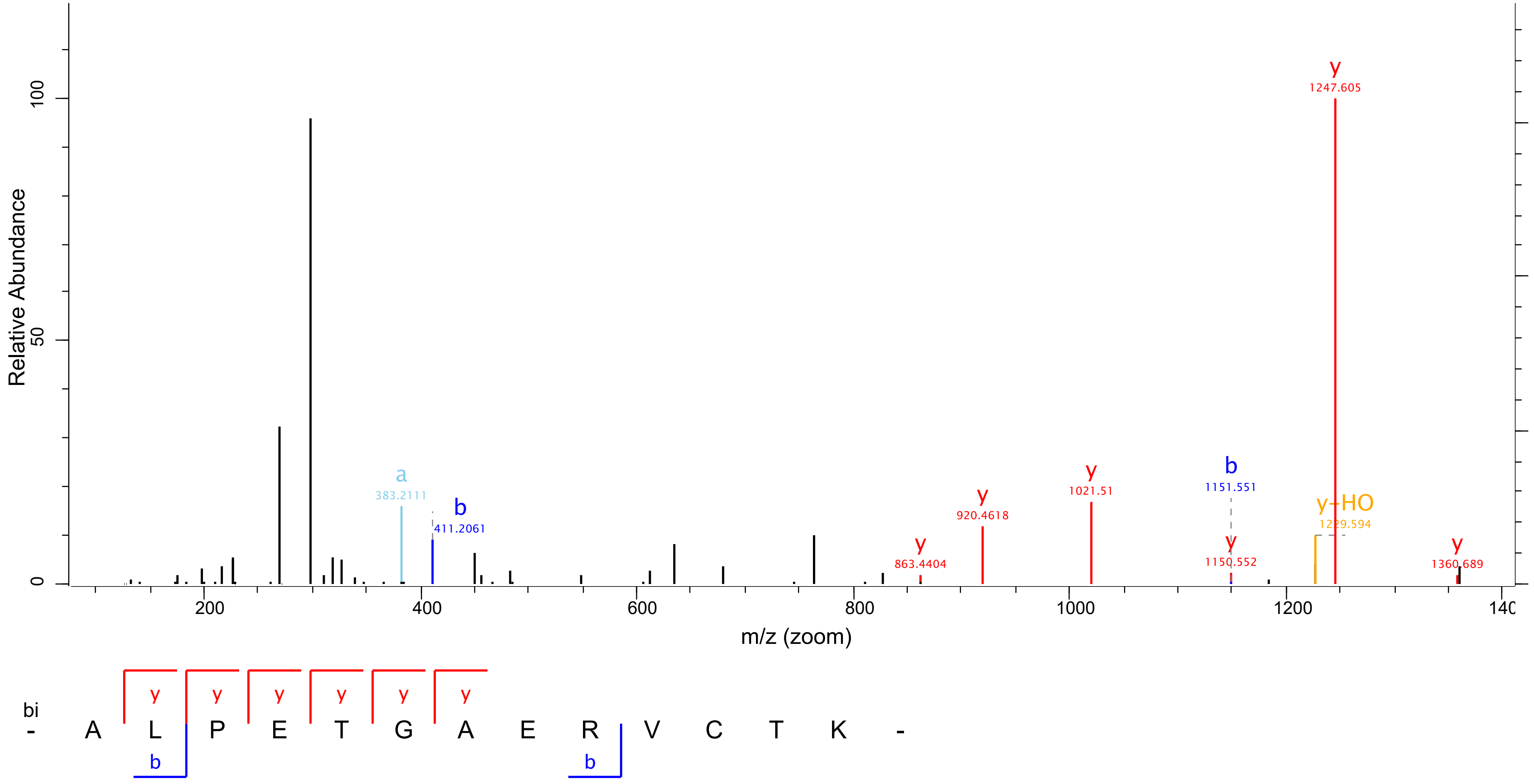

Raw File	Scan	Method	Score	m/z	Gene names
agg_pep_sort_pos_1	20808	FTMS; HCD	98.05	687.84	MARCKS

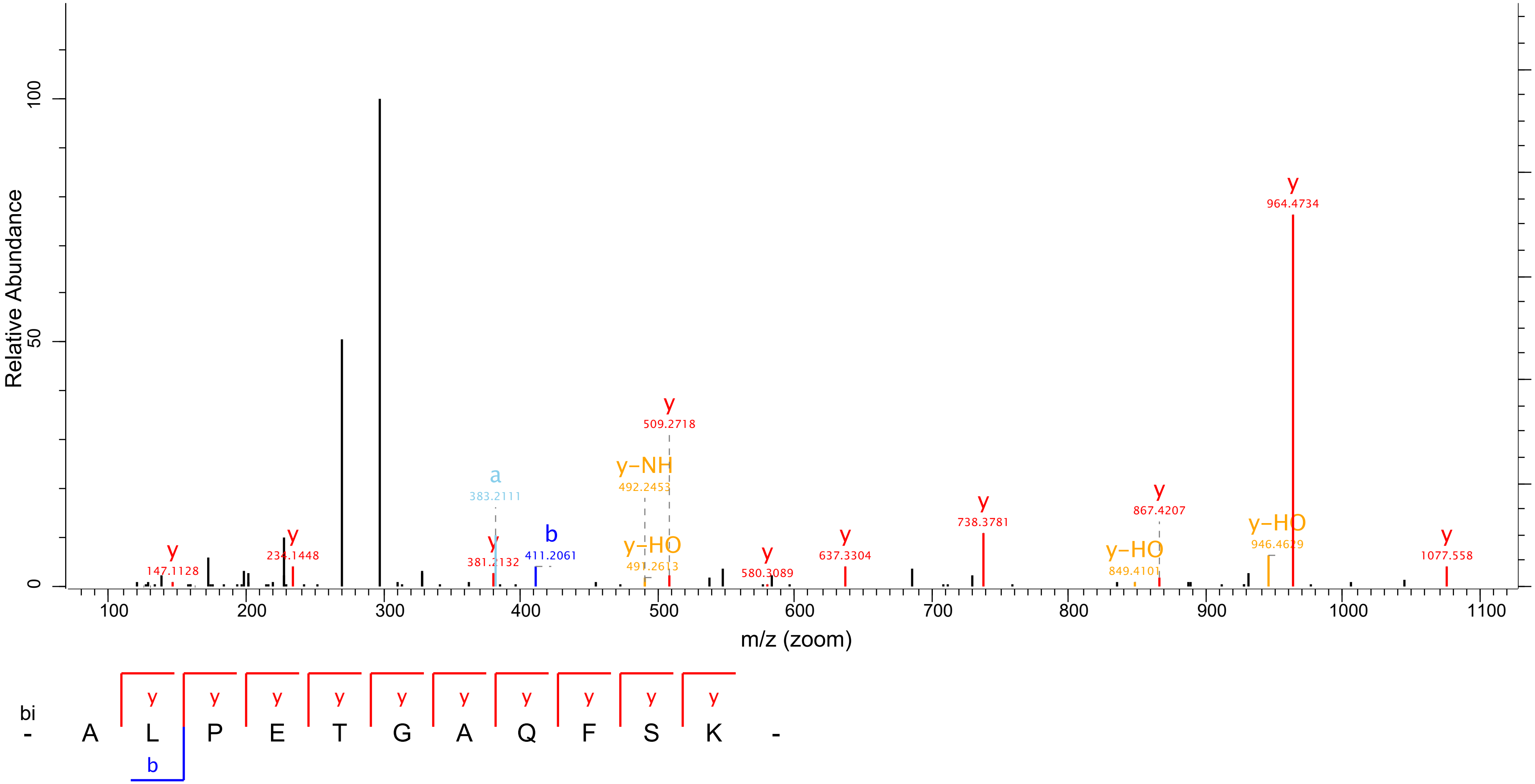

Raw File	Scan	Method	Score	m/z	Gene names
agg_pep_sort_pos_3	9068	FTMS; HCD	89.4	492.92	MBP

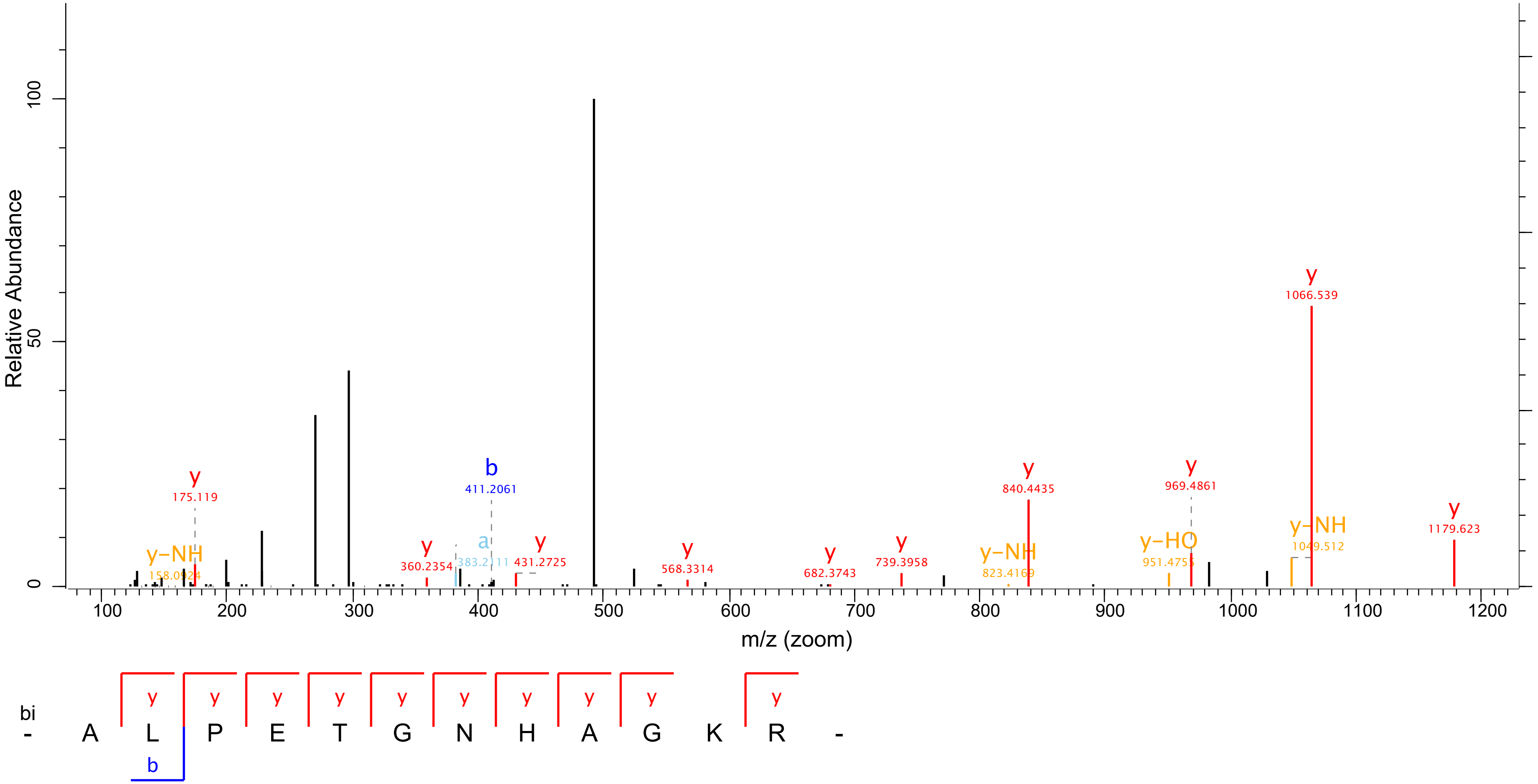

Raw File	Scan	Method	Score	m/z	Gene names
agg_pep_sort_pos_3	15847	FTMS; HCD	78.15	649.35	MRPL11

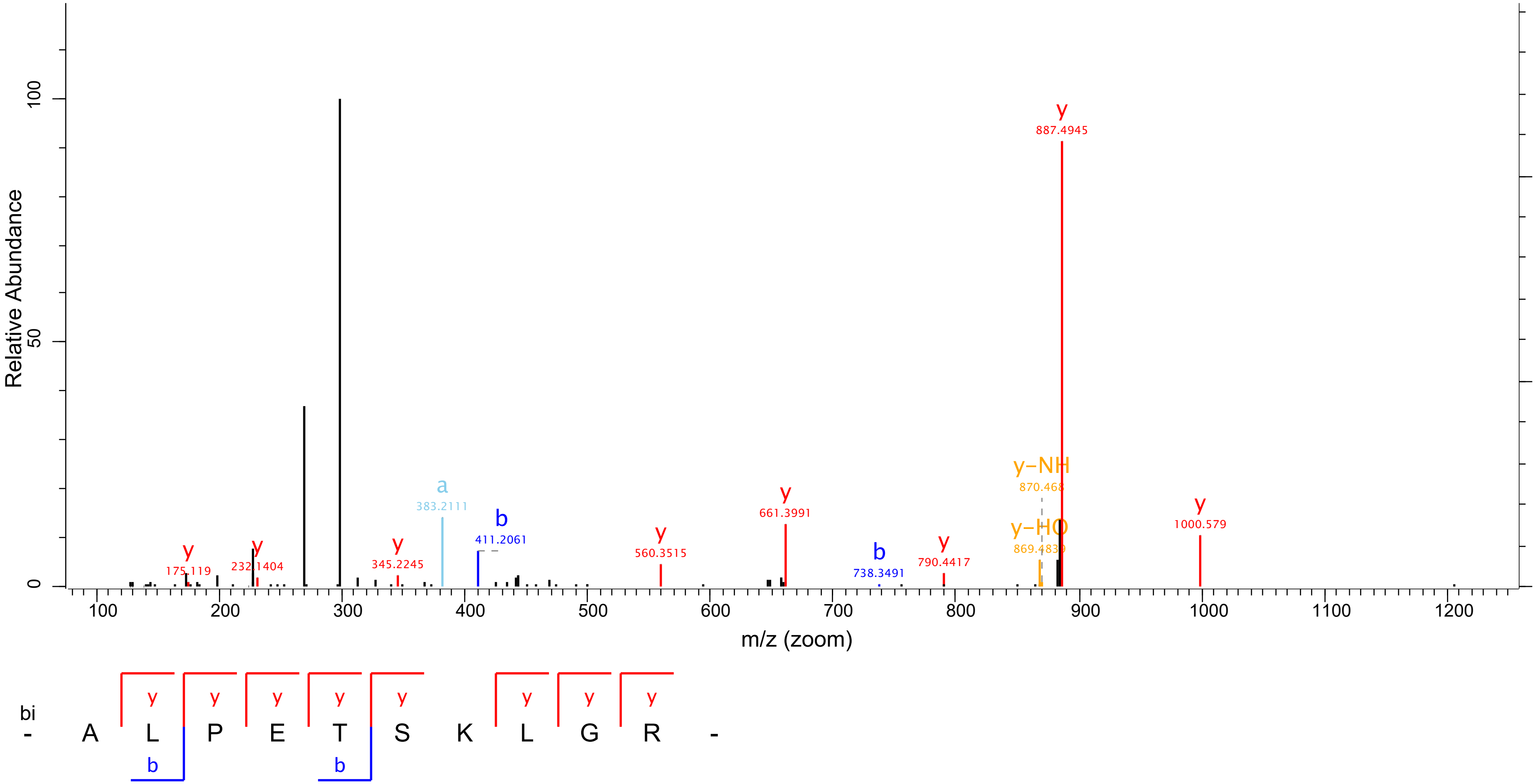

Raw File	Scan	Method	Score	m/z	Gene names
agg_pep_sort_pos_2	17309	FTMS; HCD	88.68	769.42	MSN

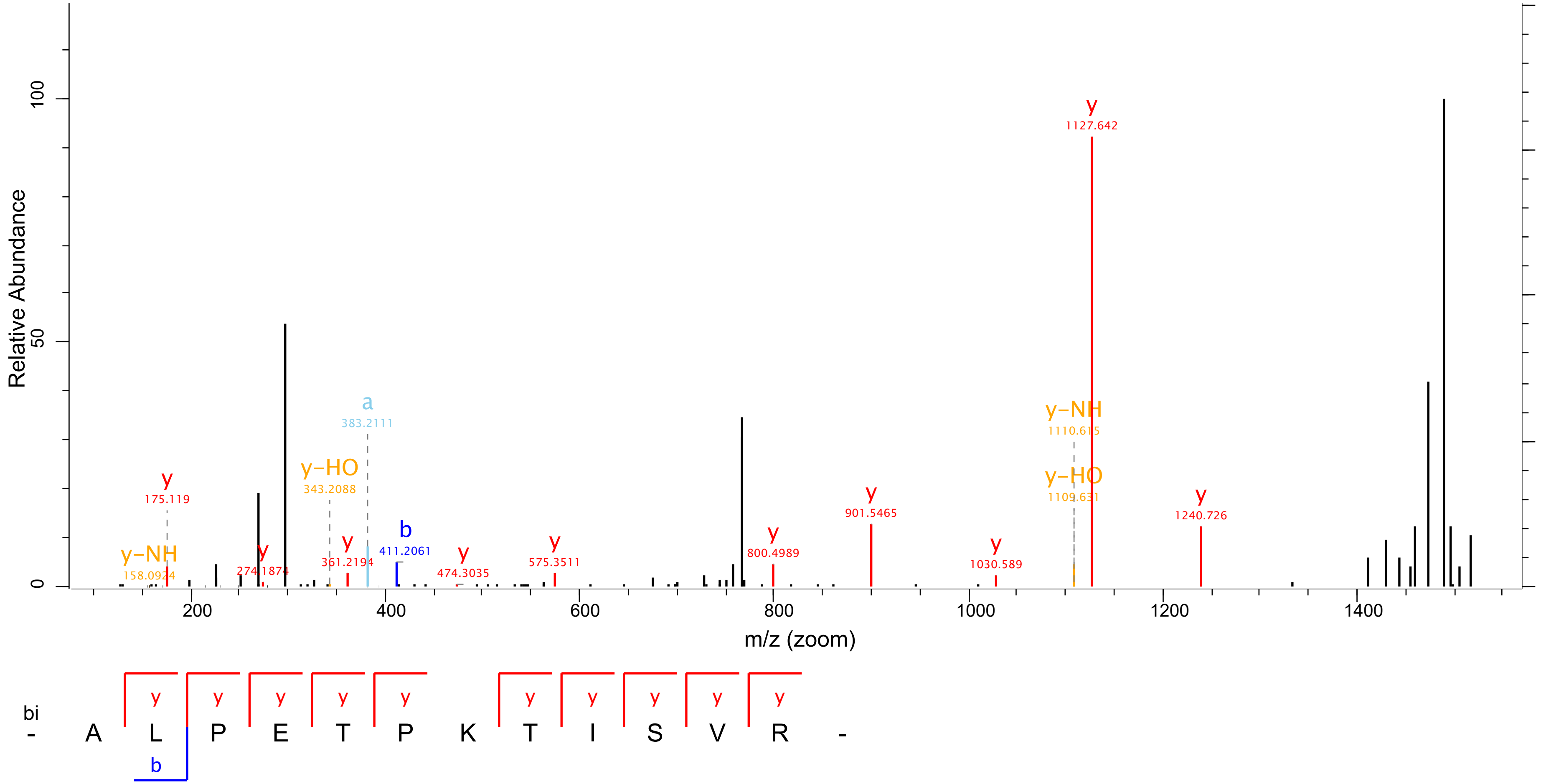

Raw File	Scan	Method	Score	m/z
agg_pep_sort_pos_3	19693	FTMS; HCD	108.4	855.1

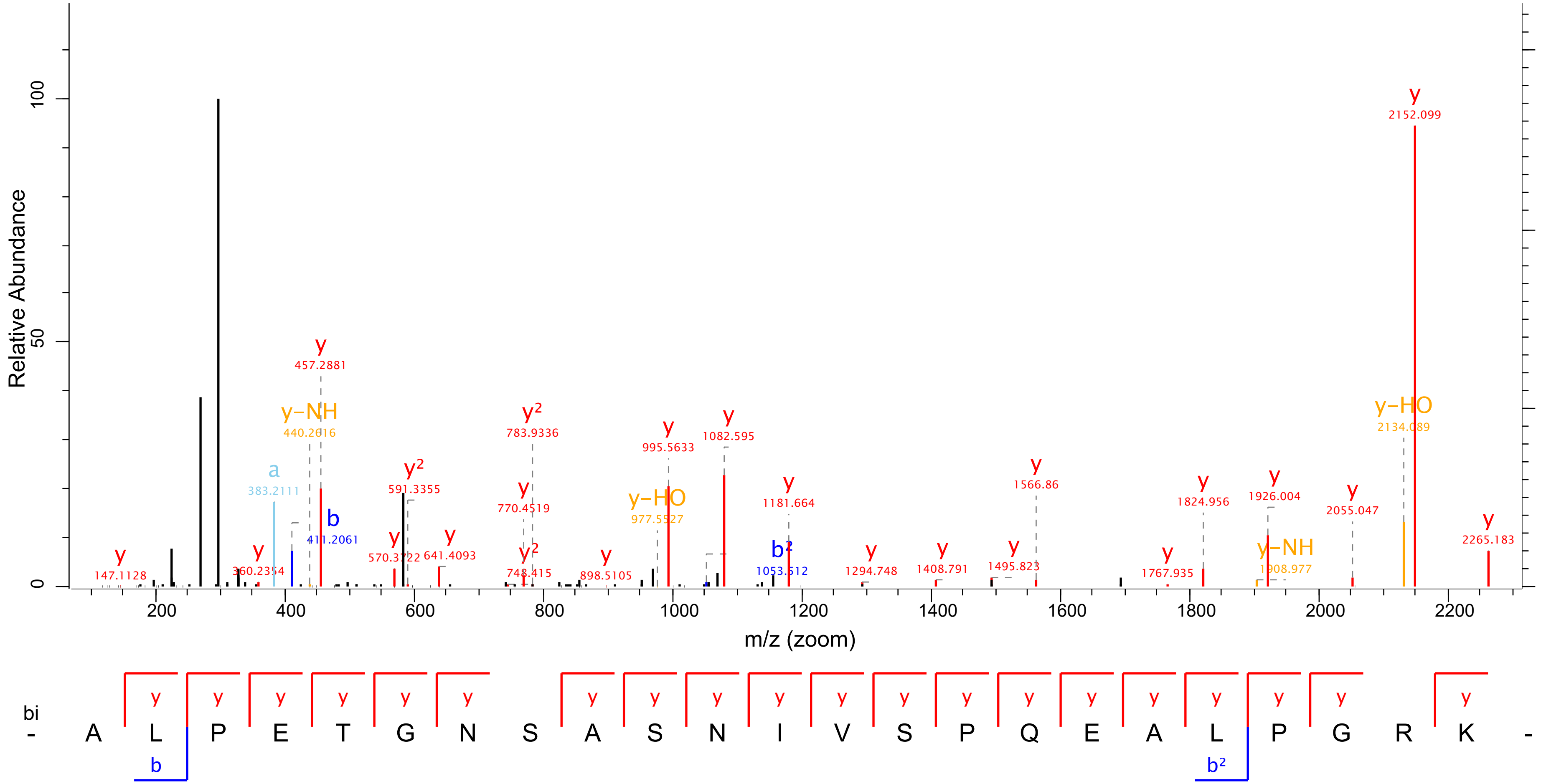

Raw File	Scan	Method	Score	m/z	Gene names
agg_pep_sort_pos_1	13552	FTMS; HCD	39.66	572.27	MYOZ2

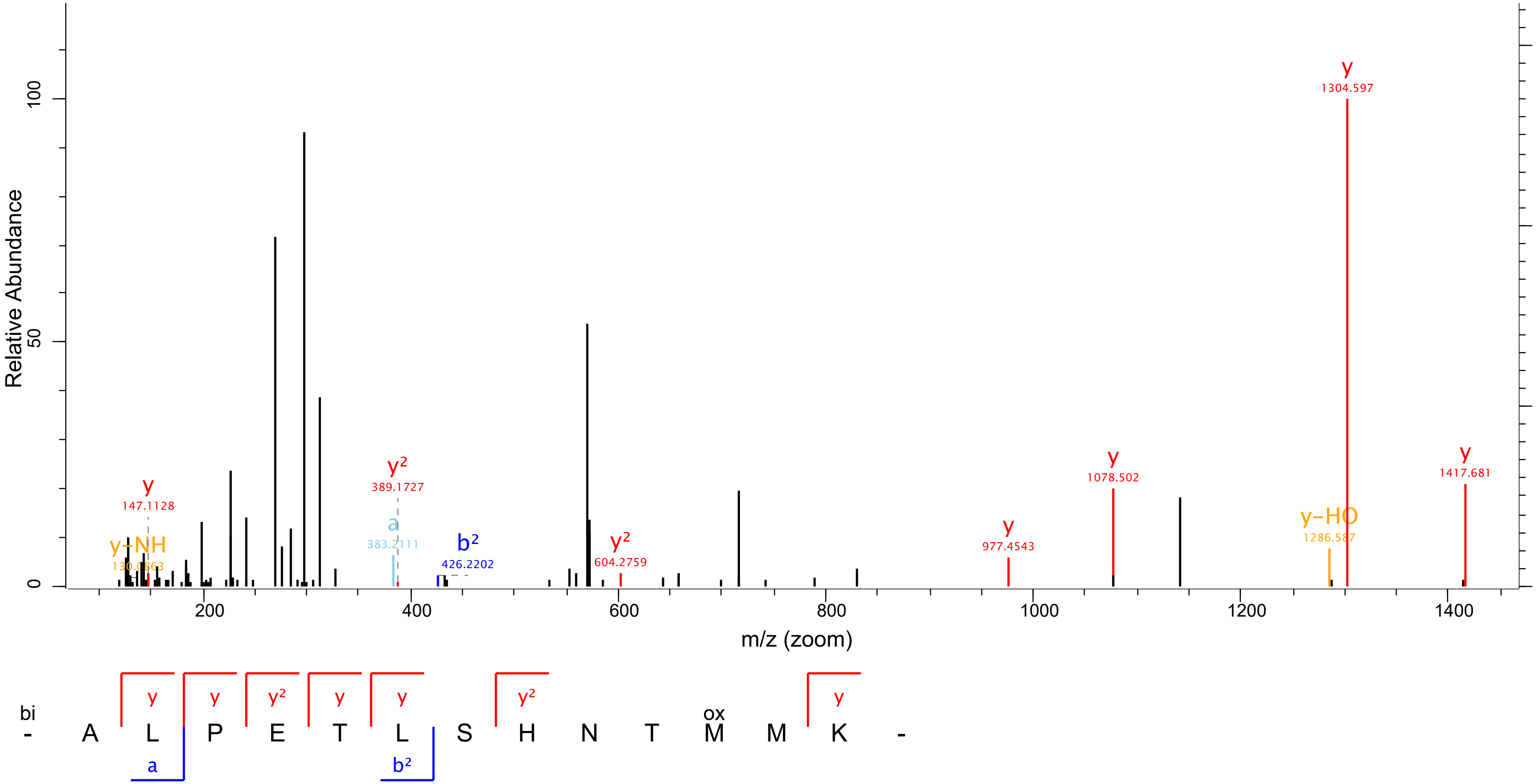

Raw File	Scan	Method	Score	m/z	Gene names
agg_pep_sort_pos_1	17626	FTMS; HCD	40.43	852.94	NBPF1

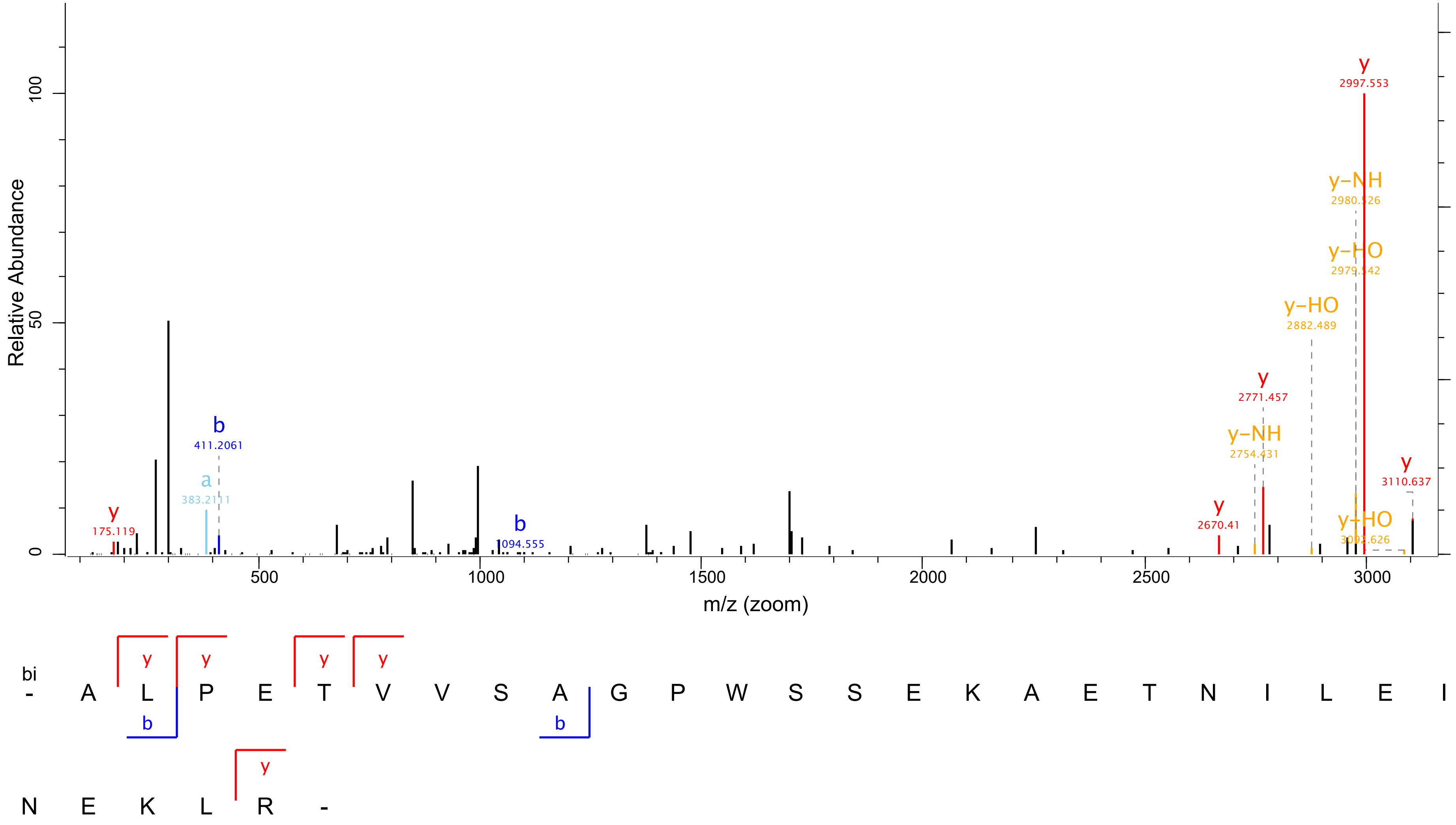

Raw File	Scan	Method	Score	m/z	Gene names
agg_pep_sort_pos_2	23621	FTMS; HCD	72.8	874.75	NCAPH

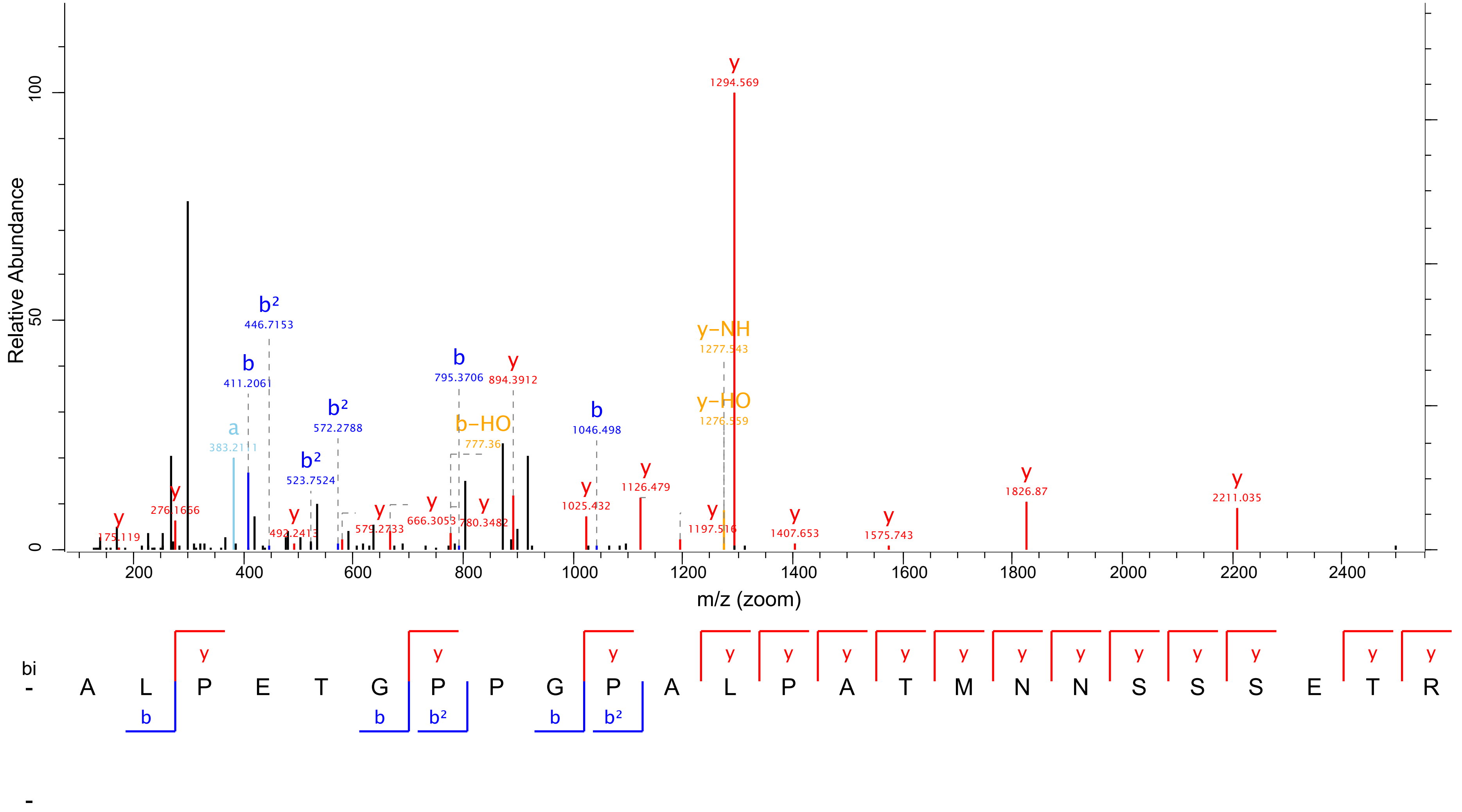

Raw File	Scan	Method	Score	m/z	Gene names
agg_pep_sort_pos_2	13981	FTMS; HCD	82.29	710.85	NEURL1B

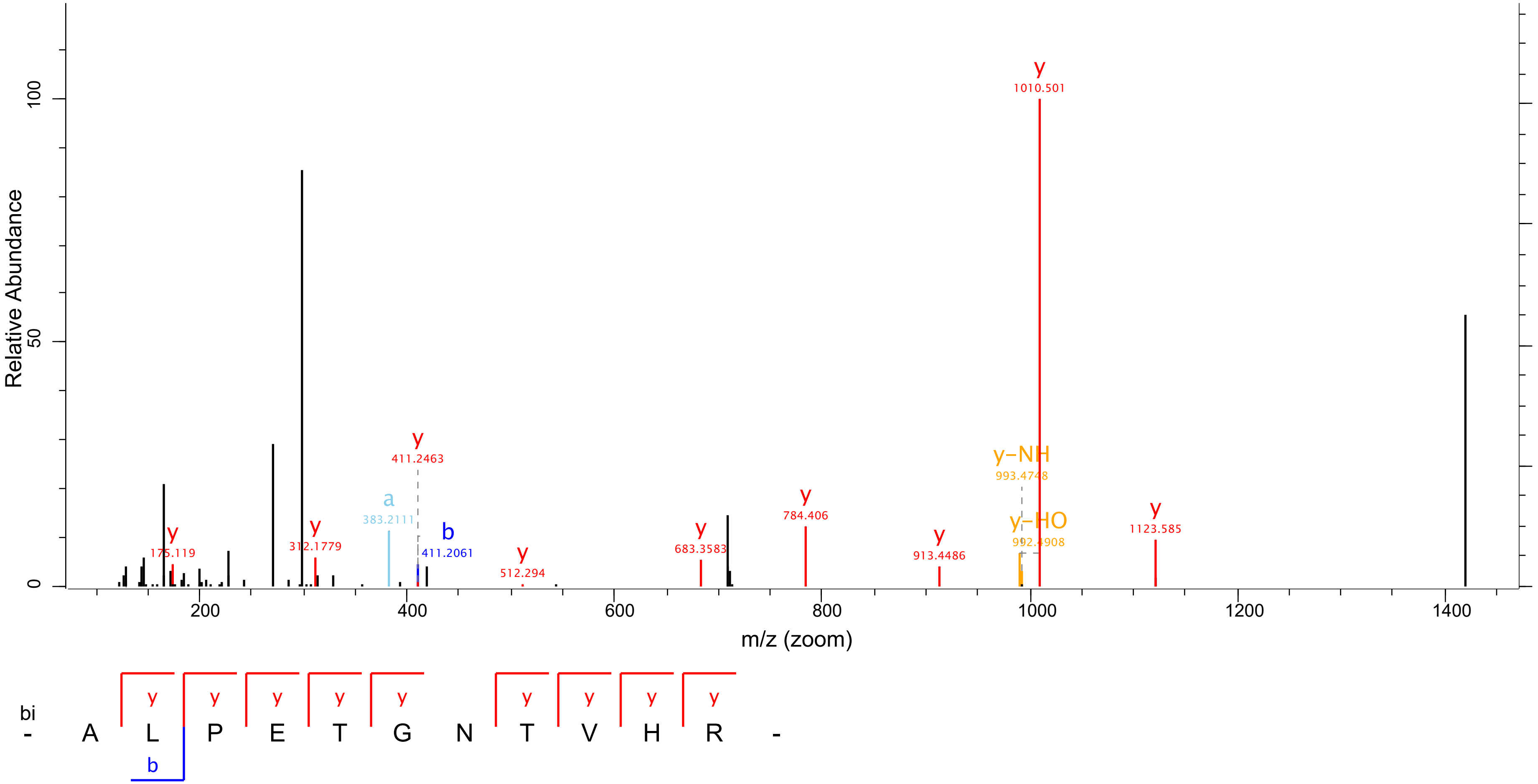

Raw File	Scan	Method	Score	m/z	Gene names
agg_pep_sort_pos_1	21547	FTMS; HCD	65.31	670.34	NOLC1

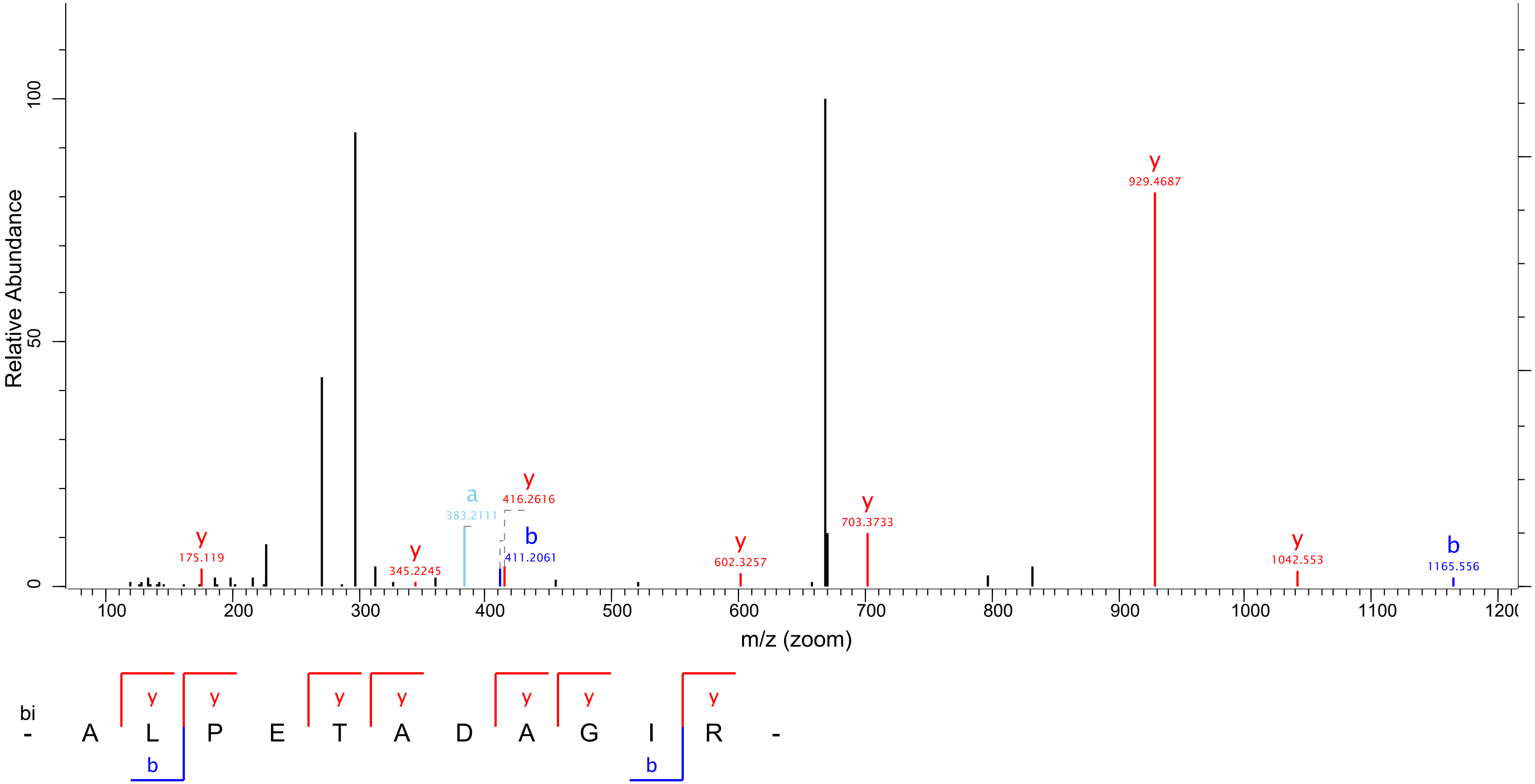

Raw File	Scan	Method	Score	m/z	Gene names
agg_pep_sort_pos_1	13372	FTMS; HCD	78.33	649.34	NOS1AP

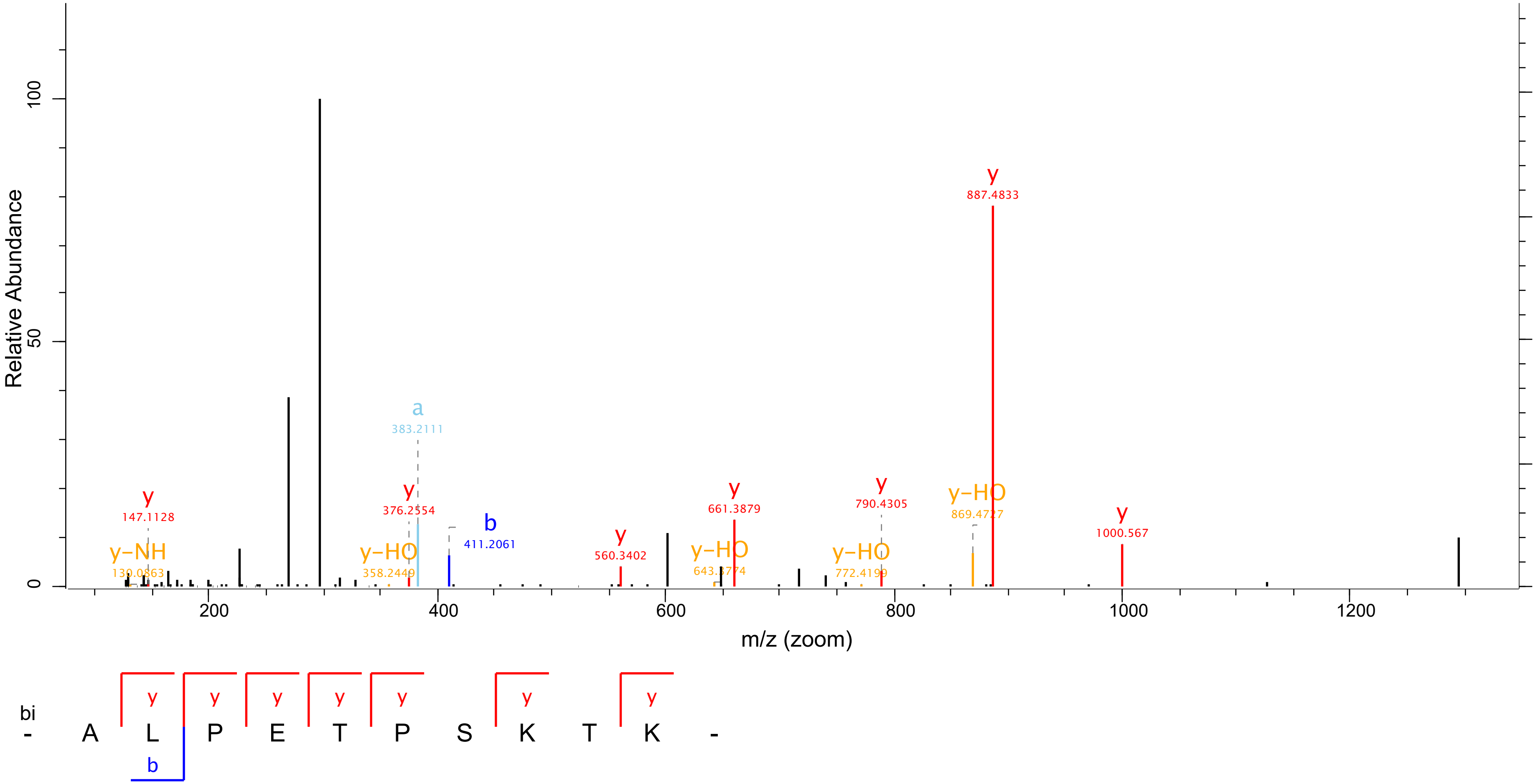

Raw File	Scan	Method	Score	m/z
agg_pep_sort_pos_1	20894	FTMS; HCD	47.29	979.97

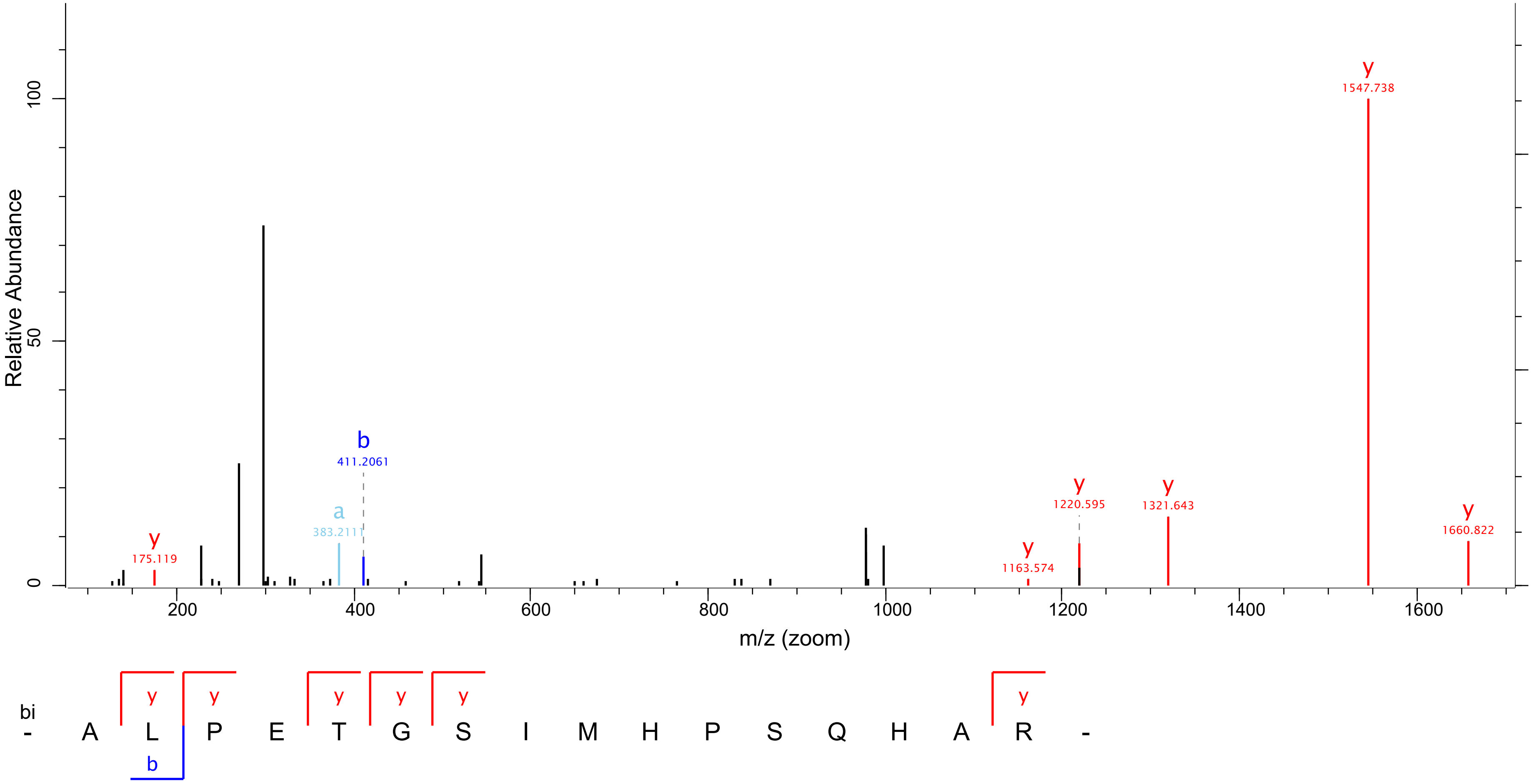

Raw File	Scan	Method	Score	m/z	Gene names
agg_pep_sort_pos_2	17596	FTMS; HCD	61.35	835.87	NUDC

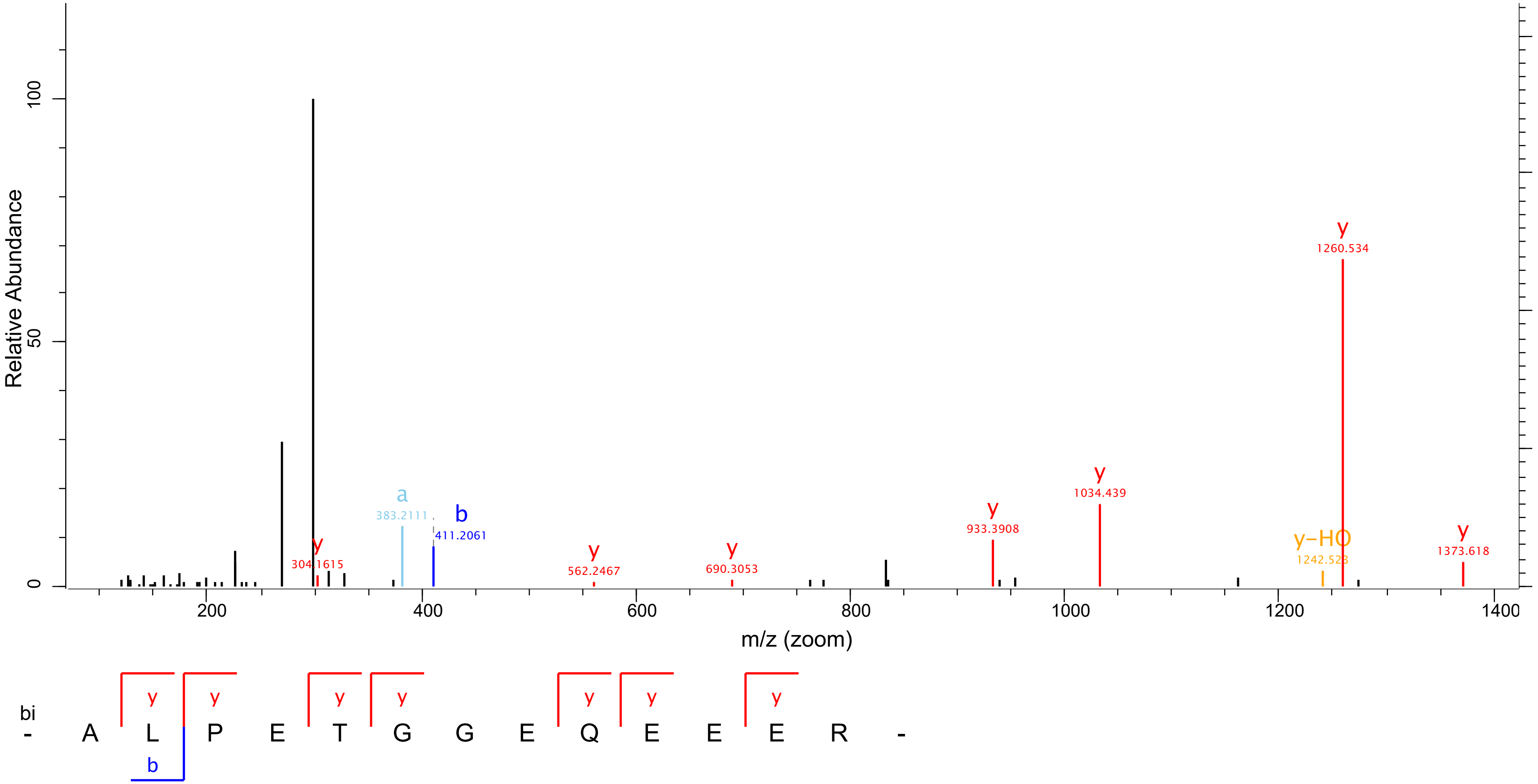

Raw File	Scan	Method	Score	m/z	Gene names
agg_pep_sort_pos_2	21201	FTMS; HCD	68.73	702.35	PPAP2A

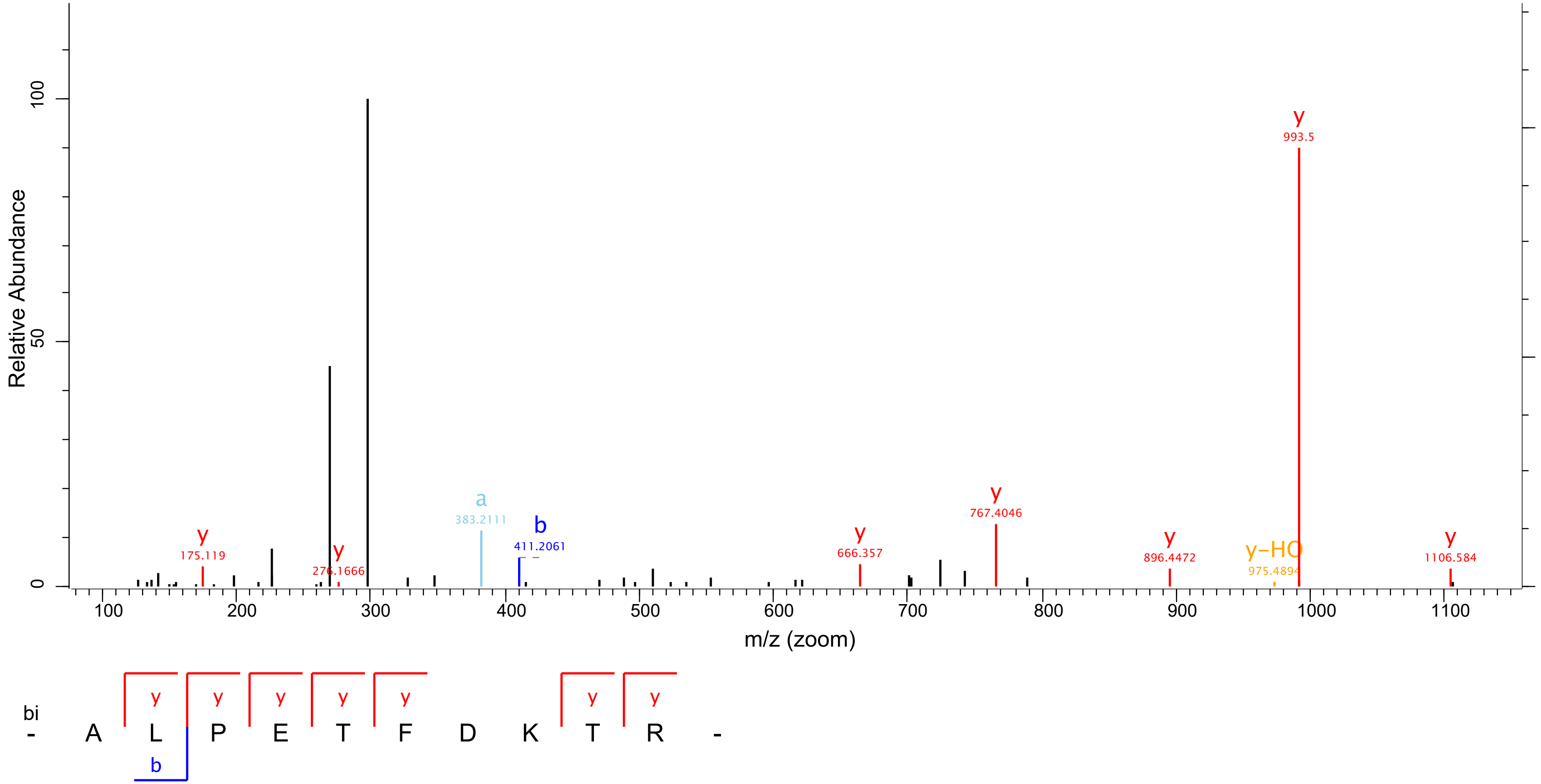

Raw File	Scan	Method	Score	m/z	Gene names
agg_pep_sort_pos_1	17696	FTMS; HCD	45.33	658.01	PPM1B

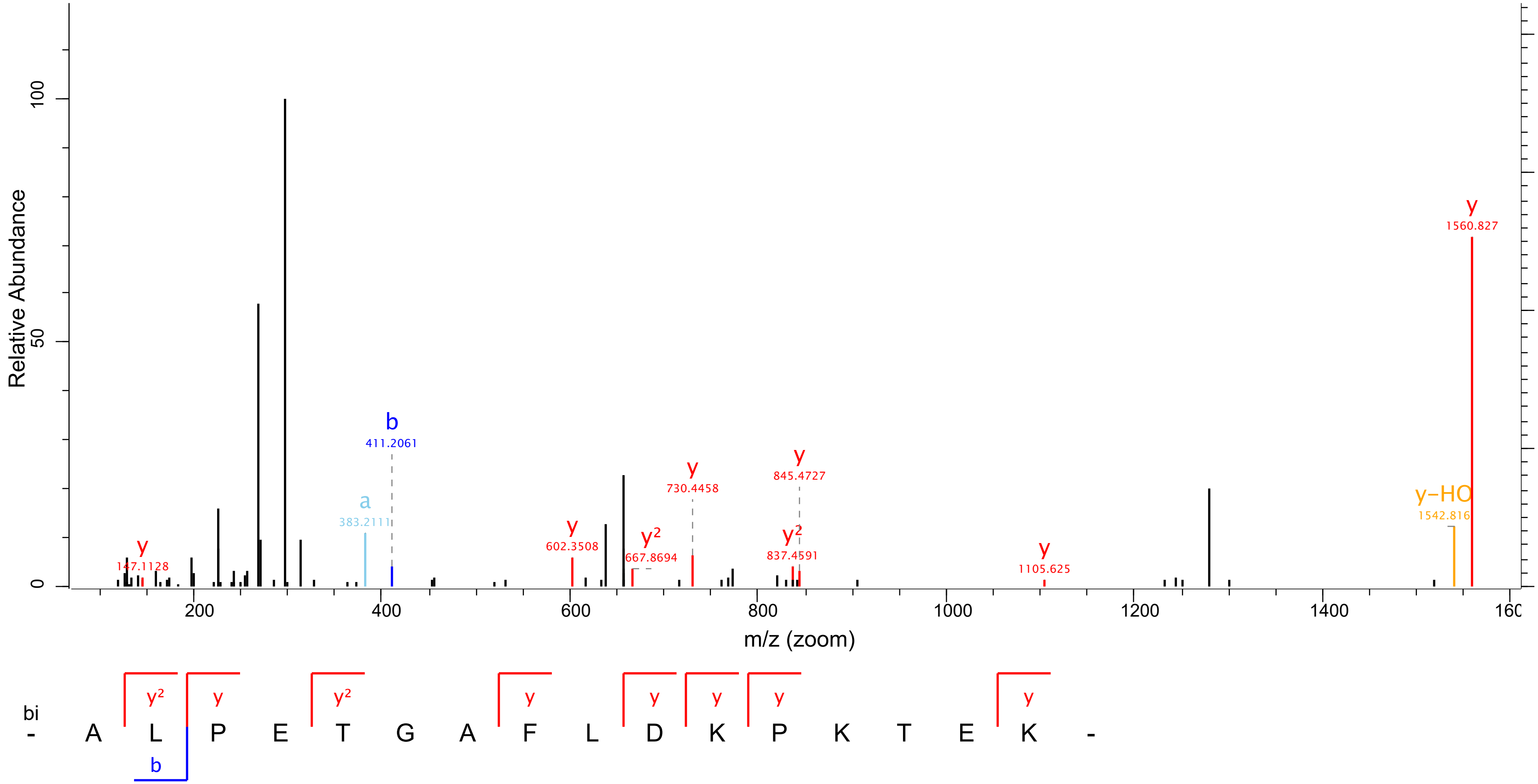

Raw File	Scan	Method	Score	m/z	Gene names
agg_pep_sort_pos_1	17865	FTMS; HCD	112.84	749.88	PPP1R9B

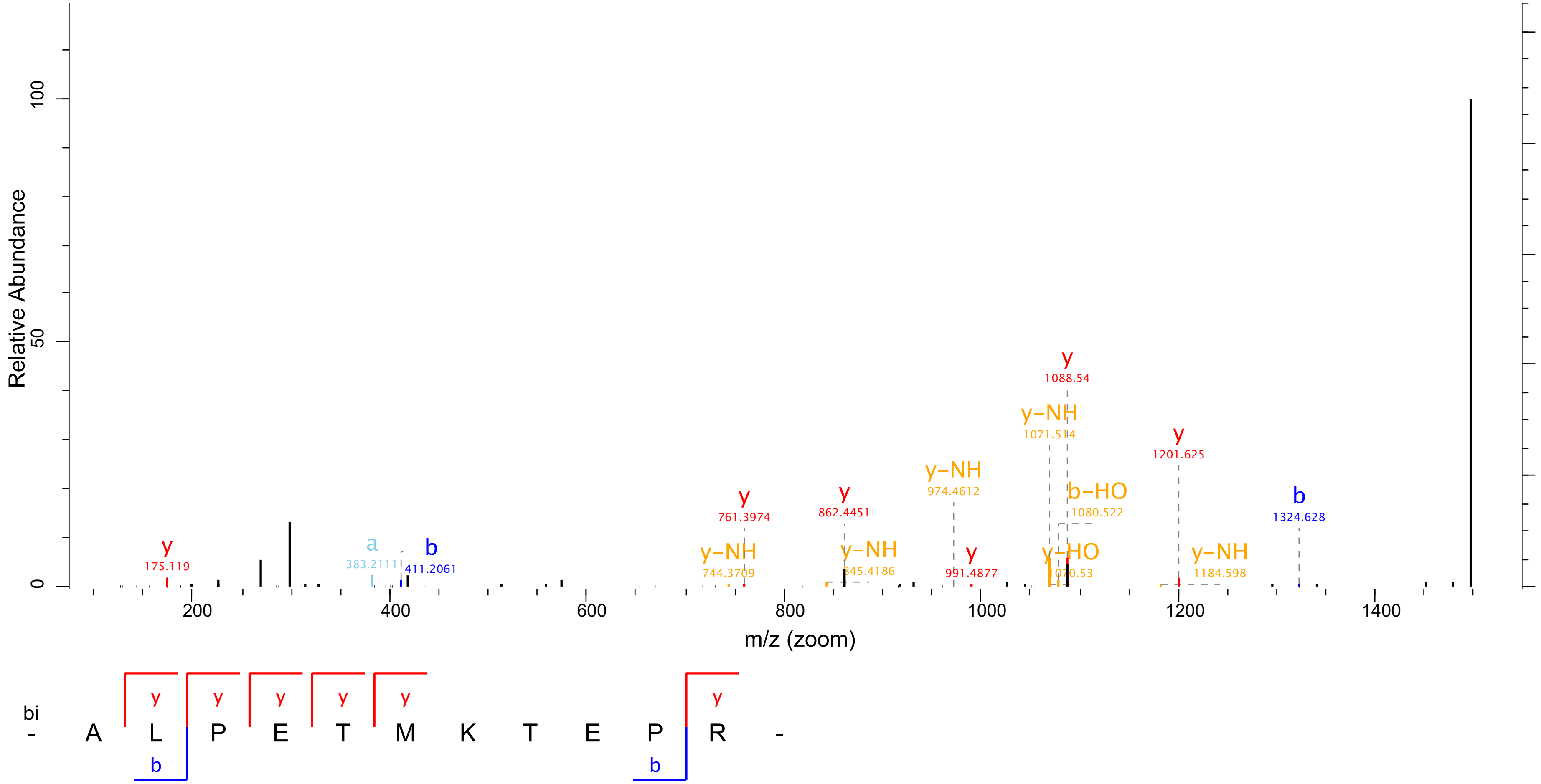

Raw File	Scan	Method	Score	m/z	Gene names
agg_pep_sort_pos_3	19856	FTMS; HCD	32.95	1075.48	PPP3R1

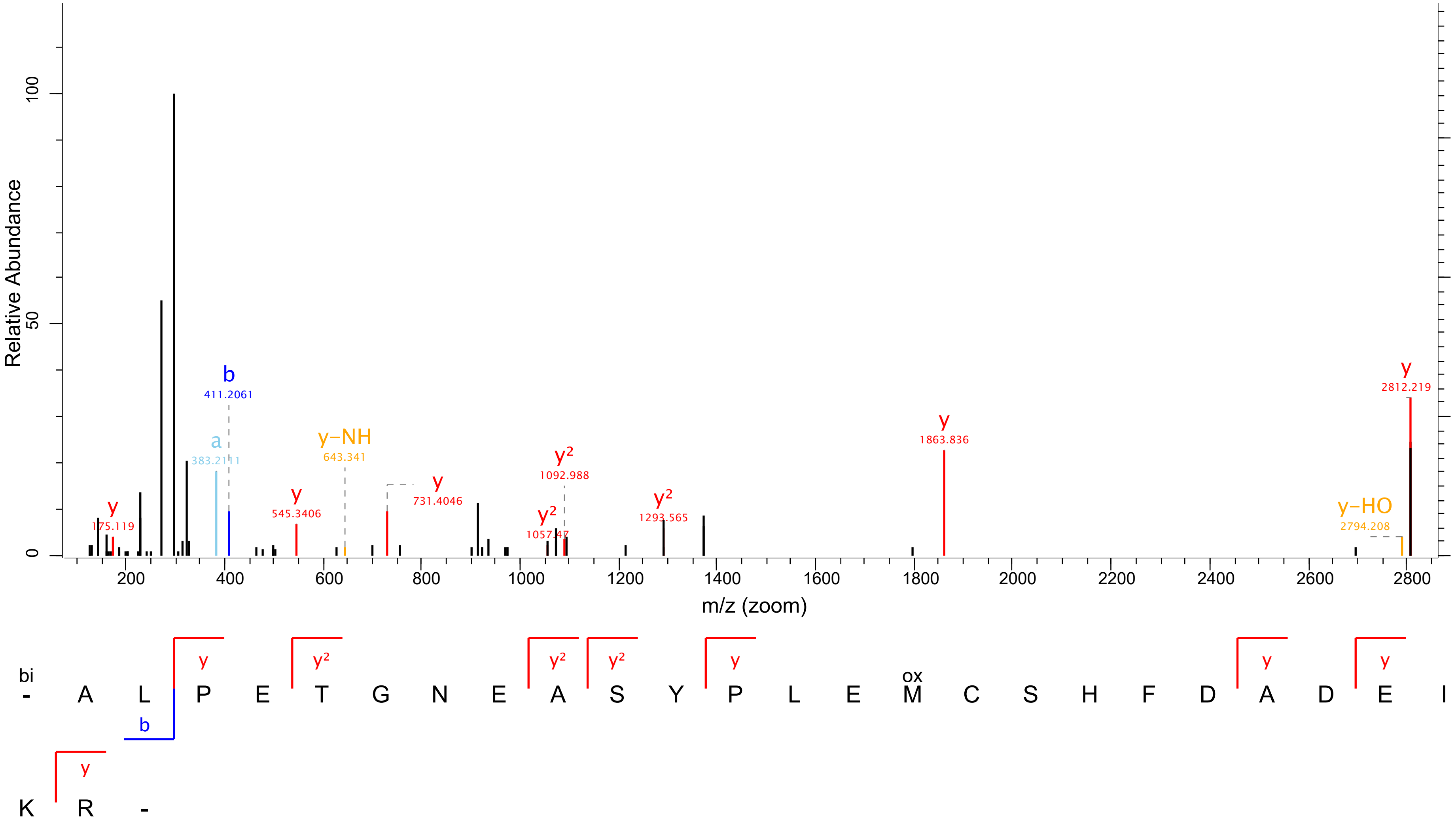

Raw File	Scan	Method	Score	m/z	Gene names
agg_pep_sort_pos_3	21538	FTMS; HCD	42.81	1069.81	PPP3R1

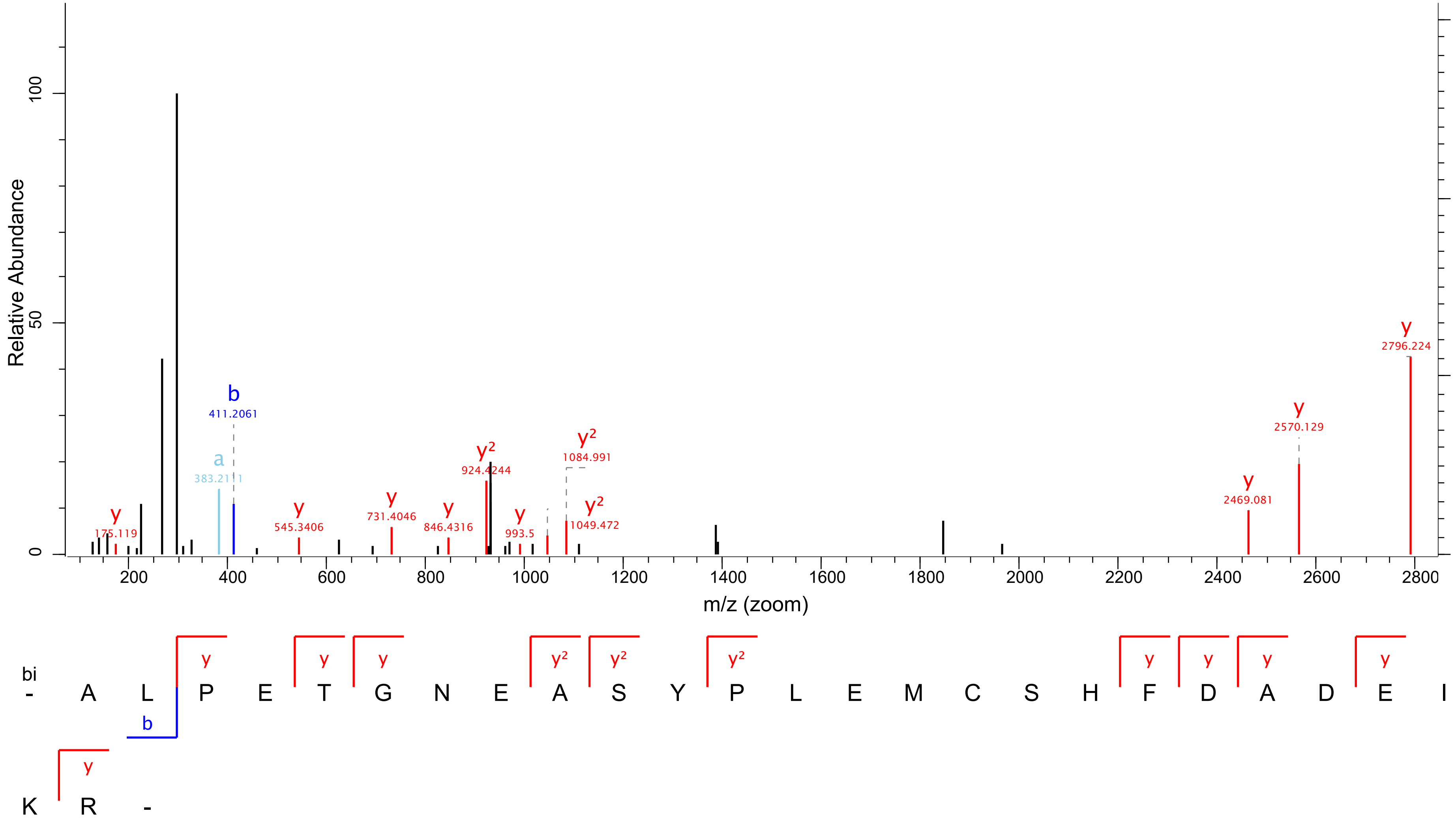

Raw File	Scan	Method	Score	m/z	Gene names
agg_pep_sort_pos_1	12752	FTMS; HCD	105.2	734.39	PRKACA

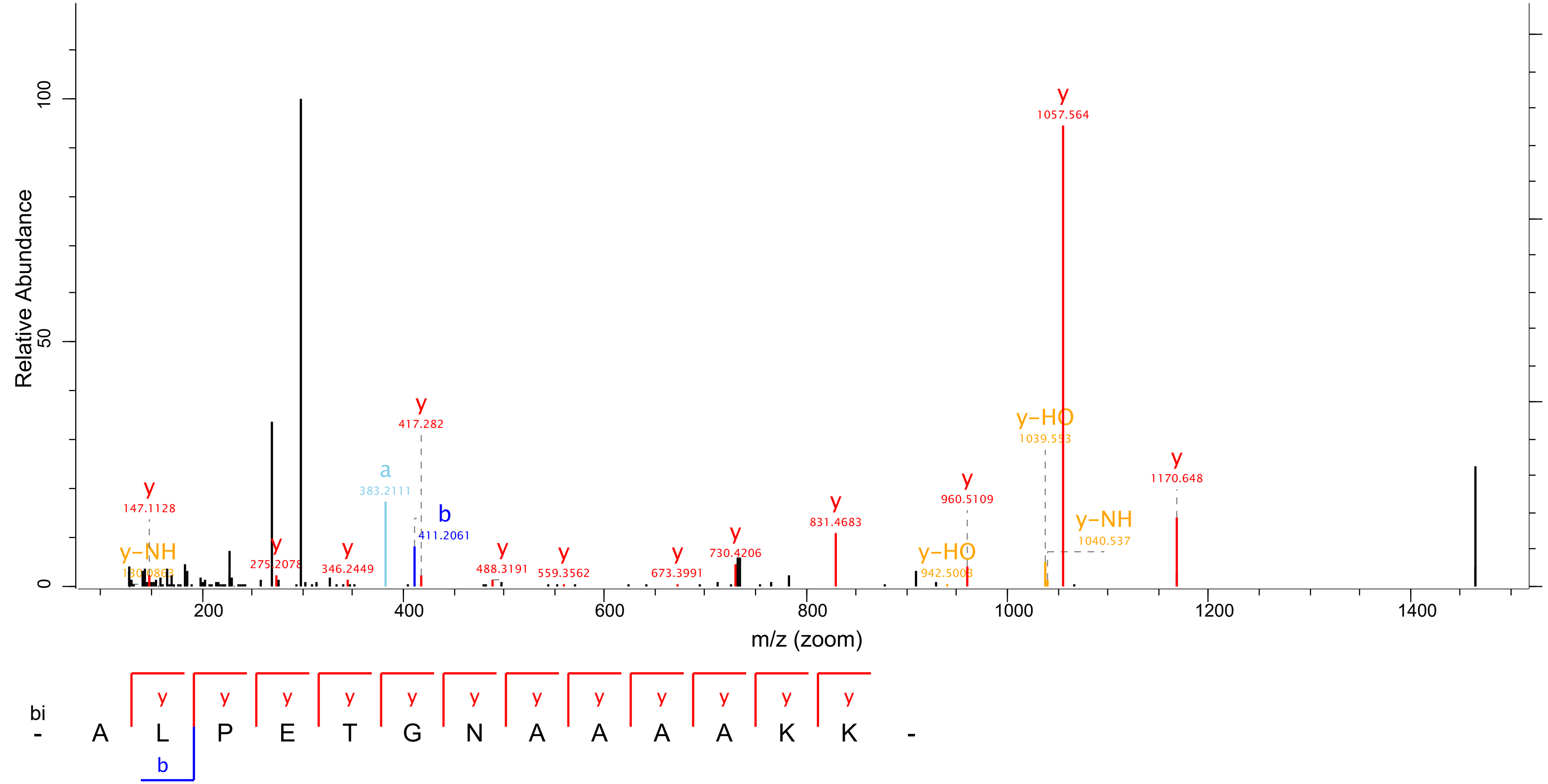

Raw File	Scan	Method	Score	m/z	Gene names
agg_pep_sort_pos_1	17256	FTMS; HCD	107.15	670.34	PRKACA

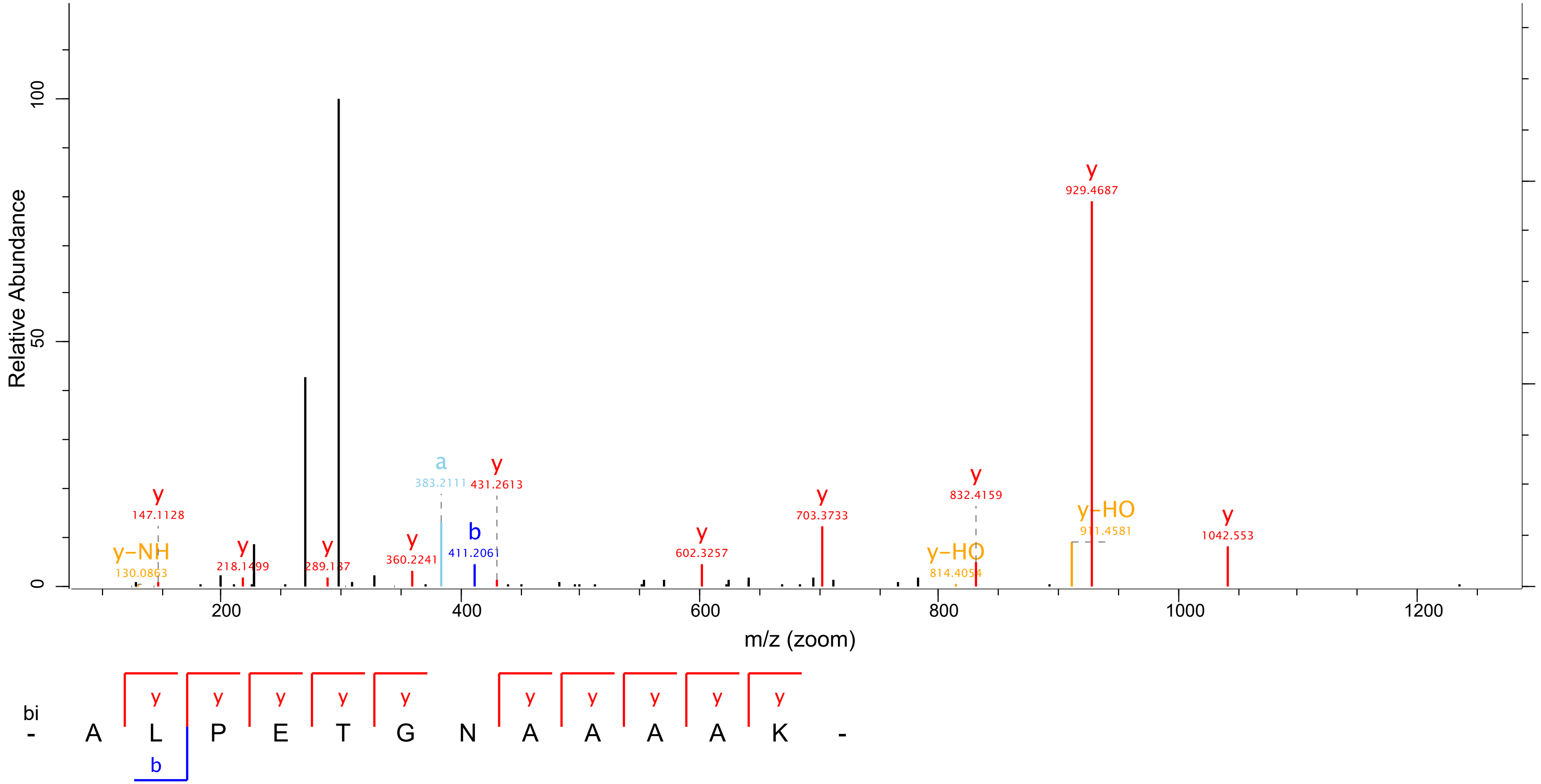

Raw File	Scan	Method	Score	m/z	Gene names
agg_pep_sort_pos_1	20947	FTMS; HCD	142.43	757.43	RPL5

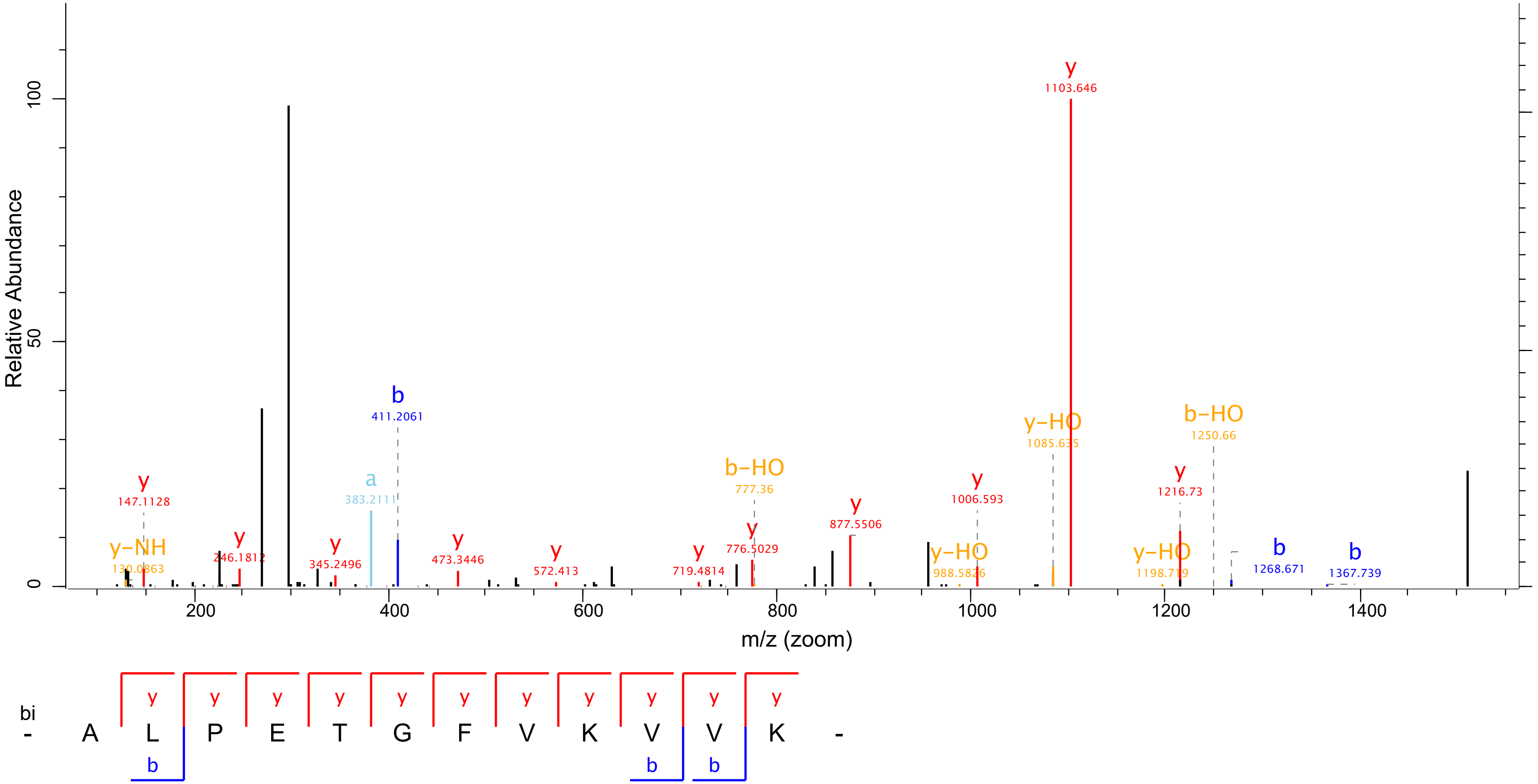

Raw File	Scan	Method	Score	m/z	Gene names
agg_pep_sort_pos_3	12249	FTMS; HCD	174.39	974.45	PSMC1

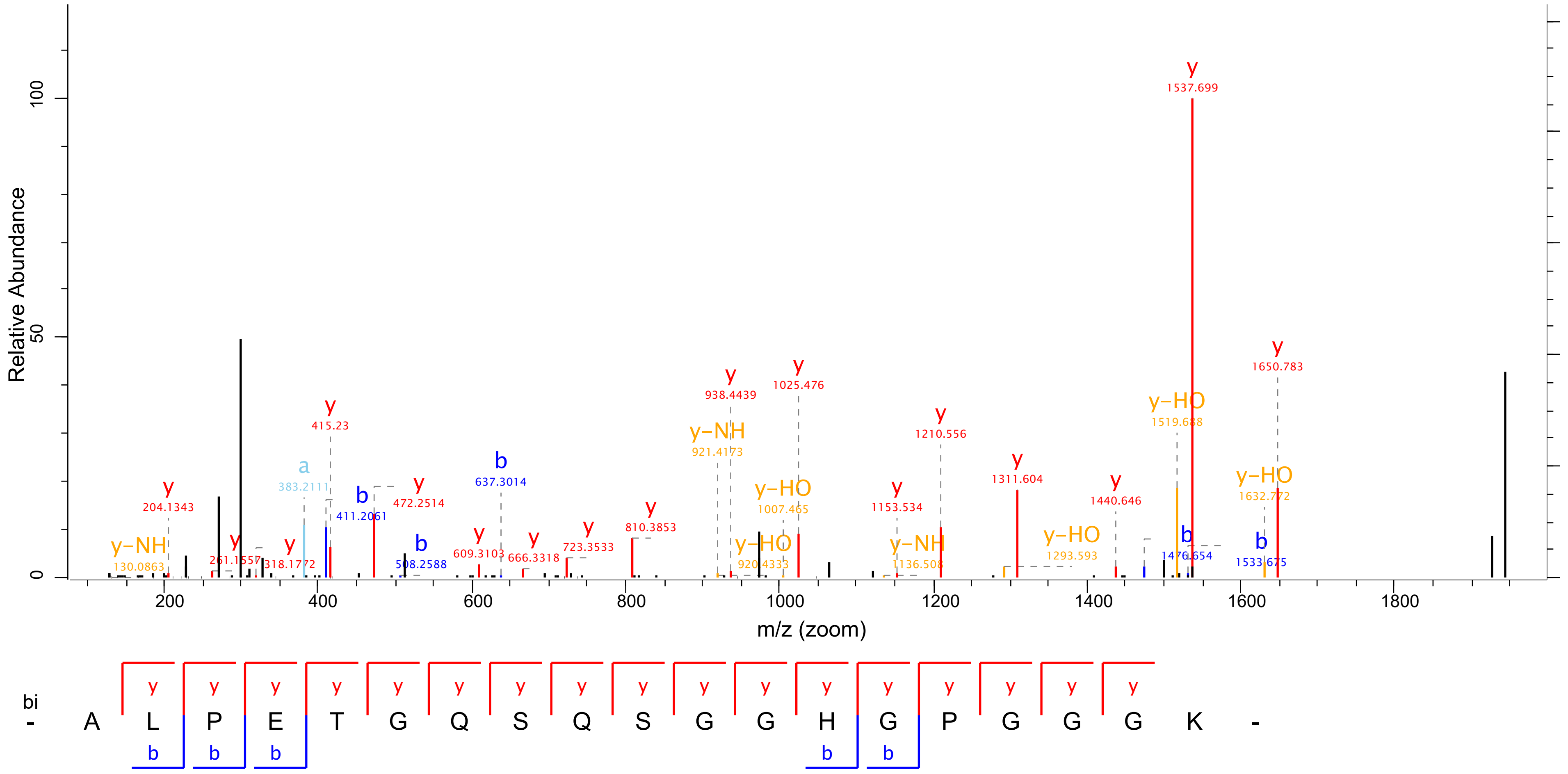

Raw File	Scan	Method	Score	m/z	Gene names
agg_pep_sort_pos_1	8569	FTMS; HCD	117.38	609.54	PSMC1

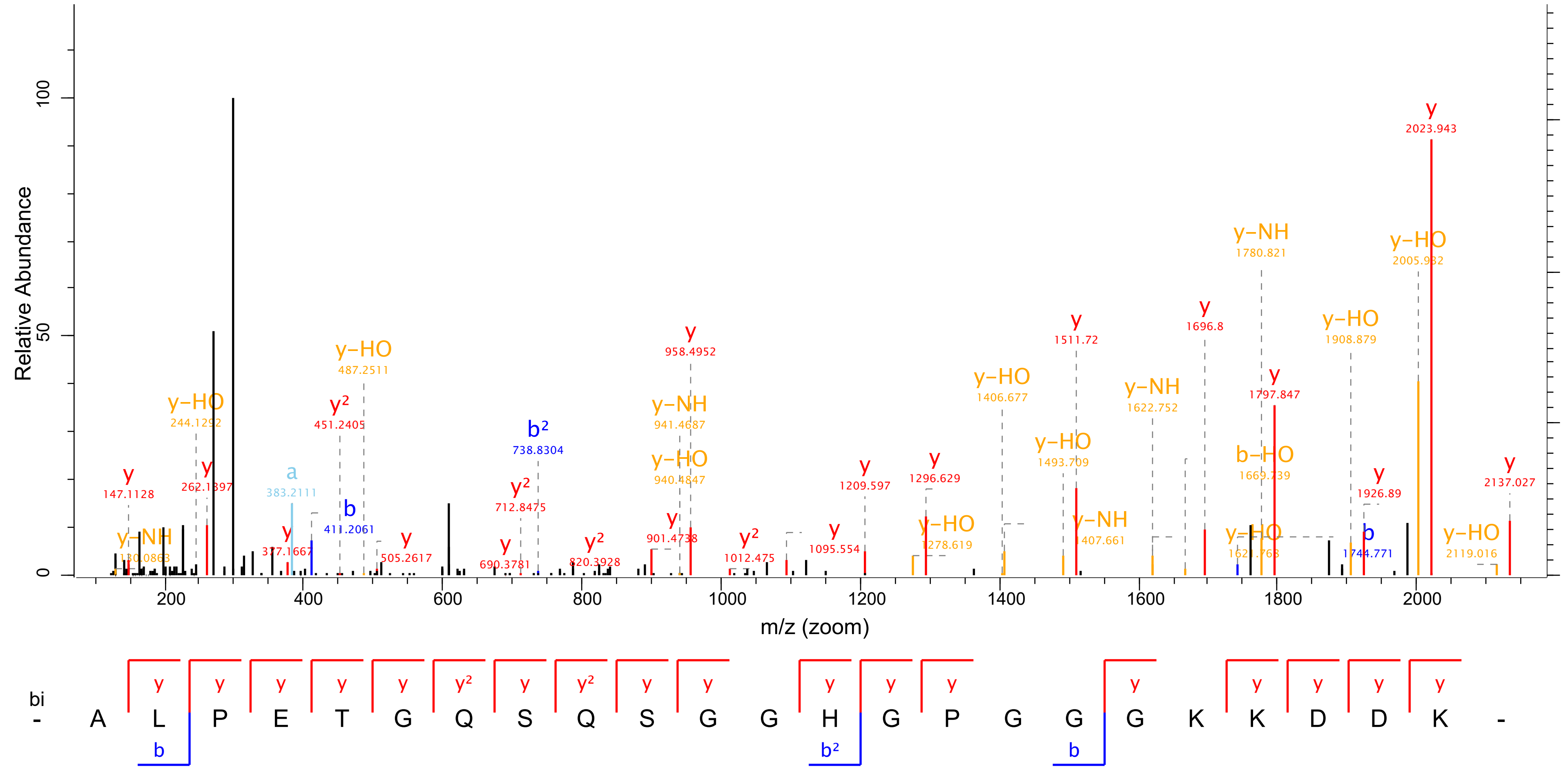

Raw File	Scan	Method	Score	m/z	Gene names
agg_pep_sort_pos_2	9365	FTMS; HCD	95.21	693	PSMC1

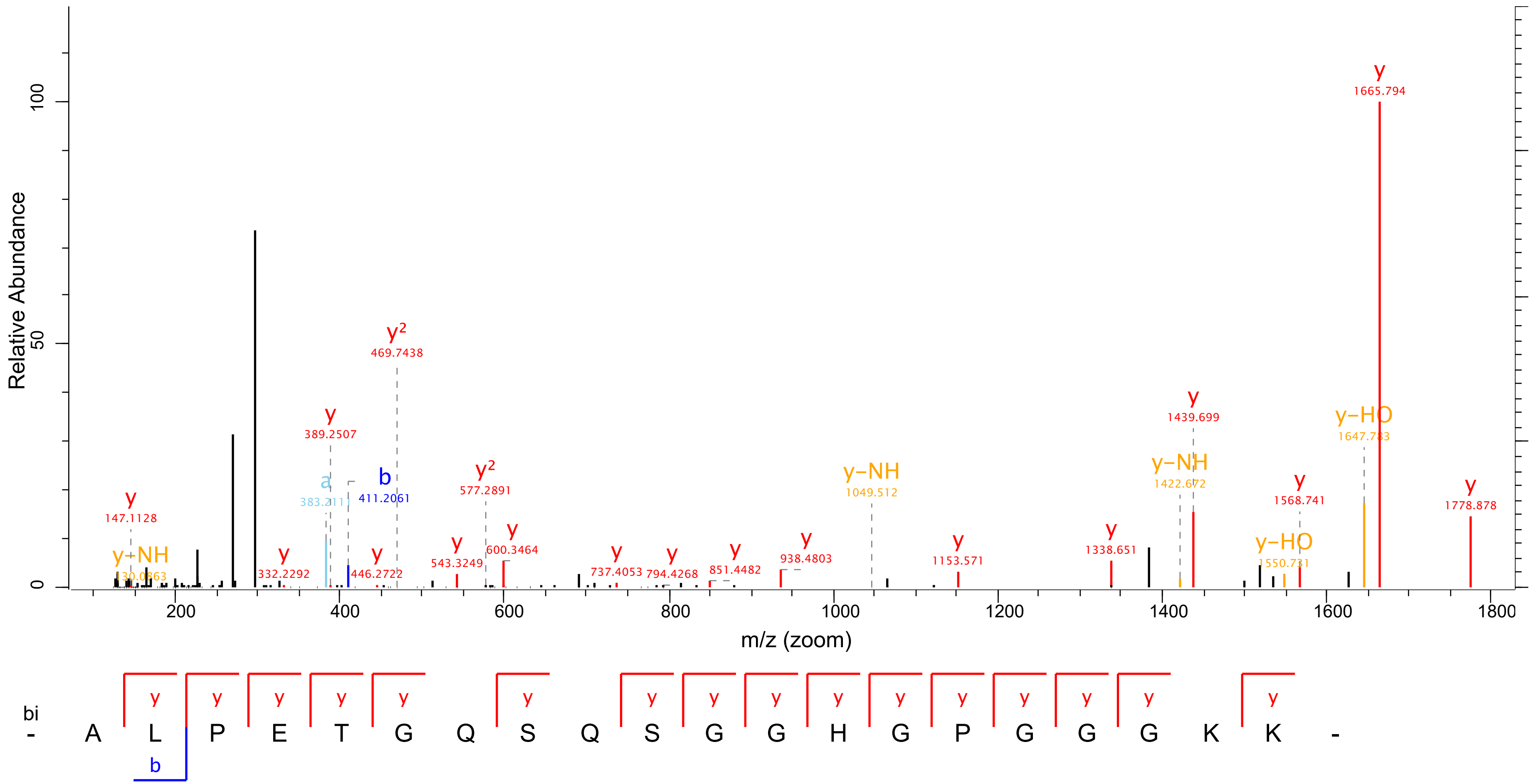

Raw File	Scan	Method	Score	m/z	Gene names
agg_pep_sort_pos_3	19264	FTMS; HCD	68.54	784.87	RBM7

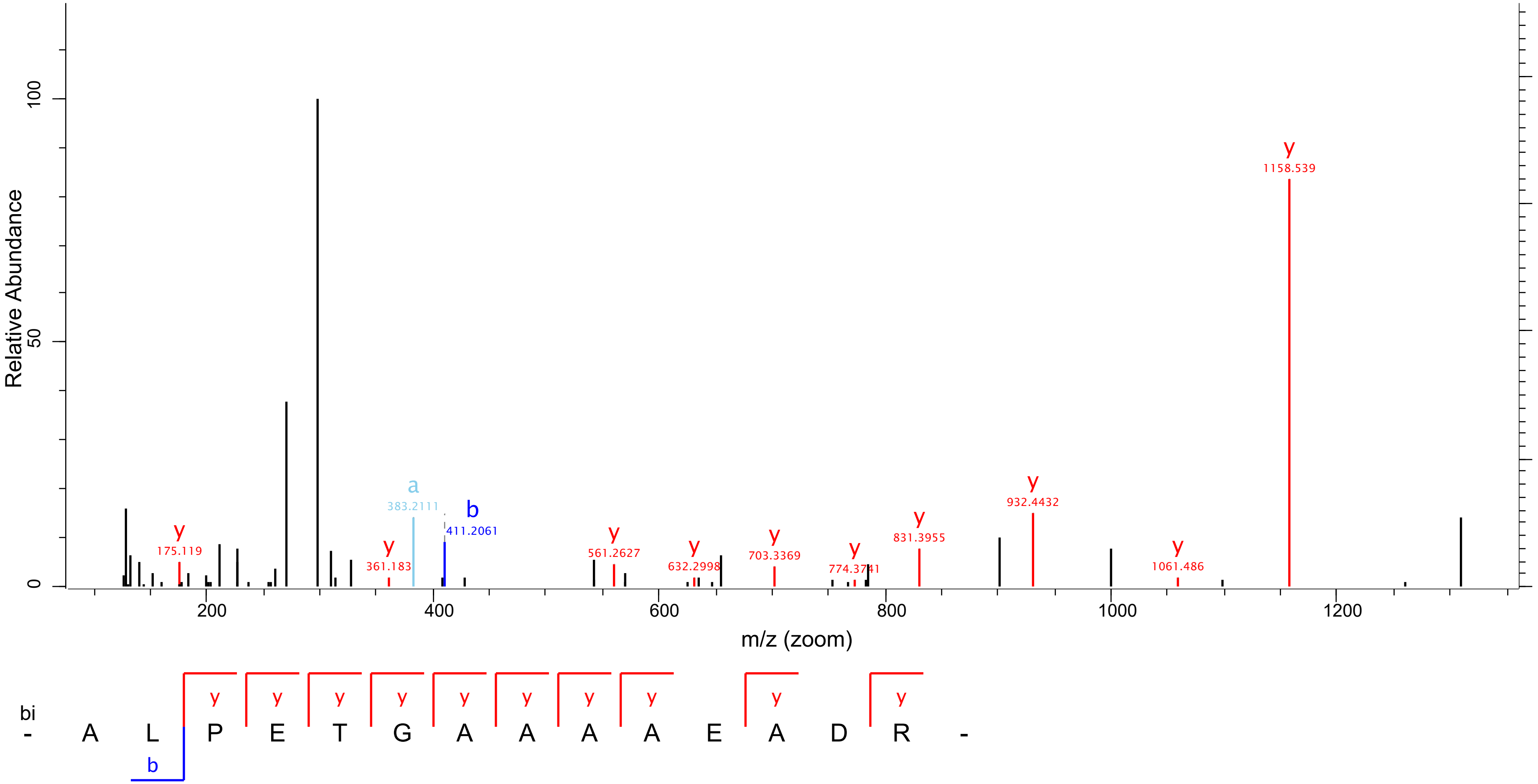

Raw File	Scan	Method	Score	m/z	Gene names
agg_pep_sort_pos_2	15298	FTMS; HCD	89.91	638	RFTN1

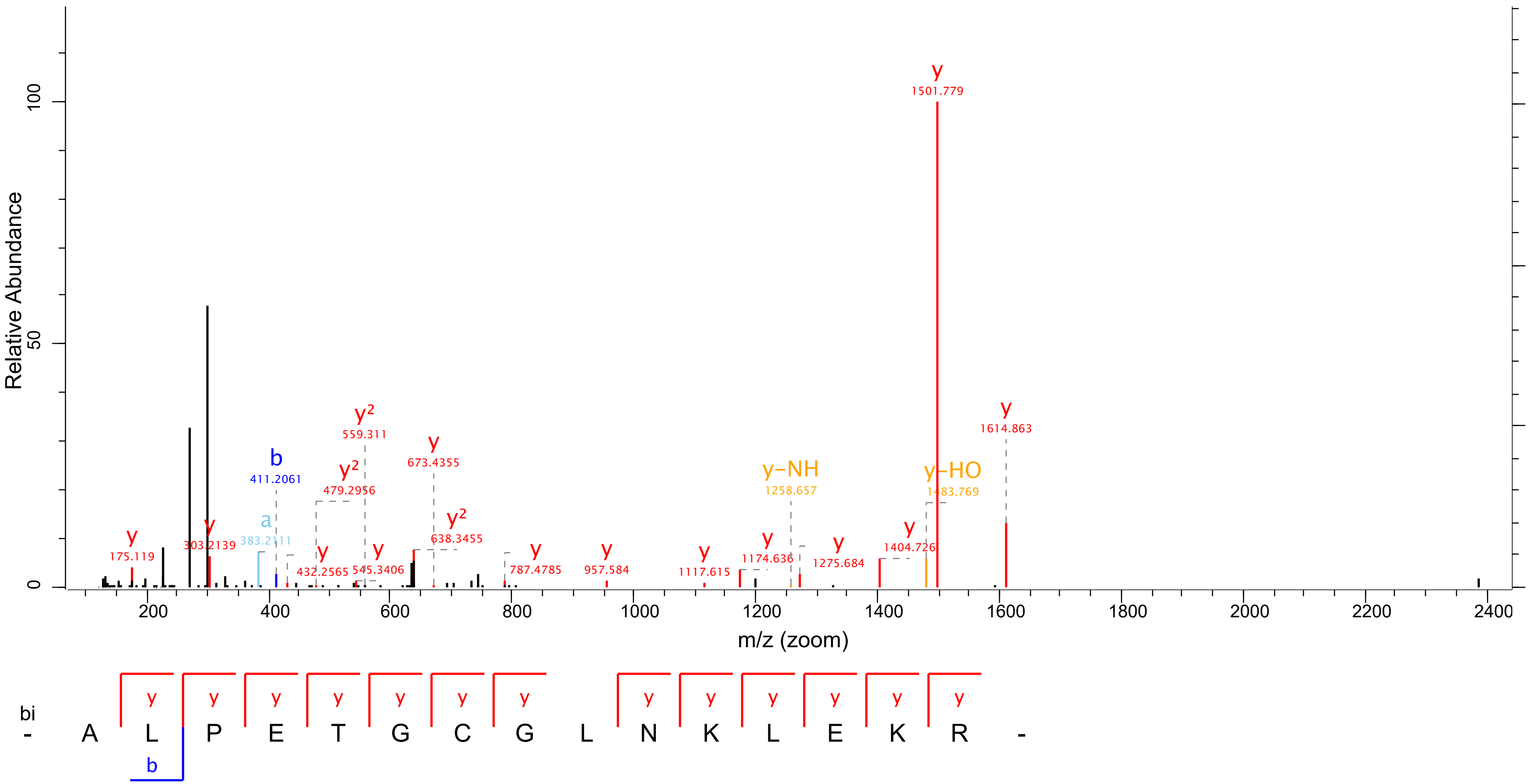

Raw File	Scan	Method	Score	m/z	Gene names
agg_pep_sort_pos_3	17788	FTMS; HCD	108.56	878.44	RFTN1

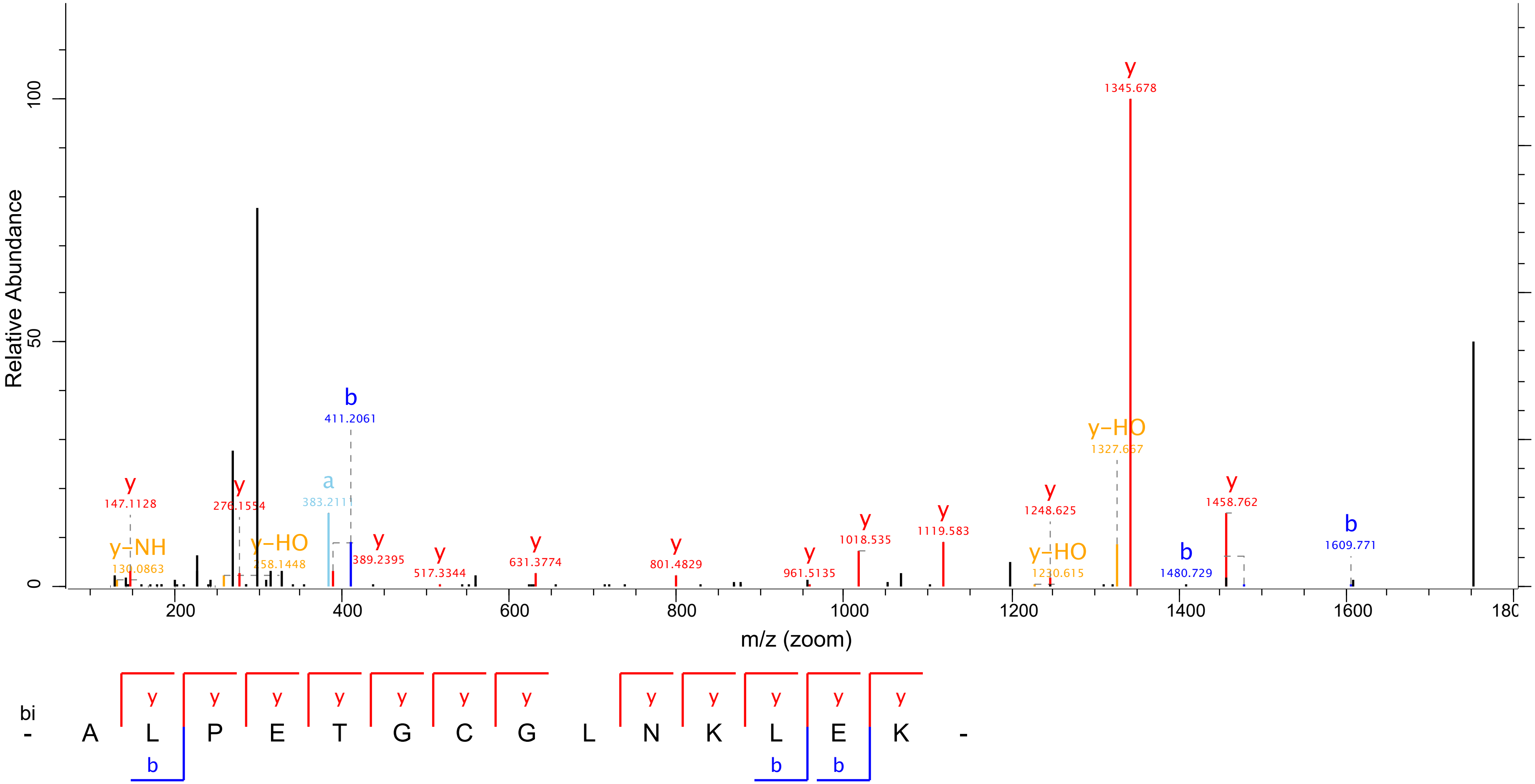

Raw File	Scan	Method	Score	m/z	Gene names
agg_pep_sort_pos_3	18976	FTMS; HCD	80.24	693.33	RFTN1

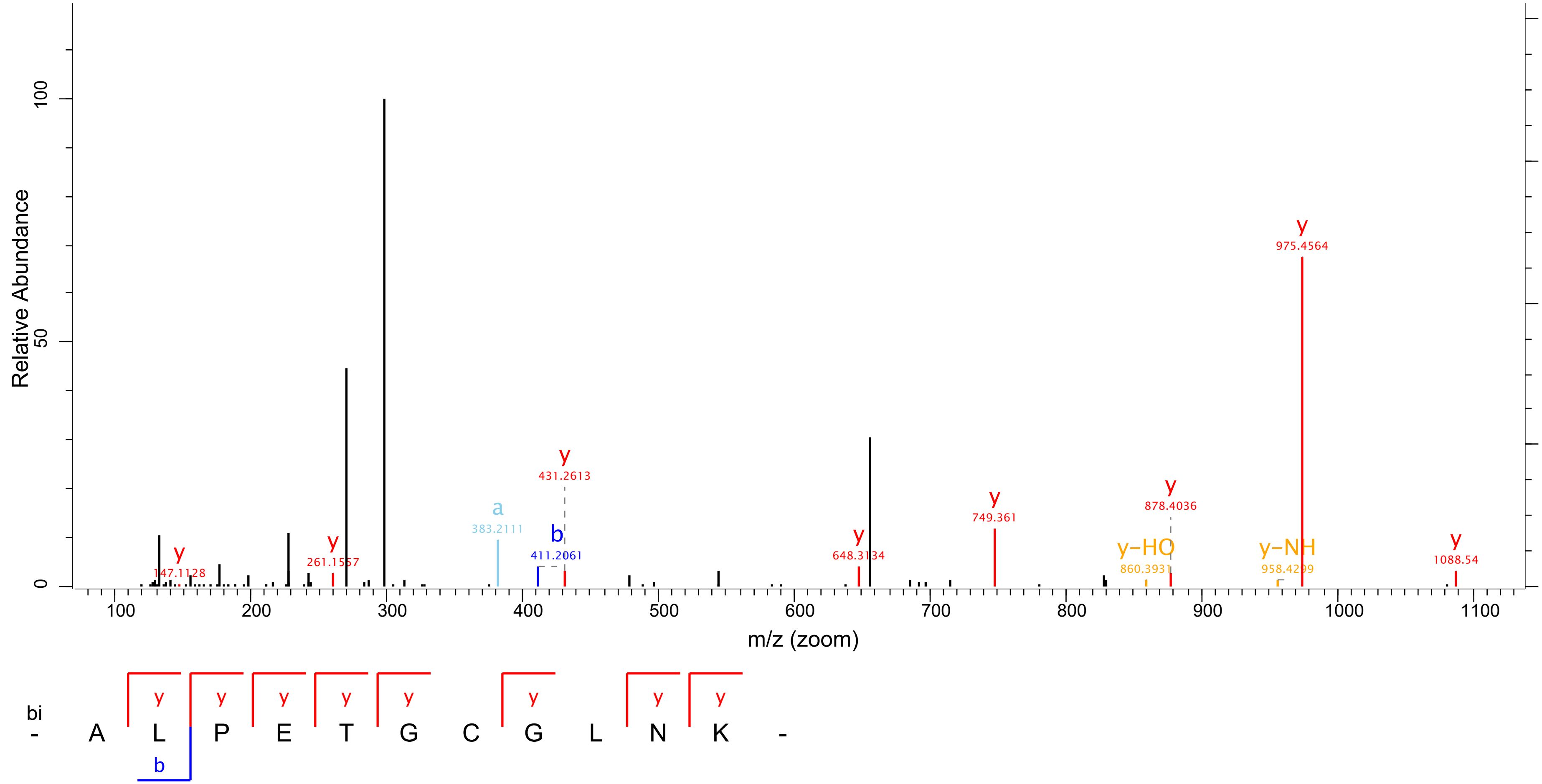

Raw File	Scan	Method	Score	m/z	Gene names
agg_pep_sort_pos_2	14730	FTMS; HCD	96.14	727.4	RIOK2

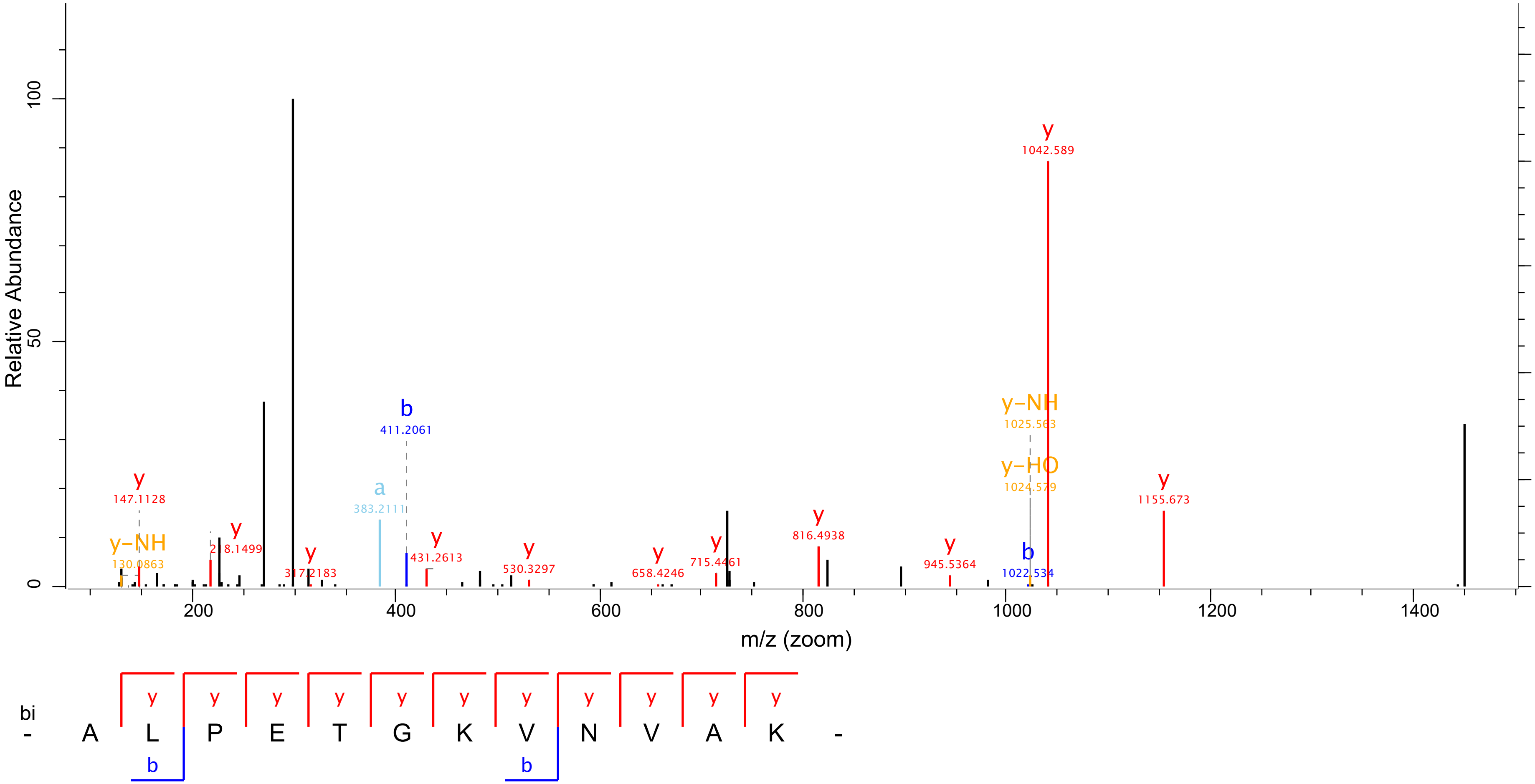

Raw File	Scan	Method	Score	m/z	Gene names
agg_pep_sort_pos_1	9890	FTMS; HCD	68.89	483.93	RPL10L;RPL10

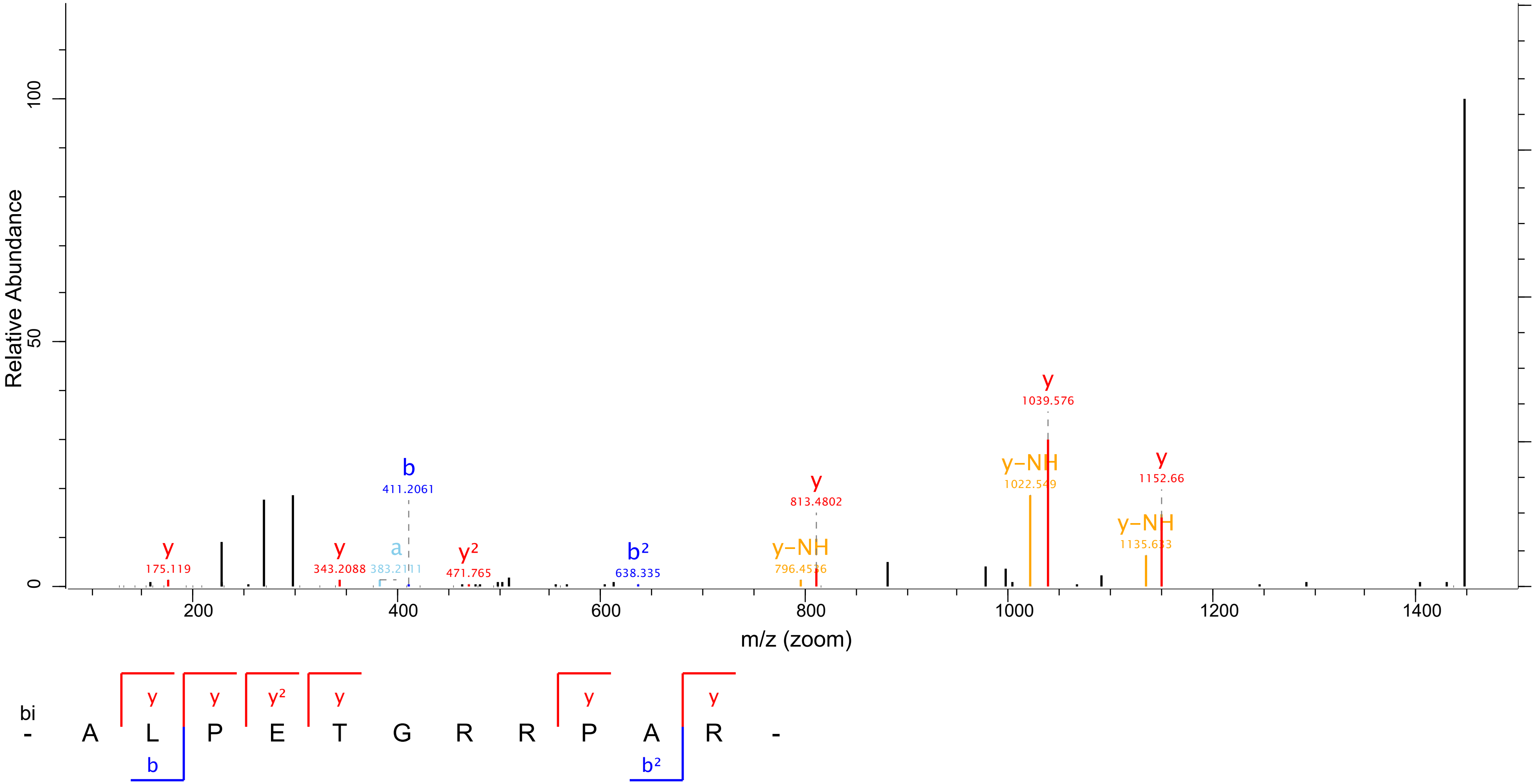

Raw File	Scan	Method	Score	m/z	Gene names
agg_pep_sort_pos_3	22319	FTMS; HCD	76.83	648.84	RPL18

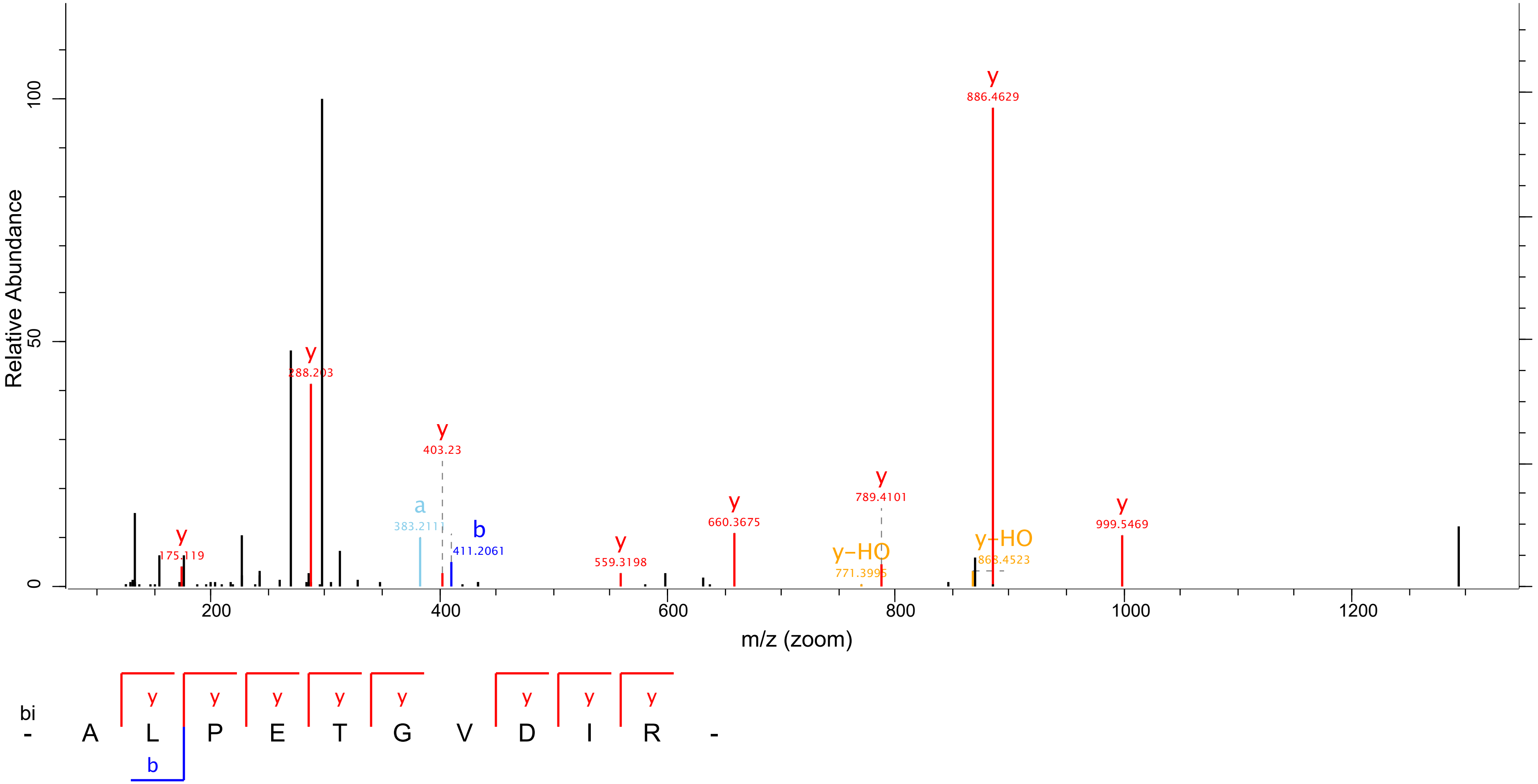

Raw File	Scan	Method	Score	m/z	Gene names
agg_pep_sort_pos_2	22697	FTMS; HCD	95.53	594.31	RPL5

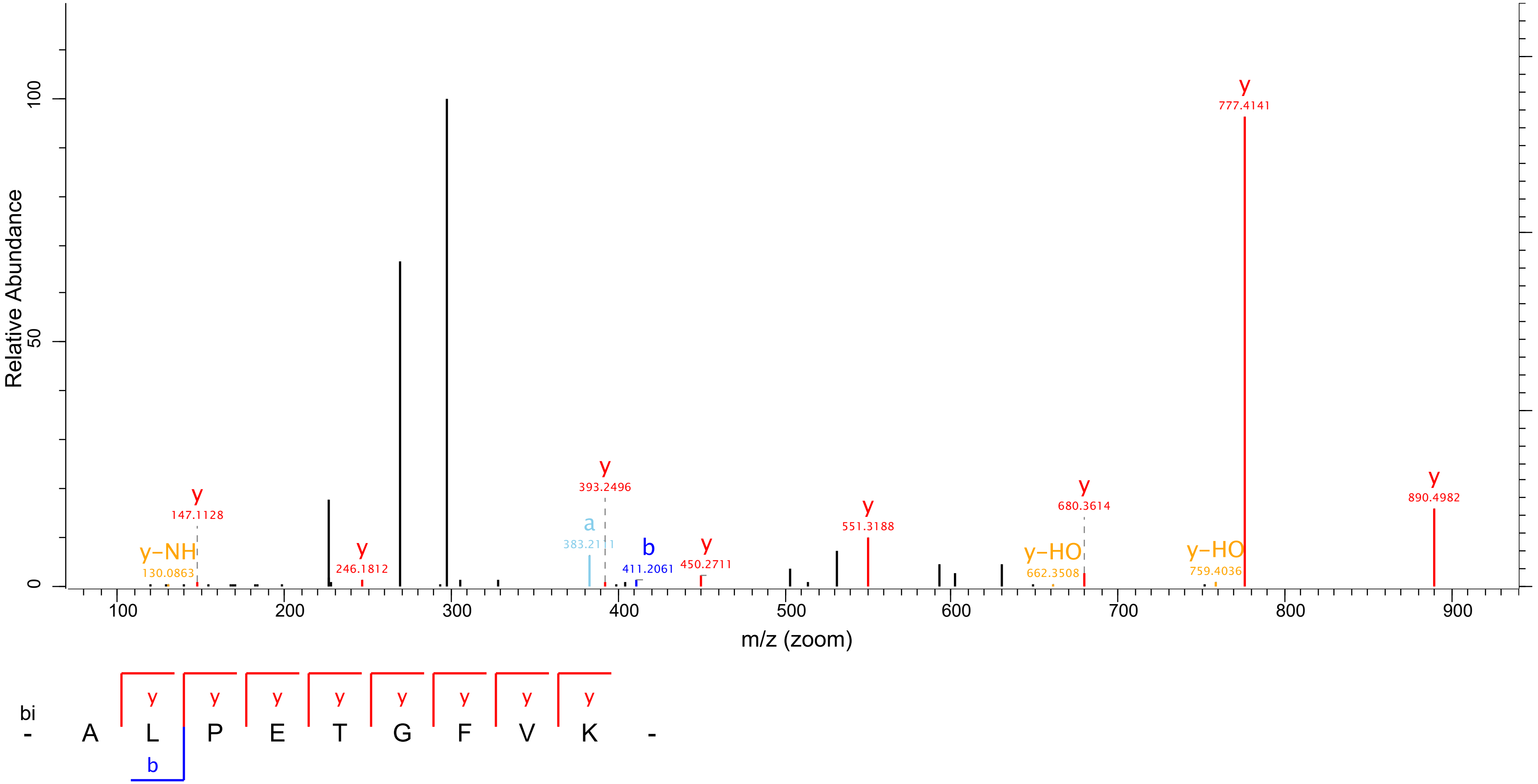

Raw File	Scan	Method	Score	m/z	Gene names
agg_pep_sort_pos_3	12082	FTMS; HCD	109.86	629.31	RPS23

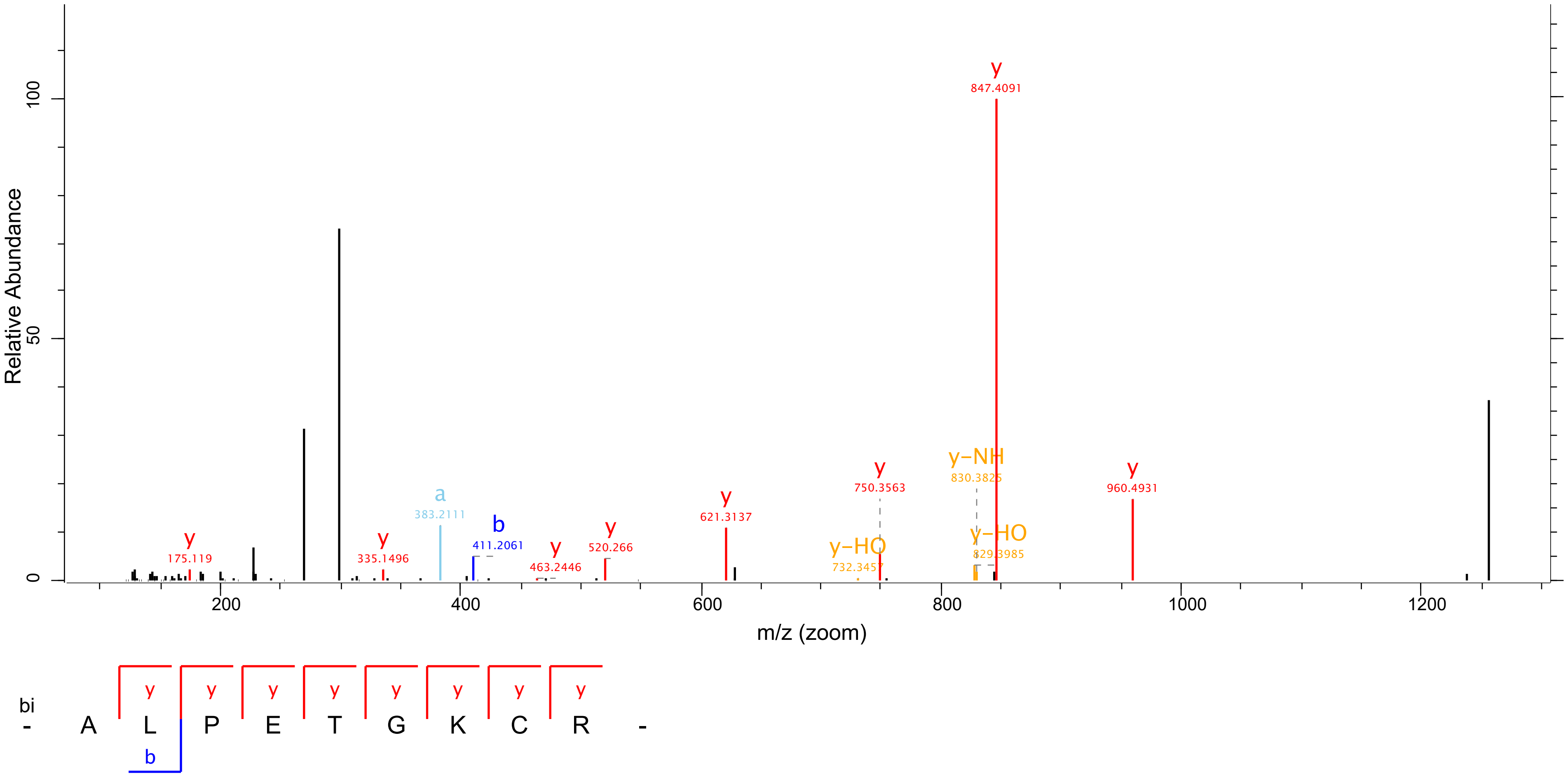

Raw File	Scan	Method	Score	m/z	Gene names
agg_pep_sort_pos_2	17042	FTMS; HCD	128.9	716.35	RPS29

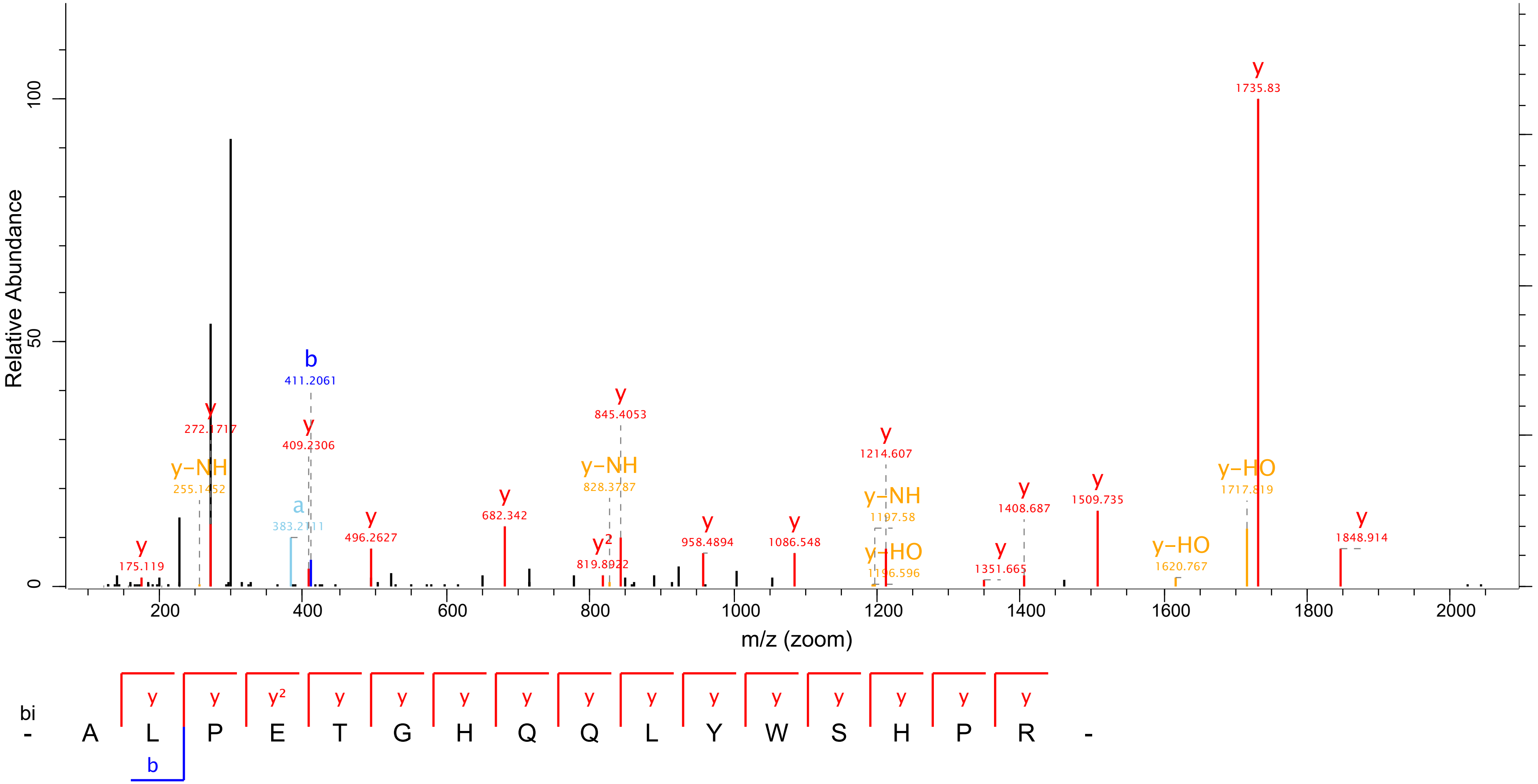

Raw File	Scan	Method	Score	m/z	Gene names
agg_pep_sort_pos_2	15731	FTMS; HCD	80.76	899.43	RTFDC1

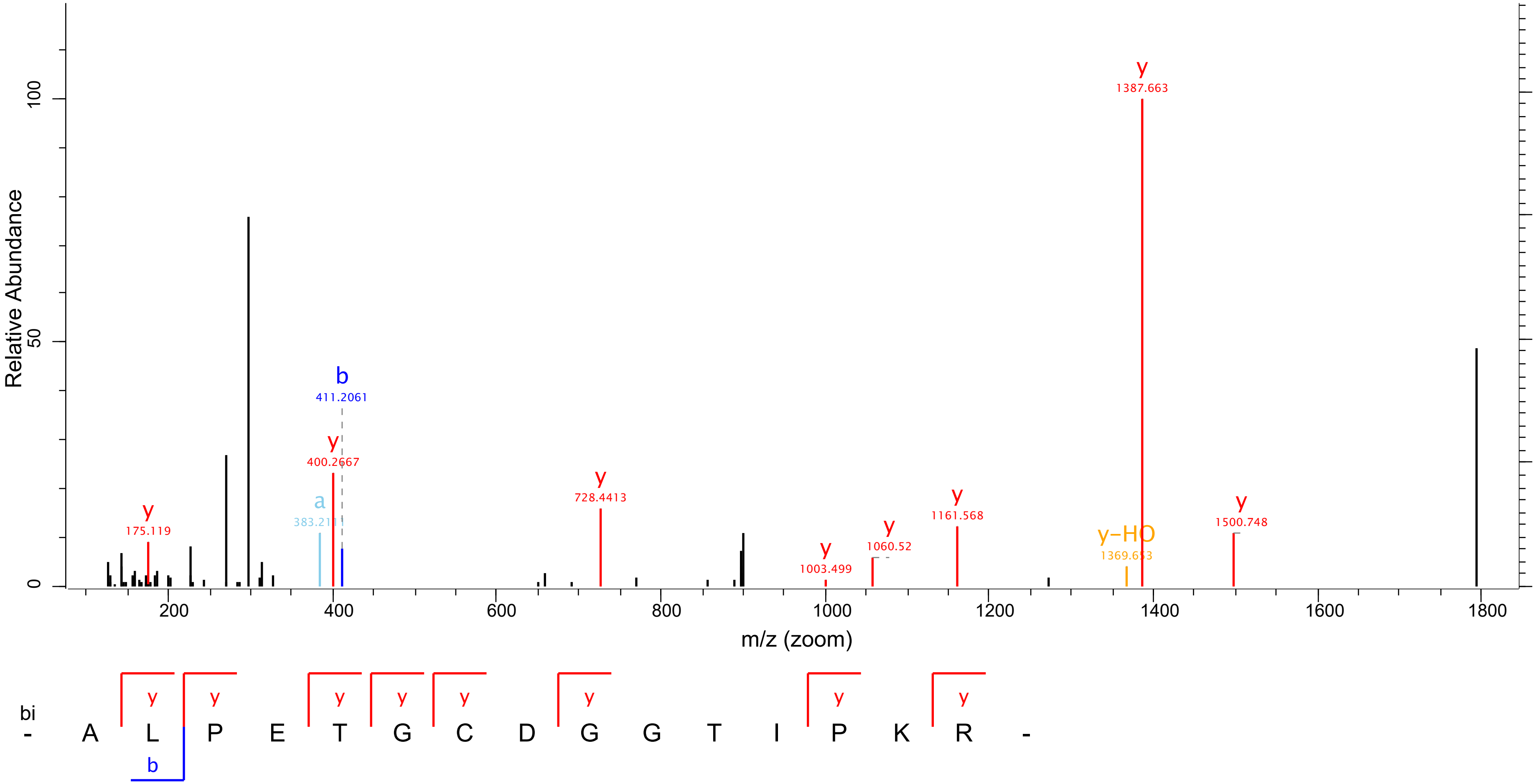

Raw File	Scan	Method	Score	m/z	Gene names
agg_pep_sort_pos_2	20000	FTMS; HCD	113.63	821.38	RTFDC1

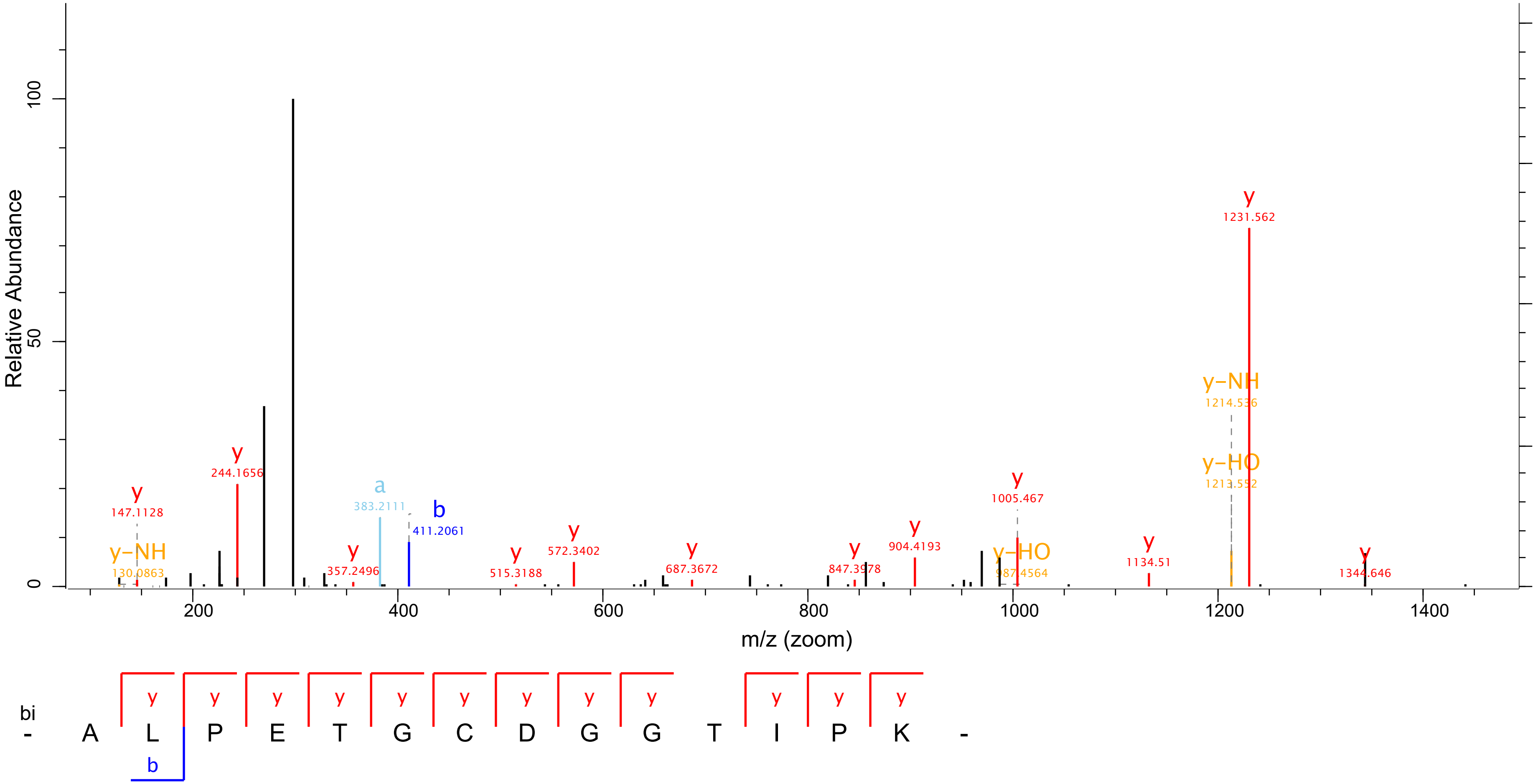

Raw File	Scan	Method	Score	m/z	Gene names
agg_pep_sort_pos_1	16795	FTMS; HCD	76.83	735.38	S100A8

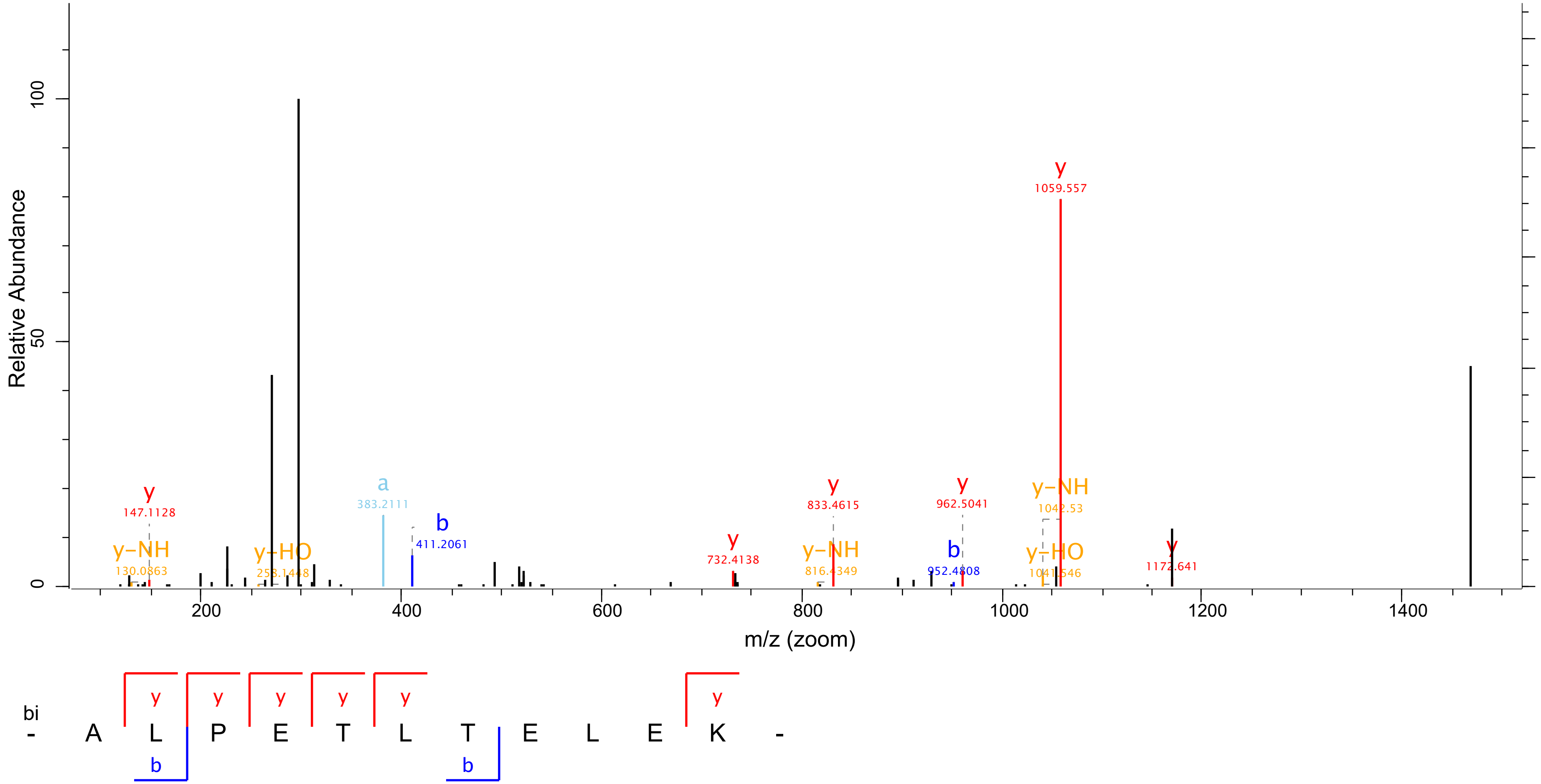

Raw File	Scan	Method	Score	m/z	Gene names
agg_pep_sort_pos_2	13126	FTMS; HCD	48.66	945.11	SCML2

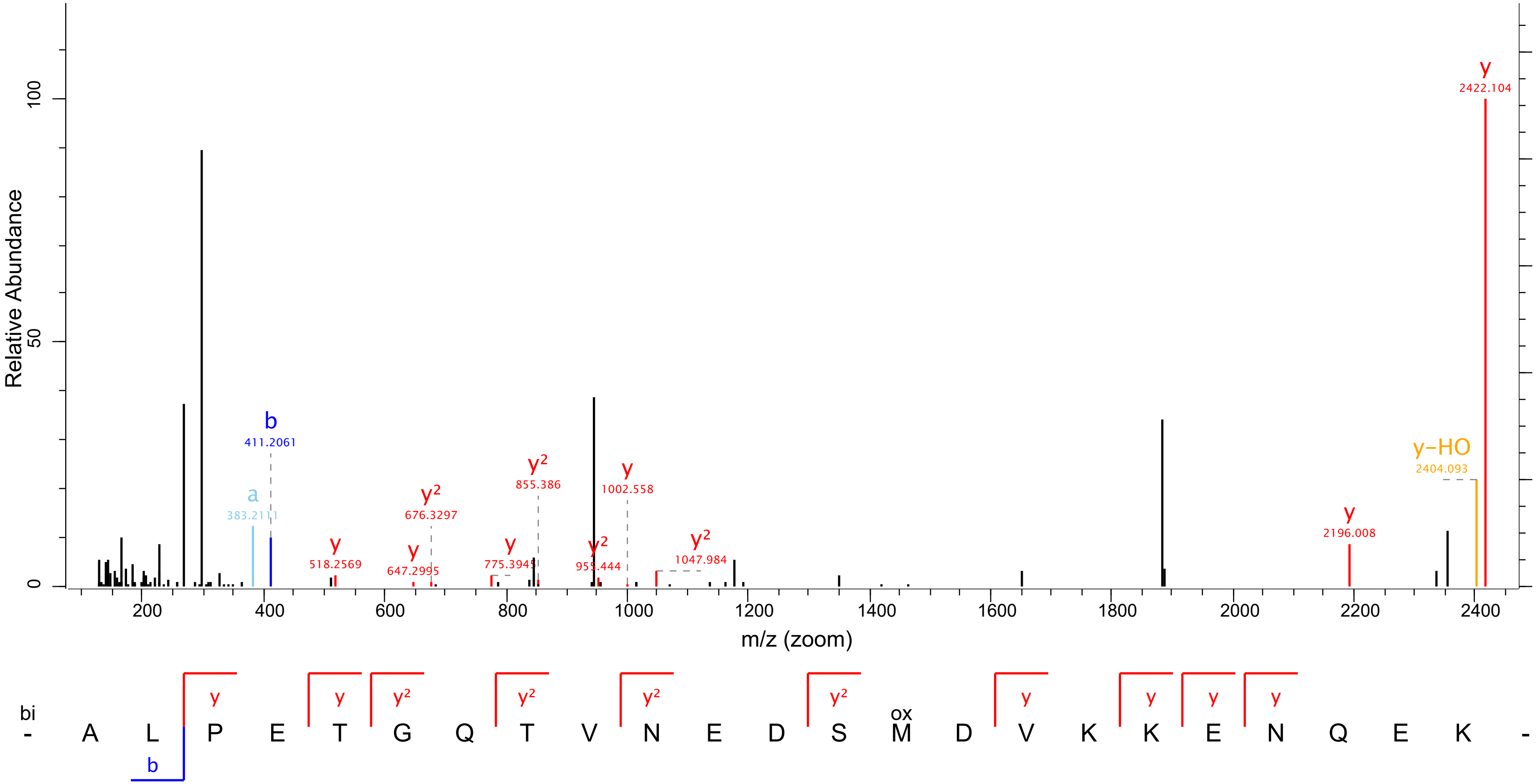

Raw File	Scan	Method	Score	m/z	Gene names
agg_pep_sort_pos_2	14590	FTMS; HCD	77.85	939.77	SCML2

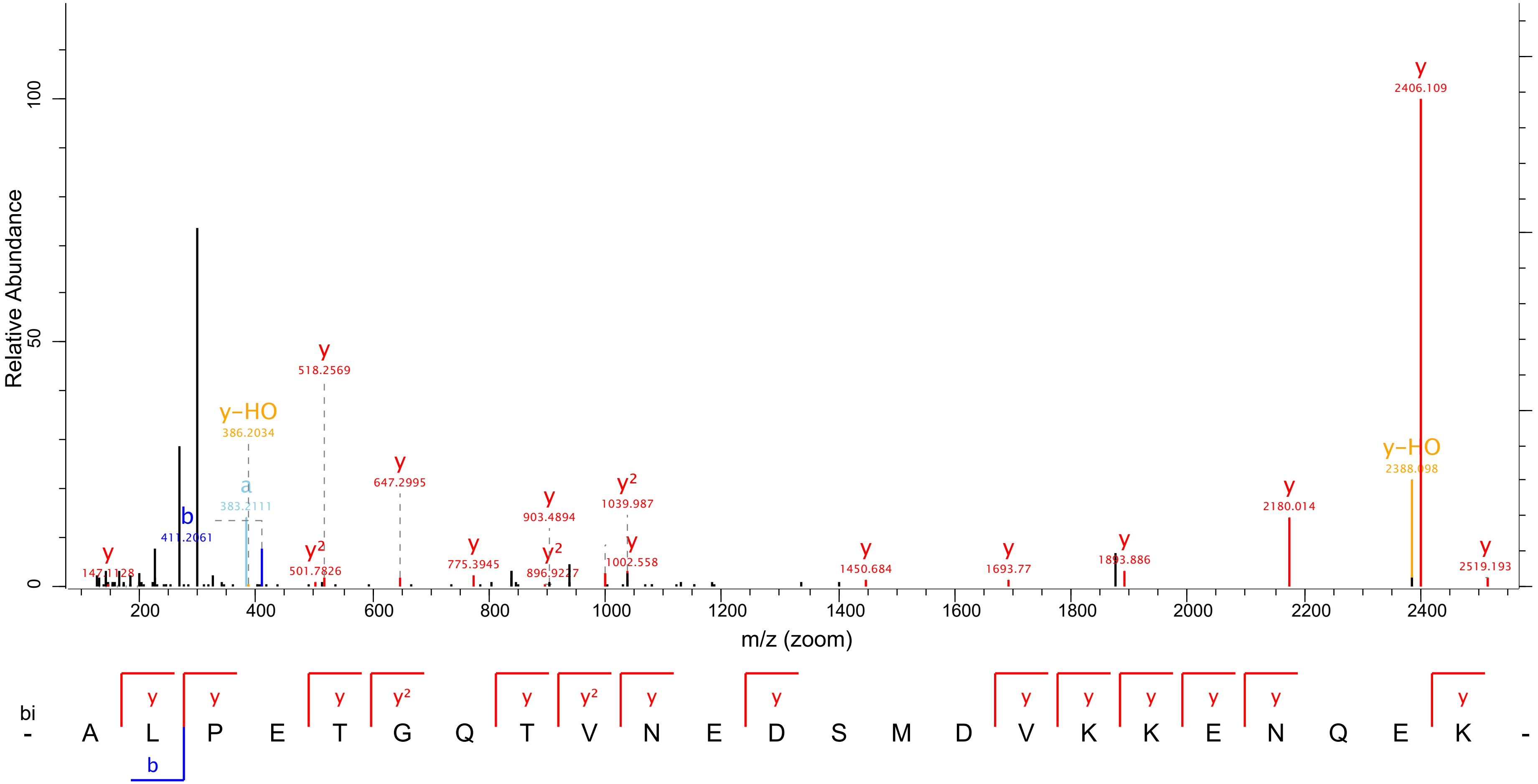

Raw File	Scan	Method	Score	m/z	Gene names
agg_pep_sort_pos_2	15363	FTMS; HCD	26.84	736.01	SCML2

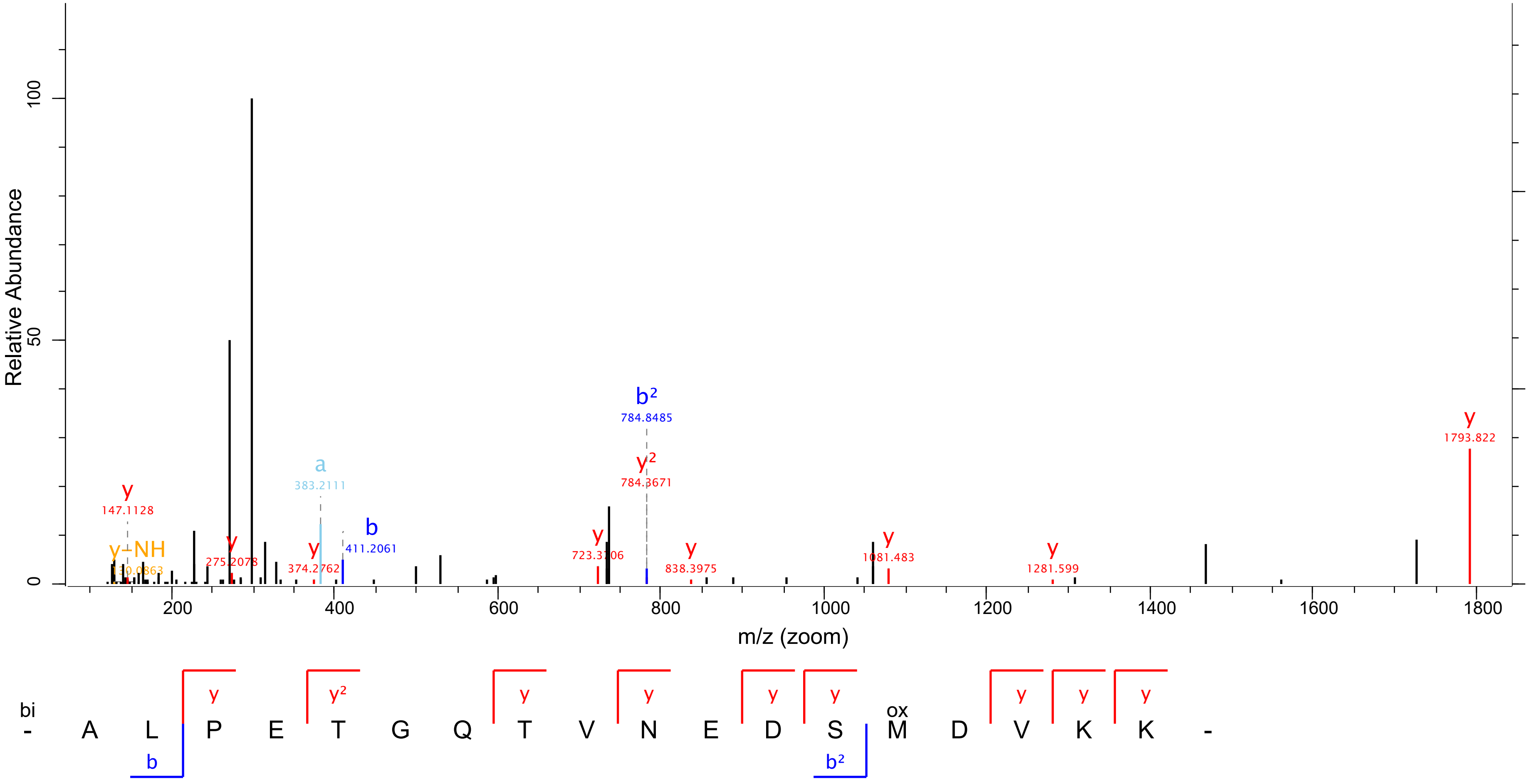

Raw File	Scan	Method	Score	m/z	Gene names
agg_pep_sort_pos_3	17179	FTMS; HCD	62.77	730.35	SCML2

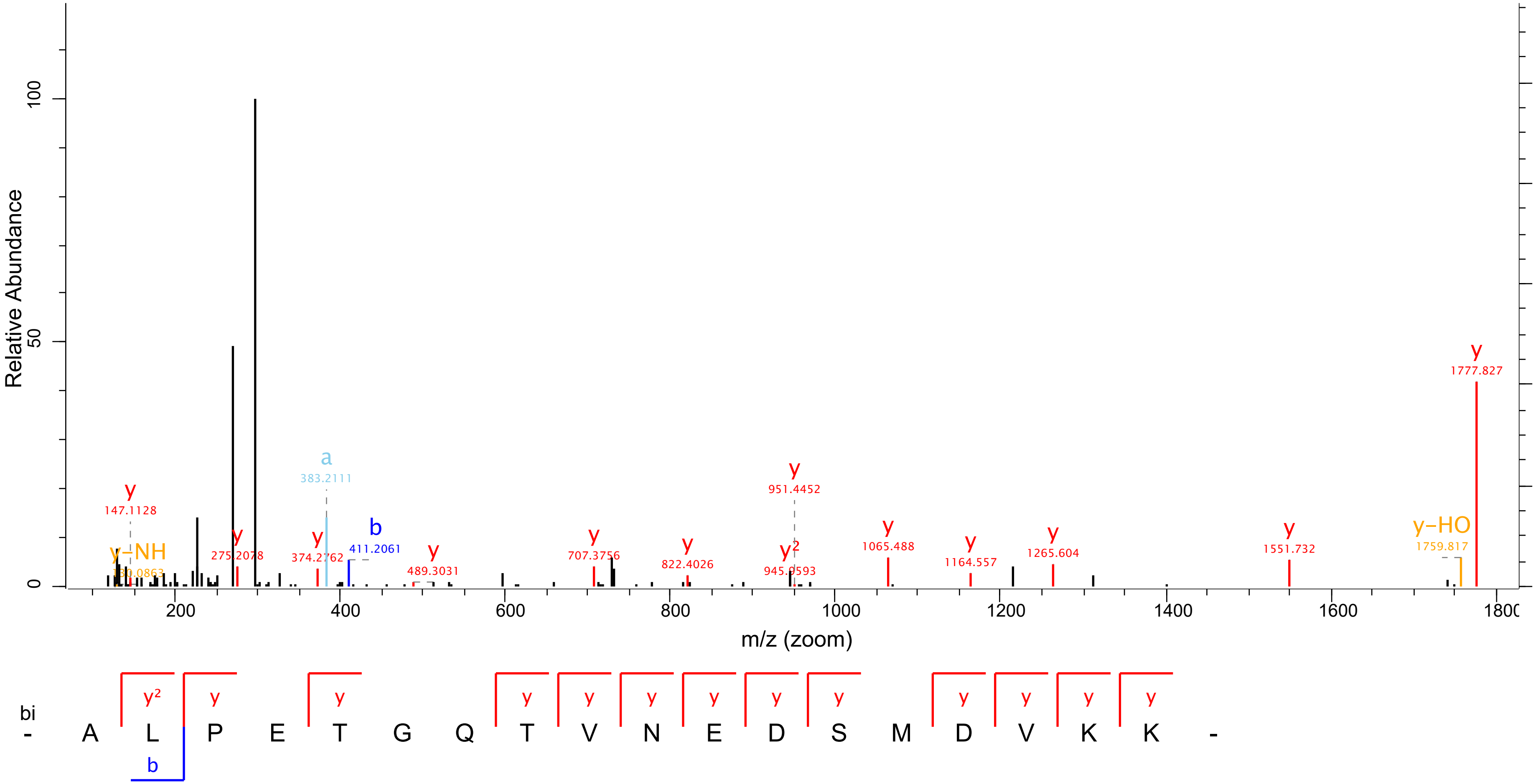

Raw File	Scan	Method	Score	m/z	Gene names
agg_pep_sort_pos_1	19823	FTMS; HCD	57.33	681.33	SDR9C7

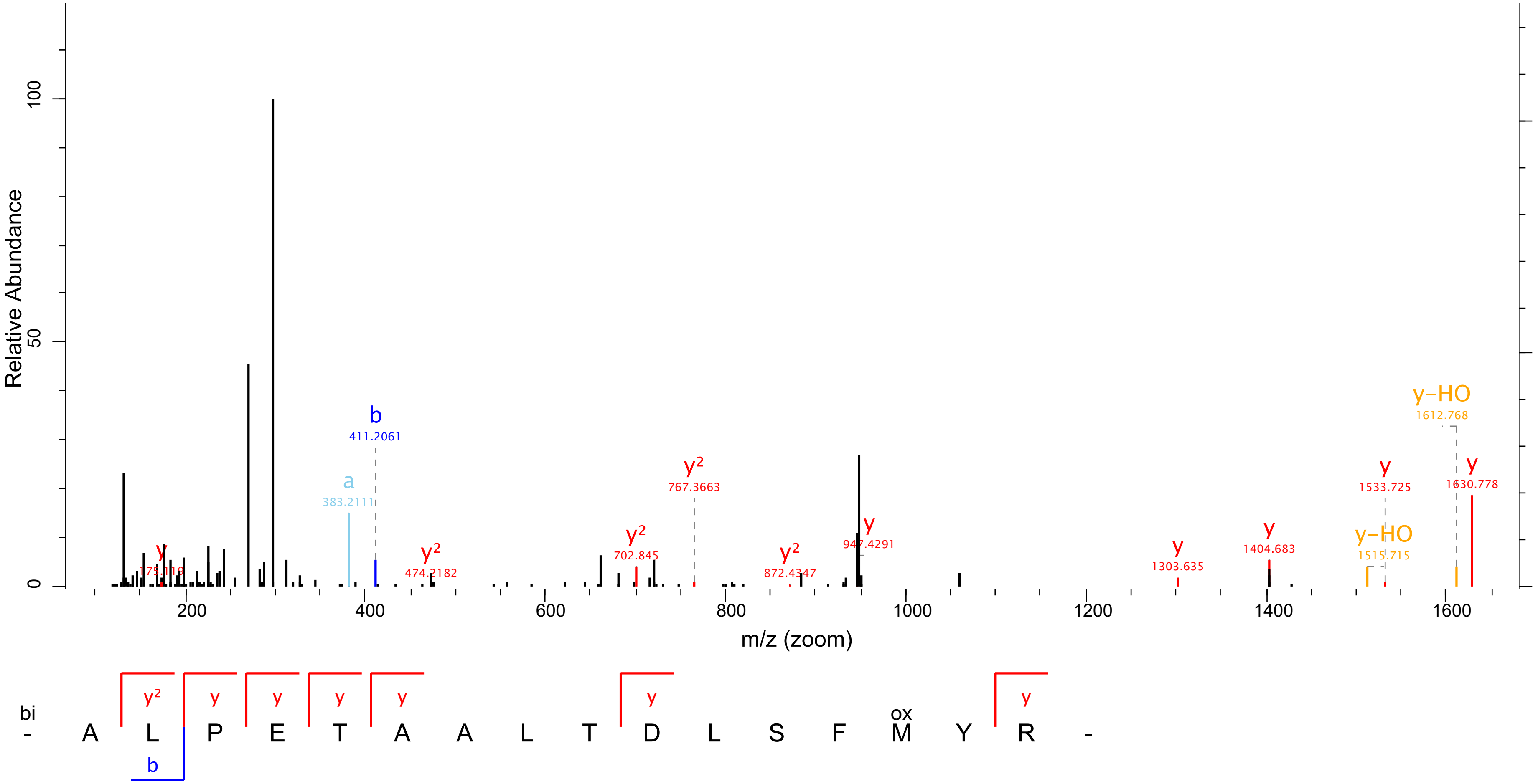

Raw File	Scan	Method	Score	m/z	Gene names
agg_pep_sort_pos_1	22938	FTMS; HCD	66.62	655.35	SEPT11

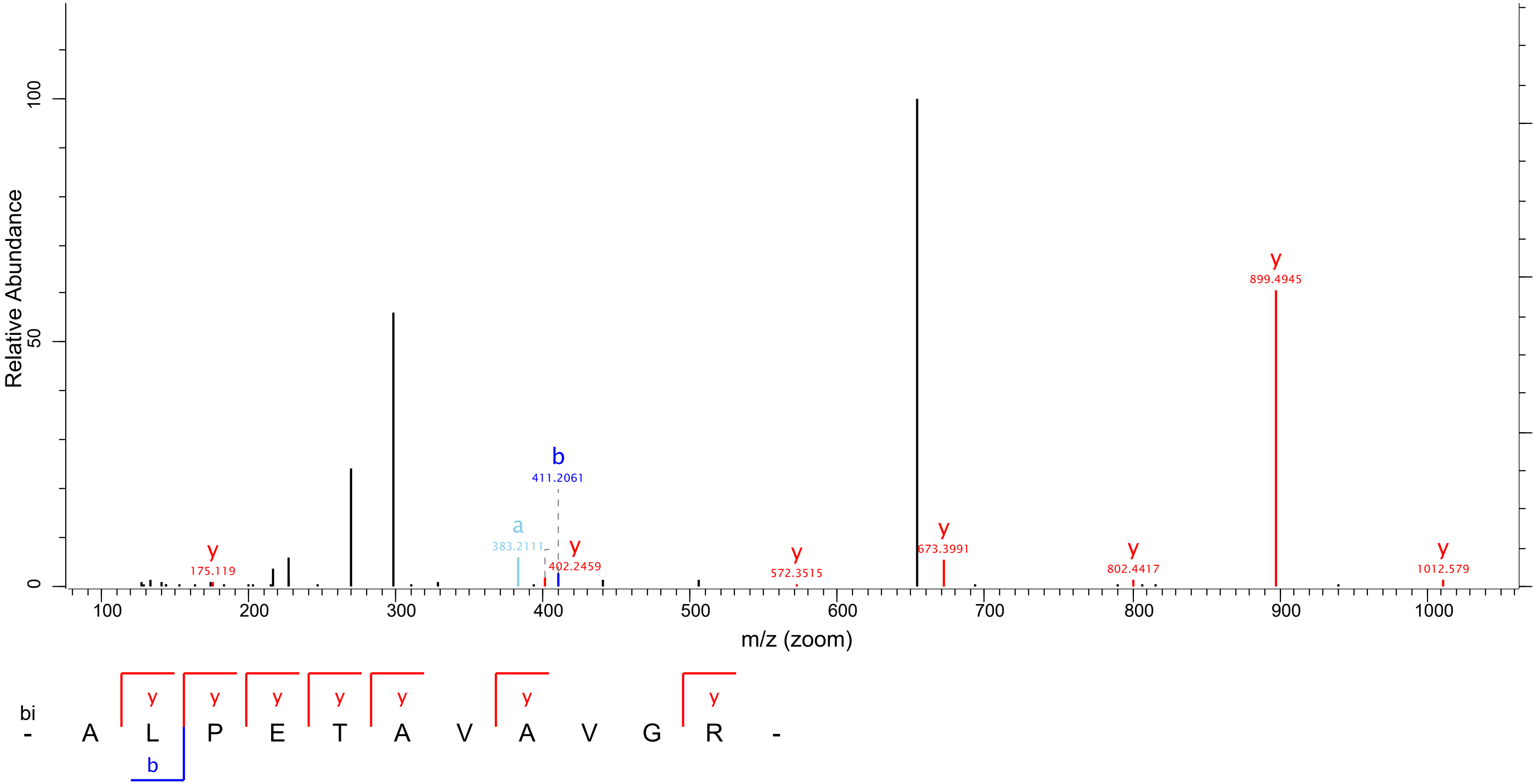

Raw File	Scan	Method	Score	m/z	Gene names
agg_pep_sort_pos_1	15979	FTMS; HCD	94.3	799.91	SH3KBP1

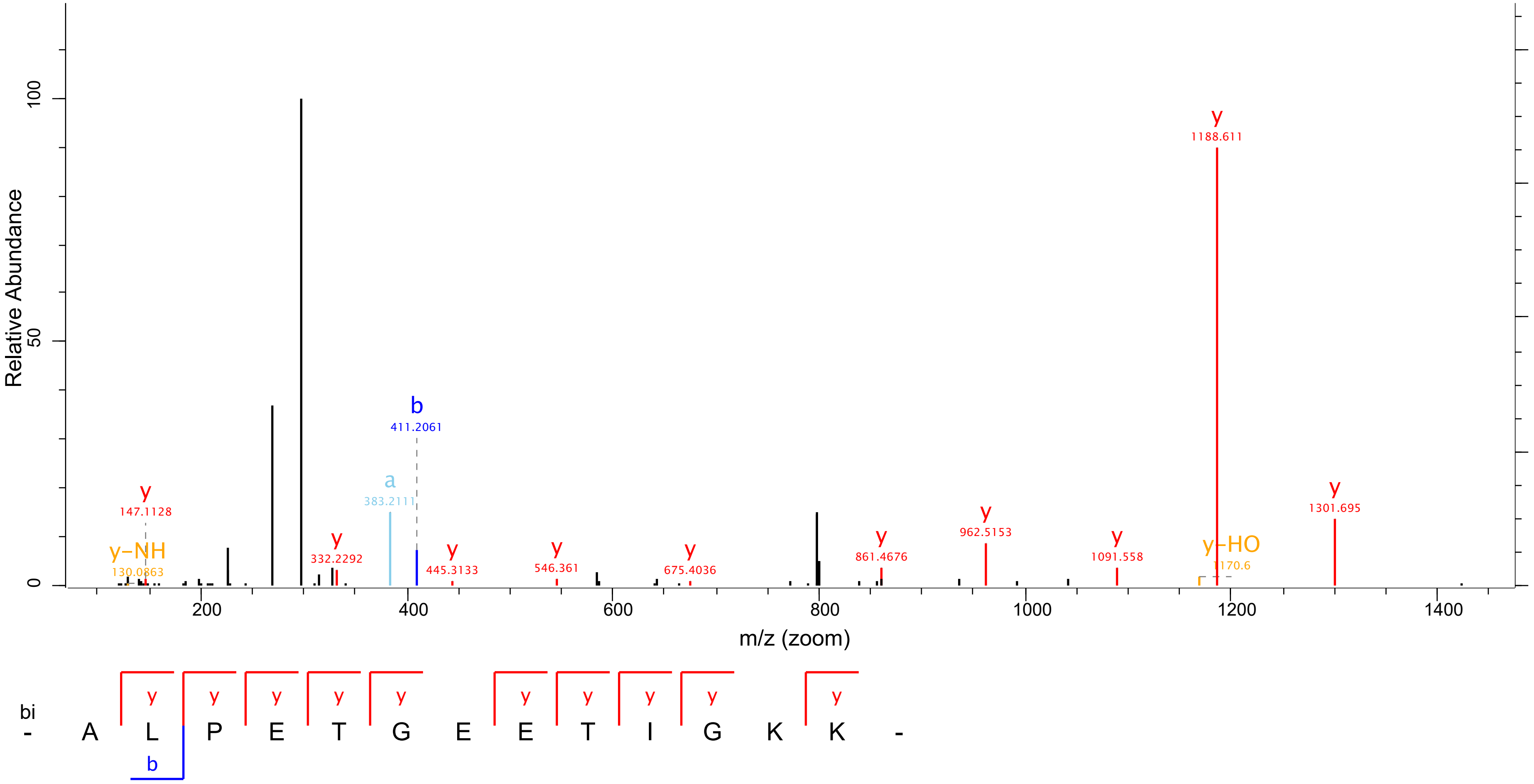

Raw File	Scan	Method	Score	m/z	Gene names
agg_pep_sort_pos_2	21675	FTMS; HCD	80.52	962.48	SLC16A3

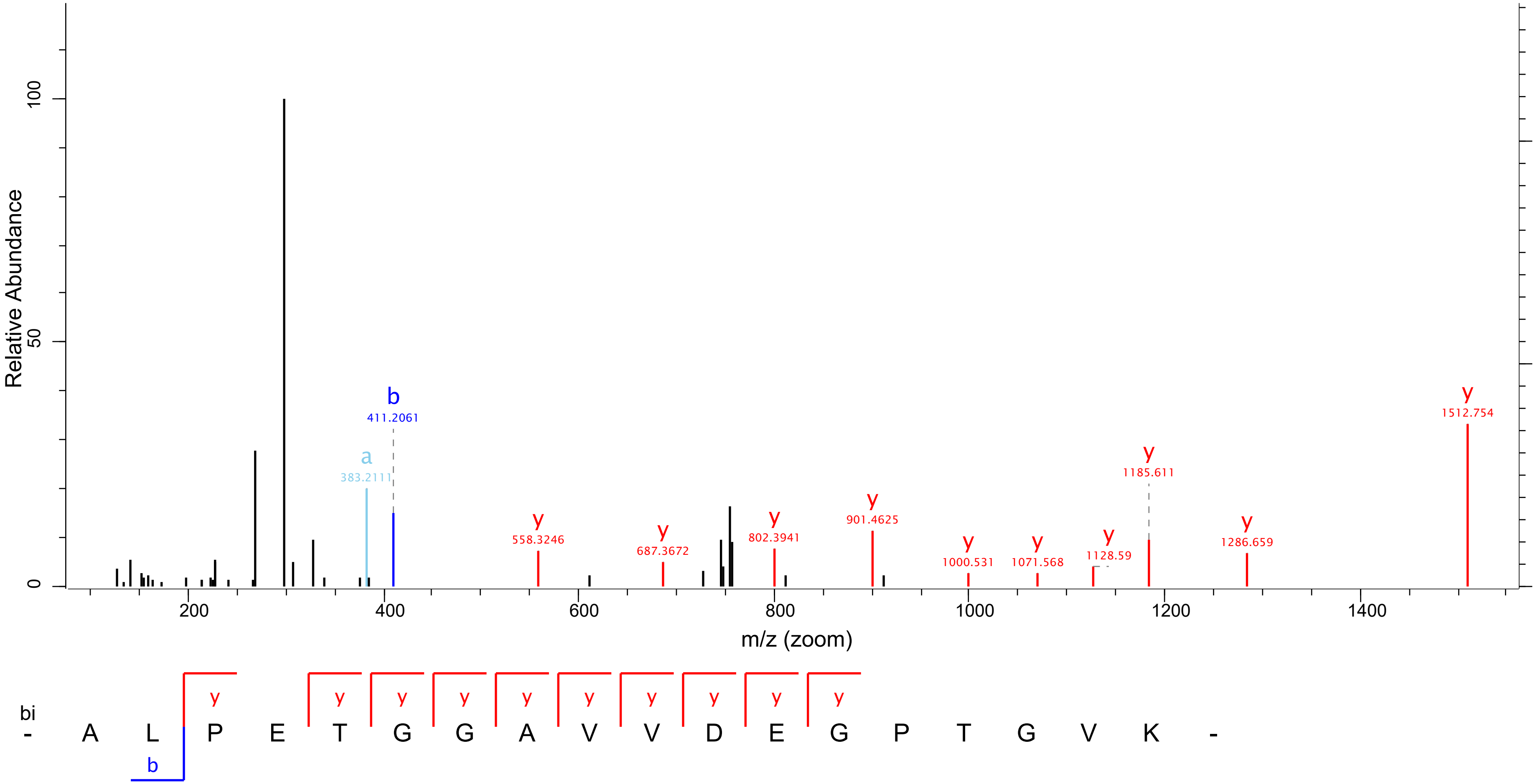

Raw File	Scan	Method	Score	m/z	Gene names
agg_pep_sort_pos_3	12803	FTMS; HCD	79.91	633.34	SLC1A1

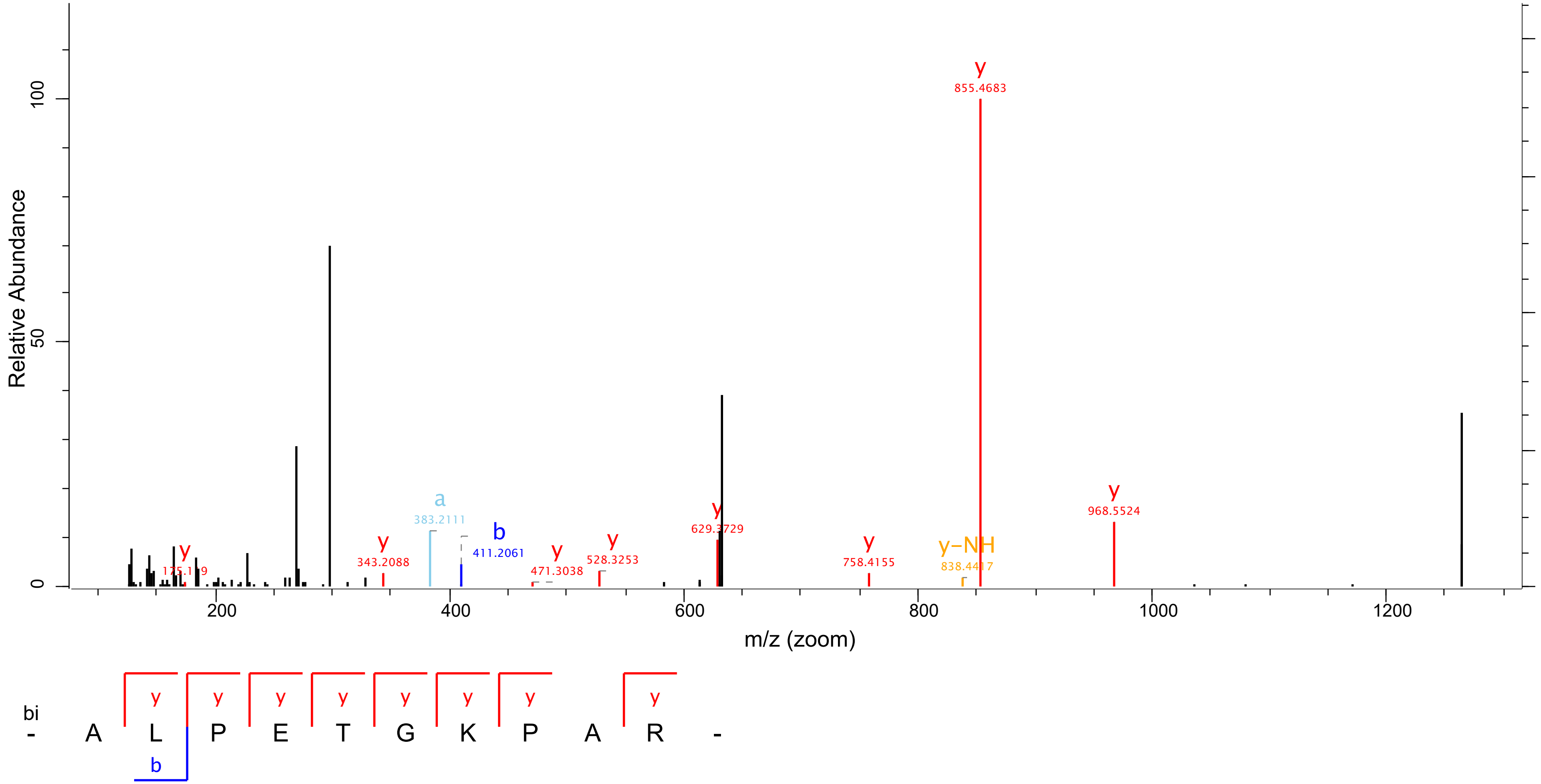

Raw File	Scan	Method	Score	m/z	Gene names
agg_pep_sort_pos_2	13478	FTMS; HCD	53.68	754.39	SLC33A1

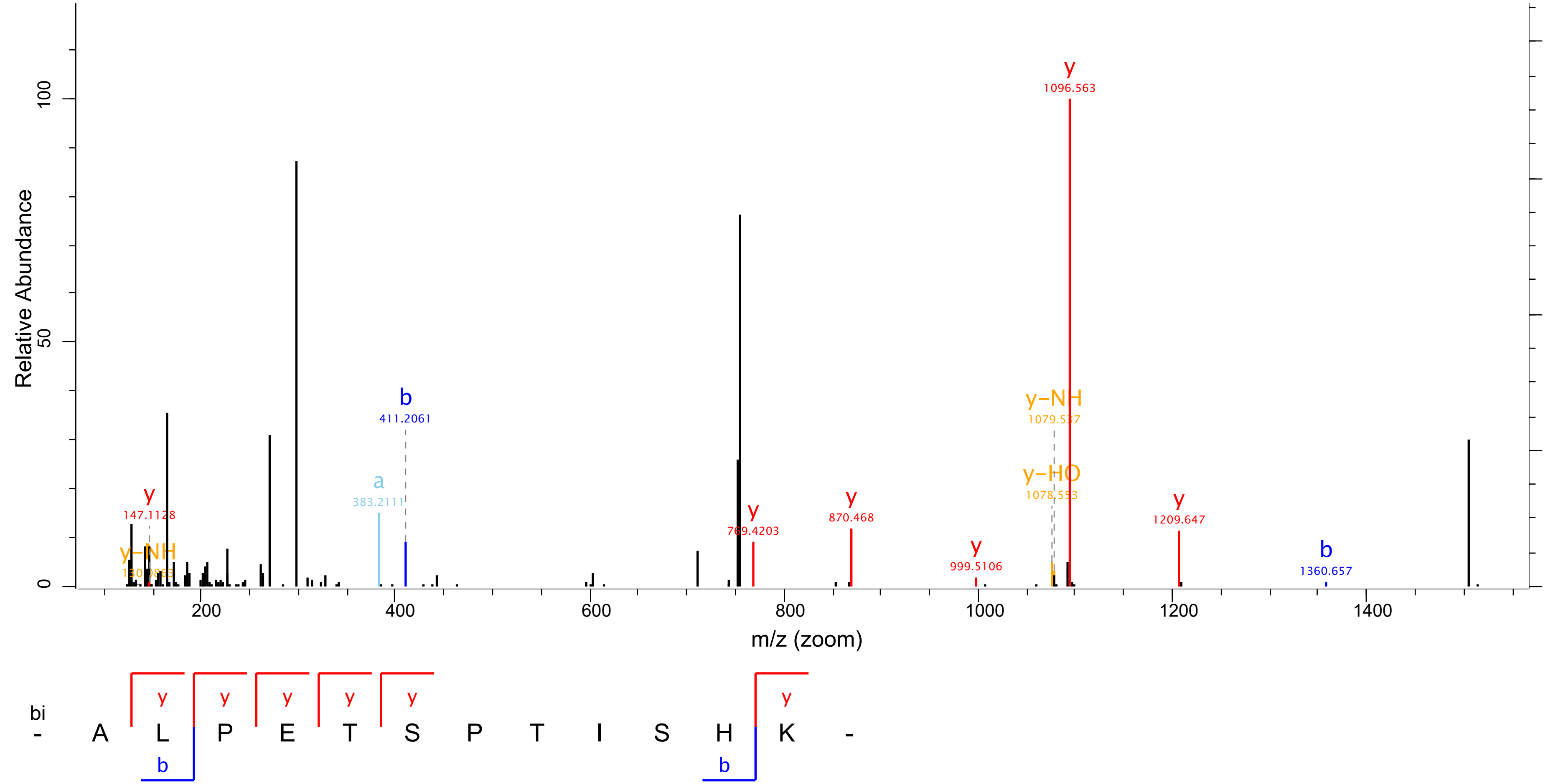

Raw File	Scan	Method	Score	m/z	Gene names
agg_pep_sort_pos_1	11877	FTMS; HCD	58.83	666.35	SLC35D1

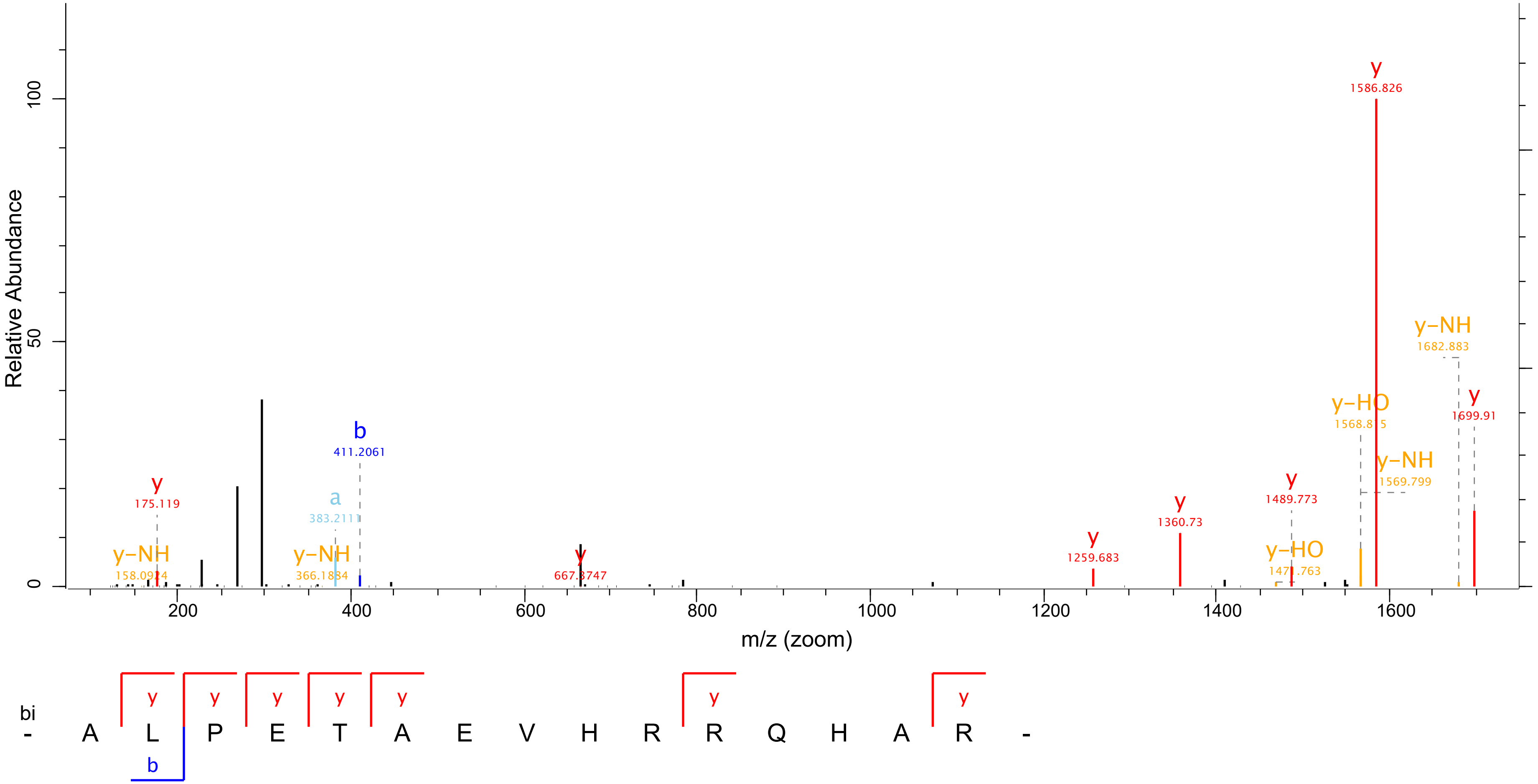

Raw File	Scan	Method	Score	m/z	Gene names
agg_pep_sort_pos_1	24686	FTMS; HCD	105.57	633.35	SLCO2A1

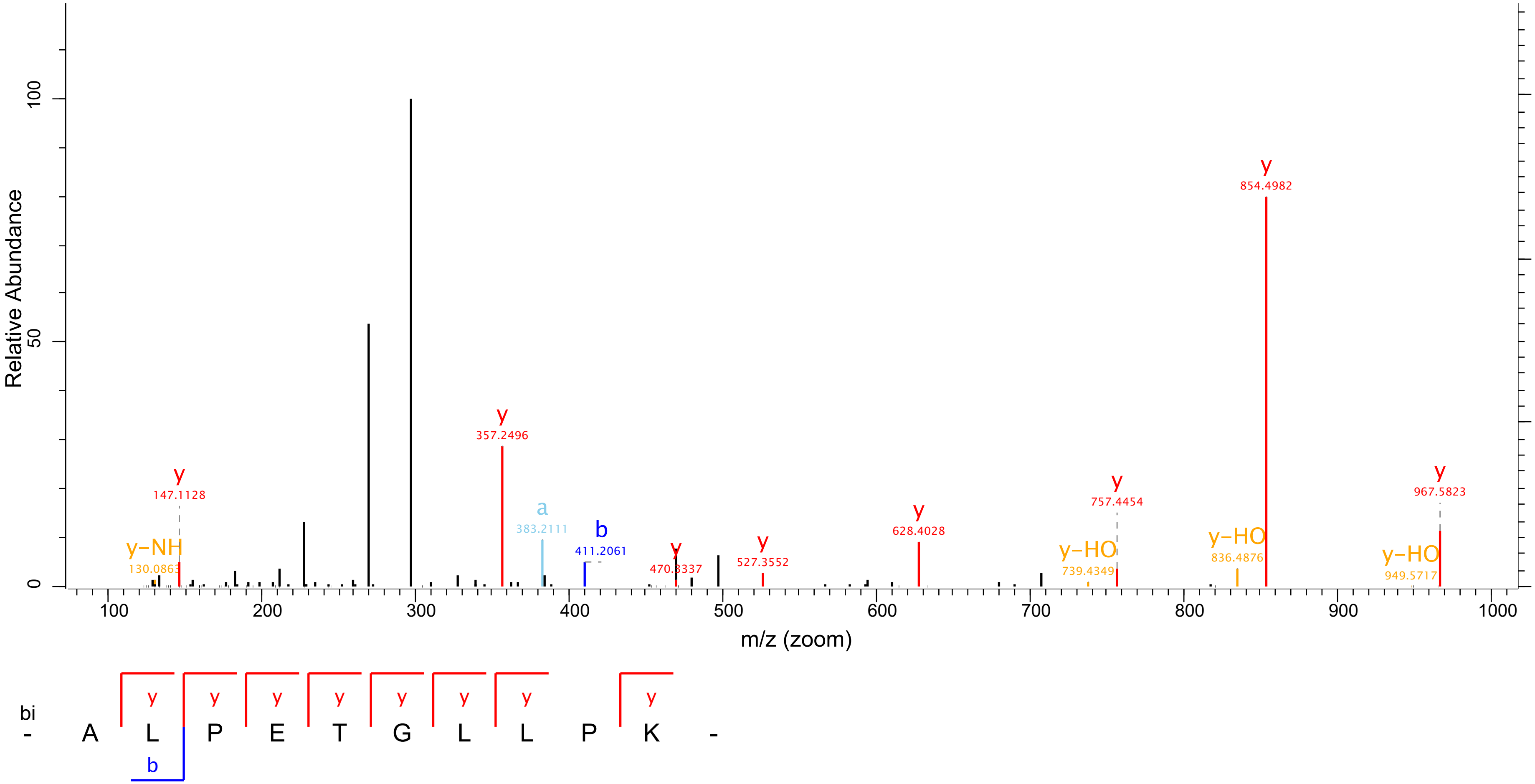

Raw File	Scan	Method	Score	m/z
agg_pep_sort_pos_2	16329	FTMS; HCD	44.57	795.08

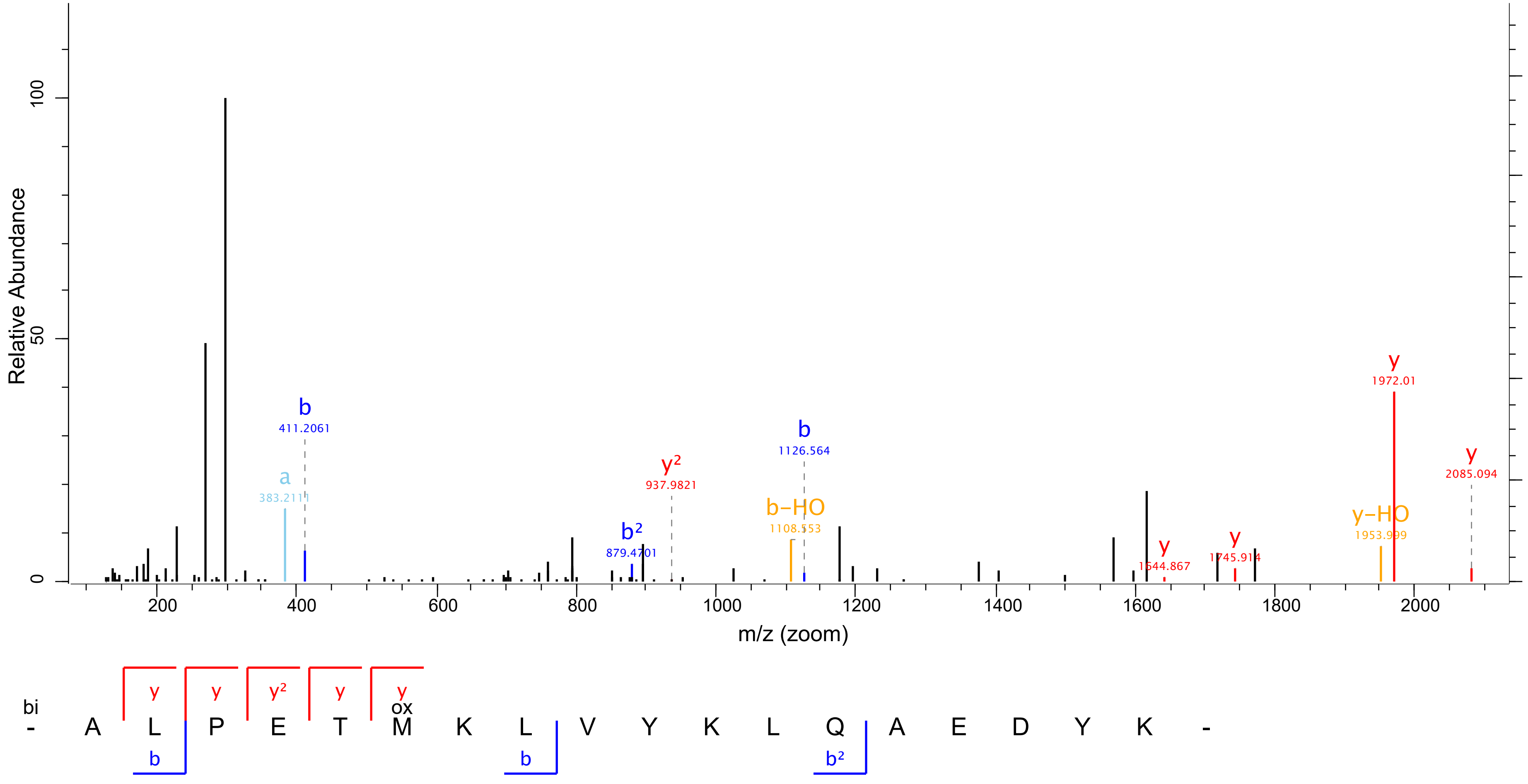

Raw File	Scan	Method	Score	m/z	Gene names
agg_pep_sort_pos_1	17536	FTMS; HCD	147.95	749.87	SPANXB1

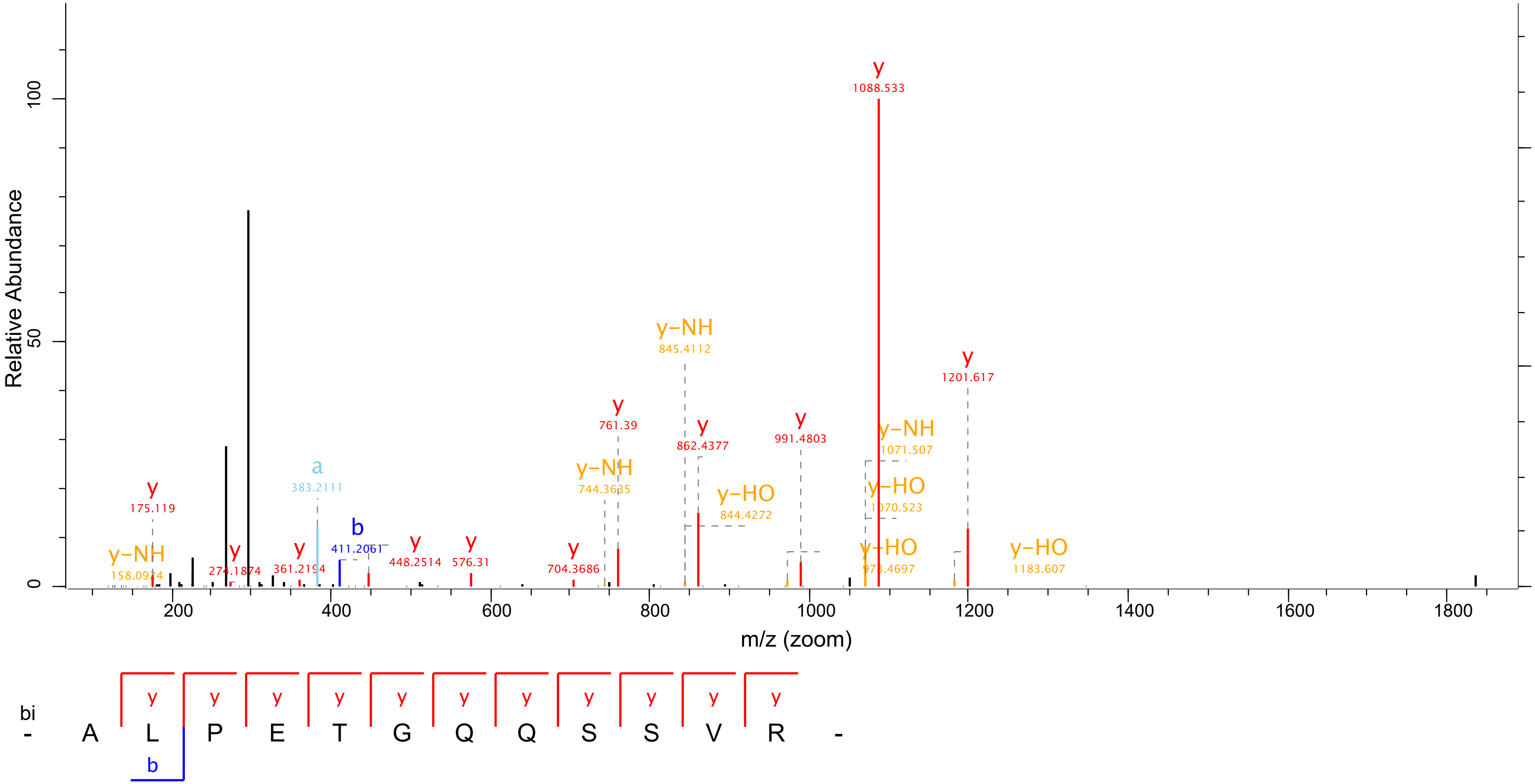

Raw File	Scan	Method	Score	m/z
agg_pep_sort_pos_3	13392	FTMS; HCD	66.38	724.1

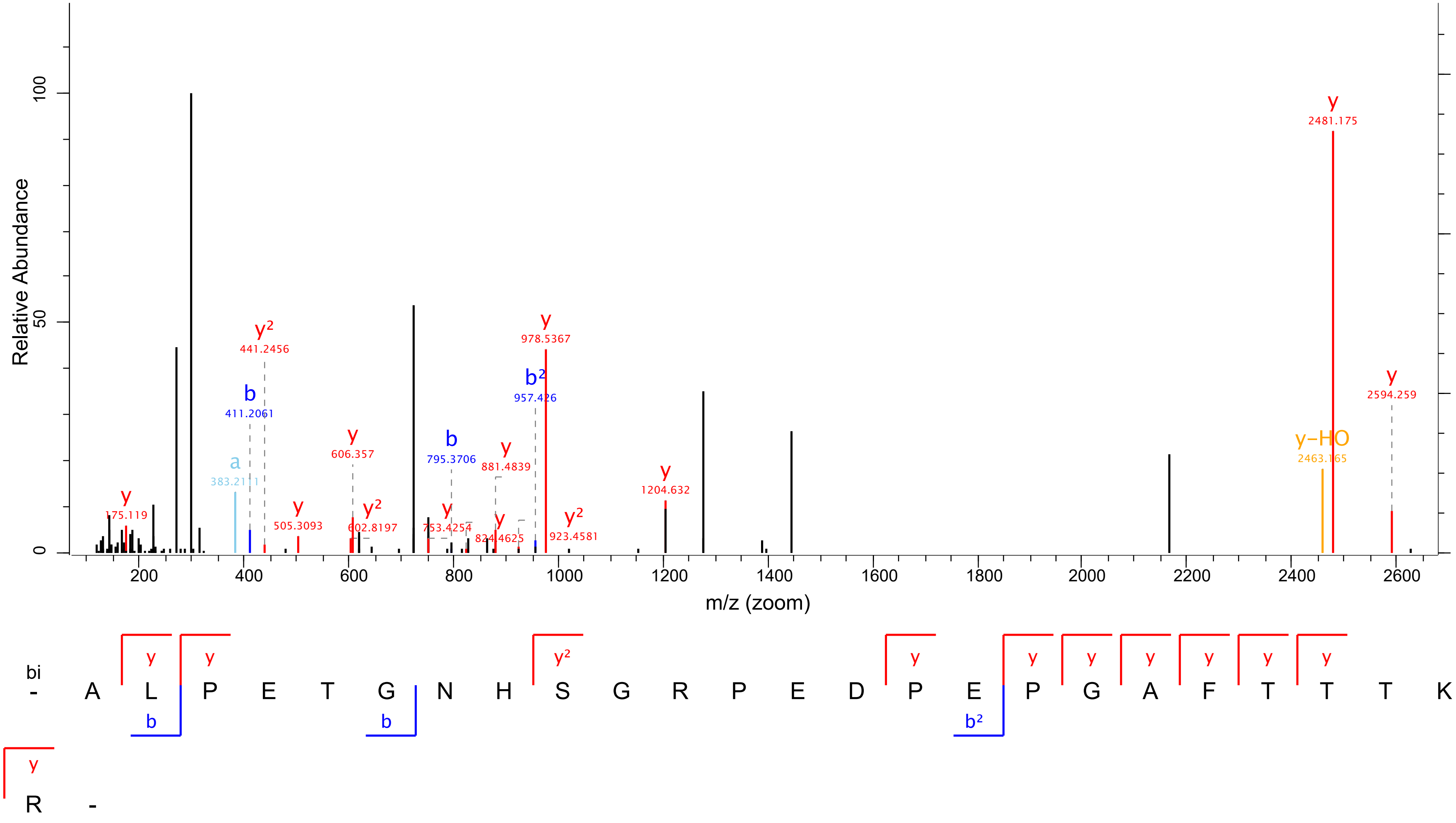

Raw File	Scan	Method	Score	m/z	Gene names
agg_pep_sort_pos_3	15899	FTMS; HCD	122.18	571.78	SRC

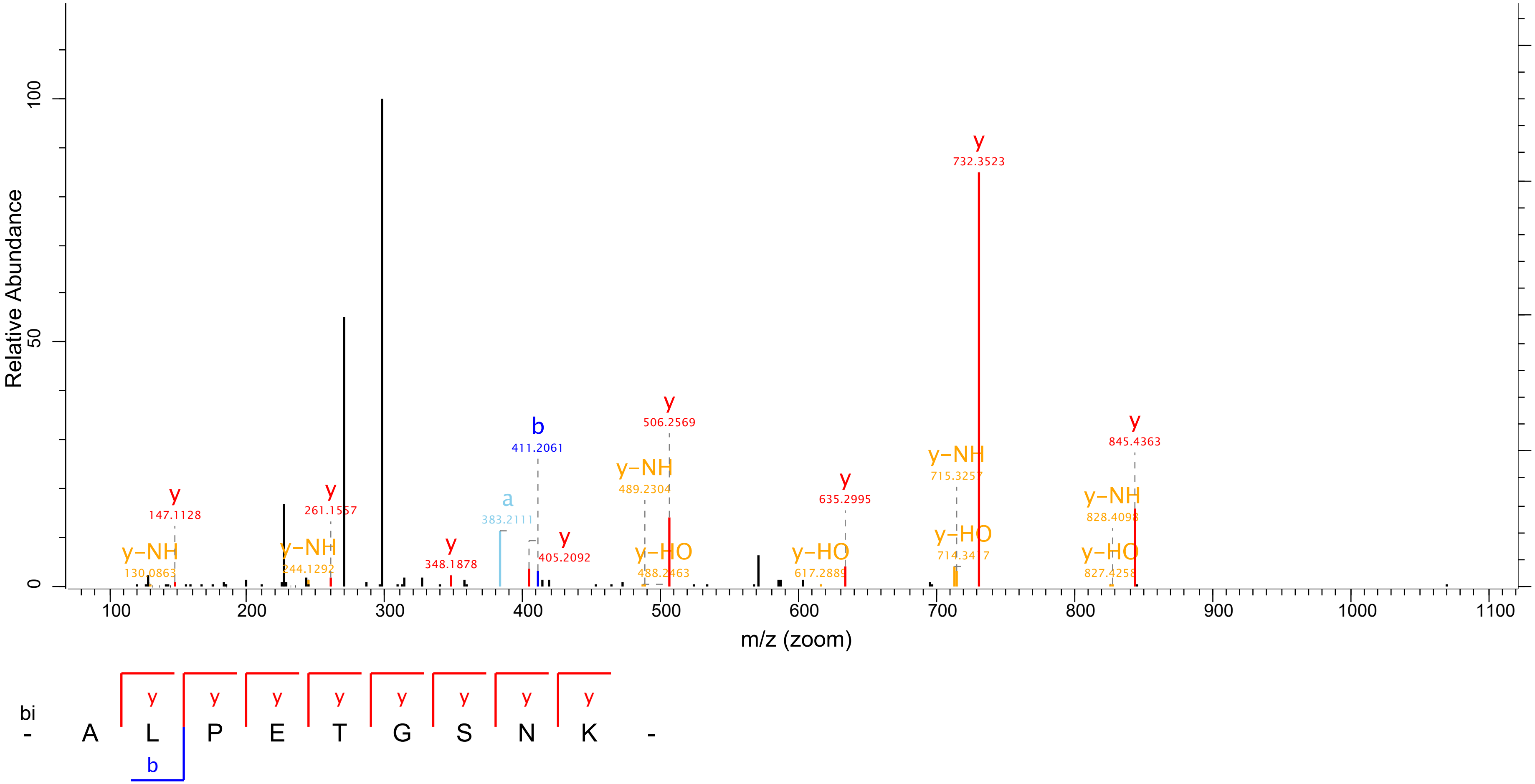

Raw File	Scan	Method	Score	m/z	Gene names
agg_pep_sort_pos_1	19096	FTMS; HCD	54.6	704.36	STX2

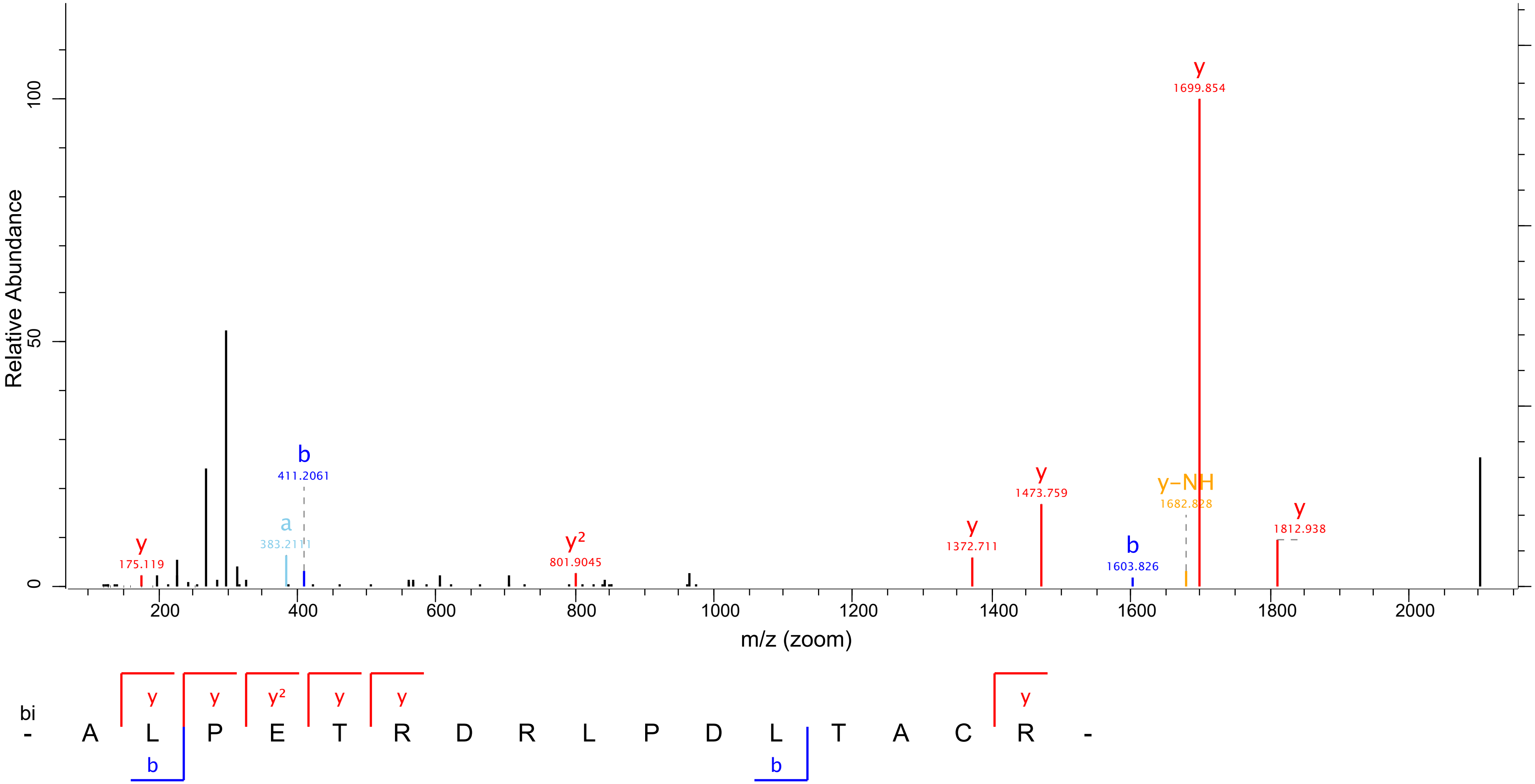

Raw File	Scan	Method	Score	m/z
agg_pep_sort_pos_1	12659	FTMS; HCD	72.29	832.4

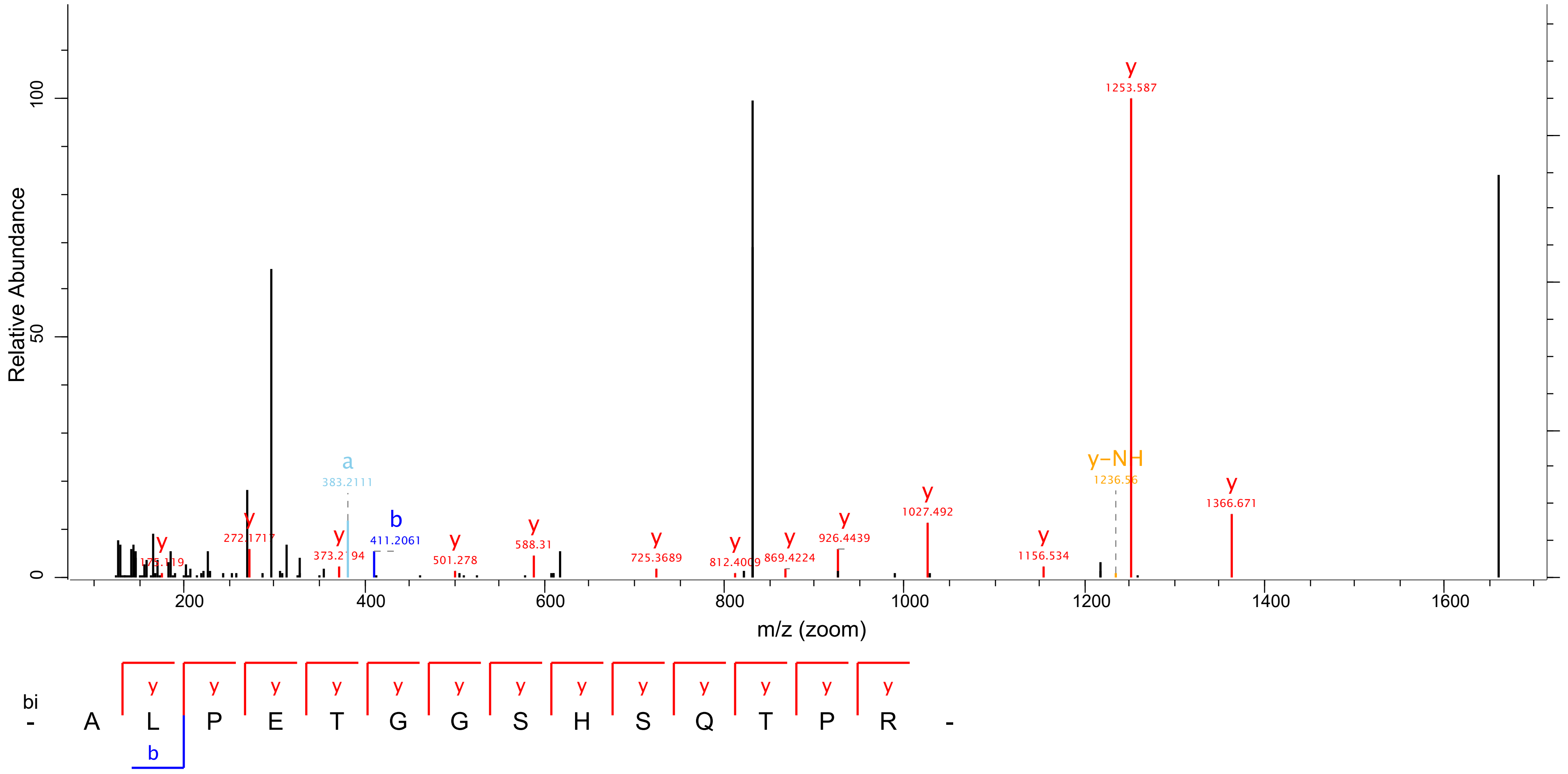

Raw File	Scan	Method	Score	m/z
agg_pep_sort_pos_1	9567	FTMS; HCD	38.38	700

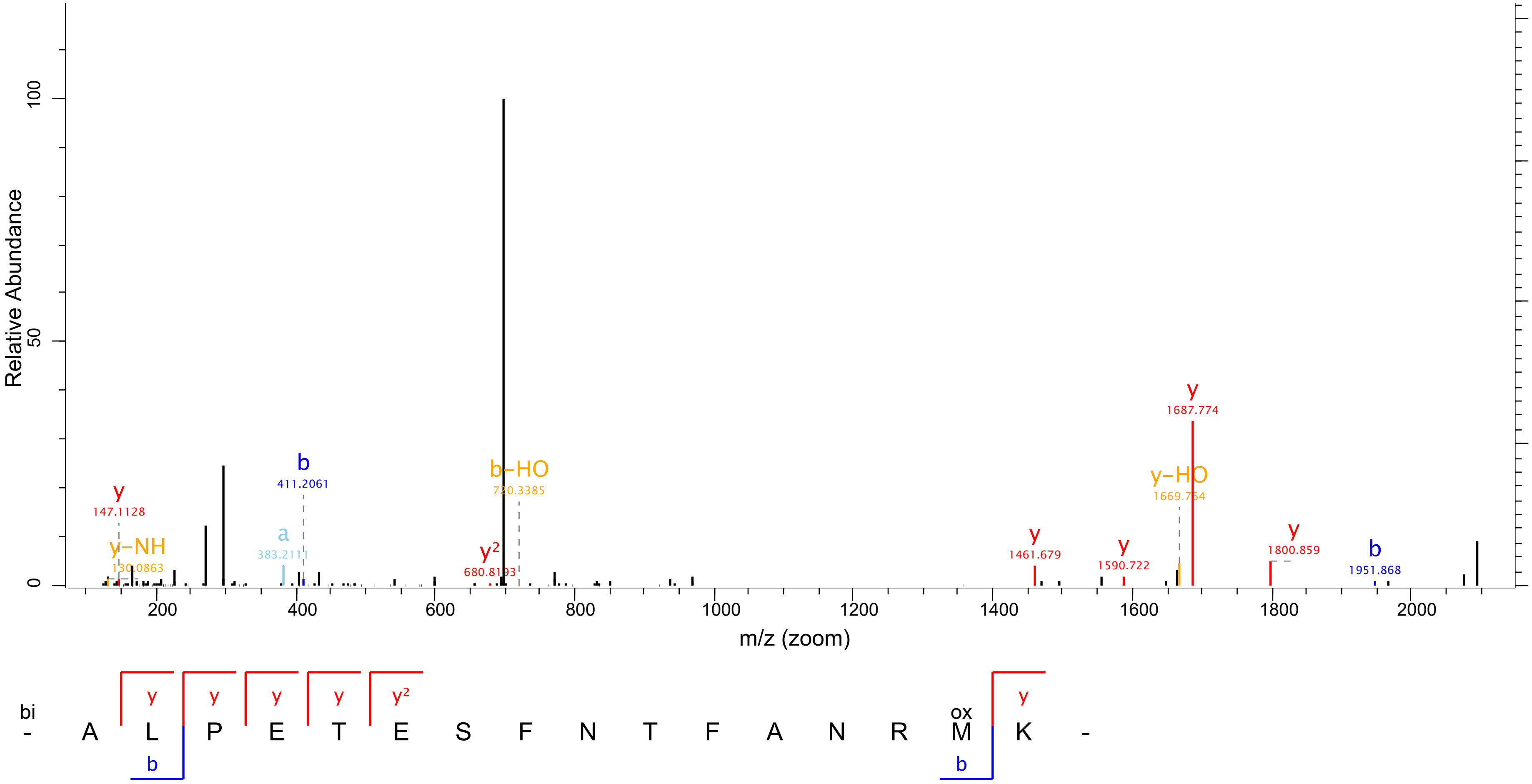

Raw File	Scan	Method	Score	m/z	Gene names
agg_pep_sort_pos_3	21147	FTMS; HCD	196.33	1105.04	TES

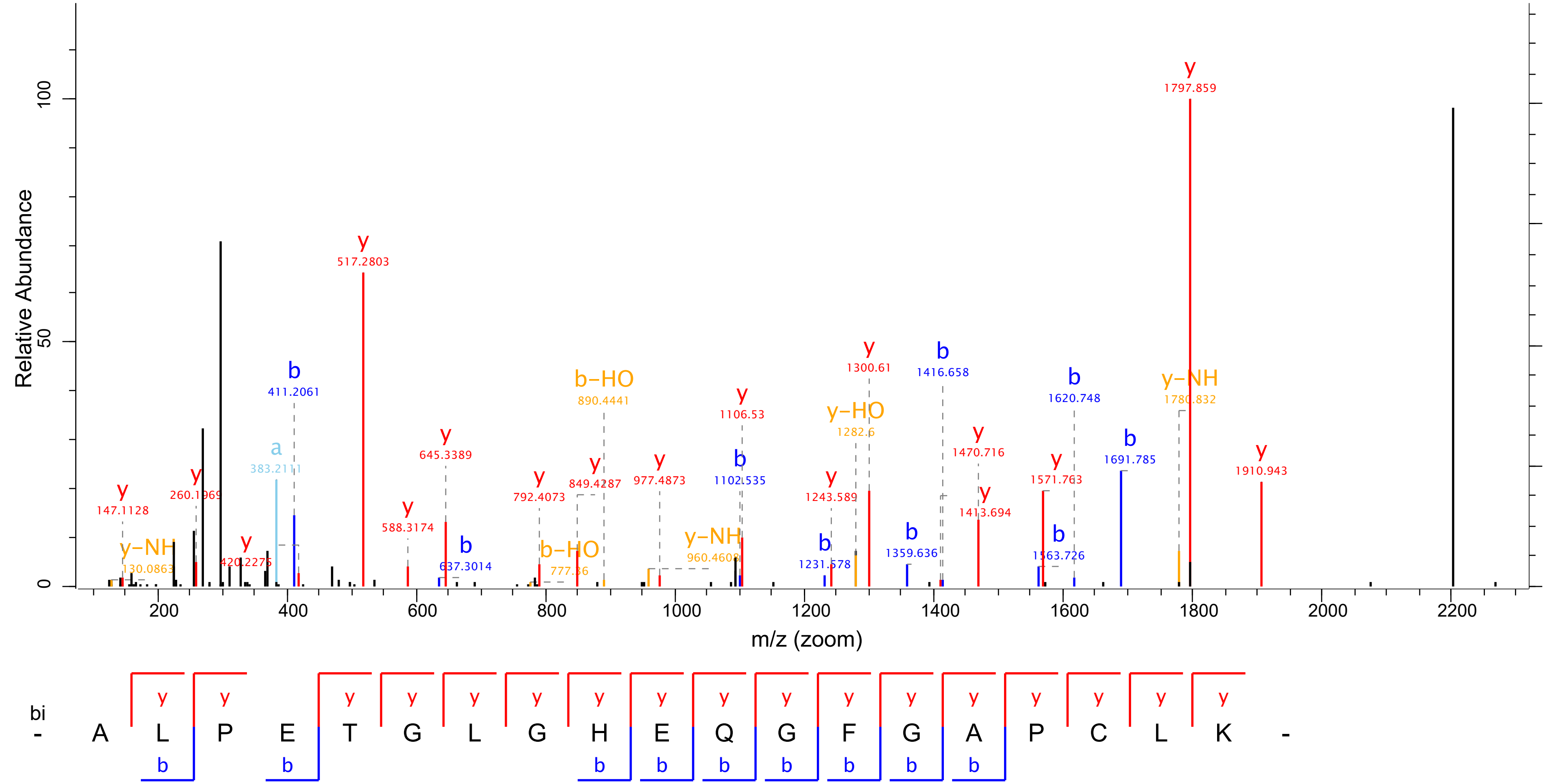

Raw File	Scan	Method	Score	m/z	Gene names
agg_pep_sort_pos_2	17865	FTMS; HCD	105.14	584.3	TEX101;SEZ6L2

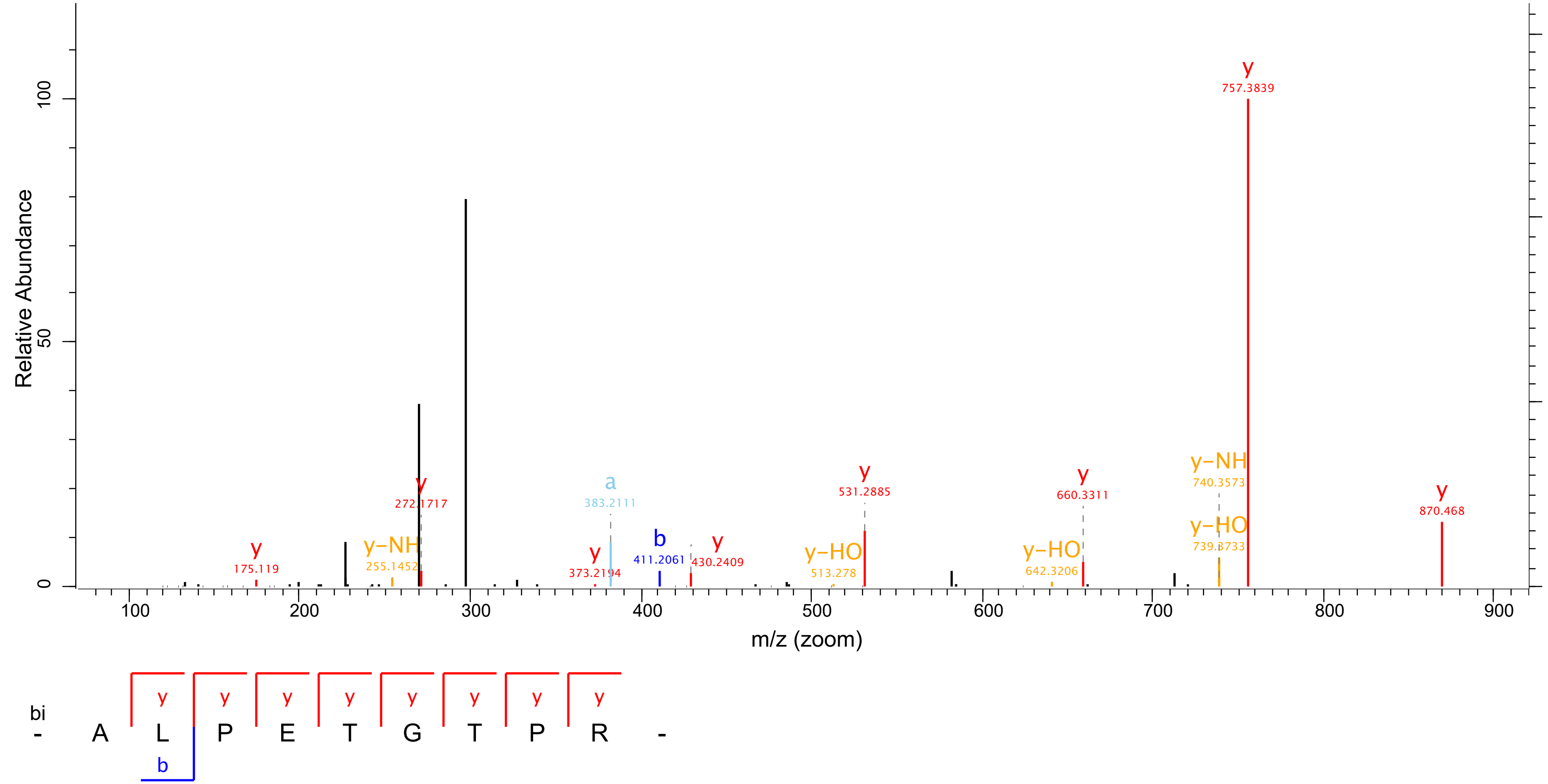

Raw File	Scan	Method	Score	m/z	Gene names
agg_pep_sort_pos_1	17360	FTMS; HCD	62.19	867.06	TIAM1

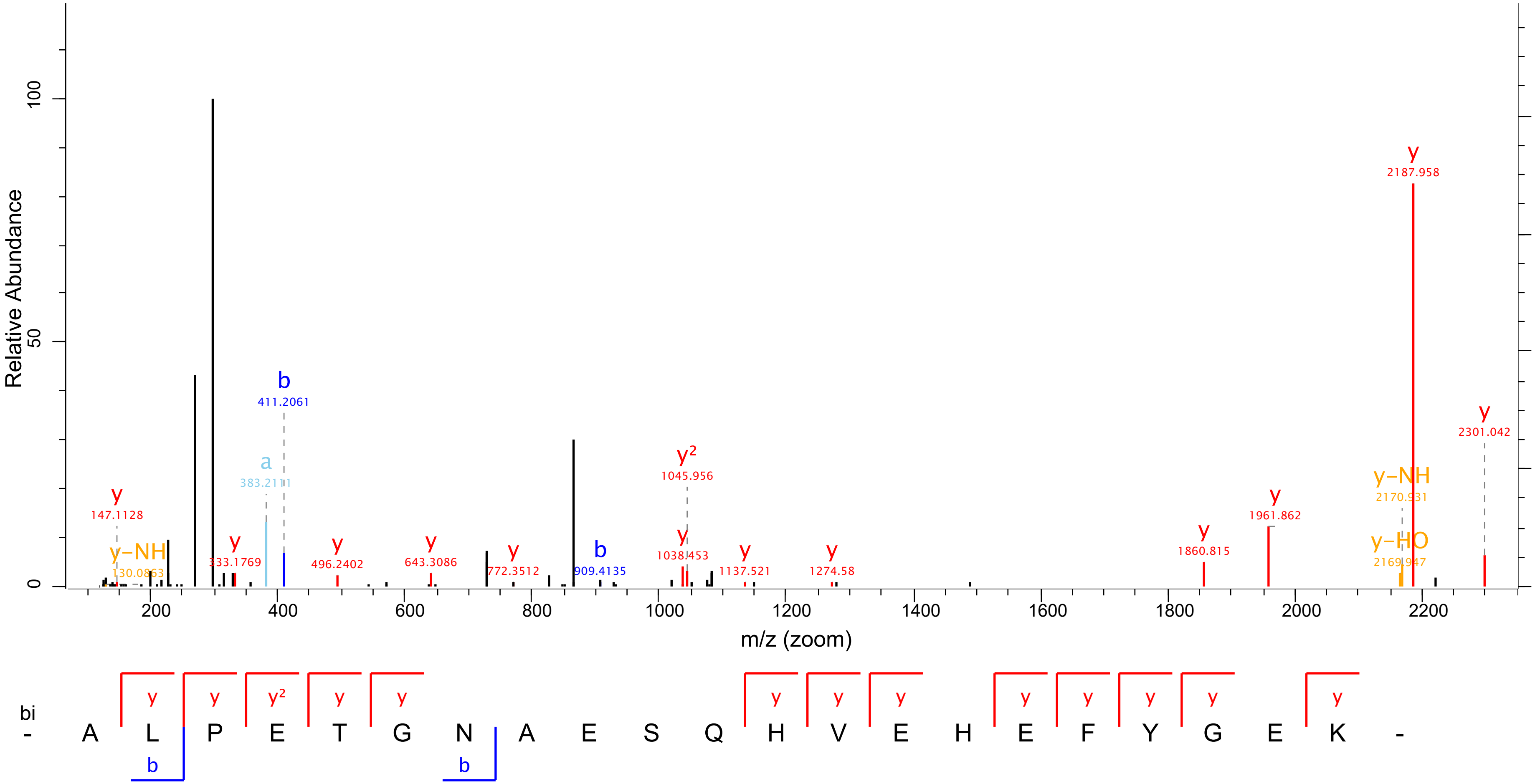

Raw File	Scan	Method	Score	m/z
agg_pep_sort_pos_3	12633	FTMS; HCD	146.07	586.65

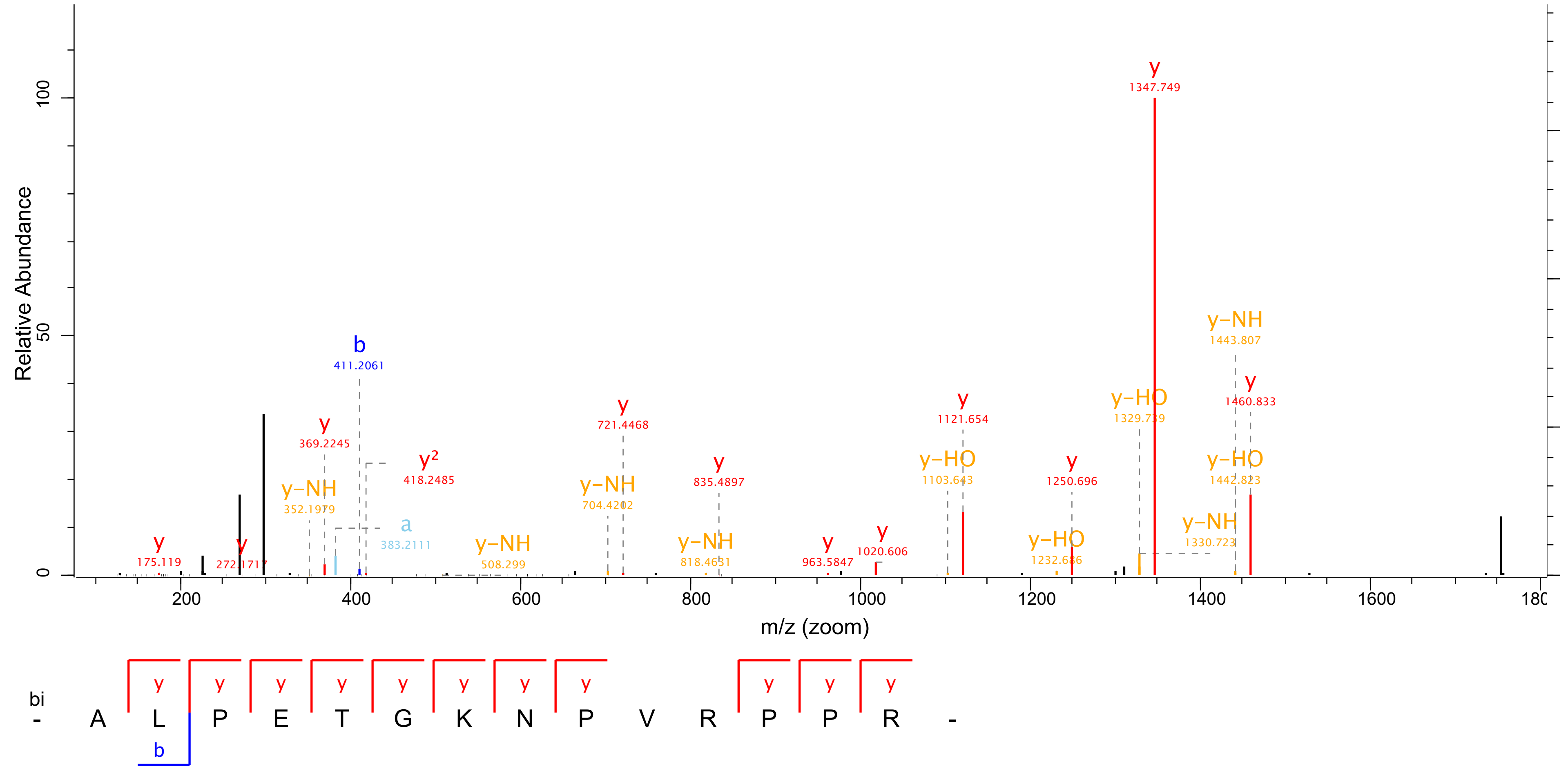

Raw File	Scan	Method	Score	m/z	Gene names
agg_pep_sort_pos_3	18153	FTMS; HCD	53.4	723.36	TMPRSS11A

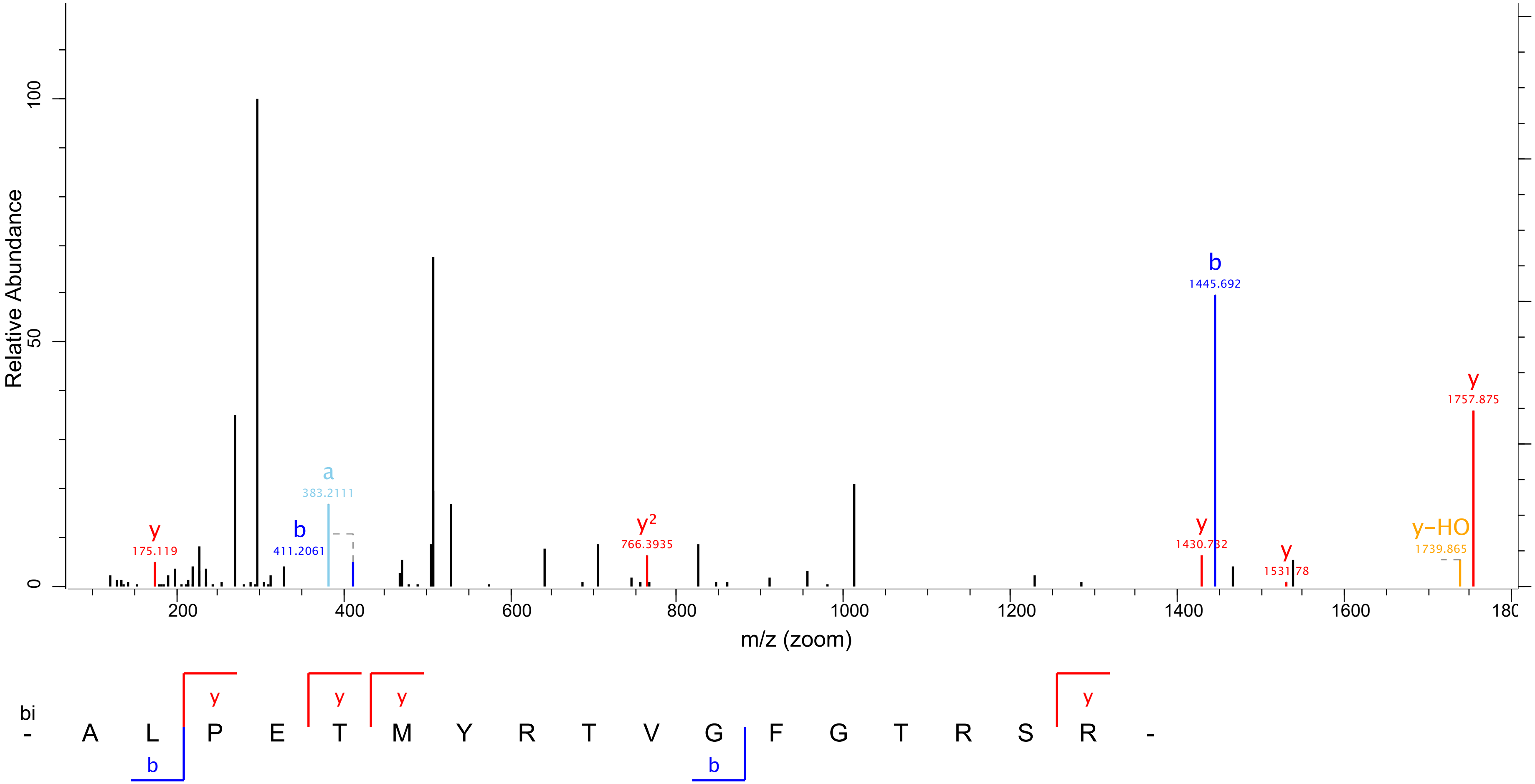

Raw File	Scan	Method	Score	m/z
agg_pep_sort_pos_3	16575	FTMS; HCD	65.56	590.31

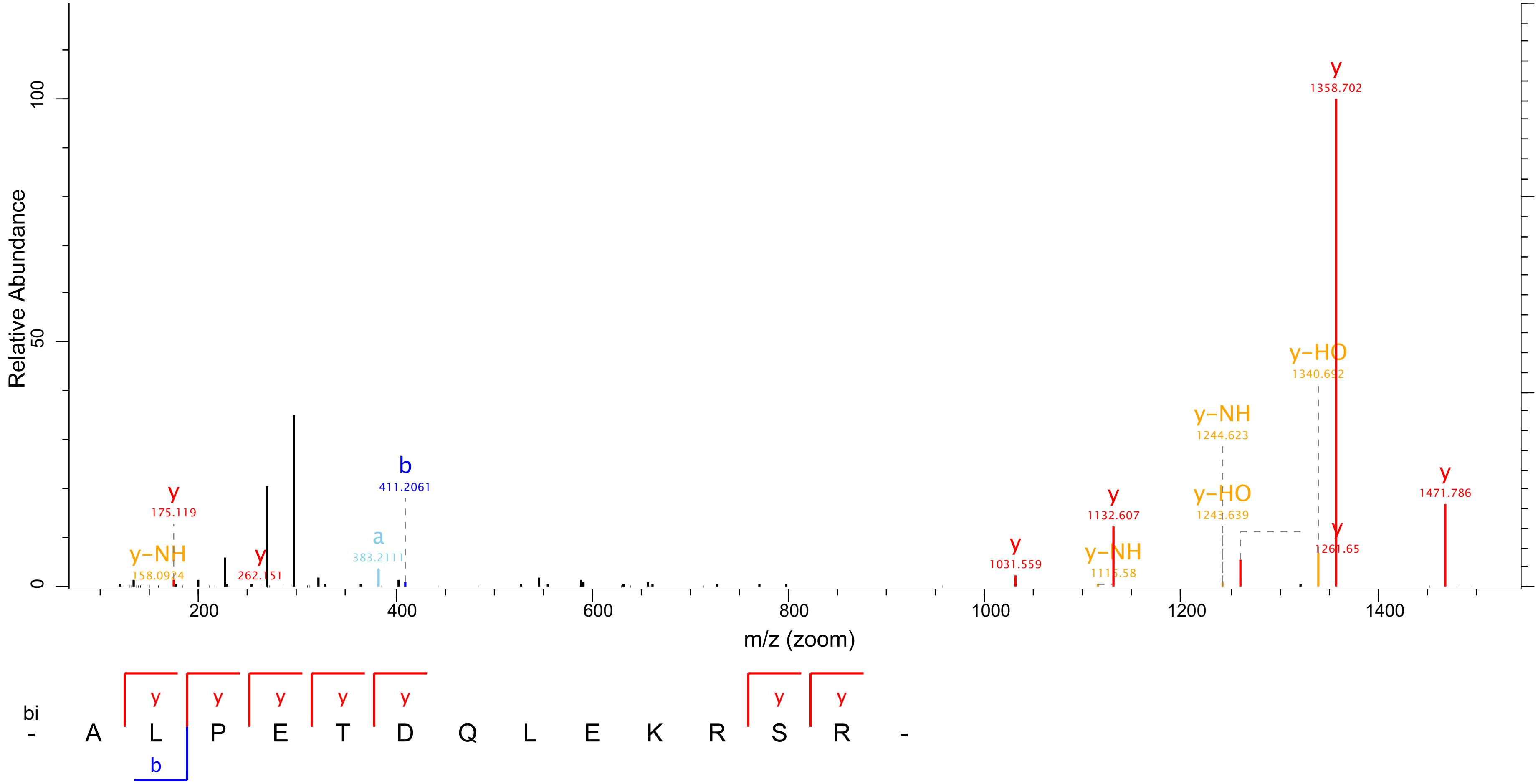

Raw File	Scan	Method	Score	m/z	Gene names
agg_pep_sort_pos_3	16908	FTMS; HCD	57.96	641.36	TPST1

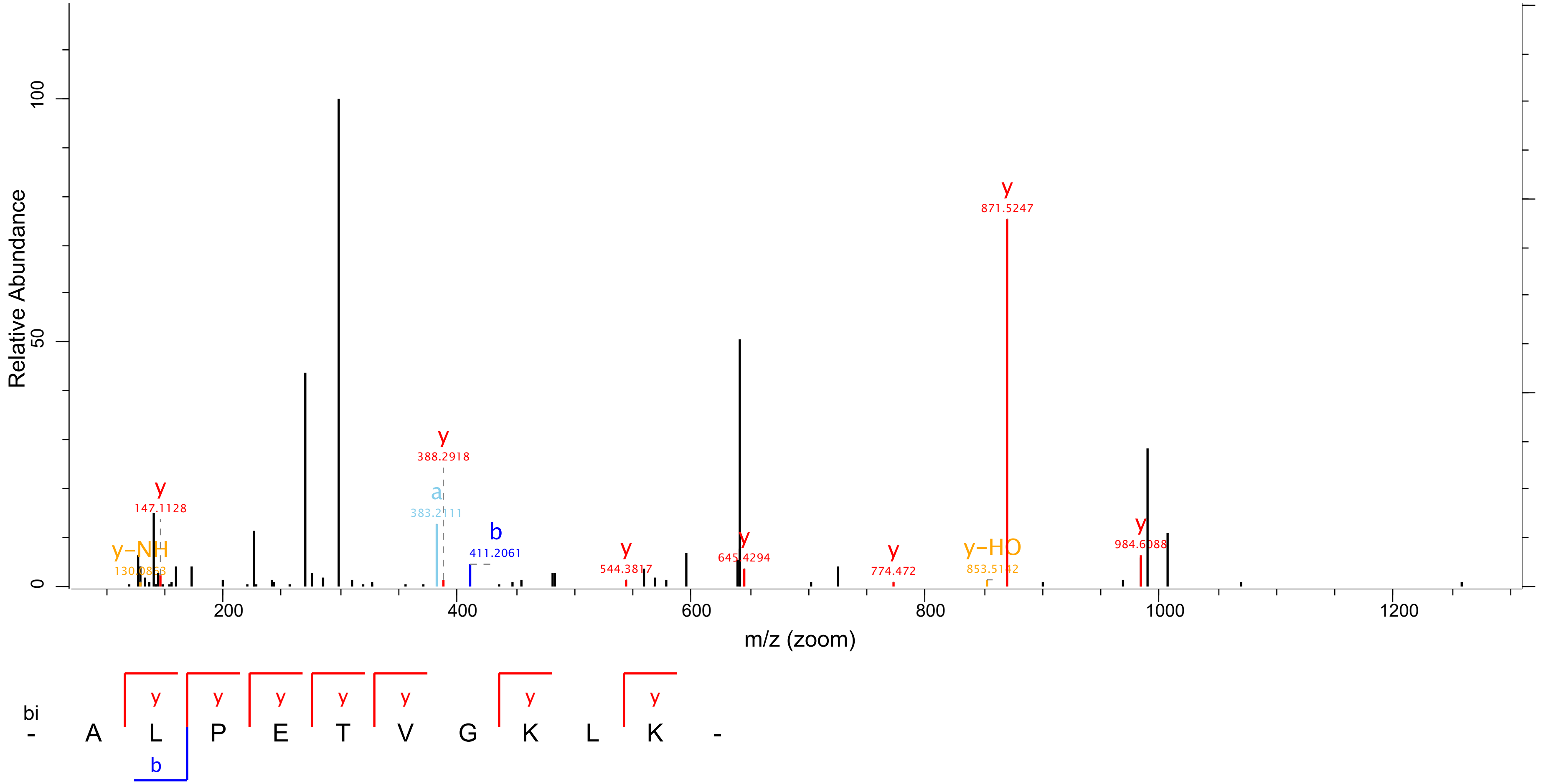

Raw File	Scan	Method	Score	m/z	Gene names
agg_pep_sort_pos_1	17295	FTMS; HCD	69.42	641.37	TPST1

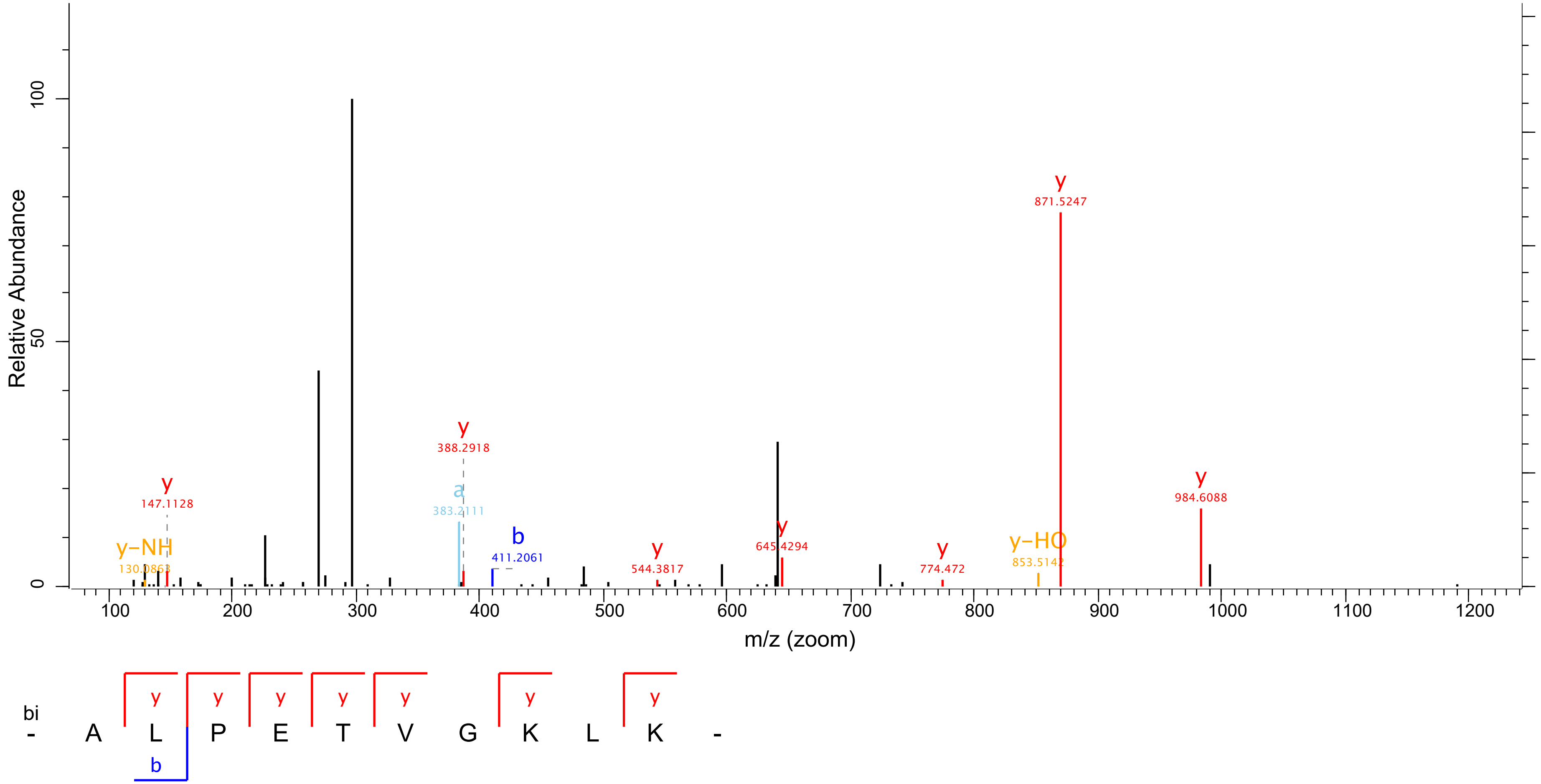

Raw File	Scan	Method	Score	m/z
agg_pep_sort_pos_1	16190	FTMS; HCD	54.81	872.44

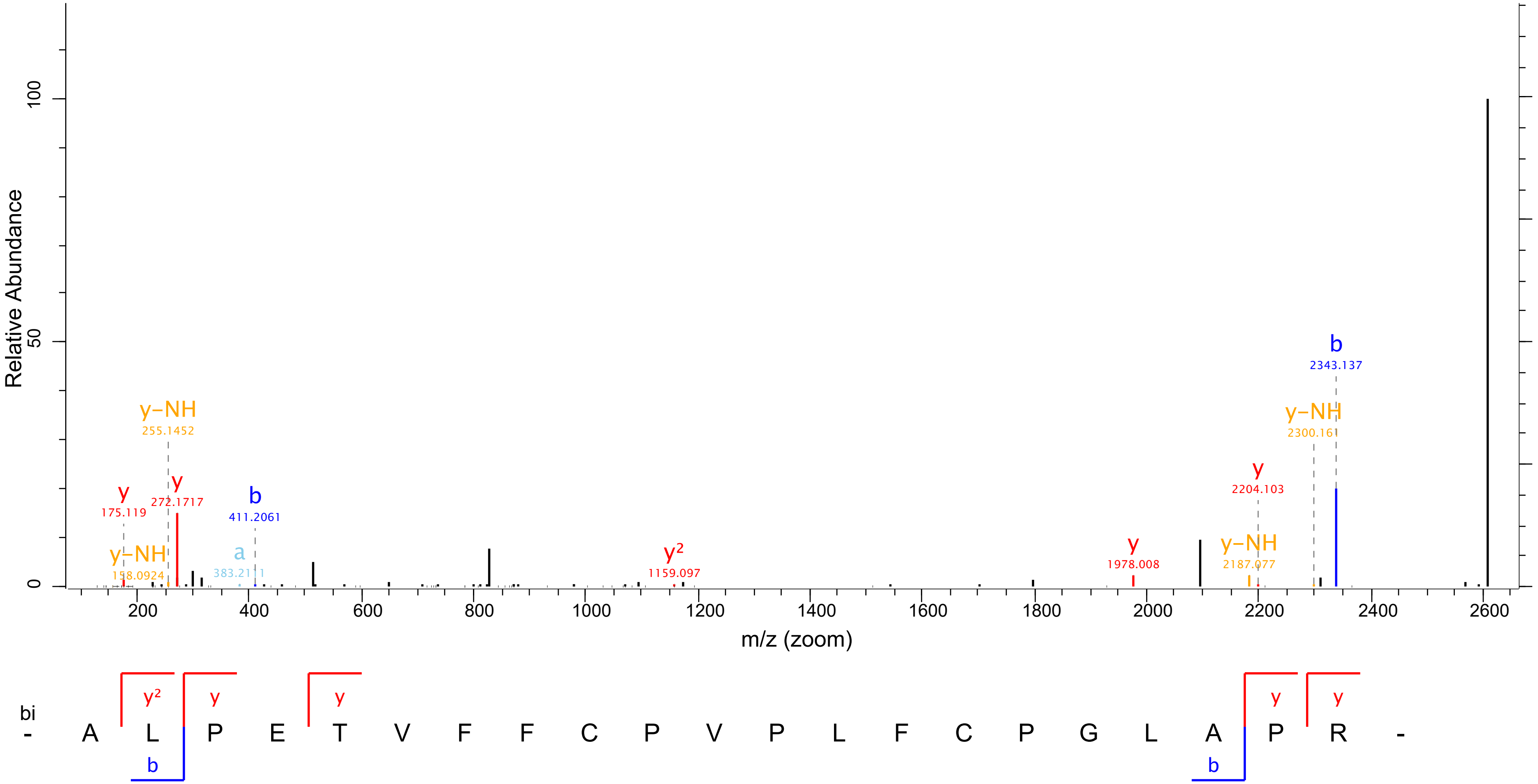

Raw File	Scan	Method	Score	m/z	Gene names
agg_pep_sort_pos_1	16987	FTMS; HCD	57.35	967.02	WASF2

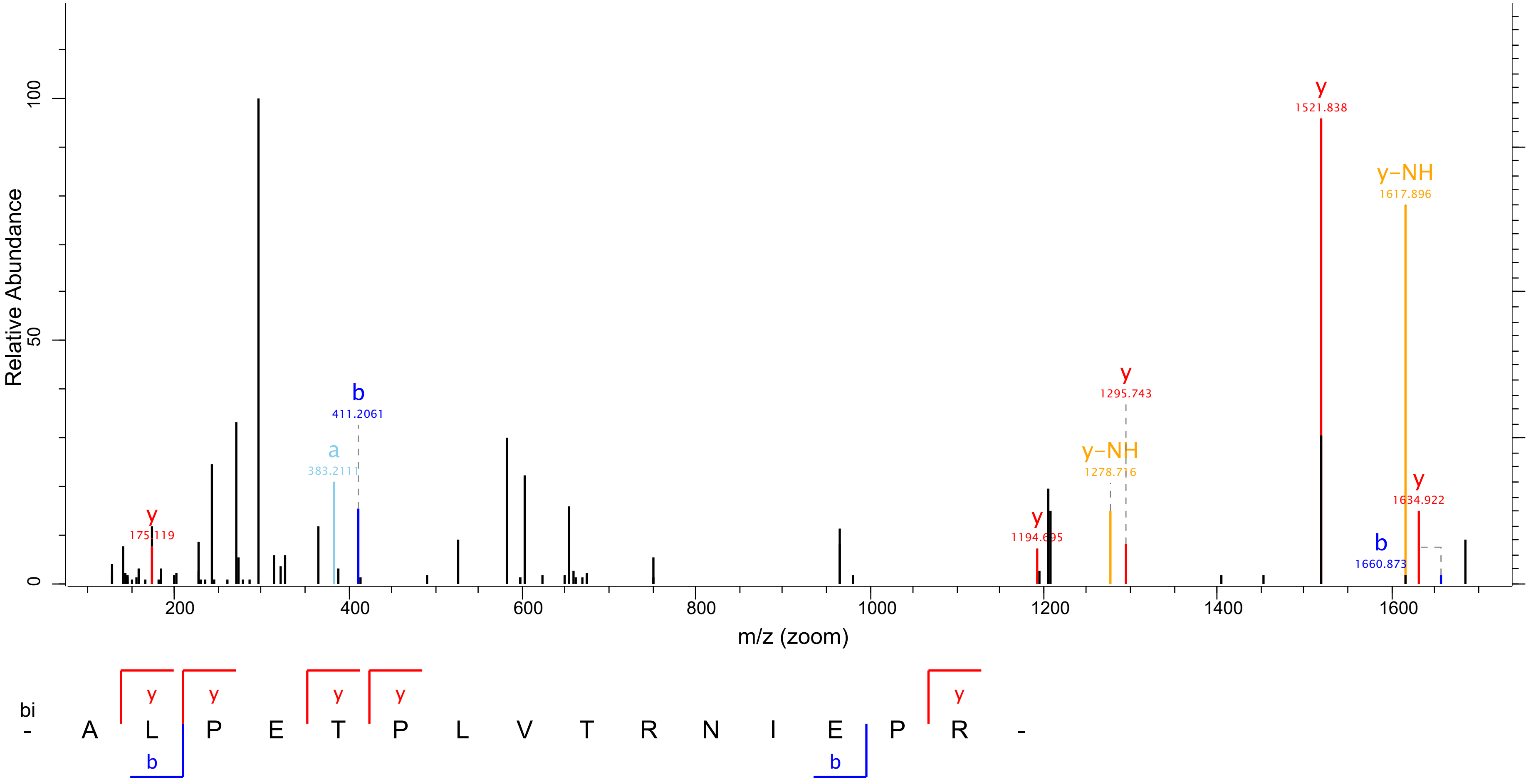

Raw File agg_pep_sort_pos_1 | Scan 12281 | Method FTMS; HCD | Score 57.8 | m/z 524.6

Raw File	Scan	Method	Score	m/z
agg_pep_sort_pos_1	12281	FTMS; HCD	57.8	524.6

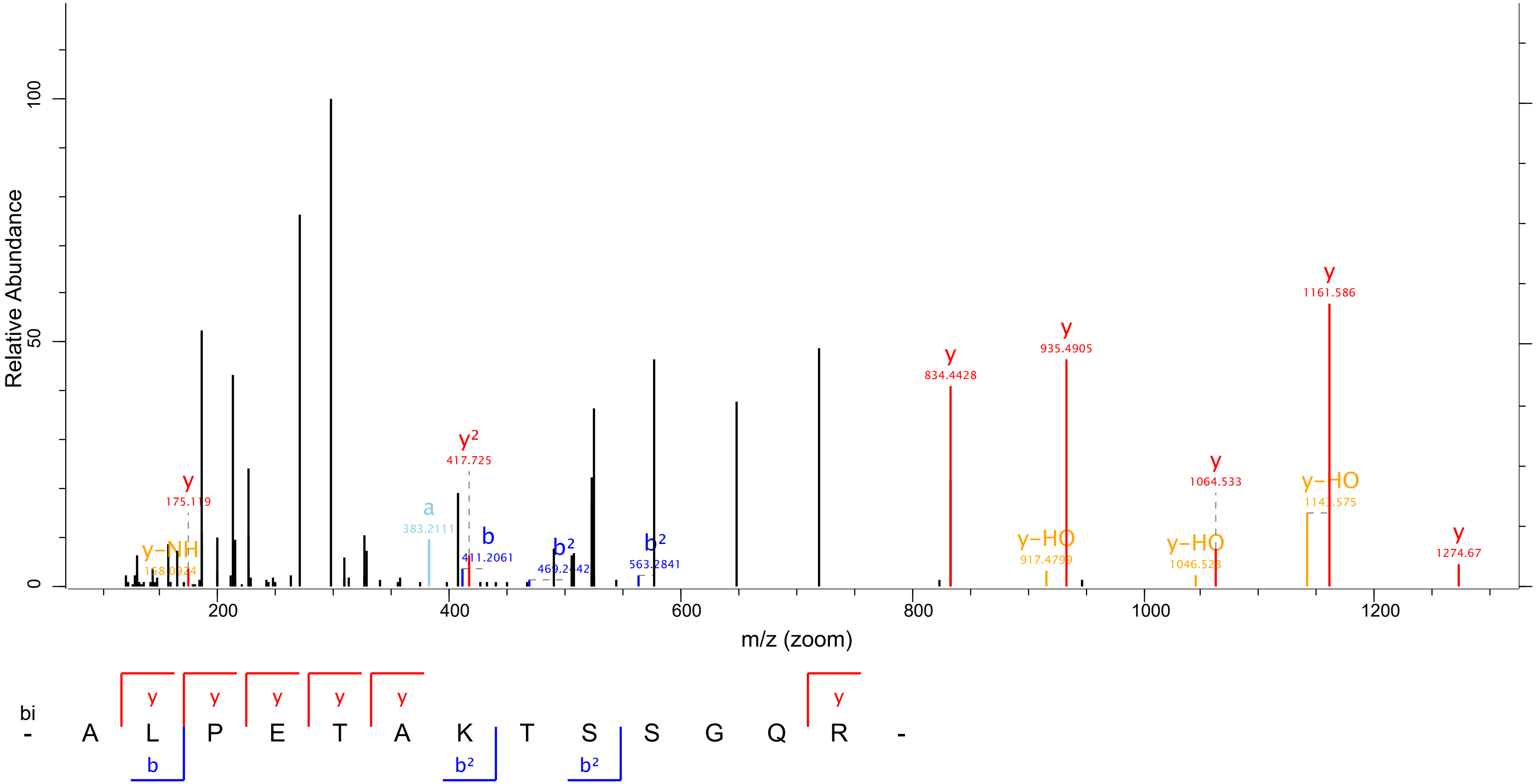

Figure S3. MS/MS spectra of Biotin-ALPET modified peptides (confident assignments) detected using chemoproteomic approach presented in Fig. 3. MS/MS spectra for a given peptide were selected based on the highest Andromeda score. All spectra contain a singly charged diagnostic ion of m/z = 227.0849 that represents the biotin-originated b-type ion. Spectra have been arranged alphabetically according to the gene names returned from the database search.

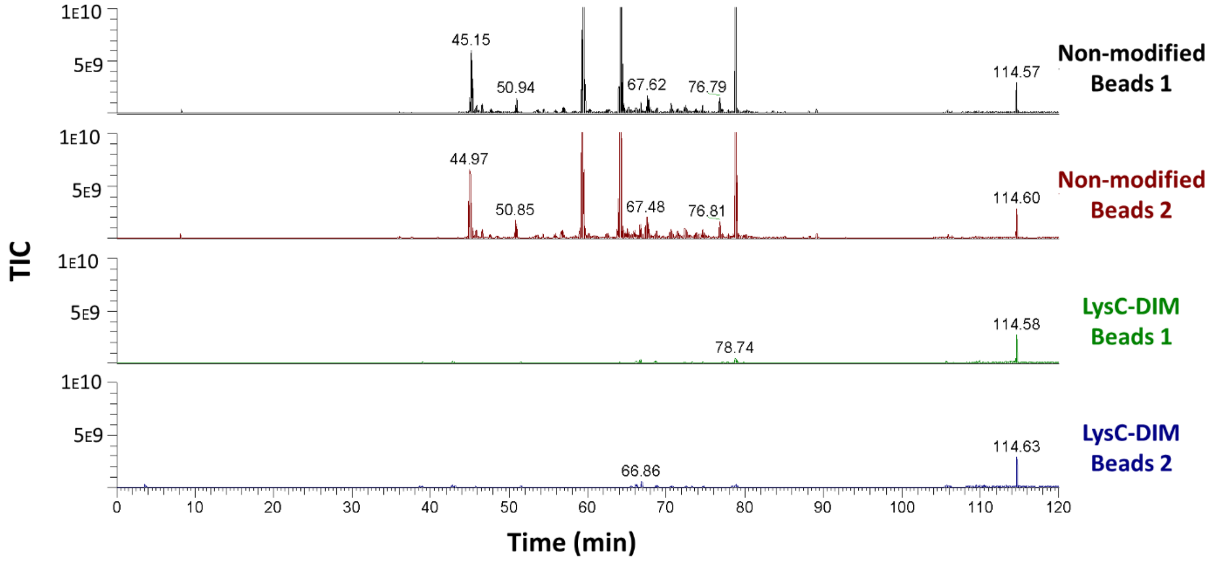

Figure S4. Capping NeutrAvidin lysines by dimethylation followed by a two-step on-bead LysC/off-bead trypsin digestion significantly reduces NeutrAvidin-derived high intensity peaks. Samples containing 200 µg of Biotin-ALPET labeled proteins, originated fromMDA-MB-231 cells, were incubated in duplicate with 200 µL of dimethylated NeutrAvidin agarose beads or with 200 µL of non-derivatized NeutrAvidin agarose beads for 2 h at RT. Samples incubated with dimethylated beads were subjected to 1 h LysC digestion followed by digestion off-bead with trypsin overnight. Samples incubated with non-derivatized beads were subjected to an overnight on-bead digestion with trypsin. The plots show total ion current (TIC) chromatograms recorded for each of the four samples.

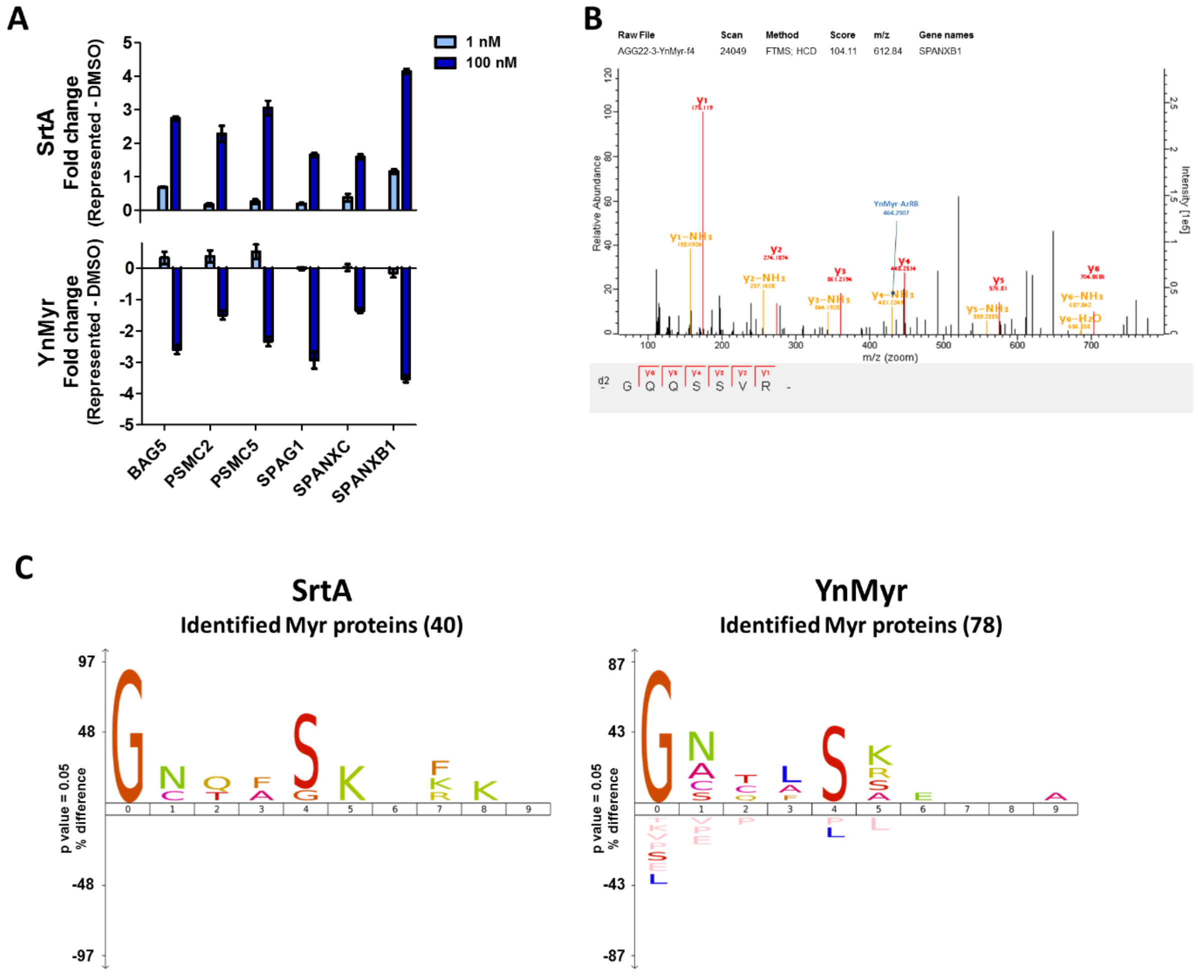

Figure S5. Comparison of SrtA-labeling with YnMyr metabolic tagging to assess NMT activity by protein-enrichment proteomics analysis. (A) Enrichment-derived intensities of significant proteins not previously known to be myristoylated. **(B)** Annotated MS/MS spectrum of SPANXB1 N-terminal peptide showing modification with YnMyr-AzRB. **(C)** Sequence logos of all known myristoylated proteins identified in SrtA and YnMyr protein-based enrichment experiments. Logos were generated with iceLogo (23), using the pre-compiled Swissprot human proteome (accessed on 17/09/2018) as the reference set.

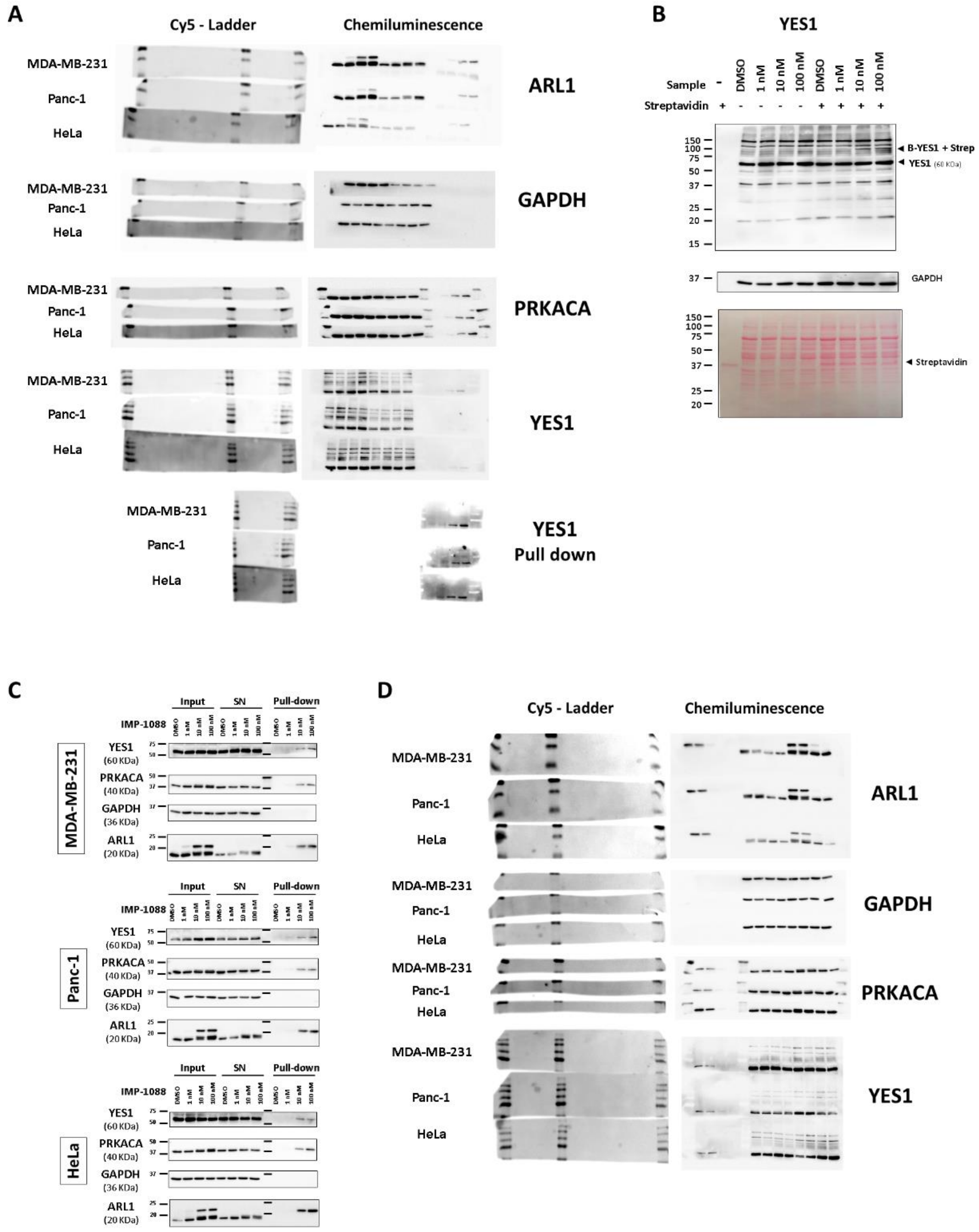

Figure S6. (A) Full membrane view of the western blots shown in Fig. 5A. (B) Streptavidin shift assay followed by western blot analysis against YES1. (C) A second biological replicate of the pull-down western blot analysis of selected myristoylated proteins showing NMTi dose-dependent increase in enrichment for all proteins but loading control (GAPDH). (D) Full membrane view of western blots shown in (C).
